# Transcriptional control of pancreatic cancer immunosuppression by metabolic enzyme CD73 in a tumor-autonomous and -autocrine manner

**DOI:** 10.1038/s41467-023-38578-3

**Published:** 2023-06-08

**Authors:** Tianyu Tang, Xing Huang, Minghao Lu, Gang Zhang, Xu Han, Tingbo Liang

**Affiliations:** 1 Zhejiang Provincial Key Laboratory of Pancreatic Disease, The First Affiliated Hospital, Zhejiang University School of Medicine, 310009 Hangzhou, Zhejiang China; 2 Department of Hepatobiliary and Pancreatic Surgery, The First Affiliated Hospital, Zhejiang University School of Medicine, 310003 Hangzhou, Zhejiang China; 3 Zhejiang Clinical Research Center of Hepatobiliary and Pancreatic Diseases, 310003 Hangzhou, Zhejiang China; 4 The Innovation Center for the Study of Pancreatic Diseases of Zhejiang Province, 310009 Hangzhou, Zhejiang China; 5 Cancer Center, Zhejiang University, 310058 Hangzhou, Zhejiang China

**Keywords:** Cancer metabolism, Cancer immunotherapy, Pancreatic cancer

## Abstract

Cancer cell metabolism contributes to the establishment of an immunosuppressive tumor microenvironment. Aberrant expression of CD73, a critical enzyme in ATP metabolism, on the cell surface results in the extracellular accumulation of adenosine, which exhibits direct inhibitory effects on tumor-infiltrating lymphocytes. However, little is known about the influence of CD73 on negative immune regulation-associated signaling molecules and transduction pathways inside tumor cells. This study aims to demonstrate the moonlighting functions of CD73 in immunosuppression in pancreatic cancer, an ideal model characterized by complex crosstalk among cancer metabolism, immune microenvironment, and immunotherapeutic resistance. The synergistic effect of CD73-specific drugs in combination with immune checkpoint blockade is observed in multiple pancreatic cancer models. Cytometry by time-of-flight analysis shows that CD73 inhibition reduces tumor-infiltrating Tregs in pancreatic cancer. Tumor cell-autonomous CD73 is found to facilitate Treg recruitment, in which CCL5 is identified as a significant downstream effector of CD73 using integrated proteomic and transcriptomic analyses. CD73 transcriptionally upregulates CCL5 through tumor cell-autocrine adenosine–Adora2a signaling-mediated activation of the p38–STAT1 axis, recruiting Tregs to pancreatic tumors and causing an immunosuppressive microenvironment. Together, this study highlights that CD73–adenosine metabolism transcriptionally controls pancreatic cancer immunosuppression in a tumor-autonomous and -autocrine manner.

## Abstract (web summary)

Expression of the metabolic enzyme CD73 has been associated with immunosuppression in cancer. Here the authors show that tumor cell-autonomous CD73 promotes regulatory T-cell accumulation and that CD73 targeting sensitizes pancreatic cancer to PD-1 blockade.

## Introduction

Metabolic reprogramming, a hallmark of tumor cells, plays a critical role in immunological tolerance and immune escape in various malignancies^1,2^. Accumulating evidence has shown that tumor cells inhibit the function of infiltrated lymphocytes via competitive uptake of available nutrients in the microenvironment that are important for the differentiation and activation of immune cells^2^. Tumor cells restrict glucose consumption by T cells via competitive uptake of glucose, thereby inducing T-cell dysfunction and exhaustion^3^. Moreover, high glucose transporter-1 (GLUT1) expression in tumors correlates with decreased T-cell infiltration^4^. Aberrant use of nutrients by tumor cells further leads to the production of numerous metabolites, which profoundly influence infiltrated immune cells. Increased aerobic glycolysis results in lactate accumulation in the microenvironment, which impairs the function of tumor-targeting T cells and NK cells^5,6^. Prostaglandin E2 accumulation in the tumor microenvironment induced by elevated arachidonic acid metabolism in tumors leads to the repolarization of macrophages from the tumor-suppressing M1 phenotype to the tumor-promoting M2 phenotype^7^. The increased metabolites and byproducts resulting from metabolic reprogramming also act as signaling molecules, contributing to the deregulation of immunosuppressive pathways in tumors. NAD+ metabolism regulates programmed cell death 1 (PD-1)-ligand 1 (PD-L1) expression in cancer cells via α-ketoglutarate-mediated epigenetic modifications^8^. Additionally, ROS produced by mitochondrial oxidative phosphorylation (OXPHOS) in cancer cells causes sustained JNK activation, which is linked to both immune-promoting and immunosuppressive roles in various tumors^9,10^.

Considering the large influence of tumor metabolism on the immune system, numerous studies have been conducted to evaluate the therapeutic potential of targeting varying metabolic enzymes in tumor metabolism to elicit sensitization to immunotherapy in various malignancies. Combined treatment with the glutaminase inhibitor CB-839 and PD-1 or cytotoxic T-lymphocyte-associated protein 4 (CTLA4)-targeted therapy increased antigen-specific T-cell infiltration and inhibited tumor growth in a melanoma model^11^. CB-1158, an arginase 1 inhibitor, has shown promising therapeutic effects in blocking myeloid cell-mediated immunosuppression^12^. Combining CB-1158 with checkpoint blockade resulted in enhanced lymphocyte infiltration and increased expression of inflammatory cytokines. In preclinical models of tumors overexpressing indoleamine 2,3-dioxygenase 1 (IDO), blocking hydrocarbon receptors improved the therapeutic efficacy of immune checkpoint blockade by disrupting immune suppression mediated by Tregs and tumor-associated macrophages^13^. The promising results in preclinical models have further led to numerous ongoing trials investigating combinations of metabolic enzyme-targeted therapies and immunotherapies (NCT02771626, NCT04265534, NCT02903914, and NCT03337698). Notably, in 2019, Long et al. reported the first phase III clinical trial of combined treatment with an IDO1 inhibitor and a PD-1 inhibitor in patients with melanoma^14^. Unfortunately, the results showed that the addition of the IDO1 inhibitor failed to improve the efficacy of PD-1-targeted monotherapy. To date, no combined treatment incorporating both metabolic enzyme-targeted therapy and immunotherapy has been approved for the clinical treatment of any type of cancer, possibly because of the complexity of tumor metabolism–immune system interactions and the inadequate understanding of the noncanonical functions of metabolic enzymes. Therefore, further studies investigating the detailed mechanisms of immunosuppressive enzymes and identifying potent metabolic targets for combined treatment with immunotherapy are urgently needed.

CD73, encoded by the *NT5E* gene, is a metabolic enzyme expressed on the cell membranes that convert extracellular adenosine triphosphate (ATP) to adenosine (ADO) in cooperation with CD39^15–19^. Previous studies demonstrated that the CD73/ADO /ADO receptor signaling axis plays critical roles in mediating both biological activities and pathological processes, including heart block, inhibition of inflammation, and attenuation of ischemia, etc^17,20–22^. In the preclinical model, genetic deletion of CD73 in mice was associated with the failure to resolve acute lung injury adequately^23^. In addition, according to previous reports, mice with ADO receptor adora2b depletion experienced more severe pulmonary inflammation when subjected to endotoxin^24^. Upregulation of CD73 expression and deregulation of the ADO signaling pathway have also been observed in various malignancies^15,25^. Previous studies have demonstrated the direct inhibitory effects of ADO on varying tumor-infiltrating immune cells (including lymphocytes and dendritic cells), which is considered the canonical mechanism by which CD73 creates an immunosuppressive microenvironment^23–28^. Recently, in a phase II clinical trial, patients with unresectable lung cancer received a combined PD-L1- and CD73-targeted therapy^29^. CD73 inhibition significantly improved the therapeutic efficacy of immune checkpoint blockade and prolonged the survival of these patients, highlighting CD73 as a promising target for sensitizing tumors to immunotherapy. Notably, recent studies have revealed the moonlighting functions of CD73 in tumors. CD73 serves as a regulator of the tumor-autonomous adenosinergic signaling pathway, promoting tumor growth independent of its canonical effect^30,31^. Ma et al. reported that CD73 promotes epithelial–mesenchymal transition (EMT) and cancer progression through the ADO–Adora2a signaling-mediated RAP1–PI3K–AKT axis^30^. CD73 has also been reported to promote the stemness of tumor cells by transcriptionally upregulating SOX9 expression via the AKT–c-Myc axis^32^. Additionally, activation of tumor-autonomous CD73–Adora2a signaling promotes cancer invasion and metastasis via the PI3K–AKT–mTOR axis^31^. However, the immunosuppressive effect of the CD73-mediated tumor-autonomous adenosinergic signaling pathway remains unclear, largely limiting the clinical translation of CD73-targeted therapeutic strategies.

In this study, we find a synergistic inhibitory effect of co-targeting CD73 and immune checkpoints in pancreatic cancer and demonstrate the significant role of tumor cell-autonomous CD73 and tumor cell-autocrine adenosine–Adora2a signaling in stimulating CCL5 transcription and Treg recruitment.

## Results

### CD73 inhibition sensitizes pancreatic cancer to PD-1 blockade

**Fig. 1:**
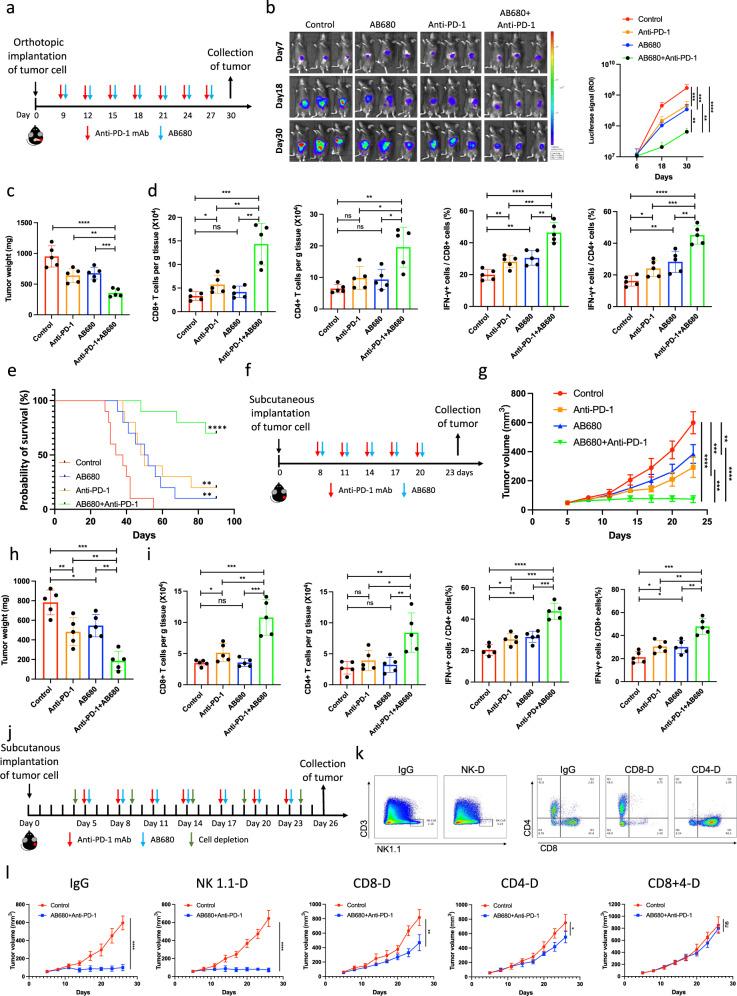
CD73 inhibition sensitizes pancreatic cancer to immunotherapy. **a**–**e** The combination of PD-1-targeted therapy and CD73-targeted therapy inhibits orthotopic pancreatic tumor growth. C57BL/6J mice (*n* = 5) were orthotopically inoculated with KPC^luciferase^ cells and treated with AB680 as well as PD-1-targeted therapy (**a**). Representative bioluminescence images of mice treated as indicated were acquired on days 7, 18, and 30 (*n* = 5) (**b**). Tumor weights (**c**) were individually recorded at the experimental endpoints. Tumor-infiltrated lymphocytes were further quantified (*n* = 5) (**d**). **e** Survival curves of mice with orthotopic pancreatic tumor treated with or without AB680 and PD-1-targeted therapy (*n* = 10). **f**–**i** The combination of PD-1-targeted therapy and AB680 inhibits subcutaneous pancreatic tumor growth. C57BL/6J mice (*n* = 5) were subcutaneously inoculated with KPC cells and treated with an anti-PD-1 Ab as well as AB680 (**f**). Tumor growth curves were generated at the indicated time points (*n* = 5) (**g**). Tumor weights were individually recorded at the experimental endpoints (*n* = 5) (**h**). Tumor-infiltrated lymphocytes were further quantified (*n* = 5) (**i**). **j**–**l** The antitumor response induced by the combination of anti-PD-1 and CD73 inhibition depends on CD4^+^ and CD8^+^ T cells. Subcutaneous tumor-bearing mice (*n* = 5) in which specific cells were depleted with the indicated Abs (anti-CD4, anti-CD8, and anti-NK1.1) were treated with combination therapy (**j**). Cell depletion was confirmed using flow cytometry (**k**). Tumor growth curves were generated at the indicated time points (*n* = 5) (**l**). Results represent means ± SD of one representative experiment in (**b**–**d)**, (**g**–**i**), and (**l**). Kaplan–Meier method and a Gehan–Breslow–Wilcoxon test are indicated in (**e**).*P < 0.05, **P < 0.01, ***P < 0.001 using a two-tailed *t*-test; ns: not significant. The exact *P*-values are shown in the Source Data. Source data are provided as a Source Data file.

**Fig. 2:**
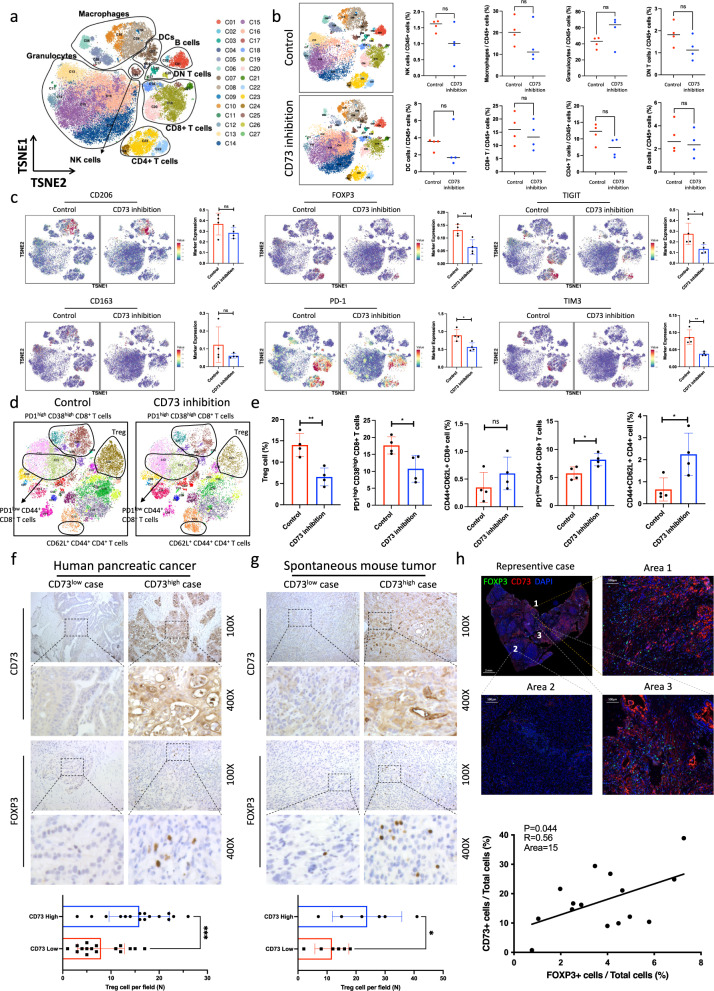
CD73 inhibition reduces tumor-infiltrating Tregs in pancreatic cancer immunotherapy. **a**–**e** CyTOF analysis of tumor-infiltrating immune cells in orthotopic tumors (*n* = 4) after treatment with AB680. Immune subsets were identified based on the tSNE plot (*n* = 4) (**a**). The proportions of different cell subsets were further quantified (**b**). The color-coded expression levels of individual markers in the two groups (*n* = 4) are presented on the tSNE plot and were further quantified (**c**). T-cell subtypes were identified based on the tSNE plot (**d**). The proportions of five T-cell subsets in the control and CD73 inhibition groups were quantified (*n* = 4) (**e**). (**f**–**h**) CD73 expression was positively correlated with Treg accumulation. Quantification of FOXP3 and CD73 IHC staining in pancreatic cancer tissue (*n* = 32; *n*: number of patients) from humans (**f**) and spontaneous pancreatic cancer tissue (*n* = 12; *n*: number of KTC/KPC mice) from mice (Scale bars: 75 μm) (**g**). Representative immunofluorescent detection of CD73 and FOXP3 in human pancreatic cancer tissue (Scale bars: 2 mm, 100 μm) (**h**). All data are representative of three independently performed experiments. Results represent means ± SD of one representative experiment in (**c**) and (**e**–**g**). *P < 0.05, **P < 0.01, ***P < 0.001 using a two-tailed *t*-test; ns: not significant. The Spearman correlations and *P*-values by Spearman’s test are indicated in (**h**). The exact *P*-values are shown in the Source Data. Source data are provided as a Source Data file.

To explore the potential synergistic effect of CD73 inhibition in combination with immunotherapy, AB680, a small-molecule inhibitor targeting CD73, was first combined with an anti-PD-1 Ab in mice orthotopically implanted with KPC cells (Fig. 1a). Compared with monotherapy, the combination therapy resulted in a more significant inhibition of tumor growth, which was quantified by bioluminescence imaging signal intensity (Fig. 1b) and tumor weights (Fig. 1c). Moreover, mice treated with the combination therapy showed similar weights compared to those of mice in the other groups, indicating that the combination treatment was well tolerated (Supplementary Fig. 1a). The potential effect of combination therapy on antitumor immune response was further assessed using flow cytometry (Fig. 2b). The combination therapy significantly increased the infiltration of CD4^+^ T cells, CD8^+^ T cells, as well as the population of IFN-γ^+^ CD4^+^ T cells and IFN-γ^+^ CD8^+^ T cells in the tumor immune microenvironment compared to those in the other groups (Fig. 1d, Supplementary Fig. 3a, b). Additionally, although monotherapy resulted in a slight extension of overall survival in mice, the combined therapeutic approach significantly enhanced the prognosis of mice with pancreatic cancer. (Fig. 1e). In addition, we combined AB680 treatment with PD-1 blockade in mice subcutaneously implanted with KPC cells (Fig. 1f). Monotherapy with AB680 or anti-PD-1 Ab partially slowed tumor progression, while combination therapy significantly increased the inhibitory effect on tumor growth (Fig. 1g, h) with no influence on mouse weight (Supplementary Fig. 1b). Flow cytometric analysis also showed that combination therapy increased the populations of infiltrated CD4^+^ T cells, CD8^+^ T cells, IFN-γ^+^ CD4^+^ T cells and IFN-γ^+^ CD8^+^ T cells in the tumor immune microenvironment (Fig. 1i, Supplementary Fig. 3c, d). To evaluate whether the combination therapy can induce durable immune memory against pancreatic cancer, we resected the tumors from C57BL/6 mice treated with the combination therapy and confirmed the disappearance of tumors after at least 3 months of observation. The mice were then rechallenged with KPC cells as the second implantation and received no additional treatment (Supplementary Fig. 4a). As expected, the growth of KPC tumors in mice was significantly inhibited compared with that in the control group (Supplementary Fig. 4b).

To identify the immune phenotype induced by the combination of CD73 inhibition and PD-1 blockade, we systematically depleted CD8^+^ T-cell, CD4^+^ T-cell, or NK-cell populations before treatment (Fig. 1j, k). Compared with IgG treatment, NK-cell depletion did not alter the inhibitory effect of the combination therapy, CD4^+^ T-cell depletion affected tumor growth more significantly than CD8^+^ T-cell depletion, whereas co-depletion of CD4^+^ T cells and CD8^+^ T cells completely abolished the antitumor effect of the combination therapy (Fig. 1l). Moreover, to evaluate which subsets play a key role in the durable antitumor immune memory induced by combination therapy, mice with depletion of distinct cell populations were rechallenged with KPC cells (Supplementary Fig. 4c). Notably, while depletion of CD8^+^ T cells partially abolished the immune memory induced by combination therapy, CD4^+^ T-cell depletion demonstrated a more significant effect on tumor growth than CD8^+^ T-cell depletion. Co-depletion of CD4^+^ T cells and CD8^+^ T cells completely abolished the durable antitumor immune memory induced by the combination therapy, and NK-cell depletion did not affect tumor growth (Supplementary Fig. 4d–j). Taken together, these results showed that combining CD73 inhibition with PD-1 blockade resulted in a synergistic inhibitory effect on tumor growth, which is T-cell dependent.

### CD73 inhibition reduced Treg accumulation and T-cell exhaustion

To further assess the alterations in the tumor microenvironment induced by CD73 inhibition, we performed cytometry by time-of-flight (CyTOF) analysis of infiltrating immune cells from eight orthotopic tumors in mice treated with and without AB680. An unsupervised density-based clustering technique, t-distributed stochastic neighbor embedding (t-SNE), was applied to analyze alterations in the cell population. The classical immune cell types from 27 clusters were identified based on well-known immune markers (Fig. 2a, Supplementary Fig. 5a). No significant differences were found in the proportions of infiltrated B cells, CD4^+^ T cells, CD8^+^ T cells, DCs, double-negative (DN) T cells, NK cells, macrophages, and granulocytes in tumors (Fig. 2b). Alterations in marker expression induced by CD73 inhibition were also evaluated (Fig. 2c). A decrease in the expression of M2 macrophage markers (CD206 and CD163) was observed, although the difference was not significant. Interestingly, significant decreases in exhausted T-cell markers (PD-1, TIGIT, and TIM3) and a Treg marker (Foxp3) were observed. Considering that the inhibitory effect induced by the combination therapy was completely T-cell dependent, we further reclustered CD3^+^ T cells based on the t-SNE analysis. As expected, CD73 inhibition resulted in very large alterations in T-cell subsets (Fig. 2d, e, Supplementary Fig. 5b). Notably, CD73 inhibition resulted in a decreased infiltration of Treg cells (CD4^+^ CD25^+^ Foxp3^+^). In addition, mice treated with CD73 inhibition therapy showed a reduced generation of exhausted CD8^+^ T cells (CD8^+^ PD-1^high^ CD38^+^). Moreover, compared to mice in the control group, mice in the CD73 inhibition group exhibited an expanded population of PD-1^low^ T cells (CD8^+^ PD-1^low^) and an increased proportion of central memory T cells (CD62L^+^ CD44^+^). These data suggested that CD73-targeted therapy may result in the inhibition of Treg accumulation in tumors, which further leads to enhanced effector T-cell activation and reduced generation of exhausted T cells.

Notably, immunohistochemical (IHC) staining of 32 pancreatic cancer patient samples (Fig. 2f) and 12 samples of spontaneous pancreatic cancer in mice (Fig. 2g) revealed that higher expression levels of CD73 in tumors were associated with increased tumor infiltration of Tregs. In addition, immunofluorescence staining of the samples demonstrated that Tregs accumulated mainly in areas with high CD73 expression in pancreatic cancer (Fig. 2h). Taken together, these data indicated that CD73 expression in pancreatic cancer may play a critical role in the regulation of Treg accumulation.

### Pancreatic cancer cell-autonomous CD73 regulates Treg recruitment

**Fig. 3:**
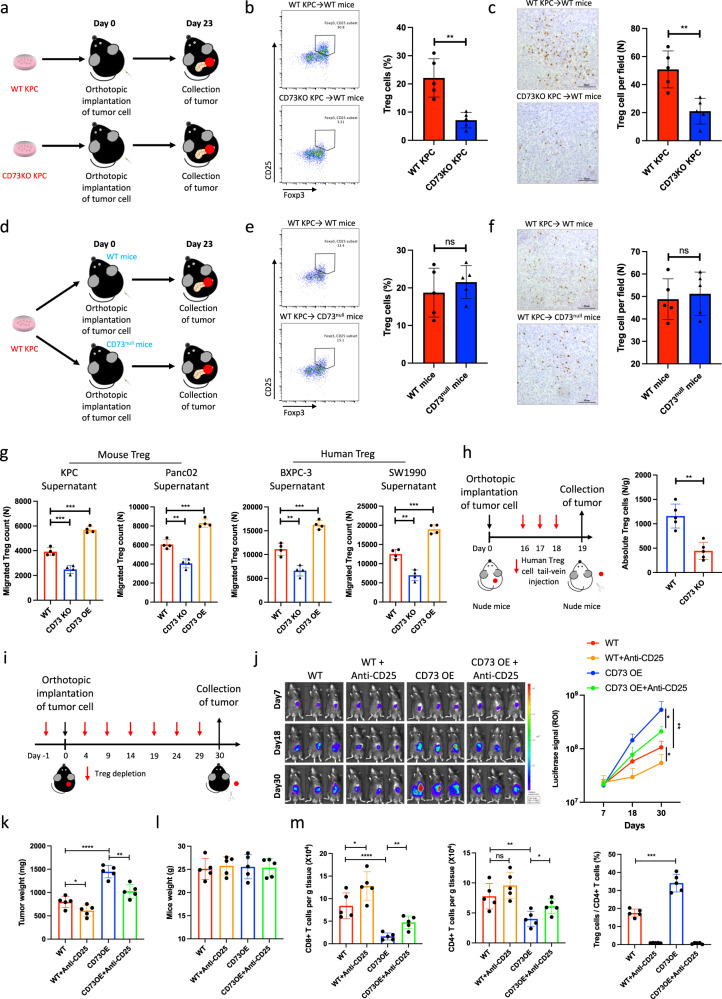
Tumor cell-autonomous CD73 facilitates Treg recruitment into the pancreatic cancer microenvironment. **a**–**f** Depletion of CD73 on orthotopic tumor cells reduced Treg accumulation in pancreatic cancer. WT C57BL/6J mice (*n* = 5) were orthotopically inoculated with WT KPC cells and CD73 KO KPC cells (**a**). Treg accumulation in tumors (*n* = 5) was analyzed and quantified by flow cytometry (**b**) and IHC staining (Scale bars: 50 μm) (**c**). WT C57BL/6J mice and CD73^null^ C57BL/6J mice (*n* = 5) were orthotopically inoculated with WT KPC cells (**d**). Treg accumulation in tumors (*n* = 5) was analyzed and quantified by flow cytometry (**e**) and IHC staining (Scale bars: 50 μm) (**f**). **g** Isolated mouse and human Tregs were incubated with culture medium supernatant from WT, CD73KO, and CD73OE KPC, Panc02, BXPC-3, and SW1990 cells, and the migrated Tregs were further quantified using flow cytometry (*n* = 4). **h** Nude mice (*n* = 5) were orthotopically inoculated with WT and CD73 KO BXPC-3 cells and were injected with human Tregs via the tail vein at indicated time points. Treg accumulation in tumors was analyzed and quantified by flow cytometry. **i**–**m** Depletion of Tregs partially abolished CD73 overexpression-induced orthotopic tumor progression. WT C57BL/6J mice (*n* = 5) were orthotopically inoculated with WT KPC cells and CD73 OE KPC cells (**i**). Tumor growth curves were generated at the indicated time points (*n* = 5) (**j**). Tumor weights were individually recorded at the experimental endpoints (*n* = 5) (**k**). Tumor weights were individually recorded at the experimental endpoints (*n* = 5) (**l**). Tumor-infiltrated CD8^+^ T cells, CD4^+^ T cells, and Tregs were further quantified (*n* = 5) (**m**). Results represent means ± SD of one representative experiment in (**b**, **c**), (**e–h**), and (**j–m**). *P < 0.05, **P < 0.01, ***P < 0.001 using a two-tailed *t*-test; ns: not significant. The exact *P*-values are shown in the Source Data. Source data are provided as a Source Data file.

CD73 is extensively expressed in different cell types in the tumor immune microenvironment, including tumor cells, Tregs, CD8^+^ T cells, and macrophages^16,33^. To identify the cell subset with a critical role in CD73-mediated Treg accumulation, we generated both CD73 knockout (KO) KPC cells and CD73^null^ C57BL/6J mice, which were confirmed by immunoblotting and multi-color IHC (Supplementary Fig. 6a–d). First, wild-type (WT) KPC and CD73 KO KPC cells were orthotopically implanted into WT C57BL/6J mice (Fig. 3a). Flow cytometry (Fig. 3b) and IHC staining (Fig. 3c) showed that the depletion of CD73 in tumor cells significantly inhibited Treg accumulation. To investigate whether CD73 depletion in the tumor immune microenvironment has the same effect on the inhibition of Tregs, WT KPC cells were orthotopically implanted into both WT mice and CD73^null^ C57BL/6J mice (Fig. 3d). However, flow cytometry (Fig. 3e) and IHC staining (Fig. 3f) showed that CD73 depletion in the tumor immune microenvironment did not affect Treg accumulation in pancreatic cancer.

Increased Treg accumulation may be mediated by multiple mechanisms, including the conversion of CD4^+^CD25^−^ T cells, proliferation of preexisting Tregs, and enhanced recruitment of Tregs. To identify the specific mechanism, we first cocultured CD4^+^CD25^−^ T cells with WT, CD73-overexpressing (OE), and CD73 KO pancreatic cancer cells, and cells treated with TGF-β were used as the positive control. No significant differences were found among the WT, CD73 OE, and CD73 KO groups, while enhanced conversion from CD4^+^CD25^−^ T cells to Tregs was observed in the TGF-β group (Supplementary Fig. 7a–d). Next, we cocultured CD4^+^ CD25^+^ Tregs with WT, CD73 OE, or CD73 KO pancreatic cancer cells. The cell proliferation capacity was assessed based on EdU incorporation analysis, and cells treated with IL-2 were used as the positive control. No significant difference was found among the WT, CD73 OE, and CD73 KO groups, whereas increased proliferation of Tregs was observed in the IL-2 group (Supplementary Fig. 7e–h). Finally, an in vitro T-cell migration assay based on the Transwell model was used to assess the recruitment of Tregs to pancreatic cancer cells. CD73 overexpression in KPC, Panc02, SW1990, and BXPC-3 cells significantly enhanced while CD73 depletion in these cells inhibited Treg migration, respectively (Fig. 3g). BXPC-3 cells with or without CD73 depletion were orthotopically implanted into nude mice to evaluate Treg recruitment in vivo. Nude mice were injected with human Treg cells via the tail vein for three consecutive days. On the fourth day, the tumors were harvested and further analyzed for Treg infiltration. Flow cytometric analysis demonstrated that CD73 depletion significantly inhibited the recruitment of human Tregs to BXPC-3 tumors in nude mice (Fig. 3h).

To assess whether tumor cell-autonomous CD73 promotes tumor progression in a Treg-dependent manner, both WT and CD73OE KPC cells were orthotopically implanted into C57BL/6J mice treated with or without anti-CD25 Ab for Treg depletion (Fig. 3i). Interestingly, Treg depletion significantly inhibited CD73 overexpression-induced tumor progression (Supplementary Fig. 3j, k). No statistical differences were found between the groups for the mice weight (Fig. 3l). In addition, Treg depletion largely abolished CD73 overexpression-induced inhibition of CD8^+^ T-cell and CD4^+^ T-cell infiltration and activation (Fig. 3m, Supplementary Fig. 8a–c). Taken together, these data indicated that pancreatic cancer cell-autonomous CD73 inhibits anticancer immunity and promotes tumor progression through enhanced recruitment of Tregs.

### CCL5 is identified as a critical effector in CD73-mediated Treg recruitment in pancreatic cancer

**Fig. 4:**
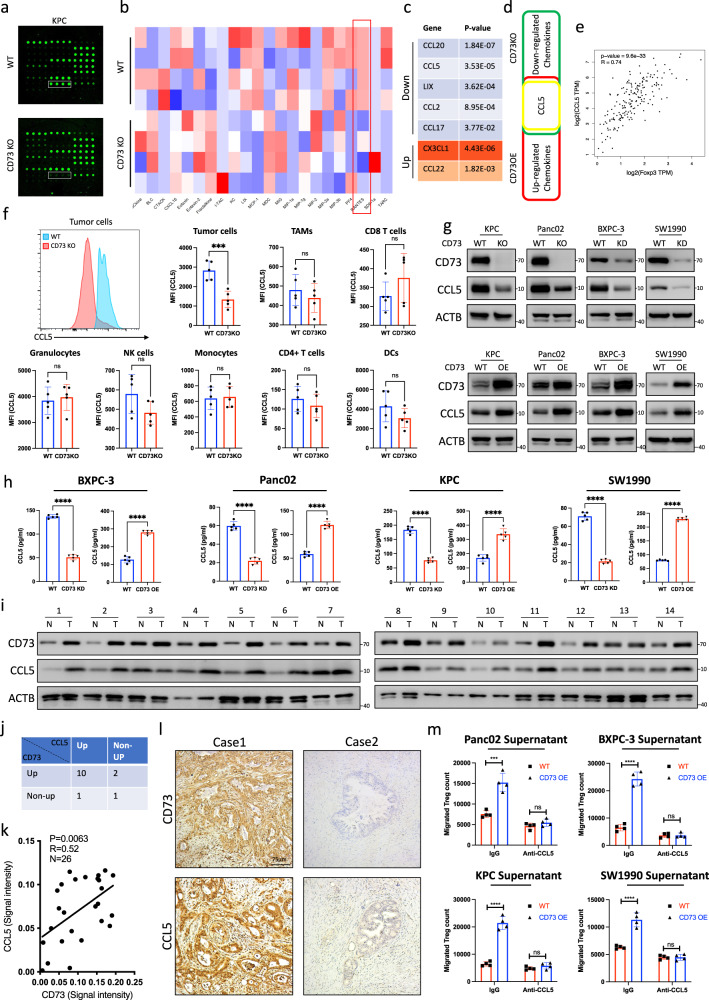
CD73 upregulates CCL5 expression for Treg recruitment in pancreatic cancer. **a**–**d** CD73 regulates chemokine expression. Differential expression of 25 chemokines was detected using chemokine arrays based on culture medium supernatant from WT and CD73 KO KPC cells (*n* = 4). Representative image of chemokine detection in WT and CD73 KO KPC cells (**a**). Heatmap (**b**) and *P*-values (**c**) of the detected chemokines. CCL5 was identified based on the intersection of the downregulated chemokines induced by CD73 depletion and the upregulated chemokines induced by CD73 overexpression (**d**). **e** Positive correlation of CCL5 with FOXP3. The correlation between CCL5 and FOXP3 was analyzed according to the pancreatic datasets in TCGA. **f** Representative images and further quantification of flow cytometric analysis of CCL5 expression in tumor cells, TAMs (tumor-associated macrophages), CD8^+^ T cells, granulocytes, NK cells, monocytes, CD4^+^ T cells, and DCs (dendritic cells) in WT and CD73KO tumors collected from immunocompetent mice (*n* = 5). **g**, **h** Maintenance of CCL5 expression by CD73 in multiple pancreatic cancer cell lines. Immunoblot analysis of CCL5 expression in CD73 KD/KO pancreatic cancer cells and cells overexpressing CD73 (**g**). The alteration of CCL5 secretion was also confirmed using ELISA in multiple pancreatic cancer cell lines (*n* = 5) (**h**). **i**, **j** CD73 and CCL5 expression in paired clinical tissue samples was evaluated using immunoblotting (**i**) and further quantified (**j**). **k**, **l** Statistical results (**k**) and representative images (**l**) of CD73 and CCL5 IHC staining in pancreatic cancer tissue (*n* = 26; *n*: numbers of patients) (Scale bars: 75 μm). **m** Mouse and human Tregs were incubated with culture medium supernatant from WT and CD73OE pancreatic cells treated with or without anti-CCL5 Abs, and the migrated Tregs were further quantified using flow cytometry (*n* = 4). All data are representative of three independently performed experiments. Results represent means ± SD of one representative experiment in (**f**), (**h**), and (**m**). *P < 0.05, **P < 0.01, ***P < 0.001 using a two-tailed *t*-test; ns: not significant. The Spearman correlations and *P*-values by Spearman’s test are indicated in (**e**, **k**). The exact *P*-values are shown in the Source Data. Source data are provided as a Source Data file.

Chemokines are among the most critical molecules that regulate the infiltration of Tregs in various malignancies^34,35^. Through screening of numerous chemokines, we found that CD73 depletion in KPC cells led to the downregulation of CCL5 expression (Fig. 4a–d) and that CD73 overexpression in Panc02 cells resulted in upregulated CCL5 (Supplementary Fig. 9a–c). Previous studies have shown that CCL5 secreted from tumor cells results in increased infiltration of Tregs in multiple cancers^36,37^. In addition, data from TCGA demonstrated that CCL5 expression positively correlated with FOXP3 expression in pancreatic cancer (Fig. 4e). According to previous reports, CCL5 was reported to be expressed by macrophages, NK cells, activated CD8 T cells, and tumor cells, etc. Therefore, we evaluate the expression level of CCL5 on different cell populations using flow cytometric analysis. Results showed that depletion of CD73 only leads to the downregulation of CCL5 in tumor cells rather than other cell populations (Fig. 4f, Supplementary Fig. 9d). We further evaluated the alterations in CCL5 expression levels in distinct pancreatic cell lines with CD73 depletion or overexpression. In Panc02, BXPC-3, SW1990, and KPC cells, CD73 depletion resulted in the downregulation of CCL5, whereas overexpression of CD73-induced upregulation of CCL5 (Fig. 4g). Using enzyme-linked immunosorbent assay (ELISA), we confirmed the increased secretion of CCL5 in the CD73 OE cells and reduced secretion of CCL5 in CD73KD and KO cells compared with the corresponding WT cells (Fig. 4h). Furthermore, the positive correlation between CD73 and CCL5 expression was confirmed by immunoblotting in 14 paired clinical tissue samples (Fig. 4i, j) and IHC staining in 26 tumor samples (Fig. 4k, l). Flow cytometry analysis confirmed high expression of CCR5 in Tregs (Supplementary Fig. 9e). Moreover, in both Panc02, BXPC-3, SW1990, and KPC cells, blocking CCL5 with a neutralizing Ab largely abolished CD73 overexpression-induced Treg migration (Fig. 4m).

### Tumor cell-autocrine adenosine–Adora2a signaling is required for the transcriptional upregulation of CCL5 by CD73

**Fig. 5:**
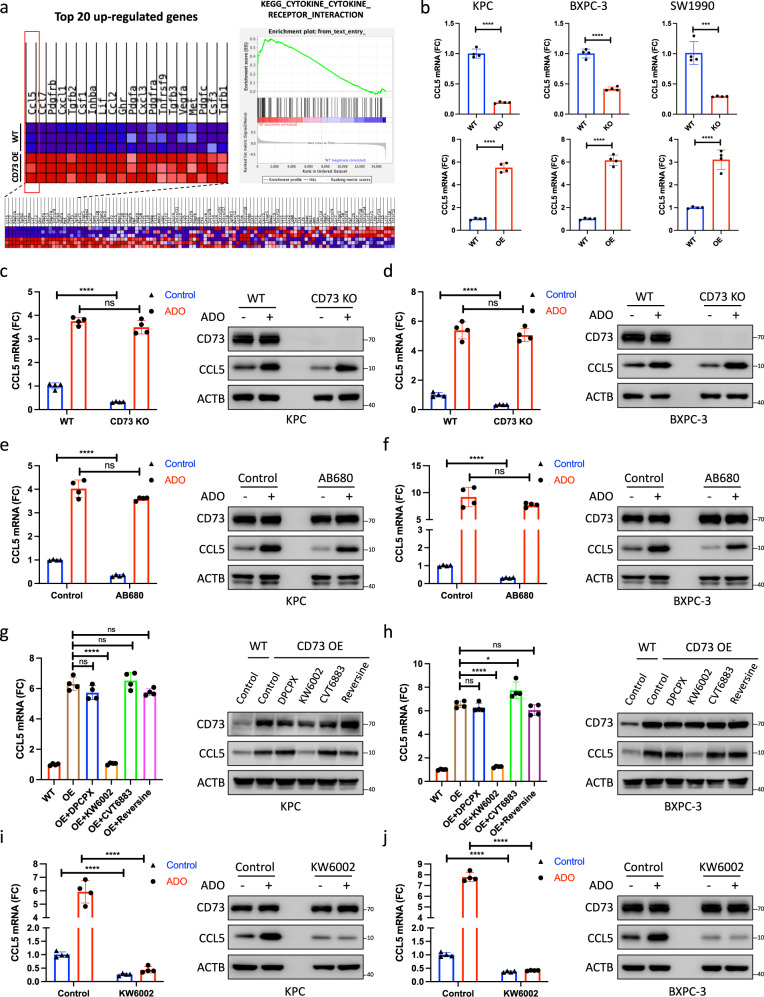
CD73 transcriptionally upregulates CCL5 by tumor cell-autocrine adenosine–Adora2a signaling. **a** Alteration of the cytokine receptor interaction pathway and the top upregulated genes induced by CD73 overexpression (*n* = 4). **b** Maintenance of the CCL5 mRNA level by CD73 in multiple pancreatic cancer cell lines. **c**–**f** Regulation of CCL5 expression by CD73 via AMP-ADO conversion. PCR and immunoblot analyses of CCL5 in WT/CD73 KO KPC cells (**c**) as well as WT/CD73 KO BXPC-3 cells (**d**) treated with or without adenosine. PCR and immunoblot analyses of CCL5 in KPC cells (**e**) and BXPC-3 cells (**f**) treated with or without AB680 and adenosine (*n* = 4). **g**–**j** Regulation of CCL5 expression by ADO via ADO–Adora2a signaling. PCR and immunoblot analyses of CCL5 in WT/CD73 OE KPC cells (**g**) and WT/CD73 OE BXPC-3 cells (**h**) treated with antagonists targeting adenosine receptors (Adora1, DPCPX; Adora2a, KW6002; Adora2b, CVT6883; Adora3, reversine). PCR and immunoblot analyses of CCL5 in KPC cells (**i**) and BXPC-3 cells (**j**) treated with or without KW6002 and adenosine (*n* = 4). All data are representative of three independently performed experiments. Results represent means ± SD of one representative experiment in (**b**–**j**). *P < 0.05, **P < 0.01, ***P < 0.001 using a two-tailed *t*-test; ns: not significant. The exact *P*-values are shown in the Source Data. Source data are provided as a Source Data file.

The expression level of chemokines can be regulated in multiple mechanisms ranging from transcription activation to post-translational degradation. To assess whether CD73 regulates the protein stability and degradation of CCL5, both WT KPC and BXPC-3 cells and the corresponding CD73-depleted cells were treated with chloroquine and MG132. Chloroquine (a lysosome inhibitor that alters the acidic pH of lysosomes and inhibits the degradation of the lysosomal protein) and MG132 (a potent proteasome inhibitor that suppresses the degradation of ubiquitin-conjugated proteins) did not affect CD73 depletion-induced CCL5 downregulation (Supplementary Fig. 10a, b). Interestingly, GSEA based on RNA-seq data of WT and CD73OE KPC cells showed significant upregulation of the chemokine and cytokine receptor interaction pathways, and CCL5 was among the most transcriptionally upregulated genes (Fig. 5a). The alterations in CCL5 transcription induced by CD73 depletion and overexpression were further confirmed in KPC, BXPC-3, and SW1990 cells (Fig. 5b). As a metabolic enzyme, tumor cell-autonomous CD73 converts adenosine monophosphate (AMP) to adenosine (ADO), which further activates distinct adenosine receptors and their downstream signaling cascades^15^. To investigate the mechanism by which CD73 regulates the expression of CCL5, both KPC and BXPC-3 cells with or without CD73 depletion were cocultured with AMP. AMP treatment resulted in increased expression of CCL5 at both the mRNA and protein levels, while upregulation of CCL5 induced by AMP treatment was largely inhibited by CD73 depletion (Supplementary Fig. 11a, b). Moreover, upregulation of CCL5 induced by AMP treatment was largely abolished by CD73 inhibition with AB680 (Supplementary Fig. 11c, d). To investigate whether the regulation of CCL5 is ADO-dependent, KPC and BXPC-3 cells with or without CD73 depletion were cocultured with ADO. ADO treatment largely rescued CD73 depletion- or inhibition-induced downregulation of CCL5 (Fig. 5c–f). Furthermore, KPC and BXPC-3 cells overexpressing CD73 were treated with inhibitors targeting Adora1, Adora2a, Adora2b, and Adora3. Treatment with KW6002, an Adora2a inhibitor, largely abolished the CD73 overexpression-induced upregulation of CCL5 (Fig. 5g, h). In addition, Adora2a inhibition by KW6002 and Adora2a depletion completely abolished the ADO-induced upregulation of CCL5 at both the mRNA and protein levels (Fig. 5i, j, Supplementary Fig. 11e, f). These results suggested that CD73 induces the upregulation of CCL5 in pancreatic cancer in an autocrine manner by converting AMP to ADO.

### Adenosine–Adora2a signaling stimulates CCL5 transcription by activating the p38–STAT1 axis

**Fig. 6:**
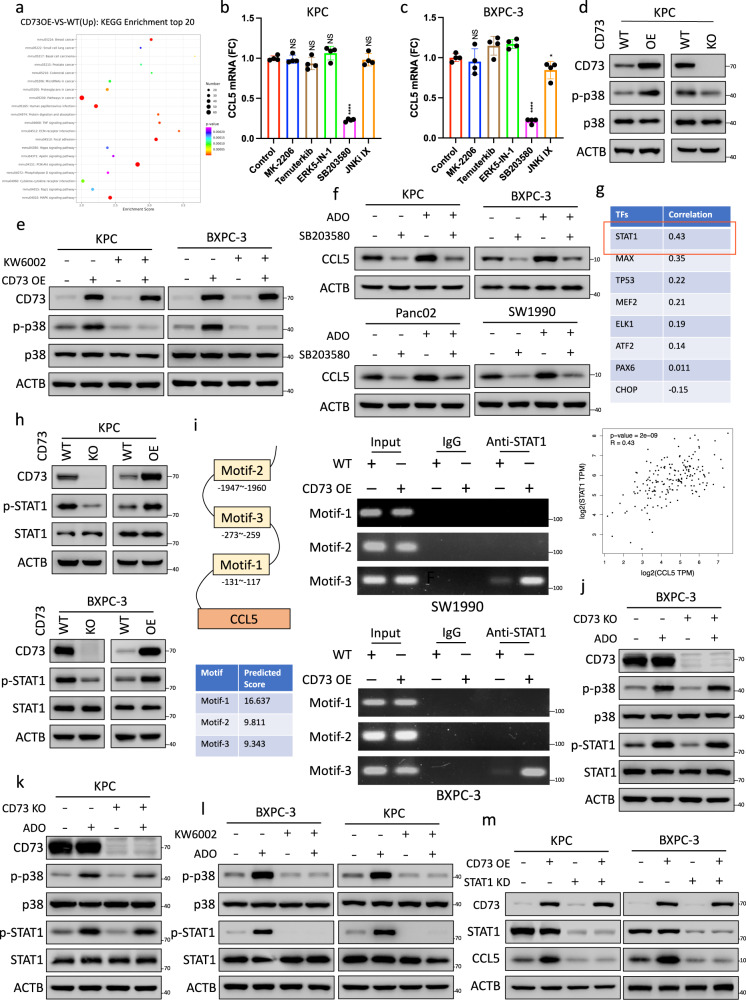
Adenosine–Adora2a signaling stimulates CCL5 transcription by activating the p38–STAT1 axis. **a** Top upregulated pathways identified from transcriptomic data of KPC cells with and without CD73 overexpression. **b**, **c** p38 inhibition reduces the expression of CCL5 at the transcriptional level. mRNA levels of CCL5 in ADO-preincubated KPC cells (**b**) and BXCP-3 cells (**c**) treated with MK-2006, temuterkib, ERK5-IN-1, SB20350, or JNK inhibitor IX (*n* = 4). **d** CD73 regulates the phosphorylation of p38. Immunoblot analysis of CD73, p-p38, and p38 in CD73 KO/OE KPC cells. **e** Regulation of p38 phosphorylation by CD73 via ADO–Adora2a signaling. Immunoblot analysis of CD73, p-p38, and p38 in CD73 KO/OE cells treated with or without KW6002. **f** ADO–Adora2a signaling regulates CCL5 expression through p38 activation. Immunoblot analysis of CCL5 in KPC and BXPC-3 cells treated with or without adenosine and SB203580. **g** Correlation analysis between classical downstream transcription factors and CCL5 based on the TCGA database. **h** CD73 regulates the phosphorylation of STAT1. Immunoblot analysis of CD73, p-STAT1, and STAT1 in CD73 KO/OE KPC cells. **i** Direct binding of STAT1 to the promoter and enhancer of CCL5. ChIP assay of the binding between STAT1 and three predicted regions in the CCL5 gene promoter in both SW1990 and BXCP-3 cells. **j**, **k** Regulation of the p38–STAT1 axis by CD73 via ADO–Adora2a signaling. Immunoblot analysis of CD73, p-p38, p38, p-STAT1, and STAT1 in CD73 WT/KO BXPC-3 (**j**) and KPC (**k**) cells treated with or without adenosine. **l** Immunoblot analysis of p-p38, p38, p-STAT1, and STAT1 in BXPC-3 and KPC cells treated with or without adenosine and KW6002. **m** Abolition of CCL5 upregulation induced by CD73 overexpression through STAT1 depletion. Immunoblot analysis of CD73, STAT1, and CCL5 in WT and CD73 KO KPC cells and BXPC-3 cells with or without STAT1 depletion. All data are representative of three independently performed experiments. Results represent means ± SD of one representative experiment in (**b**, **c**). *P < 0.05, **P < 0.01, ***P < 0.001 using a two-tailed *t*-test; ns: not significant. The Spearman correlations and *P*-values by Spearman’s test are indicated in (**g**). The exact *P*-values are shown in the Source Data. Source data are provided as a Source Data file.

KEGG enrichment analysis was performed to identify the pathways most affected by CD73 overexpression in KPC cells (Fig. 6a). Notably, both the PI3K–AKT and MAPK pathways were among the top 20 pathways upregulated by CD73 overexpression (Supplementary Fig. 12a, b). Previous studies have demonstrated that the PI3K–AKT and MAPK pathways are closely associated with the expression levels of cytokines and chemokines in various malignancies^38,39^. To evaluate whether these pathways are involved in the transcriptional upregulation of CCL5 induced by the activation of ADO–Adora2a signaling, KPC and BXPC-3 cells incubated with adenosine were subsequently screened with the corresponding inhibitors. Interestingly, SB203580, a p38 MAPK inhibitor, significantly downregulated CCL5 at both the mRNA (Fig. 6b, c) and protein levels (Supplementary Fig. 12c). In addition, we confirmed that CD73 overexpression induced the phosphorylation of p38 MAPK and that CD73 depletion inhibited p38 MAPK phosphorylation in KPC and BXPC-3 cells (Fig. 6d, Supplementary Fig. 12d). Moreover, CD73 overexpression-induced phosphorylation of p38 MAPK was largely abolished by Adora2a inhibition with KW6002, indicating that CD73 overexpression induces p38 MAPK phosphorylation in an ADO–Adora2a-dependent manner (Fig. 6e). Furthermore, the upregulation of CCL5 induced by ADO treatment or CD73 overexpression was largely abolished by p38 MAPK inhibition with SB203580 in KPC, BXPC-3, Panc02, and SW1990 cells (Fig. 6f, Supplementary Fig. 12e–h), demonstrating that ADO–Adora2a signaling induces CCL5 upregulation in a p38 MAPK-dependent manner.

The correlations between the expression of CCL5 and the classical downstream transcription factors of the p38 MAPK pathway, including STAT1, MAX, TP53, MEF2, ELK1, ATF2, PAX6, and CHOP, were evaluated based on the TCGA database (Supplementary Fig. 13a–h). Notably, STAT1 was identified as the transcription factor with the highest positive correlation (Fig. 6g). Immunoblotting results confirmed that CD73 overexpression induced and CD73 depletion inhibited STAT1 phosphorylation in KPC and BXPC-3 cells (Fig. 6h). Using JASPAR online database, three binding sites of STAT1 were found based on the human promoter and enhancer sequence of CCL5 (Supplementary Fig. 13i). To further confirm STAT1-mediated CCL5 induction, chromatin immunoprecipitation (ChIP) assays of BXPC-3 and SW1990 cells was performed. Results showed that STAT1 directly binds to motif 3 and that this binding can be enhanced by CD73 overexpression or ADO treatment (Fig. 6i, Supplementary Fig. 13j, k). Furthermore, the inhibition of STAT1 phosphorylation induced by CD73 depletion was rescued by ADO treatment in BXPC-3 and KPC cells (Fig. 6j, k), and the increased phosphorylation of STAT1 induced by ADO treatment was abolished by Adora2a inhibition with KW6002, demonstrating that CD73 overexpression induces increased phosphorylation of STAT1 in an autocrine ADO–Adora2a-dependent manner (Fig. 6l). Moreover, inhibition of p38 MAPK by SB203580 largely abolished STAT1 phosphorylation induced by either CD73 overexpression or ADO treatment, indicating that CD73-ADO–Adora2a signaling induces STAT1 phosphorylation in a p38 MAPK-dependent manner (Supplementary Fig. 13l, m). Finally, the upregulation of CCL5 induced by CD73 overexpression was completely abolished by STAT1 depletion (Fig. 6m, Supplementary Fig. 13n, o). In order to validate the generality of the phenomenon, we replicated the previous experiment on the SW1990 cell line (Supplementary Fig. 14a–o). Taken together, these data indicated that tumor cell-autocrine adenosine upregulates CCL5 secretion through the Adora2a–p38–STAT1 pathway.

### CCL5 represents a promising target for immunotherapy in pancreatic cancer

**Fig. 7:**
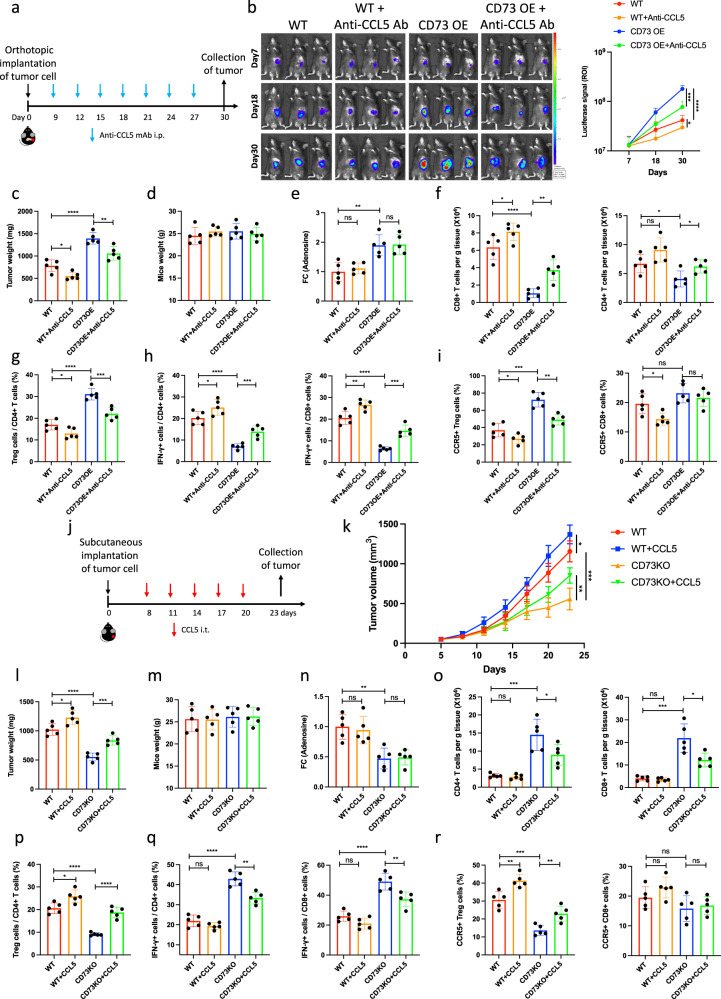
CCL5 represents a promising target for immunotherapy in pancreatic cancer. **a**–**i** Abolition of CD73 overexpression-induced orthotopic tumor progression via CCL5-targeted therapy. WT and CD73 OE KPC cells were orthotopically inoculated into the tail of the pancreas in C57BL/6J mice (*n* = 5) treated with or without CCL5-targeted therapy (**a**). Representative bioluminescence images of mice treated as indicated were acquired on days 7, 18, and 30 (*n* = 5) (**b**). Tumor weights (**c**) and mouse weight (**d**) were individually recorded at the experimental endpoints (*n* = 5). Extracellular adenosine concentration was further quantified (*n* = 5) (**e**). Infiltration of lymphocytes (**f**) and Tregs (**g**), activation of lymphocytes (**h**), as well as the proportion of CCR5^+^ CD8^+^ T cells and CCR5^+^ Tregs were further quantified (*n* = 5) (**i**). **j**–**r** Abolition of CD73 depletion-induced subcutaneous tumor inhibition via intra-tumoral injection of CCL5. WT and CD73 KO KPC cells were subcutaneously implanted in C57BL/6J mice (*n* = 5) treated with or without intra-tumoral injection of CCL5 (**j**). Tumor growth curves were generated at the indicated time points (*n* = 5) (**k**). Tumor weights (**l**) and mouse weight (**m**) were individually recorded at the experimental endpoints (*n* = 5). Extracellular adenosine concentration was further quantified (*n* = 5) (**n**). Infiltration of lymphocytes (**o**) and Tregs (**p**), activation of lymphocytes (**q**), as well as the proportion of CCR5^+^ CD8^+^ T cells and CCR5^+^ Tregs were further quantified (*n* = 5) (**r**). Results represent means ± SD of one representative experiment in (**b–i**), (**k**–**r**). *P < 0.05, **P < 0.01, ***P < 0.001 using a two-tailed *t*-test; ns: not significant. The exact *P*-values are shown in the Source Data. Source data are provided as a Source Data file.

To investigate the therapeutic potential of CCL5 in vivo and determine whether CD73-induced Treg infiltration was ADO accumulation independent, both WT and CD73OE KPC cells were orthotopically implanted into C57BL/6J mice with or without CCL5 blockade (Fig. 7a). Interestingly, CCL5 blockade significantly inhibited CD73 overexpression-induced tumor progression (Fig. 7b, c). There was no difference in the mouse weight between the groups (Fig. 7d). No significant difference for ADO accumulation in tumors was observed between groups treated with or without CCL5 blockade (Fig. 7e). In addition, CCL5 blockade treatment largely abolished CD73 overexpression-induced infiltration of Tregs as well as inhibition of CD8^+^ T-cell and CD4^+^ T-cell infiltration and activation (Fig. 7f–h, Supplementary Fig. 15a–c). Moreover, while no significant difference was observed in the CCR5^+^ CD8^+^ T-cell population, CD73 overexpression induced a significant increase in the CCR5^+^ Treg population, which was largely abolished by CCL5 blockade treatment (Fig. 7i).

In addition, both WT and CD73 KO KPC cells were subcutaneously inoculated into C57BL/6J mice with or without intra-tumoral injection of CCL5 (Fig. 7j). Interestingly, CD73 depletion-induced tumor growth inhibition was largely abolished by injection of CCL5 (Fig. 7k, l). There was no difference in the mouse weight between the groups (Fig. 7m). No significant difference for ADO accumulation in tumors was observed between groups treated with or without intra-tumoral injection of CCL5 (Fig. 7n). In addition, CCL5 injection largely abolished CD73 depletion-induced inhibition of Tregs as well as infiltration and activation of CD8^+^ T cells and CD4^+^ T cells (Fig. 7o–q, Supplementary Fig. 15d, f). Moreover, while no significant difference was observed in the CCR5^+^ CD8^+^ T-cell population, CD73 depletion induced significant inhibition in the CCR5+ Treg population, which was largely abolished by CCL5 injection (Fig. 7r). Furthermore, the prognostic value of the CD73–CCL5 axis was investigated using public databases. Based on the KM plotter database, the survival rates of patients with high expression of CD73 or CCL5 were significantly decreased in the CD8^+^ T-cell-rich groups, indicating that CD73 and CCL5 promote tumor progression by inhibiting CD8^+^ T-cell priming (Supplementary Fig. 16a, b).

## Discussion

The current study demonstrates that CD73 inhibits the antitumor immune response by promoting Treg infiltration in pancreatic cancer. Targeting CD73 significantly reduced Treg infiltration and greatly inhibited tumor growth. Notably, infiltrated Tregs have been shown to play a significant role in maintaining immunological tolerance in various malignancies^40,41^. For instance, CTLA-4^high^ Tregs inhibit the adaptive immune response by downregulating CD80/86 expression in antigen-presenting cells^36,42^. In addition, Tregs with high CD25 expression possess a high affinity for exogenous IL-2, which limits the availability of IL-2 for the activation of antitumor T cells^36,37^. Moreover, Tregs secrete large amounts of suppressive cytokines such as IL-10 and TGF-β, which further contribute to tumor immune suppression^37^. In pancreatic cancer, the immune microenvironment is reshaped toward an immunosuppressive phenotype with a prevalence of Tregs^43^. Accumulating evidence has shown that the frequency of infiltrated Foxp3^+^ Tregs is highly correlated with a poor prognosis in patients with pancreatic cancer^41^. Therefore, exploring the mechanism underlying CD73-mediated Treg recruitment may provide critical information for overcoming immunosuppression in pancreatic cancer. Notably, to eliminate tumor-infiltrated Tregs, Fc-engineered antibodies (Abs) that deplete Tregs through antibody-dependent cellular cytotoxicity (ADCC) have been developed^36,37^. However, the clinical translation of Fc-engineered Abs may face substantial challenges because of the identical surface molecules shared by Tregs and activated effector T cells^36^. In addition, systematic depletion of Tregs may not only enhance antitumor immunity but also induce a strong autoimmune response against normal self-antigens, which results in severe side effects and limits the efficacy of treatment^36^. Therefore, in patients with pancreatic cancer, targeting CD73 may constitute a promising approach for the specific depletion of tumor-infiltrating Tregs, with a low risk of eliciting autoimmunity.

In the current study, we found that tumor cell-autonomous CD73 facilitates Treg recruitment by transcriptionally upregulating CCL5 independent of its canonical effect. Numerous studies have shown that metabolic enzymes in tumor cells possess moonlighting functions, promoting tumor progression via diverse noncanonical mechanisms^44^. For instance, phosphofructokinase 1 (PFK1), a rate-limiting enzyme involved in the glycolytic pathway, was found to regulate YAP/TAZ activation by binding to TEAD transcriptional cofactors, in turn enhancing protumorigenic activities in breast cancer^45^. In addition, 6-phosphofructo-2-kinase/fructose-2,6-bisphosphatase (PFKFB), an enzyme that regulates the interconversion of fructose-2,6-bisphosphate and fructose-6-phosphate, directly phosphorylates the steroid receptor coactivator-3, which further promotes the growth and metastasis of lung cancer in a preclinical model^46^. Recently, accumulating evidence has shown that metabolic enzymes also regulate gene transcription by modulating distinct signaling pathways in various malignancies. For example, pyruvate kinase M2 (PKM2), an enzyme involved in glycolysis, directly regulates gene transcription by binding to and phosphorylating histone H3 at T11^47^. PKM-mediated phosphorylation of histone H3 plays a critical role in the epidermal growth factor (EGF)-induced expression of cyclin D1 and c-Myc, which further promotes tumor cell proliferation in brain cancer. Taken together, these observations indicate that these enzymes with moonlighting functions serve as bridges to integrate two fundamental biological processes in tumors: metabolic reprogramming and gene deregulation. As oncogenic signaling pathways upregulate the expression of certain enzymes to support rapid cell proliferation, these enzymes can reciprocally modulate the associated signaling pathways through noncanonical effects, promoting tumor growth and invasion. Therefore, revealing the noncanonical effects of enzymes such as CD73 in tumor cells may provide valuable information on the biological processes of tumor growth and facilitate the development of specific approaches to disrupt the moonlighting functions of metabolic enzymes for cancer treatment.

In the current study, we found that CD73 activates the p38–STAT1 axis through tumor cell-autocrine adenosine–Adora2a signaling, which results in the upregulation of CCL5 and further Treg recruitment in pancreatic cancer. Tumor biological behavior is significantly influenced by a variety of autocrine signaling factors^48,49^. For instance, autocrine EGF has been reported to significantly promote tumor growth and invasion in colorectal cancer^50^. In breast cancer, autocrine vascular endothelial growth factor induces cell proliferation by modulating the PI3K–AKT pathway^51^. Recently, accumulating evidence has shown that autocrine signaling factors are also involved in modulating the tumor immune microenvironment, which results in the immunological tolerance of tumor cells. For instance, autocrine TGF-β signaling induces EMT in tumor cells and increases the expression of immunosuppressive cytokines and chemokines in various malignancies^48,52^. In addition, autocrine TNF-α upregulates PD-L1 expression in tumor cells, further inhibiting the activation of antitumor lymphocytes^50,53–55^. Therefore, CD73-mediated activation of the autocrine signaling pathway may play a key immunosuppressive role in pancreatic cancer. Various downstream signaling pathways activated by autocrine adenosine signaling in the tumor immune microenvironment should be considered in the development of promising therapeutic approaches to inhibit CD73 signaling, which may maximize the efficacy of CD73-targeted therapies.

Chemokines play a significant role in the recruitment of Treg cells in various malignancies, such as CCR10-CCL28 in ovarian cancer^56^, CCR4-CCL17/22 in breast cancer and melanoma^57,58^, and CCR8-CCL1 in lung cancer^59^. In pancreatic cancer, the secretion of CCL5 by tumor cells critically contributes to the recruitment of Treg cells^60–62^. However, CCL5 can also be utilized by other immune cells, including anti-inflammatory (Treg) and pro-inflammatory (CD8^+^ T) cells, as well as immune-regulatory cells like dendritic cells. Therefore, inhibiting a chemokine can yield complex results depending on the specific situations. PDAC is characterized by increased stroma density with low oxygen tension. According to previous reports, hypoxia and HIF-1α significantly promote the upregulation of CD73 and activate adenosinergic signaling^22,63–65^. Thus, targeting the CD73/CCL5 axis may prove to be a promising strategy for enhancing immunotherapy efficacy in pancreatic cancer marked by hypoxia-induced immunosuppression.

Notably, in addition to CCL5, tumor-autonomous CD73 may upregulate other molecules involved in maintaining an immunosuppressive microenvironment via its noncanonical function. Here, we also found that upregulation of CD73-induced significant alterations in multiple pathways, including the PI3K–AKT and TNF signaling pathways, which have been shown to regulate the expression of various immune checkpoints in tumors. Moreover, autocrine adenosine may act on other receptors, including Adora1, Adora2b, and Adora3, especially in hypoxia situations, which may possibly lead to the activation of distinct immunosuppressive pathways in different malignancies. Considering the profound effects of the moonlighting function of CD73 on the immune system, further studies on its downstream signaling pathways, mediated through multiple adenosine receptors in different malignancies, are needed.

## Methods

### Ethics approval and consent to participate

All procedures involving human specimens in this study were approved by the Ethics Committee of the First Affiliated Hospital of Zhejiang University School of Medicine and were approved and supervised by the same Ethics Committee. All procedures were performed in compliance with the 1964 Declaration of Helsinki and its amendments. All individuals provided written informed consent for participation in the study. All procedures involving animals were performed in accordance with the guidelines of the Animal Ethics Committee of the First Affiliated Hospital of Zhejiang University School of Medicine.

### Abs, inhibitors, and agents

The following Abs were used for immunoblotting: rabbit anti-CD73 (D7F9A Cat#13160, Cell Signaling Technology, 1:2000 for WB, 1:100 for ICH), rabbit anti-CCL5 (E9S2K, Cat#36467, Cell Signaling Technology, 1:2000 for WB, 1:150 for ICH), rabbit anti-p38 MAPK (D13E1, Cat#8690, Cell Signaling Technology, 1:2000 for WB), rabbit anti-phospho-p38 MAPK (D3F9, Cat#4511, Cell Signaling Technology, 1:2000 for WB), rabbit anti-STAT1 (D1K9Y, Cat#14994, Cell Signaling Technology, 1:2000 for WB), rabbit anti-phospho-Stat1 (58D6, Cat#9167, Cell Signaling Technology, 1:2000 for WB), rabbit anti-phospho-Stat1 (D3B7, Cat#8826, Cell Signaling Technology, 1:2000 for WB), rabbit anti-β-Actin (8H10D10, Cat#3700, Cell Signaling Technology, 1:1000 for WB), anti-mouse HRP-linked secondary Ab (Cat#7076, Cell Signaling Technology, 1:5000 for WB), and anti-rabbit HRP-linked secondary Ab (Cat#7074, Cell Signaling Technology, 1:5000 for WB). The following Abs were used for flow cytometry: Brilliant Violet 605 anti-CD45 (I3/2.3, Cat#BD567459, BD Biosciences, 1:1000 for flow cytometry), FITC anti-CD3 (17A2, Cat#BD555274, BD Biosciences, 1:1000 for flow cytometry), APC anti-CD49b (HMα2, Cat#BD558295, BD Biosciences, 1:1000 for flow cytometry), APC-Cy7 anti-CD4 (GK1.5, Cat#BD561830, BD Biosciences, 1:1000 for flow cytometry), PE-Cy7 anti-CD8 (53-6.7, Cat#BD552877, BD Biosciences, 1:1000 for flow cytometry), Brilliant Violet 510 anti-CD44 (IM7, Cat#BD563114, BD Biosciences, 1:1000 for flow cytometry), PE anti-CD62L (MEL-14, Cat#BD553151, BD Biosciences, 1:1000 for flow cytometry), Brilliant Violet 786 anti-CD69 (H1.2F3, Cat#BD564683, BD Biosciences, 1:1000 for flow cytometry), AF700 anti-CD25 (PC61, Cat#102024, BioLegend, 1:1000 for flow cytometry), PE-CF594 rat anti-CD11b (ICRF44, Cat#562399, BD Biosciences, 1:1000 for flow cytometry), PE anti-Foxp3 (3G3, Cat#566881, BD Biosciences, 1:1000 for flow cytometry), PerCP/Cyanine5.5 anti-granzyme B (QA18A28, Cat#396412, BioLegend, 1:1000 for flow cytometry), PE anti-TNF alpha (TN3-19.12, Cat#12-7423-41, eBioscience, 1:1000 for flow cytometry), and APC anti-IFN-γ (4 S.B3, Cat#17-7319-82, eBioscience, 1:1000 for flow cytometry). The following Abs were used for in vivo experiments: Rat anti-CCL5 Antibody (MAB478-500, R&D Systems, 50 μg/mouse), Rat anti-mouse PD-1 antibody (BE0146, Bio X Cell, 120 μg/mouse), Rat anti-mouse CD8α antibody (BE0146, Bio X Cell, 250 µg/mouse), Rat anti-mouse CD4 antibody (BE0119, Bio X Cell, 250 µg/mouse) and Rat anti-mouse NK1.1 antibody (BE0036, Bio X Cell, 250 µg/mouse). The following inhibitors and agents were used: MG132 (S2619, Selleck), chloroquine (S6999, Selleck), KW6002 (S2790, Selleck), DPCPX (HY-100937, MedChemExpress), CVT-6883 (HY-10081, MedChemExpress), reversine (S7588, Selleck), MK-2206 (S1078, Selleck), temuterkib (S8534, Selleck), ERK5-IN-1 (S7334, Selleck), SB203580 (S1076, Selleck), and JNK inhibitor IX (S7508, Selleck).

### Cell lines and cell culture

SW1990, BXPC-3, and PANC02 cell lines were purchased from the American Type Culture Collection (ATCC, Manassas, VA, USA). The KPC cell line obtained from the LSL-Kras G12D/+; LSL-Trp53 R172H/+; Pdx1-Cre mouse model, which was kindly provided by Prof. Raghu Kalluri (Department of Cancer Biology, Division of Basic Sciences, MD Anderson Cancer Center, Houston, TX, USA). SW1990, BXPC-3, and PANC02 were cultured in 1640 medium (SH30027.0, Life Sciences) supplemented with 10% FBS and 1% Pen/Strep (SH30022.01, Life Sciences). KPC was cultured in DMEM medium (SH30243.01, Life Sciences) supplemented with 10% FBS and 1% Pen/Strep (SH30022.01, Life Sciences). All cell lines were incubated at 37 °C under 5% CO_2_. Mycoplasma contamination was routinely evaluated by PCR.

### ELISA

Culture supernatants were individually collected. ELISA kits were used to quantify CCL5 secreted from KPC and Panc02 cells (ab100739, Abcam), as well as SW1990 and BXPC-3 cells (ab174446, Abcam).

### Bioinformatic analysis

GEPIA2 (http://gepia2.cancer-pku.cn, version 2) is an open-access online tool for the analysis of RNA expression data from 9736 tumors and 8587 normal samples from TCGA and GTEx projects using a standard processing pipeline. In this study, GEPIA2 was used to evaluate the correlations between the expression of CCL5 and various potential transcription factors. The significance of the correlations between the investigated genes was assessed using Pearson correlation analysis, and a value of *P* < 0.05 was used as the threshold. The Kaplan–Meier plotter (http://kmplot.com) is an open-access online tool for exploring the correlation between gene expression and survival in more than 30,000 samples based on databases including GEO, EGA, and TCGA. In this study, the Kaplan–Meier plotter was used to evaluate the prognostic value of the CD73–CCL5 axis in pancreatic cancer patients with distinct levels of CD8^+^ T-cell infiltration. Overall survival analyses were performed using the Kaplan–Meier method, and the Mantel–Cox test was used to assess statistical significance. A 50% (Median) cutoff was applied for both the low- and high-expression groups. A *P*-value <0.05 was used as a threshold.

### RNA-seq

RNA-seq was performed by OEbiotech Inc. (Shanghai, China) following a previously published protocol^66^. Briefly, RNA was extracted using TRIzol reagent, and RNA purity and integrity were assessed. Sequencing libraries were generated according to the manufacturer’s instructions. Raw data were analyzed using Trimmomatic, and clean reads were obtained after removing low-quality reads. The clean reads were then mapped to distinct genes using HISAT2. Fragments per kilobase of transcript per million mapped reads (FPKM) of each gene were obtained after calculation based on the read count and length of the gene. GSEA was performed, and gene sets containing at least 10 genes with a *P*-value of <0.05 in each pathway were reported.

### Animal care and use

C57BL/6J (male, 6 weeks old) and BALB/c (male, 6 weeks old) mice were purchased from the Nanjing Biomedical Research Institute of Nanjing University. CD73 (*NT5E*) knockout mice were generated by Cyagen Biosciences Inc. The mice under study were reared in a specific-pathogen-free (SPF) environment at the Experimental Animal Center, First Affiliated Hospital, School of Medicine, Zhejiang University. The ambient temperature was maintained at 21–22 °C, with humidity levels ranging between 48 and 52%. The mice were subjected to a conventional 12:12 light/dark cycle, with lights being turned on at 6:00 a.m. and turned off at 6:00 p.m. Animal experiments were approved by the Institutional Animal Care and Use Committee of the First Affiliated Hospital, College of Medicine, Zhejiang University. For CD73 (NT5E) knockout mice, both male and female mice aged 6–8 weeks were used at the time of experiments unless indicated otherwise. For C57BL/6J and BALB/c mice, male mice aged 8 weeks were used. Empirical determination was used to determine sample sizes for mouse experiments. Mice were assigned randomly to either the control or experimental groups in a gender- and age-matched manner. For orthotopic tumor implantation, 8-week-old male BALB/c nude mice or C57BL/6 mice were orthotopically injected into the tail of the pancreas with 5 × 10^5^ KPC/KPC^luciferase^ cells. All mice were marked before being randomly allocated to the groups. One week after the tumor cells were injected, the treatment was initiated. For subcutaneous tumor implantation, 8-week-old male BALB/c nude mice or C57BL/6 mice were subcutaneously injected with 5 × 10^5^ KPC/KPC *NT5E* KO cells into the right flank. Five days after the tumor cells were injected, the treatment was initiated. An anti-mouse PD-1 Ab (Bio X Cell, West Lebanon, NH, USA) was intraperitoneally injected at a dose of 120 μg/mouse once every 3 days. An anti-mouse CCL5 Ab (R&D Systems, Minneapolis, MN, USA) was intraperitoneally injected at a dose of 50 μg/mouse once every 3 days. AB680 (MedChemExpress, USA) was intraperitoneally injected at a dose of 150 μg/mouse once every 3 days. Anti-CD8, anti-CD4, anti-NK1.1, and anti-CD25 Abs (Bio X Cell, West Lebanon, NH, USA) were administered at a dose of 250 µg/mouse once every 5 days beginning one day before treatment or rechallenge. Intra-tumoral injection of exogenous CCL5 (R&D Systems, Minneapolis, MN, USA) was administered at a dose of 1 μg/mouse in 25 μl PBS. Orthotopic tumor growth was evaluated using bioluminescence imaging. Subcutaneous tumor growth was evaluated by measuring the tumors with a caliper and calculating the tumor volume as length × width^2^ × 0.5. The maximal tumor size/burden permitted by our ethics committee review board was 2000 mm^3^. We confirm that none of the mice included in this study exceeded this limit. The mice were weighed before sacrifice. Tumors were harvested, weighed, and divided into segments for subsequent analysis.

### Overexpression and depletion of genes in cell lines

To generate cells stably expressing CD73, KPC, Panc02, BXPC-3, and SW1990 cells were infected with Flag-CD73 lentiviral particles (ObiO Technology, Shanghai). After 48 h of infection, the cells were selected in a medium containing 40 μg/ml puromycin for 14 days. To generate CD73 KD/KO KPC, Panc02, BXPC-3, and SW1990 cells, the cells were transfected with human CD73 double nickase plasmid (sc-423919-NIC, Santa Cruz) and mouse CD73 double nickase plasmid (sc-400307-NIC, Santa Cruz) and then sorted by GFP fluorescence. Similarly, to generate STAT1 KD cells, KPC and BXPC-3 cells were transfected with human STAT1 double nickase plasmid (sc-400086-NIC, Santa Cruz) and mouse STAT1 double nickase plasmid (sc-423174-NIC, Santa Cruz) and then sorted by GFP fluorescence. After the cells were subcloned, PCR and western blot analyses were used to determine the efficiency of overexpression or depletion.

### Western blot analysis

RIPA lysis buffer (P0013B, Beyotime Biotechnology) with 1 mM phenylmethanesulfonyl fluoride (ST505, Beyotime Biotechnology) was used to lyse cells following centrifugation at 12,000 × *g* for 15 min. The protein concentration in the supernatant was measured after boiling for 5 min in 1× NuPAGE LDS sample buffer, and proteins were separated by 8–12% SDS‒PAGE. The proteins were then transferred onto a PVDF membrane (IPFL00010, Millipore). The membrane was blocked with 5% skimmed milk in TBST for 1 h and incubated with the indicated Abs overnight. After three washes with TBST, the membranes were incubated with HRP-conjugated Abs and visualized using a ChemiScope Touch imaging system (Clinx Science Instruments).

### (Multiplex) IHC staining

Human pancreatic adenocarcinoma cancer tissue specimens were obtained from the First Affiliated Hospital of Zhejiang University School of Medicine, including 32 patients who underwent surgical resection for pancreatic cancer. The cohort consisted of 19 male and 13 female patients with a mean age of 60 years (range: 42–76 years). Informed consent was obtained from participants. Formalin-fixed, paraffin-embedded sections of human and mouse tumor tissues were sliced and placed on SuperFrost Plus glass slides (4-μm thick) for the evaluation of FOXP3, CD73, and CCL5 expression. After deparaffinization and rehydration, the slides were incubated overnight with primary Abs against the target proteins. After three washes with PBS, the slides were incubated with HRP-conjugated Abs, and the targeted proteins were visualized using a diaminobenzidine (DAB) chromogen kit (BDB2004, Biocare) after staining with diluted hematoxylin for 3 min. The ImageScope software (Leica Biosystems, Wetzlar, Germany) was used to acquire representative images. Multiplex IHC staining for CD73 and CCL5 was performed on the human pancreatic cancer samples. Tissue sections (4-μm thick) were stained using a multiplex immunohistochemistry/immunofluorescence staining kit (abs50012, Absin, Shanghai, China) following the manufacturer’s instructions. Antigen retrieval was performed using sodium citrate buffer. Tissue sections were blocked with 3% BSA buffer for 30 min. Next, the sections were washed three times with PBST and incubated with a primary Ab for 1 h at room temperature. The sections were then incubated with HRP-conjugated anti-rabbit IgG at room temperature for 30 min, and staining was developed using a fluorescent dye diluted in the signal amplification reagent. The primary Abs were removed by repeating the antigen retrieval step, and the sections were then incubated with another primary Ab. After staining, the sections were counterstained with DAPI and mounted in a glycerol and gelatin mounting medium. Images of the stained sections were acquired using a confocal laser scanning microscope.

### Flow cytometry

The tumors were resected at the end of the in vivo experimental period. Single-cell suspensions were obtained from fresh tumor tissues by incubation with a mixture of RPMI-1640 medium containing 2% FBS, 3 mM CaCl_2_, 200 U/ml collagenase (17104019, Thermo Fisher Scientific), and 10 μg/ml DNase (D5025, Sigma‒Aldrich) with shaking at 200 rpm and 37 °C for 1 h. Invitrogen LIVE/DEAD Fixable Dead Cell Stain was used to distinguish dead cells, and live cells were further incubated with an anti-CD16/CD32 Ab for 30 min to block Fc-mediated reactions. To analyze immune cell infiltration, CD45, CD3, CD4, CD8, CD25, PD-1, CD44, CD62L, CD11b, CD11c, Ly6G, Ly6C, F4/80, MHC II, CD206, CD86, and CD68 proteins on the cell surface were stained. To quantify effector T-cell cytokine expression, some cells were incubated in a culture medium containing 5 ng/ml PMA, 500 ng/ml ionomycin, brefeldin A, and GolgiStop at 37 °C for 4 h. Granzyme B, TNF-α, and IFN-γ were stained using a fixation/permeabilization solution kit (555028, BD Biosciences). All samples were analyzed using a Fortessa cell analyzer (BD Biosciences), and the data were analyzed using FlowJo software (v10.4).

### CyTOF

CyTOF analysis was performed by PLTTech Inc. (Hangzhou, China) following a previously published protocol^67^. Briefly, single-cell suspensions were obtained by dissociating tumor tissues using DNase, collagenase IV, and hyaluronidase. Samples were incubated with Abs according to an in-house developed panel and finally loaded onto a CyTOF system (Helios, Fluidigm, San Francisco, CA, USA) for signal detection and analysis. The tSNE was applied to identify immune cell types after nonlinear dimensionality reduction.

### Treg migration assays

For the mouse Treg migration assay, Tregs were first isolated from splenocytes (130-091-041, Miltenyi Biotec). For the human Treg migration assay, Tregs were first isolated from the peripheral blood of patients (130-091-301, Miltenyi Biotec). The supernatant from tumor cells under different treatment conditions was plated in the lower chambers, and Tregs were plated in Transwell inserts (5.0-μm pore size). FBS-free DMEM (GIBCO, Invitrogen) was used as a culture medium. After 48 h, cells in the lower chambers were collected and enumerated by flow cytometry with counting beads.

### ChIP

ChIP was performed using a ChIP assay kit (P2078, Beyotime). Briefly, SW1990 WT/CD73OE and BXPC-3 WT/CD73 OE cells at 80% confluence were treated with 1% formaldehyde for 15 min at 37 °C, followed by neutralization with glycine for 5 min. After washing with PBS, the cells were collected in sodium dodecyl sulfate (SDS) lysis buffer and sonicated for 10 min. After centrifugation, the supernatants were collected and incubated with an anti-STAT1 Ab or IgG overnight at 4 °C, followed by incubation with protein G magnetic beads for 6 h at 4 °C. The immunoprecipitated protein‒DNA complexes were eluted with 10 μg/ml proteinase K at 45 °C for 1 h. The immunoprecipitated DNA was further purified, collected using a DNA extraction kit, and analyzed by PCR. The primer sequences used for ChIP-PCR were as follows.

Motif 1: Forward primer: CCCTTTATAGGGCCAGTTGAGG

Reverse primer: ACTCGAATTTCCGGAGGCTA

Motif 2: Forward primer: ACCCCCTACTGAGATCATTTTC

Reverse primer: ACTCTGCCTCAAAGTCATCGT

Motif 3: Forward primer: TCTCCCTCACTGCTCTCTCA

Reverse primer: CCTGTCCTAACTGCCACTCC

### Statistical analysis

The Student’s *t*-test was used for comparisons between the two groups. One-way analysis of variance (ANOVA) was used to compare multiple groups. The log-rank test or the Gehan–Breslow–Wilcoxon test was used for survival analysis. Pearson’s correlation analysis was used to assess correlations between different genes. Statistical analysis was performed using SPSS 18.0 and GraphPad Prism 7.0. The results are presented as mean ± standard deviation (SD) of at least three independent experiments, and statistical significance was set at *P* < 0.05.

### Reporting summary

Further information on research design is available in the Nature Portfolio Reporting Summary linked to this article.

## Supplementary information

Supplementary Information

Peer Review File

Reporting Summary

## Data Availability

Figure 4e, Supplementary Figs. 13a–h, 13i and 16a, b were generated from publicly available databases, including GEPIA2 (http://gepia2.cancer-pku.cn), JASPAR (https://jaspar.genereg.net/) and Kaplan–Meier Plotter (http://kmplot.com/analysis). The RNA-Seq data have been deposited in the Bioproject database under the accession code PRJNA934940. The remaining data can be found in the Article, Supplementary Information or Source Data file, which are provided alongside this paper. Source data are provided with this paper.

## Source data

Source data
