# Supplementary material for: Transcriptional control of pancreatic cancer immunosuppression by metabolic enzyme CD73 in a tumor-autonomous and -autocrine manner
Source: Nat Commun. 2023 Jun 8;14:3364. doi: 10.1038/s41467-023-38578-3 (PMC10250326; doi:10.1038/s41467-023-38578-3)
Supplement: Supplementary file 1 — Supplementary Information [file 41467_2023_38578_MOESM1_ESM.docx]

**Supplementary Figures**

**
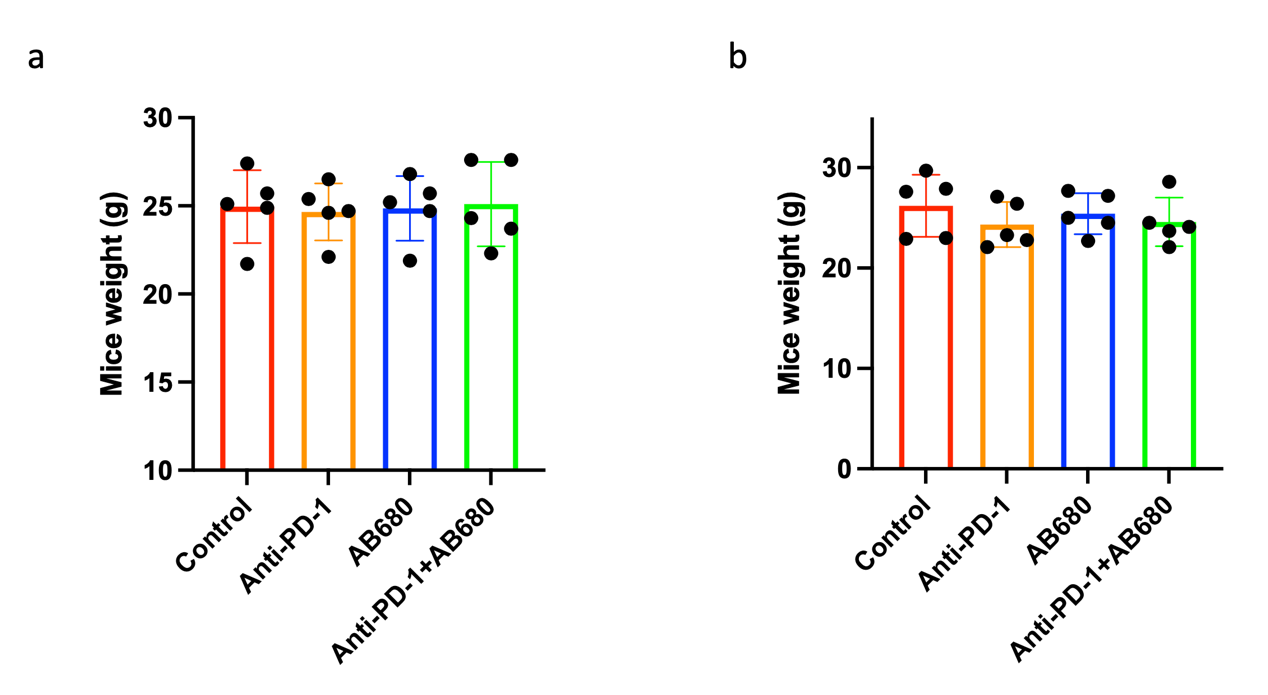
**

**Supplementary Fig. 1: The combination of AB680 and anti-PD-1 Abs was well tolerated.** (a-b) Weight of mouse implanted with orthotopic tumor (a) or subcutaneous tumor (b) were individually recorded at the experimental endpoints (n=5). Results represent means ± SD of one representative experiment in **a-b**. *P < 0.05, **P < 0.01, ***P < 0.001 using a two-tailed t-test; ns: not significant. The exact p values are shown in the Source Data. Source data are provided as a Source Data file.

**
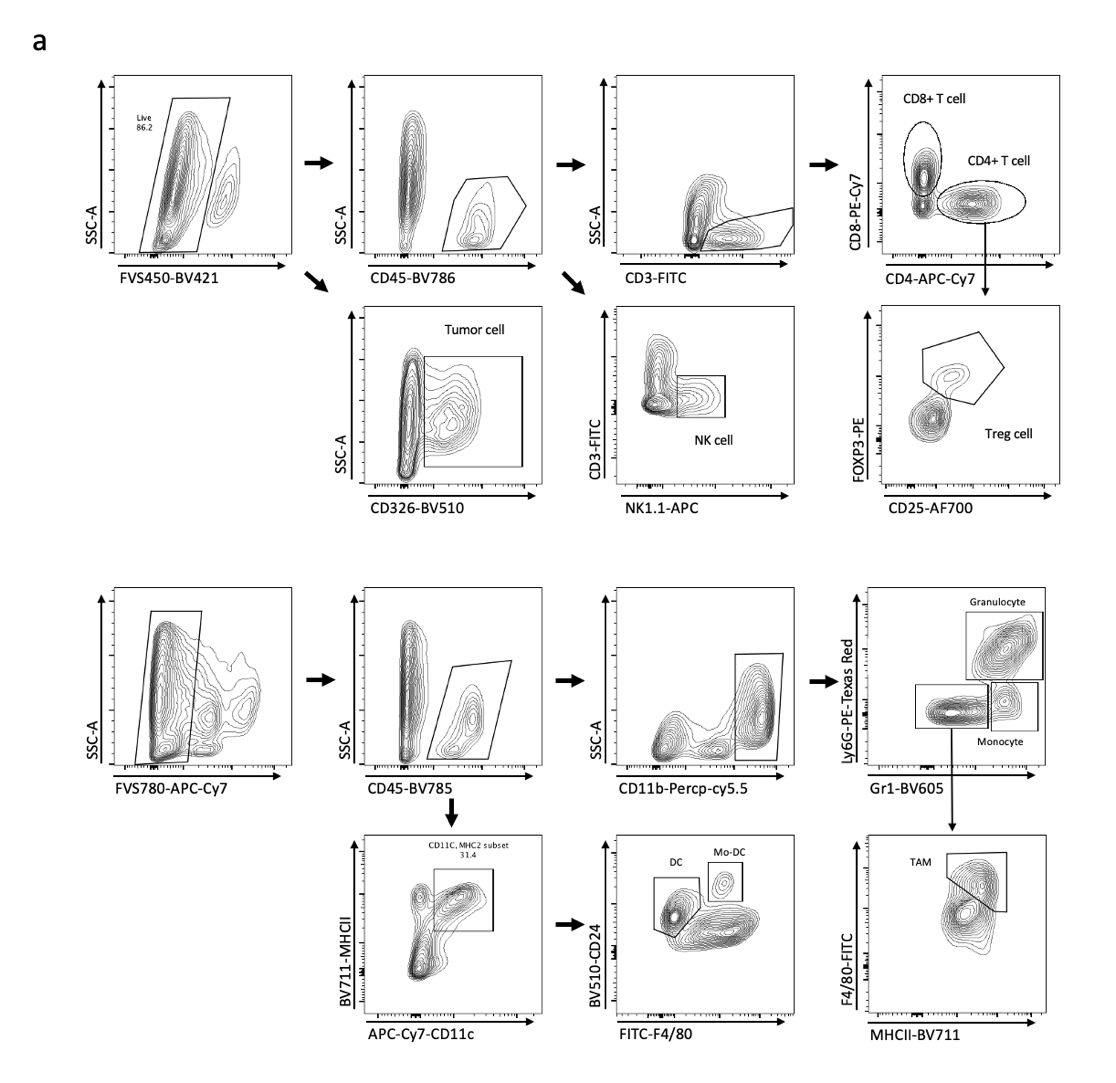
**

**Supplementary Fig. 2: Gating strategy for flow cytometry in mouse pancreas tumor.** (a) Gating strategy of immune cell population in KPC tumors.

**
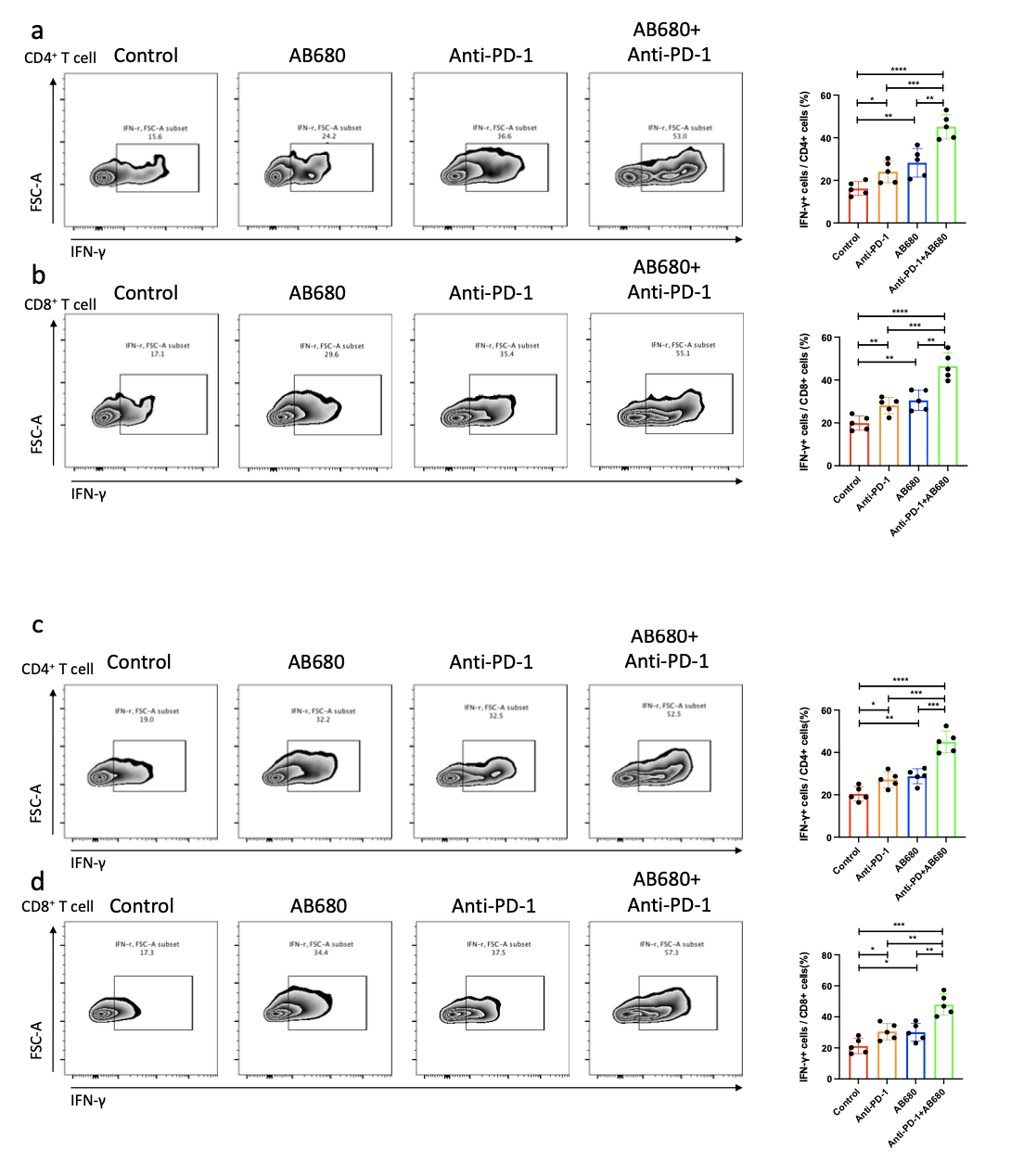
**

**Supplementary Fig. 3: CD73 inhibition enhanced the activation of lymphocytes in combination with PD-1 targeted therapy.** (a-b) The combination of AB680 and anti-PD-1 Abs enhanced the activation of lymphocytes in orthotopic tumors (n=5). Representative images of activated CD4^+^ T cells (a) and activated CD8^+^ T cells (b). (c-d) The combination of AB680 and an anti-PD-1 Ab enhanced the activation of lymphocytes in subcutaneous tumors (n=5). Representative images of activated CD4^+^ T cells (c) and activated CD8^+^ T cells (d). Results represent means ± SD of one representative experiment in **a-d**. *P < 0.05, **P < 0.01, ***P < 0.001 using a two-tailed t-test; ns: not significant. The exact p values are shown in the Source Data. Source data are provided as a Source Data file.


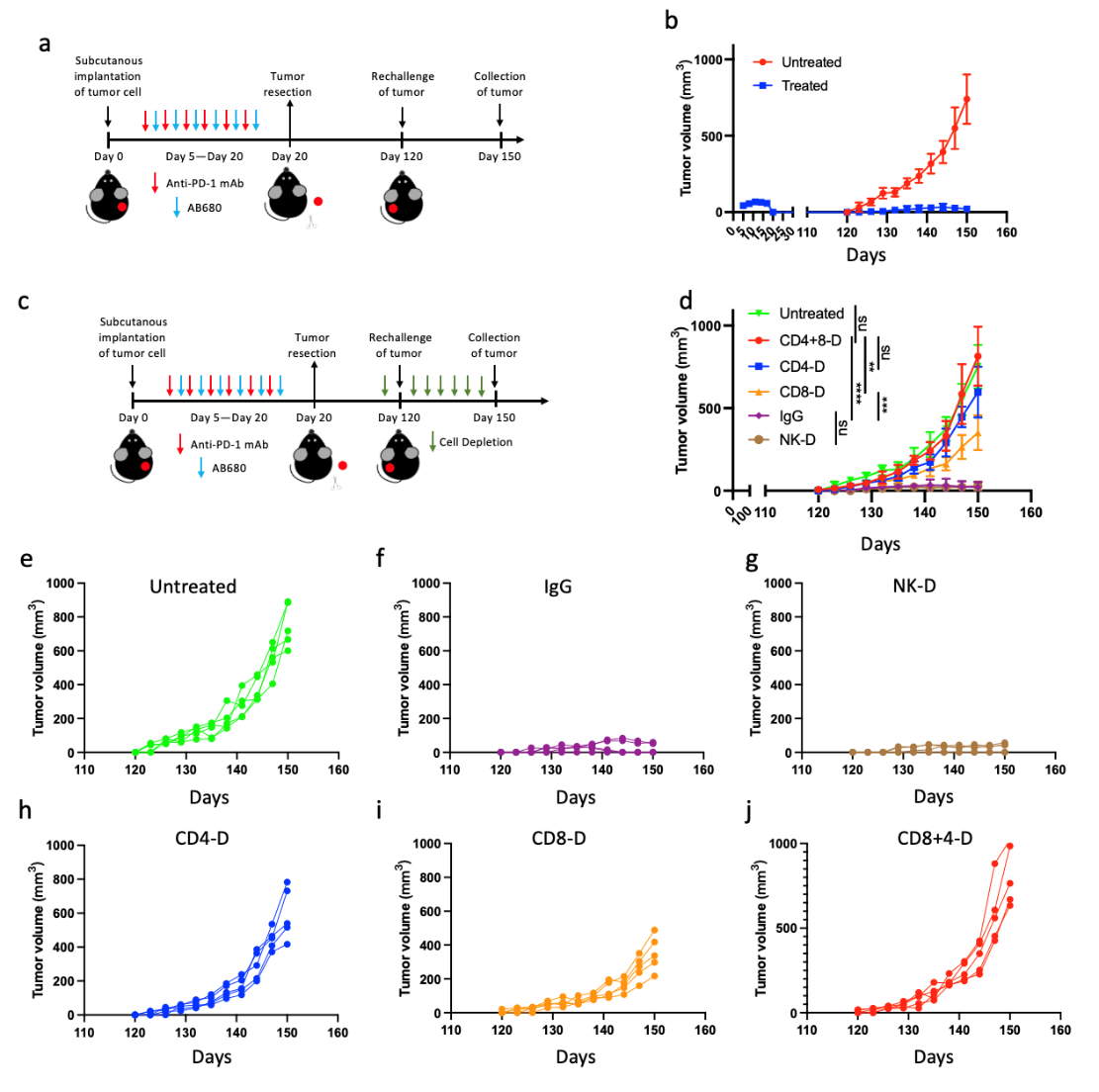


**Supplementary Fig. 4: The combination of CD73 inhibition and PD-1 targeted therapy induces long-term T-cell-dependent antitumor immunologic memory.** (a) Initial KPC cells were implanted subcutaneously prior to combination therapy and resection on day 20 (n=5). The mice were rechallenged with a second implantation of KPC cells. (b) Tumor growth curves were individually recorded at the indicated time points (n=5). (c-j) The long-term immune response induced by the combination of CD73 inhibition and PD-1 targeted therapy was T-cell dependent. Initial KPC cells were implanted subcutaneously prior to combination therapy and resection on day 20. The mice (n=5) were subjected to immune cell depletion before and after being rechallenged with a second implantation of KPC cells (c). T-cell depletion abolished the long-term immunologic memory induced by the combination therapy (d). Growth curves of tumors from untreated mice (e) as well as mice treated with IgG (f), an anti-NK1.1 Ab (g), an anti-CD4 Ab (h), an anti-CD8 Ab (i), and anti-CD4+anti-CD8 Abs (j). Results represent means ± SD of one representative experiment in **b, d**. *P < 0.05, **P < 0.01, ***P < 0.001 using a two-tailed t-test; ns: not significant. The exact p values are shown in the Source Data. Source data are provided as a Source Data file.


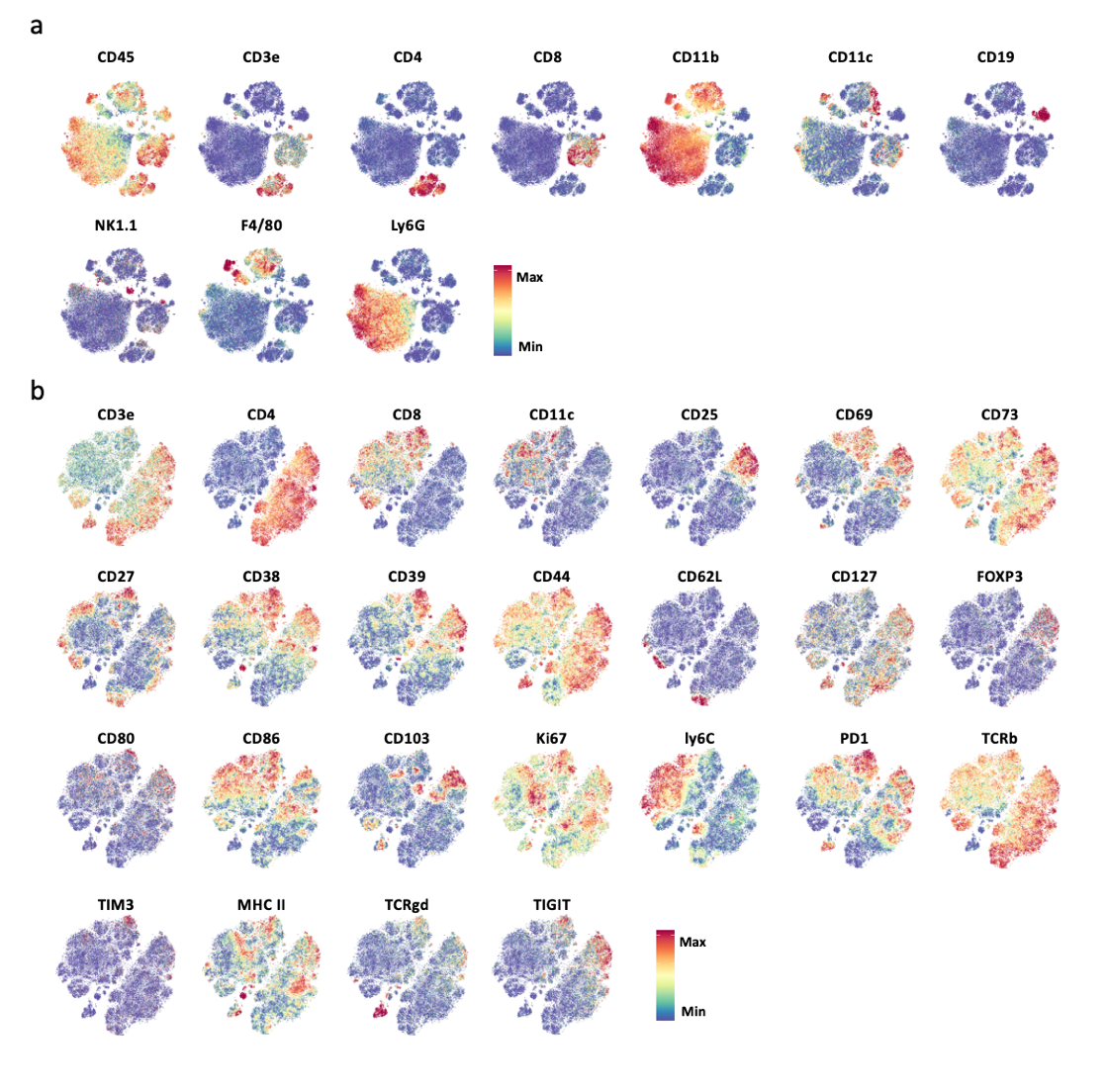


**Supplementary Fig. 5: Expression levels of immune markers on tSNE plots.** (a) tSNE plots of CD45^+^ immune cells with expression levels of markers for distinct cell subsets. (b) tSNE plots of CD3^+^ T cells with varying markers for distinct subsets of T cells.

**
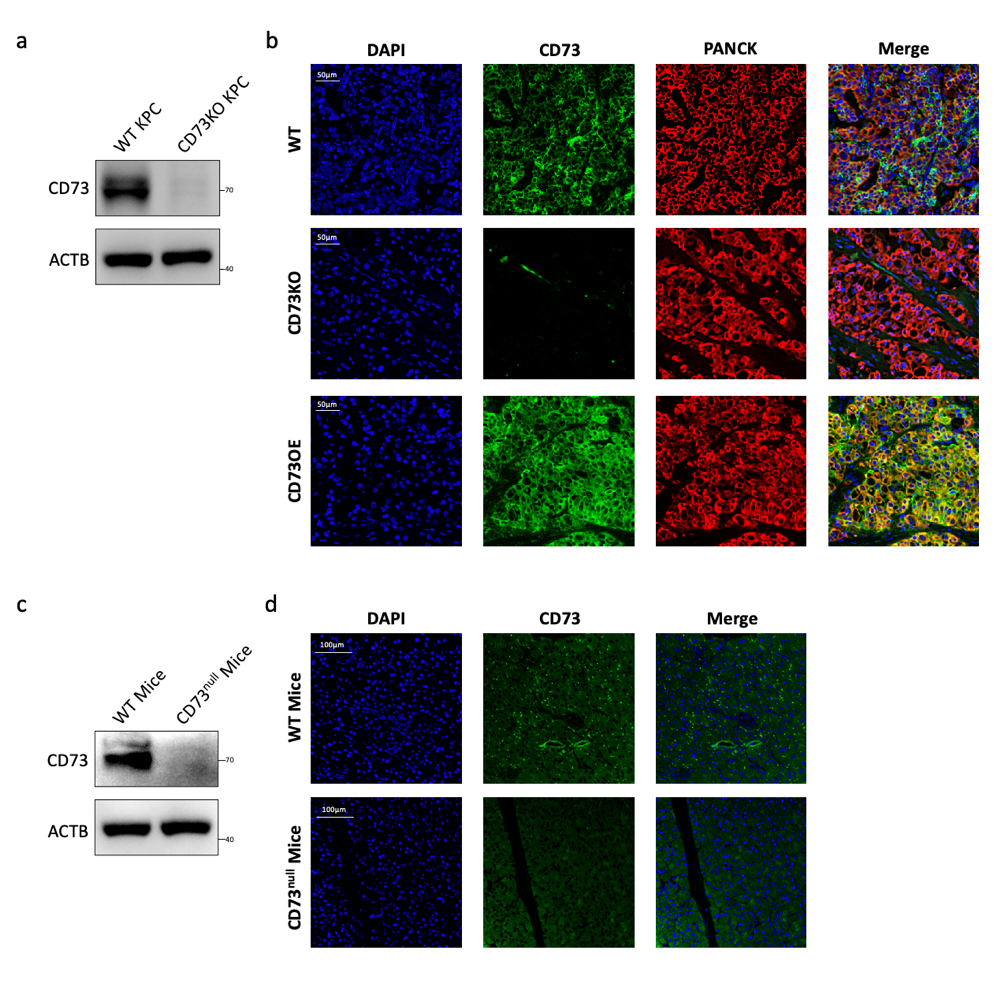
**

**Supplementary Fig. 6: Expression levels of CD73 in tumors and pancreatic tissue of mice.** (a) Immunoblot analysis of WT and CD73KO KPC cells. (b) mICH of orthotopically implanted WT, CD73KO and CD73OE KPC tumors (Scale bars: 50 μm). (c) Immunoblot analysis of pancreas tissue of WT mice and CD73^null^ C57BL/6J mice. (d) mICH of pancreas tissue of WT mice and CD73^null^ C57BL/6J mice. All data are representative of three independently performed experiments (Scale bars: 100 μm).


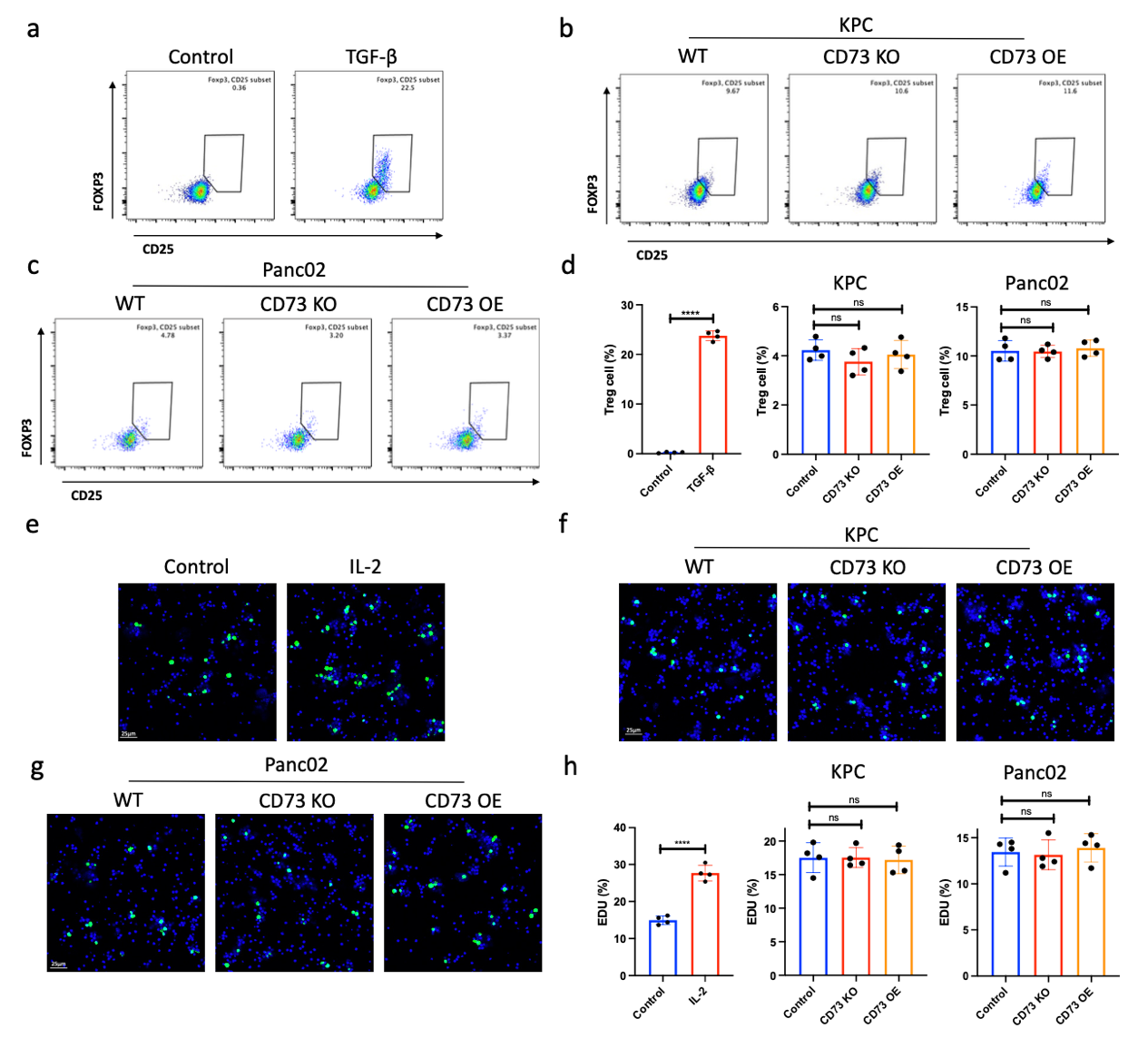


**Supplementary Fig. 7: Tumor cell-autonomous CD73 does not affect Treg proliferation and conversion.** (a-d) Tumor cell-autonomous CD73 does not affect Treg conversion. Isolated CD4^+^ CD25^-^ cells were incubated with WT and CD73 KO/OE pancreatic cells (n=4). Representative images of CD4^+^CD25^-^ cells treated with TGF-β (a). Representative images of CD4^+^CD25- cells cocultured with KPC cells (b) and Panc02 cells (c). The results were further quantified (n=4) (d). (e-h) Tumor cell-autonomous CD73 does not affect Treg proliferation. Isolated Tregs were incubated with WT and CD73 KO/OE pancreatic cells (n=4). Blue dots: DAPI, Green dots: EdU. Representative images of Tregs treated with IL-2 (Scale bars: 25 μm) (e). Representative images of Tregs cocultured with KPC cells (f) and Panc02 cells (g) (Scale bars: 25 μm). The results were further quantified (n=4) (h). Results represent means ± SD of one representative experiment in **d, h**. *P < 0.05, **P < 0.01, ***P < 0.001 using a two-tailed t-test; ns: not significant. The exact p values are shown in the Source Data. Source data are provided as a Source Data file.

**
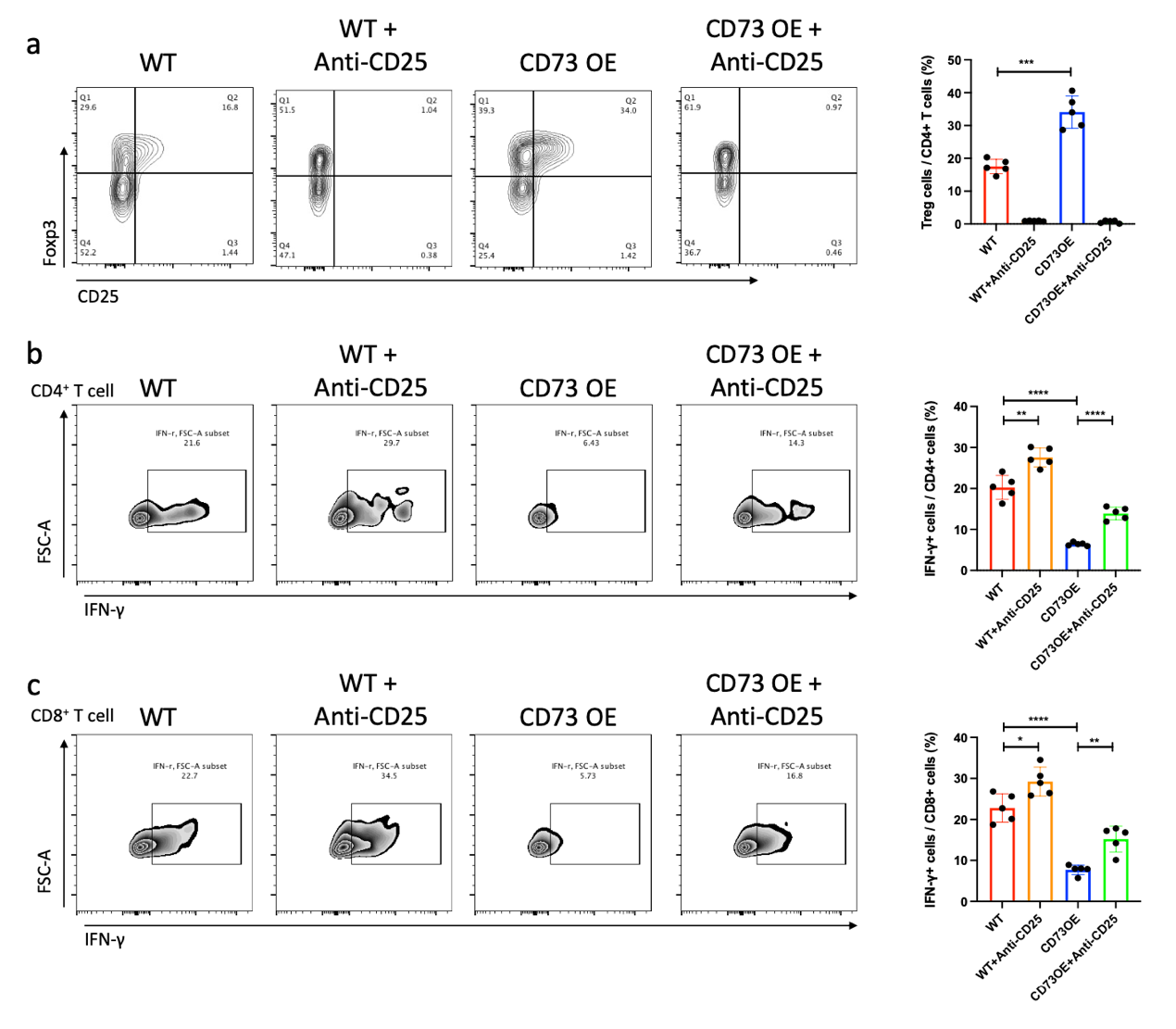
**

**Supplementary Fig. 8: Treg depletion partially rescues the immune inhibition caused by CD73 overexpression.** (a) Representative images of infiltrated Tregs. (b-c) Representative images of activated CD4^+^ T cells (b) and CD8^+^ T cells (c). Results represent means ± SD of one representative experiment in **a-c**. *P < 0.05, **P < 0.01, ***P < 0.001 using a two-tailed t-test; ns: not significant. The exact p values are shown in the Source Data. Source data are provided as a Source Data file.


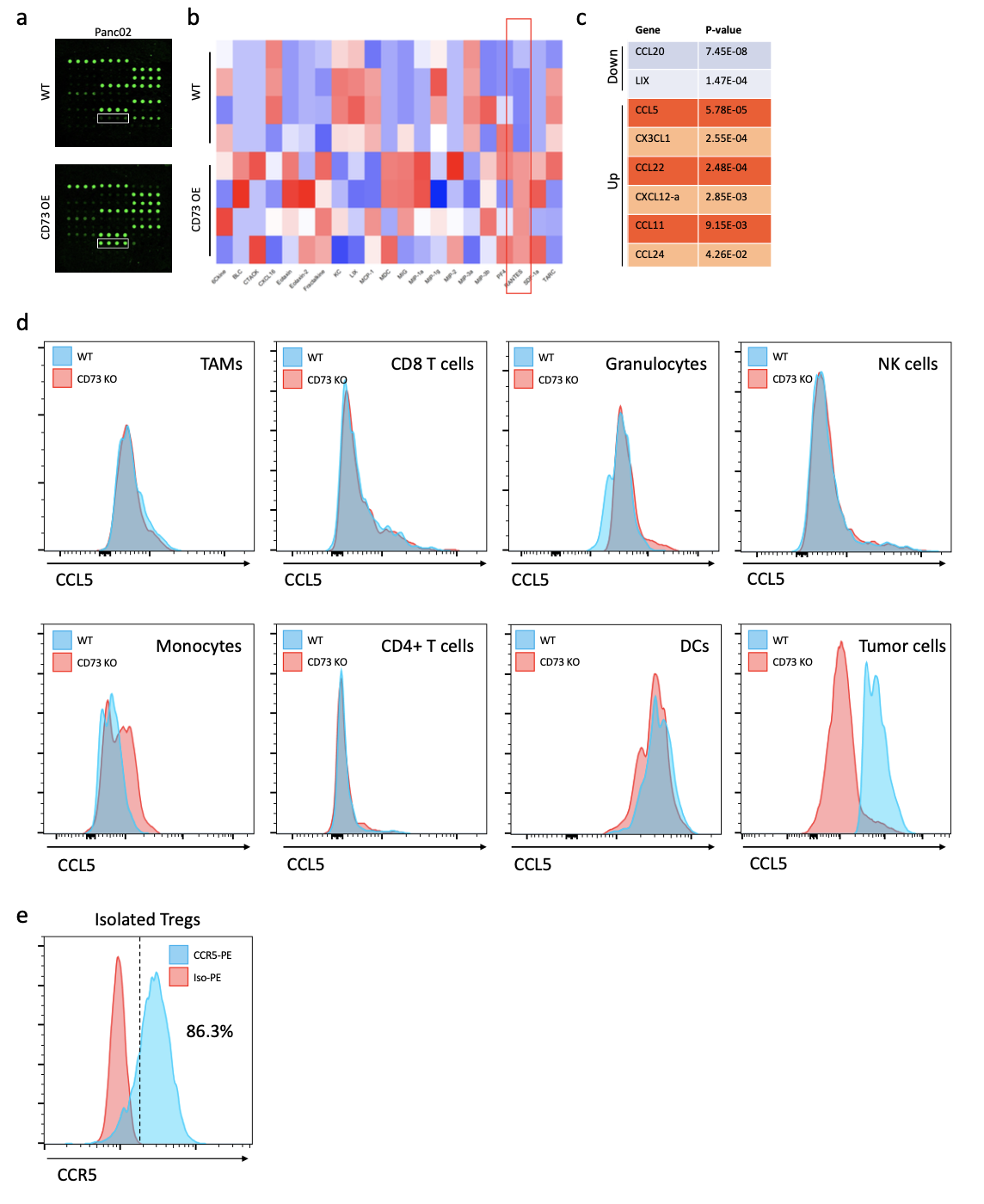


**Supplementary Fig. 9: CD73 induced Treg infiltration via CCL5-CCR5 axis.** (a-c) CD73 regulates chemokine expression. Differential expression of 25 chemokines was detected using chemokine arrays based on culture medium supernatant from WT and CD73 OE Panc02 cells (n=4). Representative image of chemokine detection in WT and CD73 OE PANC02 cells (a). Heatmap (b) and P values (c) of the detected chemokines. (d) Representative images of CCL5 expression of tumor cells and immune infiltrated cells in WT and CD73KO KPC tumors collected from immunocompetent mice. (e) Representative images of CCR5 expression of Tregs isolated from splenocytes of immunocompetent mice.


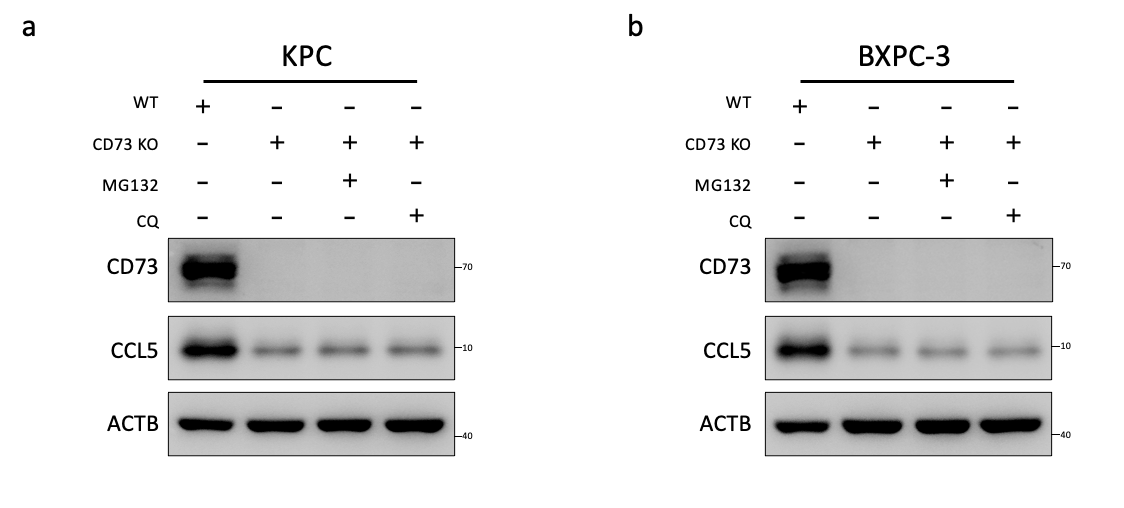


**Supplementary Fig. 10: CD73 regulates CCL5 expression independent of proteasomal and lysosomal degradation.** (a-b) Immunoblot analysis of CD73 and CCL5 in WT and CD73 KO KPC cells (a) as well as BXPC-3 cells (b) treated with MG132 and chloroquine. All data are representative of three independently performed experiments.

**
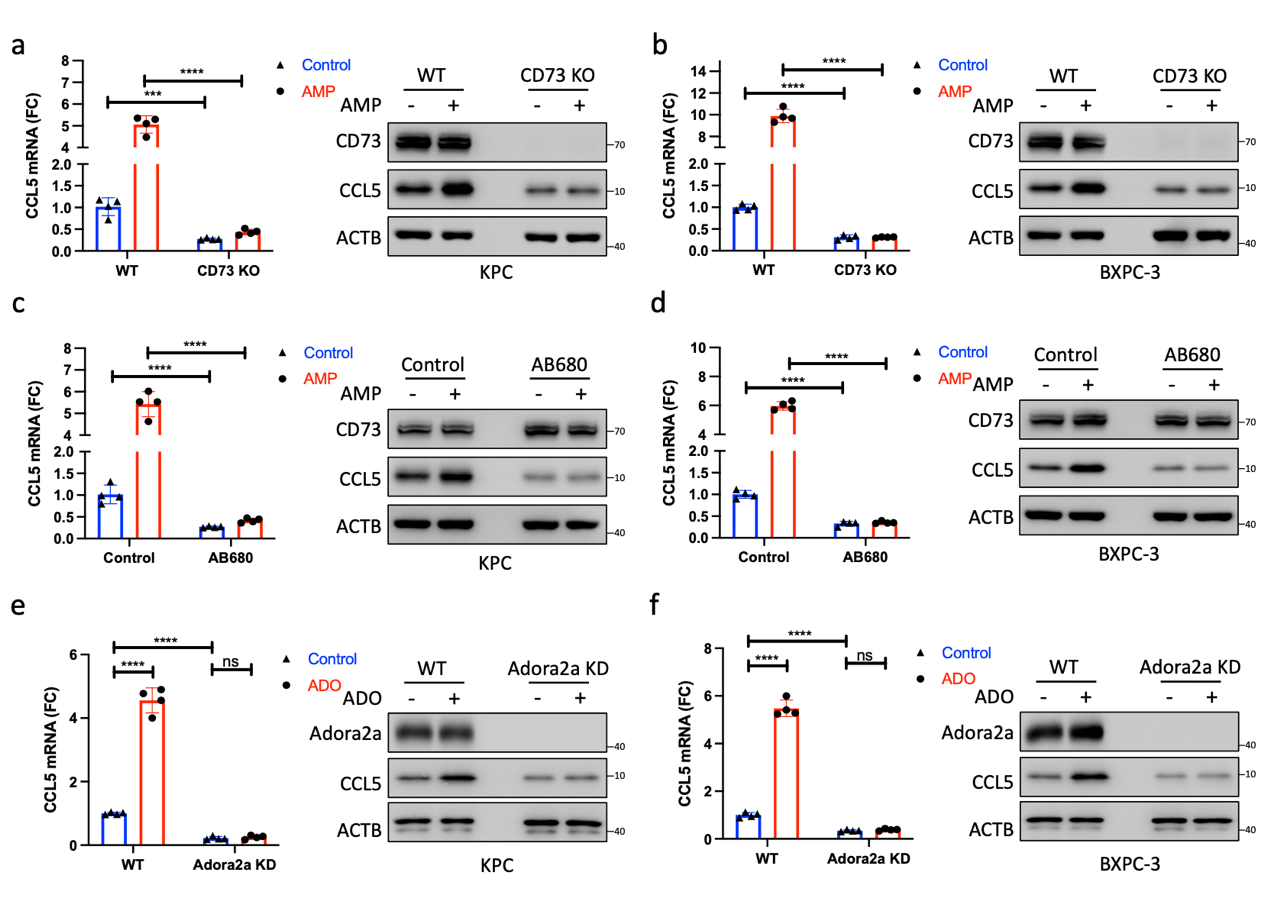
**

**Supplementary Fig. 11: CD73 regulates CCL5 expression by converting AMP to ADO.** (a-b) PCR and immunoblot analyses of CCL5 in WT/CD73 KO KPC cells (a) as well as WT/CD73 KO BXPC-3 cells (b) treated with or without adenosine monophosphate (n=4). (c-d) PCR and immunoblot analyses of CCL5 in KPC cells (c) and BXPC-3 cells (d) treated with or without adenosine monophosphate and AB680 (n=4). (e-f) PCR and immunoblot analyses of CCL5 in KPC cells (e) and BXPC-3 cells (f) treated with or without adenosine and Adora2a depletion (n=4). Results represent means ± SD of one representative experiment in **a-f**. *P < 0.05, **P < 0.01, ***P < 0.001 using a two-tailed t-test; ns: not significant. The exact p values are shown in the Source Data. Source data are provided as a Source Data file.

**
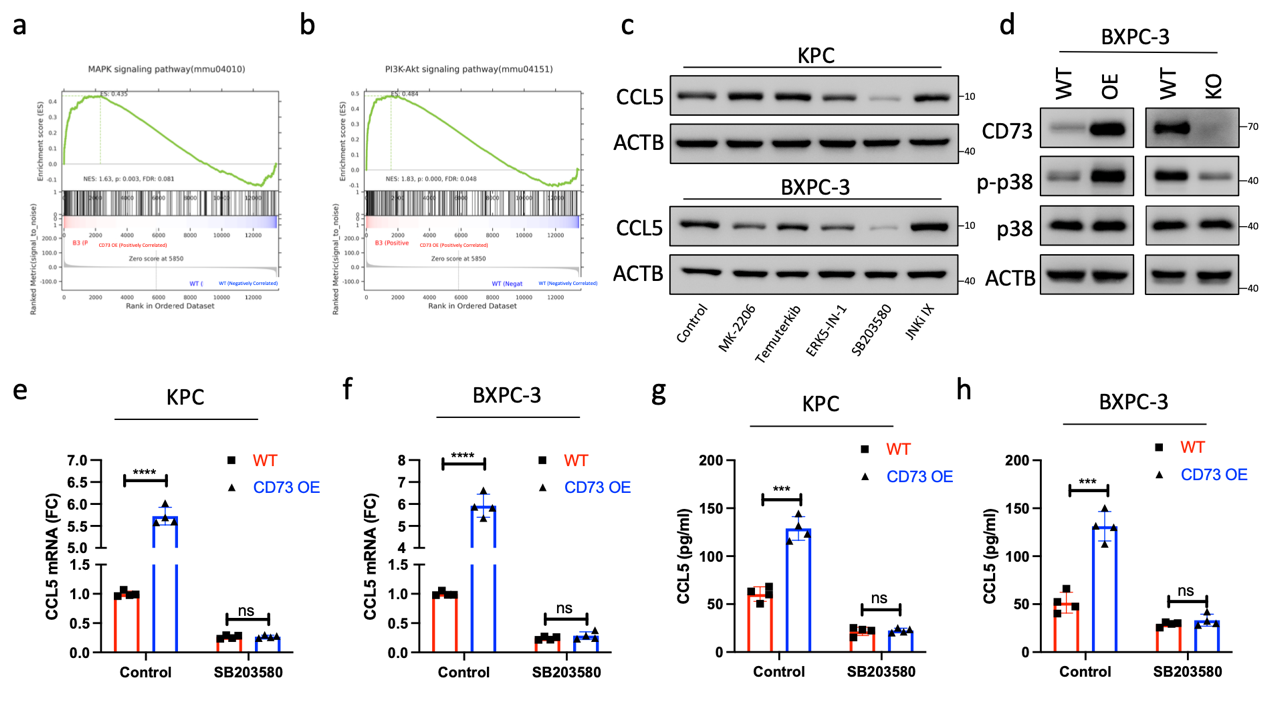
**

**Supplementary Fig. 12: CD73 regulates CCL5 expression via the p38 MAPK pathway.** (a-b) Top differentially regulated pathways identified from transcriptomic data of WT and CD73 OE KPC cells by GSEA, including the MAPK pathway (a) and PI3K–AKT pathway (b). (c) Immunoblot analysis of CCL5 in ADO-preincubated KPC cells and BXCP-3 cells treated with MK-2006, temuterkib, ERK5-IN-1, SB20350, or JNK inhibitor IX. (d) CD73 regulates the phosphorylation of p38. Immunoblot analysis of CD73, p-p38, and p38 in CD73 KO/OE BXPC-3 cells. (e-h) CD73 regulates CCL5 expression through the p38 MAPK pathway. mRNA levels of CCL5 in WT and CD73 OE KPC cells (e) and BXPC-3 cells (f) treated with or without SB203580 (n=4). ELISA of CCL5 in WT and CD73 OE KPC cells (g) and BXPC-3 cells (h) treated with or without SB203580 (n=4). Results represent means ± SD of one representative experiment in **e-h**. *P < 0.05, **P < 0.01, ***P < 0.001 using a two-tailed t-test; ns: not significant. The exact p values are shown in the Source Data. Source data are provided as a Source Data file.

**
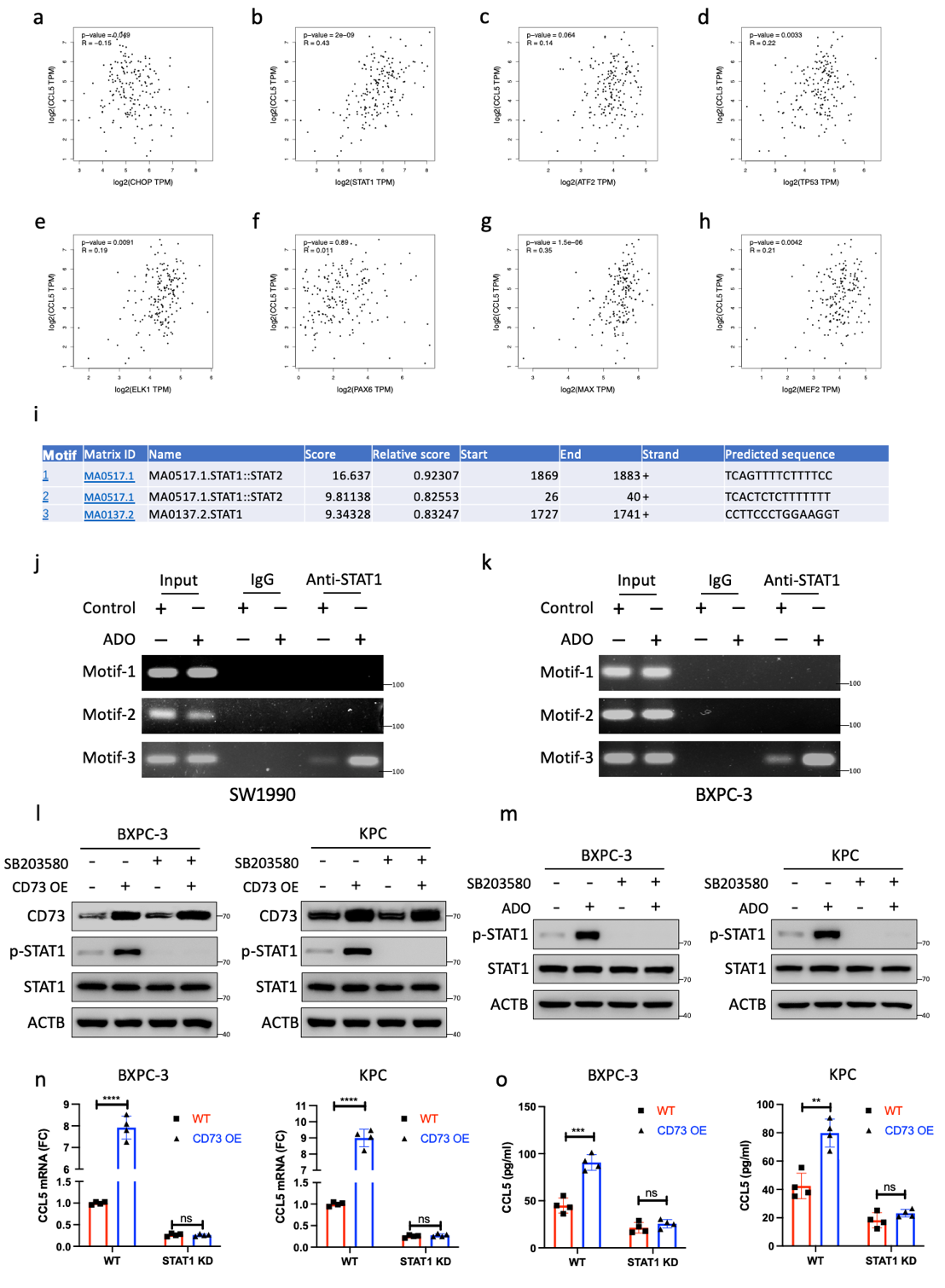
**

**Supplementary Fig. 13: p38 MAPK regulates CCL5 expression via STAT1 phosphorylation.** (a-h) The correlations between the expression of CCL5 and that of the classical downstream transcription factors of the p38 MAPK pathway, including CHOP (a), STAT1 (b), ATF2 (c), TP53 (d), ELK1 (e), PAX6 (f), MAX (g), and MEF2 (h), was evaluated based on the TCGA database. (i) Predicted STAT1 binding sites based on JASPAR database. (j-k) Direct binding of STAT1 to the promoter and enhancer of CCL5. ChIP assay of the binding between STAT1 and three predicted regions in the CCL5 gene promoter in both SW1990 (j) and BXCP-3 (k) cells treated with or without adenosine. (l-m) CD73 regulates CCL5 expression through the p38 MAPK pathway. Immunoblot analysis of CD73, p-STAT1, and STAT1 in WT and CD73 pancreatic cancer cells treated with or without SB203580 (l). Immunoblot analysis of p-STAT1 and STAT1 in pancreatic cancer cells treated with or without adenosine and SB203580 (m). (n-o) Abolition of CCL5 upregulation induced by CD73 overexpression through STAT1 depletion (n=4). PCR (n) and ELISA of (o) CCL5 in WT and CD73 OE pancreatic cells with or without STAT1 depletion (n=4). All data are representative of three independently performed experiments. Results represent means ± SD of one representative experiment in **n-o**. *P < 0.05, **P < 0.01, ***P < 0.001 using a two-tailed t-test; ns: not significant. The Spearman correlations and p-values by Spearman’s test are indicated in **a-h.** The exact p values are shown in the Source Data. Source data are provided as a Source Data file.

**
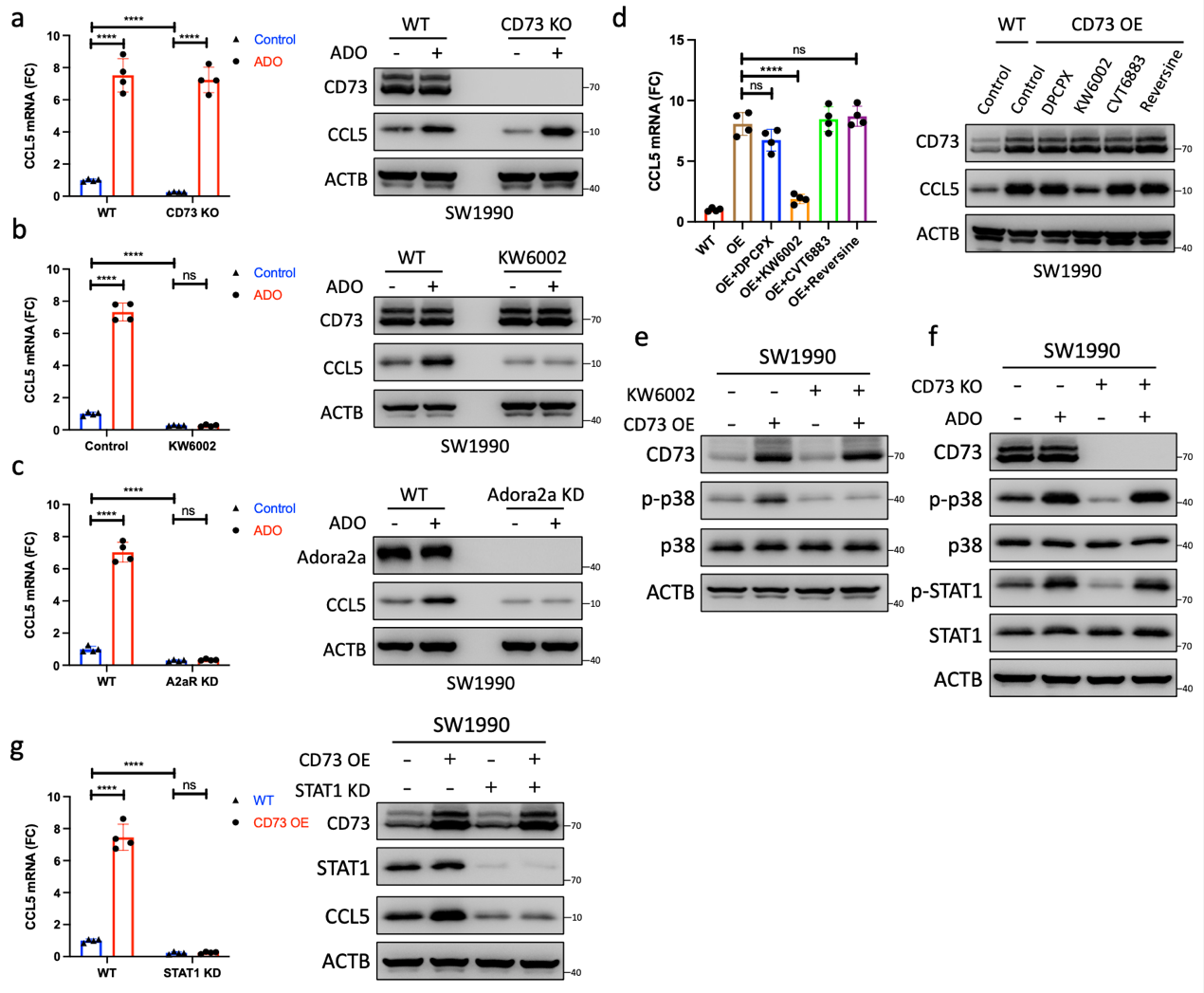
**

**Supplementary Fig. 14: Generality of the phenomenon on pancreatic cell line.** (a) PCR and immunoblot analyses of CCL5 in WT/CD73 KO SW1990 cells treated with or without adenosine (n=4). (b) PCR and immunoblot analyses of CCL5 in SW1990 cells treated with or without KW6002 and adenosine (n=4). (c) PCR and immunoblot analyses of CCL5 in SW1990 cells treated with or without adenosine and Adora2a depletion (n=4). (d) PCR and immunoblot analyses of CCL5 in WT/CD73 OE SW1990 cells treated with antagonists targeting adenosine receptors (Adora1, DPCPX; Adora2a, KW6002; Adora2b, CVT6883; Adora3, reversine) (n=4). (e) Immunoblot analysis of CD73, p-p38, and p38 in CD73 KO/OE cells treated with or without KW6002. (f) Immunoblot analysis of CD73, p-p38, p38, p-STAT1, and STAT1 in CD73 WT/KO SW1990 cells treated with or without adenosine. (g) PCR and immunoblot analyses of CCL5 in WT and CD73 OE SW1990 cells with or without STAT1 depletion (n=4). *P < 0.05, **P < 0.01, ***P < 0.001 using a two-tailed t-test; ns: not significant. The exact p values are shown in the Source Data. Source data are provided as a Source Data file.

**
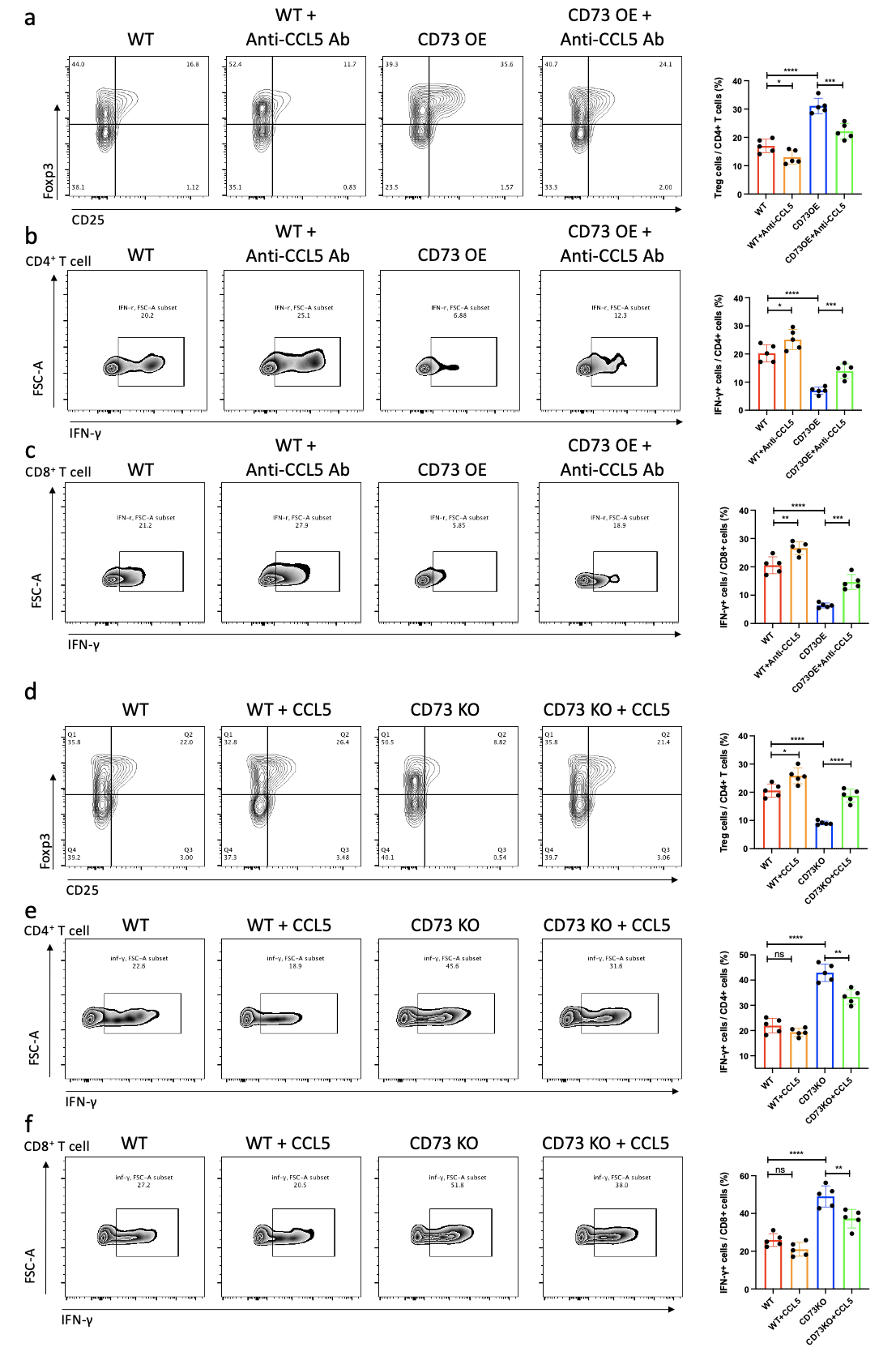
**

**Supplementary Fig. 15: Targeting the tumor cell-autonomous CD73–CCL5 pathway enhanced T-cell activation.** (a-c) Inhibtion of CD73 overexpression-induced orthotopic tumor growth by CCL5 blockade treatment (n=5). Representative images of infiltrated Treg cells (a), activated CD4^+^ T cells (b) and CD8^+^ T cells (c). (d-f) Abolition of CD73 depletion-induced subcutaneous tumor inhibition by intra-tumoral injection of CCL5 (n=5). Representative images of infiltrated Treg cells (d), activated CD4^+^ T cells (e) and CD8^+^ T cells (f). Results represent means ± SD of one representative experiment in **a-f**. *P < 0.05, **P < 0.01, ***P < 0.001 using a two-tailed t-test; ns: not significant. The exact p values are shown in the Source Data. Source data are provided as a Source Data file.


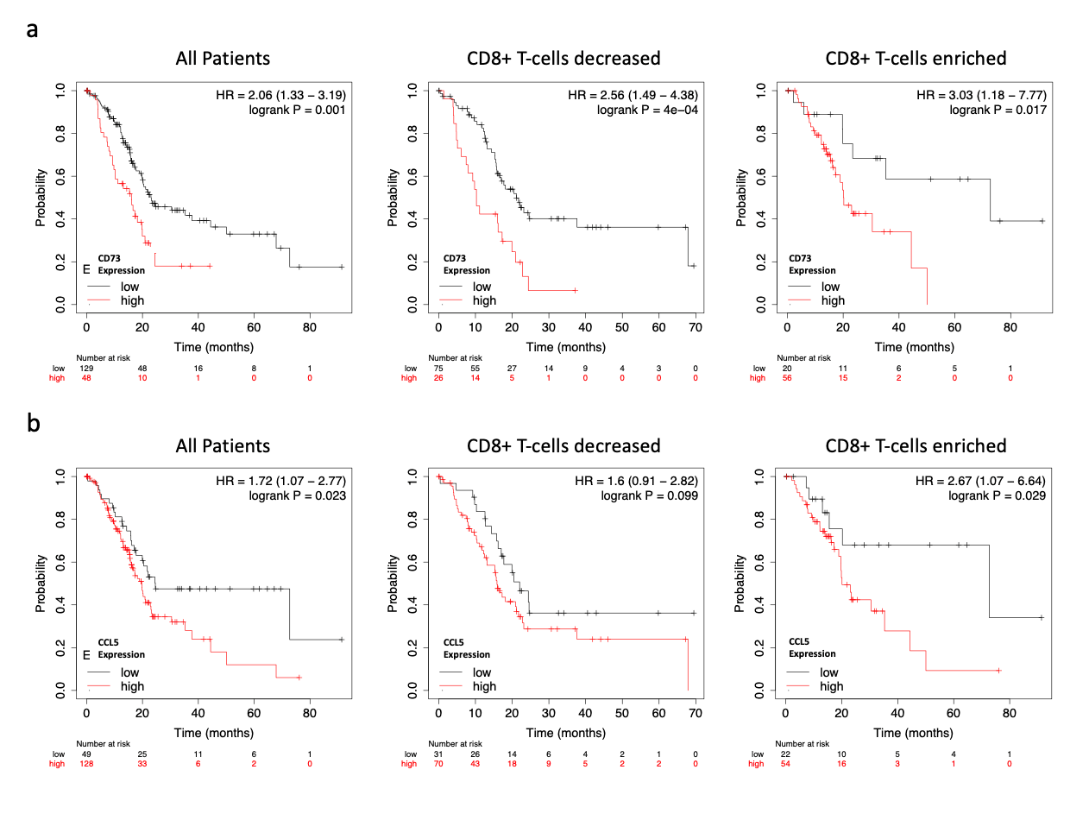


**Supplementary Fig. 16: The prognostic value of the CD73–CCL5 axis in pancreatic cancer.** (a) Survival analysis of CD8^+^ T-cell-enriched, CD8+ T-cell-depleted, and all pancreatic cancer patients with low or high CD73 expression based on the KM plotter database. (b) Survival analysis of CD8^+^ T-cell-enriched, CD8+ T-cell-depleted and all pancreatic cancer patients with low or high CCL5 expression based on the KM plotter database. The Hazard Ratios (HR) and p-values by the log-rank (Mantel–Cox) test are indicated in **a**,**b**.
