# Supplementary material for: Transcriptional control of pancreatic cancer immunosuppression by metabolic enzyme CD73 in a tumor-autonomous and -autocrine manner
Source: Nat Commun. 2023 Jun 8;14:3364. doi: 10.1038/s41467-023-38578-3 (PMC10250326; doi:10.1038/s41467-023-38578-3)
Supplement: Supplementary file 3 — Reporting Summary [file 41467_2023_38578_MOESM3_ESM.pdf]

Corresponding author(s): Xing Huang, Tingbo Liang

Last updated by author(s): Apr 4, 2023

Reporting Summary

Nature Portfolio wishes to improve the reproducibility of the work that we publish. This form provides structure for consistency and transparency in reporting. For further information on Nature Portfolio policies, see our Editorial Policies and the Editorial Policy Checklist.

Statistics

For all statistical analyses, confirm that the following items are present in the figure legend, table legend, main text, or Methods section.

n/a	Confirmed	
☐	☒	The exact sample size (*n*) for each experimental group/condition, given as a discrete number and unit of measurement
☐	☒	A statement on whether measurements were taken from distinct samples or whether the same sample was measured repeatedly
☐	☒	The statistical test(s) used AND whether they are one- or two-sided<br>*Only common tests should be described solely by name; describe more complex techniques in the Methods section.*
☐	☒	A description of all covariates tested
☐	☒	A description of any assumptions or corrections, such as tests of normality and adjustment for multiple comparisons
☐	☒	A full description of the statistical parameters including central tendency (e.g. means) or other basic estimates (e.g. regression coefficient) AND variation (e.g. standard deviation) or associated estimates of uncertainty (e.g. confidence intervals)
☐	☒	For null hypothesis testing, the test statistic (e.g. *F*, *t*, *r*) with confidence intervals, effect sizes, degrees of freedom and *P* value noted<br>*Give P values as exact values whenever suitable.*
☒	☐	For Bayesian analysis, information on the choice of priors and Markov chain Monte Carlo settings
☒	☐	For hierarchical and complex designs, identification of the appropriate level for tests and full reporting of outcomes
☒	☐	Estimates of effect sizes (e.g. Cohen's *d*, Pearson's *r*), indicating how they were calculated

Our web collection on statistics for biologists contains articles on many of the points above.

Software and code

Policy information about availability of computer code

Data collection	Applied Biosystems 7500 Fast Real-Time PCR System (Applied Biosystems) for rt-PCR;<br>ImageScope software 12.1(Leica Biosystems) for IHC;<br>Beckman CytoFLEX LX for FACS;<br>LSRFortessa (BD) for FACS;<br>ChemiScopeTouch (Clinx Science Instruments) for western blotting.<br>Maxquant search engine (v.1.5.2.8)<br>Akoya Vectra Polaris (Akoya Biosciences) for multi-color IHC.
Data analysis	FlowJo software version 10.4 (TreeStar) for analysis of FACS results;<br>Image J software version 1.8.0 (NIH) for quantification of western blotting bands;<br>3D HISTECH quant center software version 2.1 (3D HISTECH Ltd) for quantification of IHC images;<br>GraphPad Prism software version 7.0 (GraphPad Software, Inc. USA) and SPSS 19.0 for all statistical analysis and P values.<br>Akoya Biosciences inForm (Akoya Biosciences) for quantification and analysis of multi-color IHC.

For manuscripts utilizing custom algorithms or software that are central to the research but not yet described in published literature, software must be made available to editors and reviewers. We strongly encourage code deposition in a community repository (e.g. GitHub). See the Nature Portfolio guidelines for submitting code & software for further information.

Data

Policy information about availability of data

All manuscripts must include a data availability statement. This statement should provide the following information, where applicable:

- Accession codes, unique identifiers, or web links for publicly available datasets
- A description of any restrictions on data availability
- For clinical datasets or third party data, please ensure that the statement adheres to our policy

All data generated or analyzed during this study are included in this published article and its supplementary information files.
Fig. 4e, Supplementary Figs. 13a-h, 13i and 16a-b were generated from publicly available databases, including GEPIA2 (http://gepia2.cancer-pku.cn), JASPAR (https://jaspar.genereg.net/) and Kaplan–Meier Plotter (http://kmplot.com/analysis). The RNA-Seq data have been deposited in the Bioproject database under the accession code PRJNA934940. The remaining data can be found in the Article, Supplementary Information or Source Data file, which are provided alongside this paper.

Human research participants

Policy information about studies involving human research participants and Sex and Gender in Research.

Reporting on sex and gender	The findings apply to both sexes. The information involved in sex data was provided in Results and Methods.
Population characteristics	Human pancreatic adenocarcinoma cancer tissue specimens were obtained from male and female PDAC patients, including 32 patients who underwent surgical resection for pancreatic cancer. The cohort consisted of 19 male and 13 female patients with a mean age of 60 years (range: 42-76 years). The majority of patients were diagnosed with stage I (n=10) or stage II (n=14) pancreatic cancer, while 8 patients were diagnosed with stage III disease.
Recruitment	Human pancreatic adenocarcinoma cancer tissue specimens were obtained from the Department of Hepatobiliary and Pancreatic Surgery, the First Affiliated Hospital, School of Medicine, Zhejiang University.
Ethics oversight	The protocol was approved by the Institutional Review Board at the First Affiliated Hospital, School of Medicine, Zhejiang University. Written informed consent was obtained from each patient at the time of enrollment.

Note that full information on the approval of the study protocol must also be provided in the manuscript.

Field-specific reporting

Please select the one below that is the best fit for your research. If you are not sure, read the appropriate sections before making your selection.

☒ Life sciences ☐ Behavioural & social sciences ☐ Ecological, evolutionary & environmental sciences

For a reference copy of the document with all sections, see nature.com/documents/nr-reporting-summary-flat.pdf

Life sciences study design

All studies must disclose on these points even when the disclosure is negative.

Sample size	Sample sizes based on several factors, including the variability of the data, the purpose of the experiment, the available resources, and previous research in the field. Previous publications containing similar procedure to maintain the balance between reaching the statistical significance and minimizing the number of animal/reagents use. Published papers was used as references (PMID:34315872; 35288467; 30786811; 32768595).
Data exclusions	No data was excluded.
Replication	All data from at least three biological replications are presented as mean ± SD. We confirm that all attempts at replication were successful.
Randomization	For in vivo experiments, animals were randomly allocated to each group as described in the form below. For cell line based experiments, randomization was not required because all samples were analyzed equally.
Blinding	Cellular and biochemical experiments were not performed in a blinding manner, since data is quantitative nature. As for animal experiments, two individual investigators were blind to group allocation during data collection and analysis. The first investigator prepared the drugs and labeled their names with ABCD, and then the second investigator performed drug injection and subsequent measurement and analysis according to the ABCD serial number.

Reporting for specific materials, systems and methods

We require information from authors about some types of materials, experimental systems and methods used in many studies. Here, indicate whether each material, system or method listed is relevant to your study. If you are not sure if a list item applies to your research, read the appropriate section before selecting a response.

Materials & experimental systems

n/a	Involved in the study
☐	☒ Antibodies
☐	☒ Eukaryotic cell lines
☒	☐ Palaeontology and archaeology
☐	☒ Animals and other organisms
☒	☐ Clinical data
☒	☐ Dual use research of concern

Methods

n/a	Involved in the study
☒	☐ ChIP-seq
☐	☒ Flow cytometry
☒	☐ MRI-based neuroimaging

Antibodies

Antibodies used

The following Abs were used for in vivo experiments: Rat anti-CCL5 Antibody (Clone # 53405 , Cat#MAB478, R&D Systems, 50 μg/mouse), Rat anti-mouse PD-1 antibody (Clone:RMP1-14, Cat#BE0146, Bio X Cell, 120 μg/mouse), Rat anti-mouse CD8α antibody (Clone:2.43, Cat#BE0146, Bio X Cell, 250 μg/mouse), Rat anti-mouse CD4 antibody (Clone:YTS 191, Cat#BE0119, Bio X Cell, 250 μg/mouse) and Rat anti-mouse NK1.1 antibody (Clone:PK136, Cat#BE0036, Bio X Cell, 250 μg/mouse). The following Abs were used for immunoblotting: rabbit anti-CD73 (Clone: D7F9A Cat#13160, Cell Signaling Technology, 1:2000 for WB, 1:100 for ICH), rabbit anti-CCL5 (Clone: E9S2K, Cat#36467, Cell Signaling Technology, 1:2000 for WB, 1:150 for ICH), rabbit anti-p38 MAPK (D13E1, Cat#8690, Cell Signaling Technology, 1:2000 for WB), rabbit anti-phospho-p38 MAPK (D3F9, Cat#4511, Cell Signaling Technology, 1:2000 for WB), rabbit anti-STAT1 (D1K9Y, Cat#14994, Cell Signaling Technology, 1:2000 for WB), rabbit anti-phospho-Stat1 (58D6, Cat#9167, Cell Signaling Technology, 1:2000 for WB), rabbit anti-phospho-Stat1 (D3B7, Cat#8826, Cell Signaling Technology, 1:2000 for WB), rabbit anti-β-Actin (8H10D10, Cat#3700, Cell Signaling Technology, 1:1000 for WB), anti-mouse HRP-linked secondary Ab (Cat#7076, Cell Signaling Technology, 1:5000 for WB), and anti-rabbit HRP-linked secondary Ab (Cat#7074, Cell Signaling Technology, 1:5000 for WB). The following Abs were used for flow cytometry: Brilliant Violet 605 anti-CD45 (I3/2.3, Cat#BD567459, BD Biosciences, 1:1000 for flow cytometry), FITC anti-CD3 (17A2, Cat#BD555274, BD Biosciences, 1:1000 for flow cytometry), APC anti-CD49b (HMα2, Cat#BD558295, BD Biosciences, 1:1000 for flow cytometry), APC-Cy7 anti-CD4 (GK1.5, Cat#BD561830, BD Biosciences, 1:1000 for flow cytometry), PE-Cy7 anti-CD8 (53-6.7, Cat#BD552877, BD Biosciences, 1:1000 for flow cytometry), Brilliant Violet 510 anti-CD44 (IM7, Cat#BD563114, BD Biosciences, 1:1000 for flow cytometry), PE anti-CD62L (MEL-14, Cat#BD553151, BD Biosciences, 1:1000 for flow cytometry), Brilliant Violet 786 anti-CD69 (H1.2F3, Cat#BD564683, BD Biosciences, 1:1000 for flow cytometry), AF700 anti-CD25 (PC61, Cat#102024, BioLegend, 1:1000 for flow cytometry), PE-CF594 rat anti-CD11b (ICRF44, Cat#562399, BD Biosciences, 1:1000 for flow cytometry), PE anti-Foxp3 (3G3, Cat#566881, BD Biosciences, 1:1000 for flow cytometry), PerCP/Cyanine5.5 anti-granzyme B (QA18A28, Cat#396412, BioLegend, 1:1000 for flow cytometry), PE anti-TNF alpha (TN3-19.12, Cat#12-7423-41, eBioscience, 1:1000 for flow cytometry), and APC anti-IFN-γ (4S.B3, Cat#17-7319-82, eBioscience, 1:1000 for flow cytometry).

Validation

All antibodies were validated by manufacturers detailed information could be found on website.
Rat anti-CCL5 Antibody (Cat#MAB478, R&D Systems) https://www.rndsystems.com/cn/products/mouse-ccl5-rantes-antibody-53405_mab478
Rat anti-mouse PD-1 antibody (Cat#BE0146, Bio X Cell) https://bioxcell.com/invivomab-anti-mouse-pd-1-cd279-be0146
Rat anti-mouse CD8α antibody (BE0146, Bio X Cell) https://bioxcell.com/invivomab-anti-mouse-cd8a-be0061
Rat anti-mouse CD4 antibody (Cat#BE0119, Bio X Cell) https://bioxcell.com/invivomab-anti-mouse-cd4-be0119
Rat anti-mouse NK1.1 antibody (Cat#BE0036, Bio X Cell) https://bioxcell.com/invivomab-anti-mouse-nk1-1-be0036
rabbit anti-CD73 (Cell Signaling Technology, 1:2000 for WB) https://www.cellsignal.com/products/primary-antibodies/nt5e-cd73-d7f9a-rabbit-mab/13160
rabbit anti-p38 MAPK (Cat#8690, Cell Signaling Technology) https://www.cellsignal.com/products/primary-antibodies/p38-mapk-d13e1-xp-rabbit-mab/8690
rabbit anti-phospho-p38 MAPK (Cat#4511, Cell Signaling Technology) https://www.cellsignal.com/products/primary-antibodies/phospho-p38-mapk-thr180-tyr182-d3f9-xp-rabbit-mab/4511
rabbit anti-STAT1 (Cat#14994, Cell Signaling Technology) https://www.cellsignal.com/products/primary-antibodies/stat1-d1k9y-rabbit-mab/14994
rabbit anti-phospho-Stat1 (Cat#9167, Cell Signaling Technology) https://www.cellsignal.com/products/primary-antibodies/phospho-stat1-tyr701-58d6-rabbit-mab/9167
rabbit anti-phospho-Stat1 (Cat#8826, Cell Signaling Technology) https://www.cellsignal.com/products/primary-antibodies/phospho-stat1-ser727-d3b7-rabbit-mab/8826?_=1680626340582&Ntt=8826&tahead=true
rabbit anti-β-Actin (Cat#3700, Cell Signaling Technology) https://www.cellsignal.com/products/primary-antibodies/b-actin-8h10d10-mouse-mab/3700
anti-mouse HRP-linked secondary Ab (Cat#7076, Cell Signaling Technology) https://www.cellsignal.com/products/secondary-antibodies/anti-mouse-igg-hrp-linked-antibody/7076
anti-rabbit HRP-linked secondary Ab (Cat#7074, Cell Signaling Technology) https://www.cellsignal.com/products/secondary-antibodies/anti-rabbit-igg-hrp-linked-antibody/7074?_=1680626638853&Ntt=7074&tahead=true
Brilliant Violet 605 anti-CD45 (Cat#BD567459, BD Biosciences) https://www.bdbiosciences.com/zh-cn/products/reagents/flow-cytometry-reagents/research-reagents/single-color-antibodies-ruo/bv605-rat-anti-mouse-cd45.567459
FITC anti-CD3 (Cat#BD555274, BD Biosciences)
https://www.bdbiosciences.com/zh-cn/products/reagents/flow-cytometry-reagents/research-reagents/single-color-antibodies-ruo/fitc-rat-anti-mouse-cd3-molecular-complex.555274
APC anti-CD49b (Cat#BD558295, BD Biosciences)
https://www.bdbiosciences.com/zh-cn/products/reagents/flow-cytometry-reagents/research-reagents/single-color-antibodies-ruo/

apc-hamster-anti-mouse-cd49b.558295
APC-Cy7 anti-CD4 (Cat#BD561830, BD Biosciences) https://www.bdbiosciences.com/zh-cn/products/reagents/flow-cytometry-reagents/research-reagents/single-color-antibodies-ruo/apc-cy-7-rat-anti-mouse-cd4.561830
PE-Cy7 anti-CD8 (Cat#BD552877, BD Biosciences) https://www.bdbiosciences.com/zh-cn/products/reagents/flow-cytometry-reagents/research-reagents/single-color-antibodies-ruo/pe-cy-7-rat-anti-mouse-cd8a.552877
Brilliant Violet 510 anti-CD44 (Cat#BD563114, BD Biosciences) https://www.bdbiosciences.com/zh-cn/products/reagents/flow-cytometry-reagents/research-reagents/single-color-antibodies-ruo/bv510-rat-anti-mouse-cd44.563114
Brilliant Violet 786 anti-CD69 (Cat#BD564683, BD Biosciences) https://www.bdbiosciences.com/zh-cn/products/reagents/flow-cytometry-reagents/research-reagents/single-color-antibodies-ruo/bv786-hamster-anti-mouse-cd69.564683
PE-CF594 rat anti-CD11b (Cat#562399, BD Biosciences) https://www.bdbiosciences.com/zh-cn/products/reagents/flow-cytometry-reagents/research-reagents/single-color-antibodies-ruo/pe-cf594-mouse-anti-human-cd11b.562399
PE anti-Foxp3 (Cat#566881, BD Biosciences) https://www.bdbiosciences.com/zh-cn/products/reagents/flow-cytometry-reagents/research-reagents/single-color-antibodies-ruo/pe-mouse-anti-mouse-foxp3.566881
PE anti-TNF alpha (Cat#12-7423-41, eBioscience) https://www.thermofisher.cn/cn/zh/antibody/product/TNF-alpha-Antibody-clone-TN3-19-12-Monoclonal/12-7423-41
APC anti-IFN-γ (Cat#17-7319-82, eBioscience) https://www.thermofisher.cn/cn/zh/antibody/product/IFN-gamma-Antibody-clone-4S-B3-Monoclonal/17-7319-82
AF700 anti-CD25 (Cat#102024, BioLegend) https://www.biolegend.com/en-us/products/alexa-fluor-700-anti-mouse-cd25-antibody-3389
PerCP/Cyanine5.5 anti-granzyme B (Cat#396412, BioLegend) https://www.biolegend.com/en-us/products/percp-cyanine5-5-anti-humanmouse-granzyme-b-recombinant-antibody-17879

Eukaryotic cell lines

Policy information about cell lines and Sex and Gender in Research

Cell line source(s)	The KPC cell line, derived from the spontaneous tumor of a KrasLSL-G12D; Trp53LSL-R172H; Pdx1-Cre mouse model, was a kind gift from the laboratory of Prof. Raghu Kalluri (MD Anderson Cancer Center, Houston, TX, USA). BXPC-3,PANC-1,PANC02, SW1990 cell lines were purchased from the ATCC (American Type Culture Collection).
Authentication	To authenticate cell lines, we used short tandem repeat profiling method as exhaustively described by Asadi Jahanbakhsh and colleagues (Khosravi Ayyoob et al, Tumor Biol. (2016) 37:3197-3204).
Mycoplasma contamination	All cell lines used for experiments in this study were routinely subjected to examination of mycoplasma contamination using a MycoAlert Mycoplasma Detection Kit purchased from Lonza (Catalog number LT07). All cell lines were tested negative for mycoplasma contamination.
Commonly misidentified lines (See ICLAC register)	None of the cell lines used in this work was listed as "Misidentified Cell Line" in the ICLAC database. Furthermore, we have authenticated all the cell lines used in this study.

Animals and other research organisms

Policy information about studies involving animals; ARRIVE guidelines recommended for reporting animal research, and Sex and Gender in Research

Laboratory animals	C57BL/6J (male, 6 weeks old) and BALB/c (male, 6 weeks old) mice were purchased from the Nanjing Biomedical Research Institute of Nanjing University. CD73 (NT5E) knockout mice were generated by Cyagen Biosciences Inc. Mice were maintained in a specific-pathogen-free (SPF) environment in the Experimental Animal Center, the First Affiliated Hospital, School of Medicine, Zhejiang University. Mice were maintained at macroenvironmental temperature of 21-22°C, humidity (48-52%), in a conventional 12:12 light/dark cycle with lights on at 6:00 a.m. and off at 6:00 p.m
Wild animals	This study did not involve wild animals.
Reporting on sex	The findings apply to both sexes.
Field-collected samples	This study did not involve the sample-collected from field.
Ethics oversight	All animal experiments were approved by the Ethics Committee of the First Affiliated Hospital, School of Medicine, Zhejiang University, and the animals' suffering was minimized to improve their welfare.

Note that full information on the approval of the study protocol must also be provided in the manuscript.

Flow Cytometry

Plots

Confirm that:

☒ The axis labels state the marker and fluorochrome used (e.g. CD4-FITC).

☒ The axis scales are clearly visible. Include numbers along axes only for bottom left plot of group (a 'group' is an analysis of identical markers).

☒ All plots are contour plots with outliers or pseudocolor plots.

☒ A numerical value for number of cells or percentage (with statistics) is provided.

Methodology

Sample preparation	Samples tissues were mechanically dissociated into small pieces using scissors and scalpels, placed in DMEM containing 2% FBS, collagenase IV (1 mg/ml) (17104019, Thermo Fisher Scientific), DNase (10 µg/ml) (D5025, Sigma-Aldrich), Dispase (0.6 mg/ml) (17105041, Gibco) plus CaCl2 (3 mM) (21115, Sigma-Aldrich), and then incubated at 37 °C under shaking at 200 rpm for 40 to 60 min. Digestion was stopped by the addition of RPMI containing 10% FBS, and dissociated tissues were filtered through 40-µm Cell Strainers (08-771-1, Thermo Fisher Scientific) and washed in PBS. Cells were resuspended in 36% Percoll solution (GE Healthcare) containing 4% 10 PBS and 60% serum-free DMEM and subjected to density gradient centrifugation to remove non-immune cells.
Instrument	Beckman CytoFLEX LX, BD FORTESSA
Software	FlowJo software version 10.4
Cell population abundance	1:1000 of LIVE/DEAD Fixable Violet Dead Cell Stain Kit for live/dead discrimination (L34955, Thermo Fisher Scientific).
Gating strategy	Forward scatter area (FSC-A) vs. side scatter height (SSC-H) with gate 1 to separate grouping cell populations and debris. Side scatter area (SSC-A) vs. side scatter height (SSC-H) with gate 2 to separate single cells from aggregates. Forward scatter area (FSC-A) vs. Viability dyes (LIVE/DEAD Fixable Violet Dead Cell Stain Kit, for 405 nm excitation) with gate 3 to exclude dead cells. Subsetting gates rely on the expression levels of markers in the analysis, and what those markers identify.

☒ Tick this box to confirm that a figure exemplifying the gating strategy is provided in the Supplementary Information.
