# Supplementary material for: Transcriptional control of pancreatic cancer immunosuppression by metabolic enzyme CD73 in a tumor-autonomous and -autocrine manner
Source: Nat Commun. 2023 Jun 8;14:3364. doi: 10.1038/s41467-023-38578-3 (PMC10250326; doi:10.1038/s41467-023-38578-3)

Figure 4g

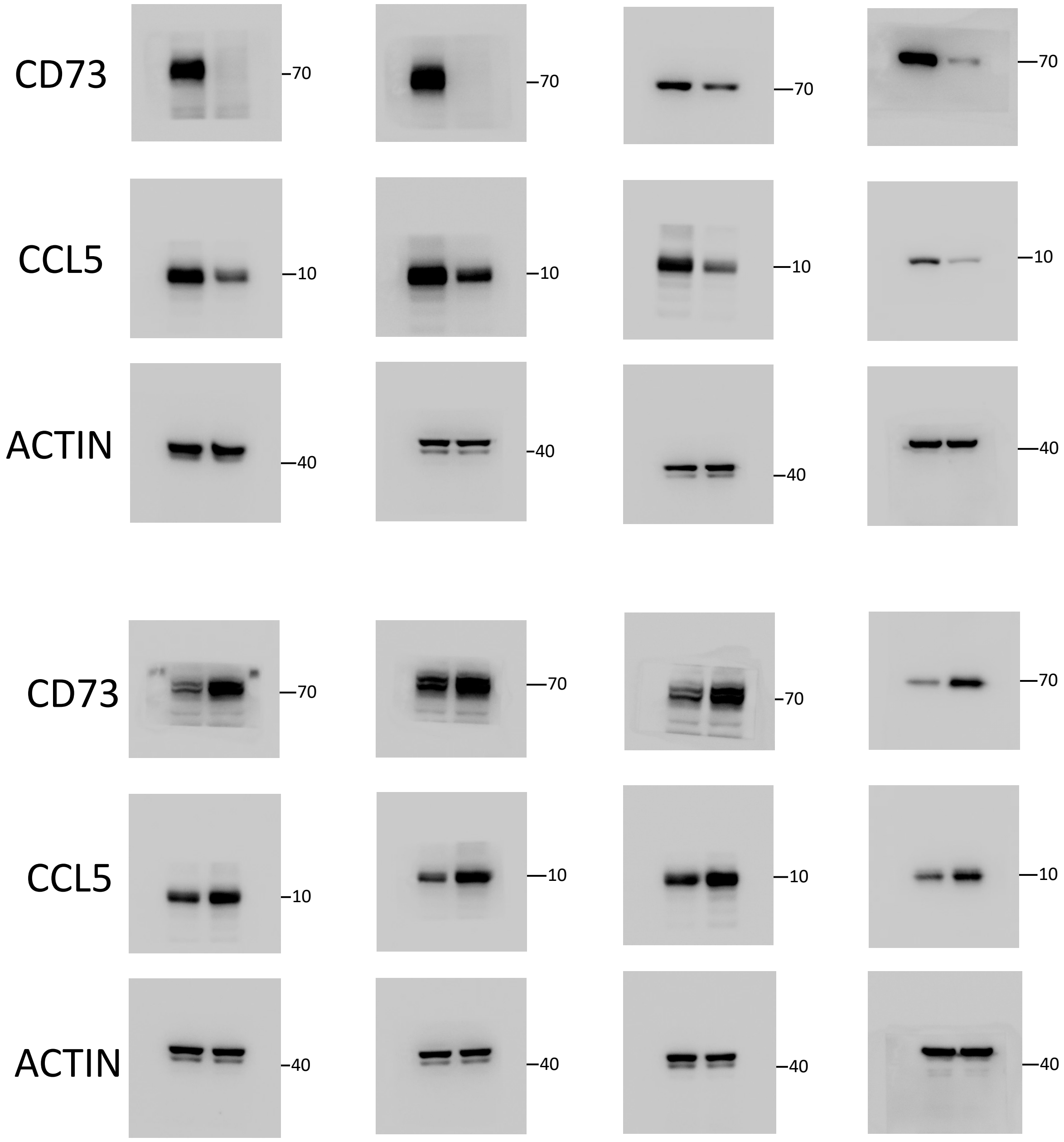
CD73
70
70
70
70
CCL5
10
10
10
10
ACTIN
40
40
40
40
CD73
70
70
70
70
CCL5
10
10
10
10
ACTIN
40
40
40
40

Figure 4i

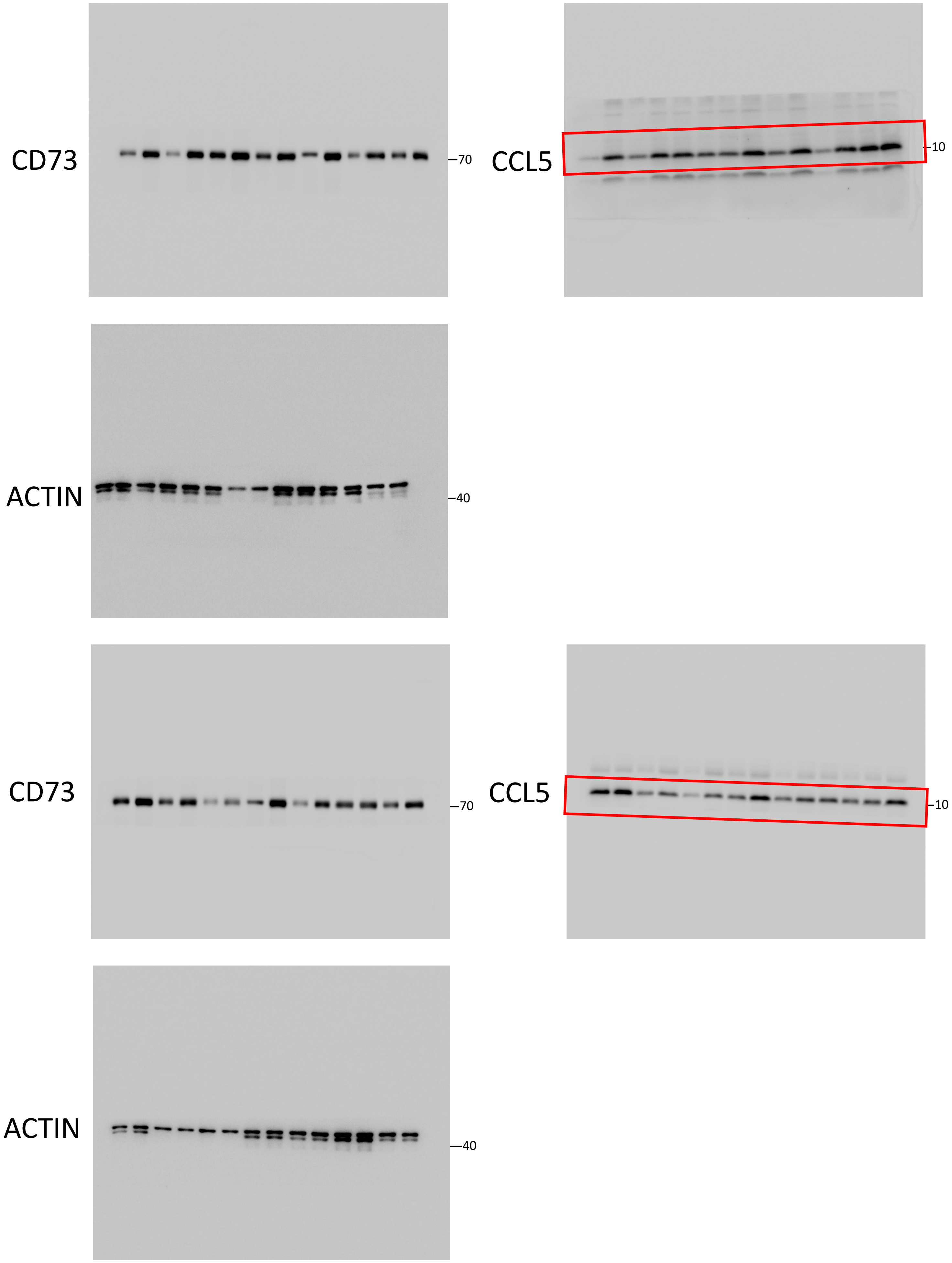

Supplement: Supplementary file 4 — Source data [file 41467_2023_38578_MOESM4_ESM.zip › Source data/Figure 4/Figure 4-uncropped gels.pdf]
