# Supplementary material for: Transcriptional control of pancreatic cancer immunosuppression by metabolic enzyme CD73 in a tumor-autonomous and -autocrine manner
Source: Nat Commun. 2023 Jun 8;14:3364. doi: 10.1038/s41467-023-38578-3 (PMC10250326; doi:10.1038/s41467-023-38578-3)

Supplementary Figure 13j

Motif1

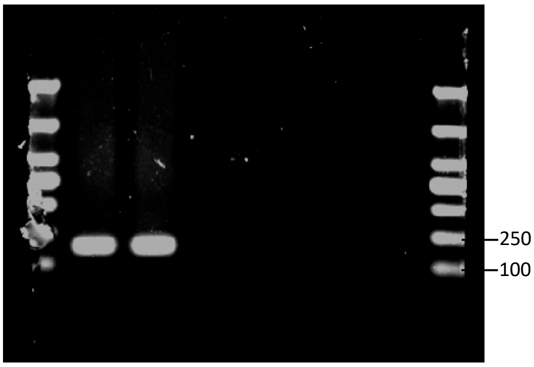

Motif2

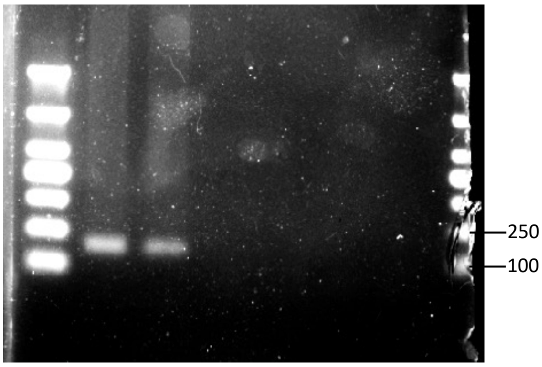

Motif3

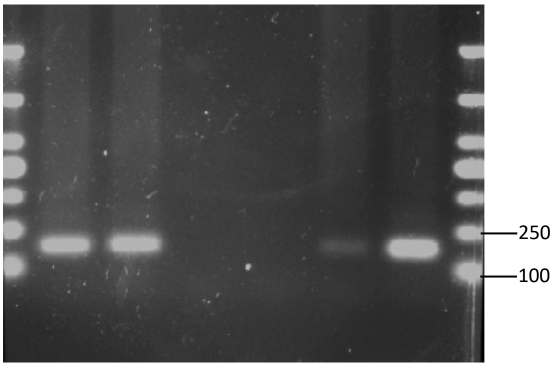

Supplementary Figure 13k

Motif1

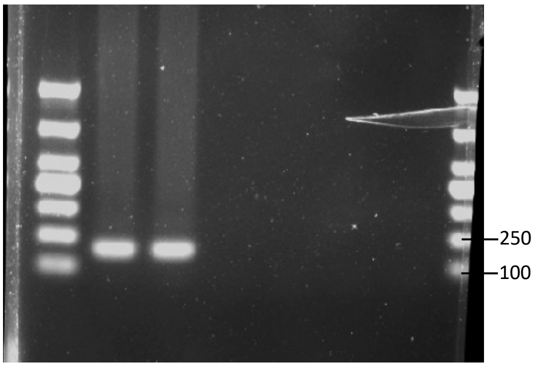

Motif2

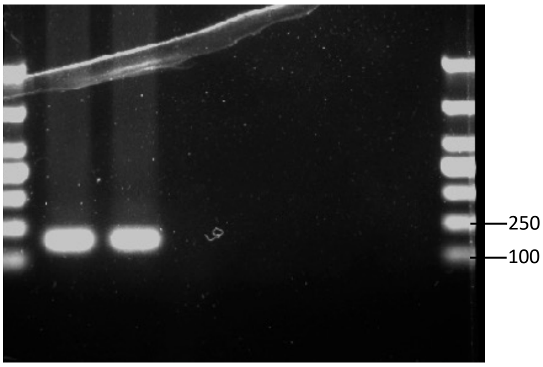

Motif3

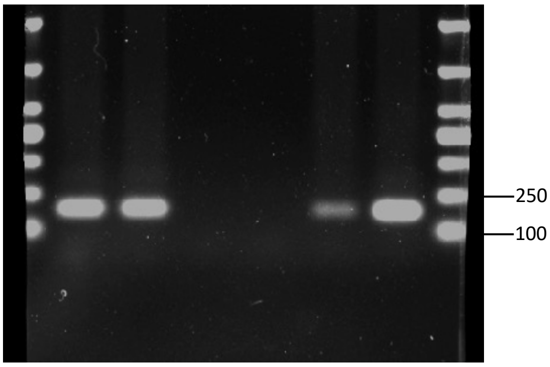

Supplementary Figure 13l

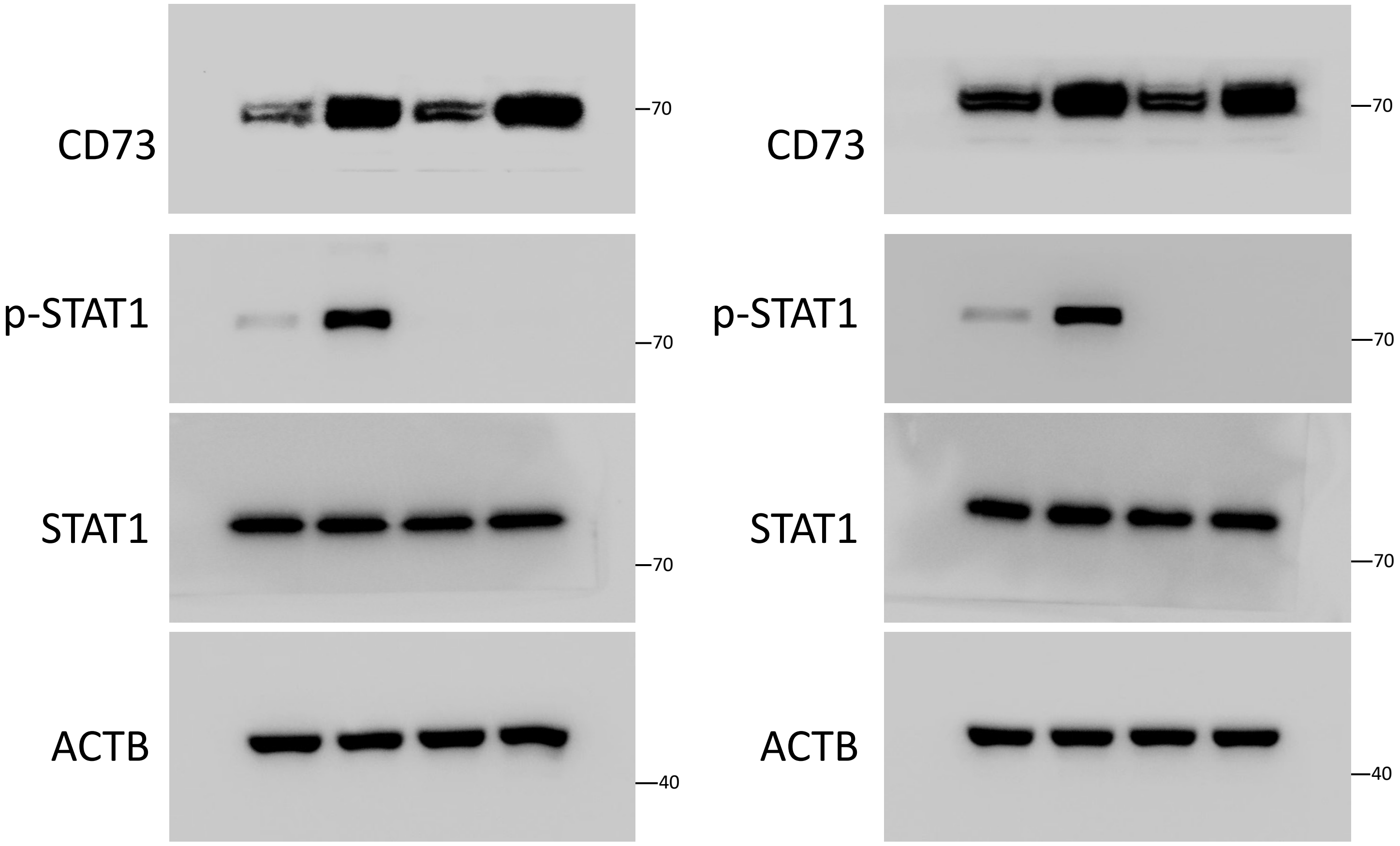

Supplementary Figure 13m

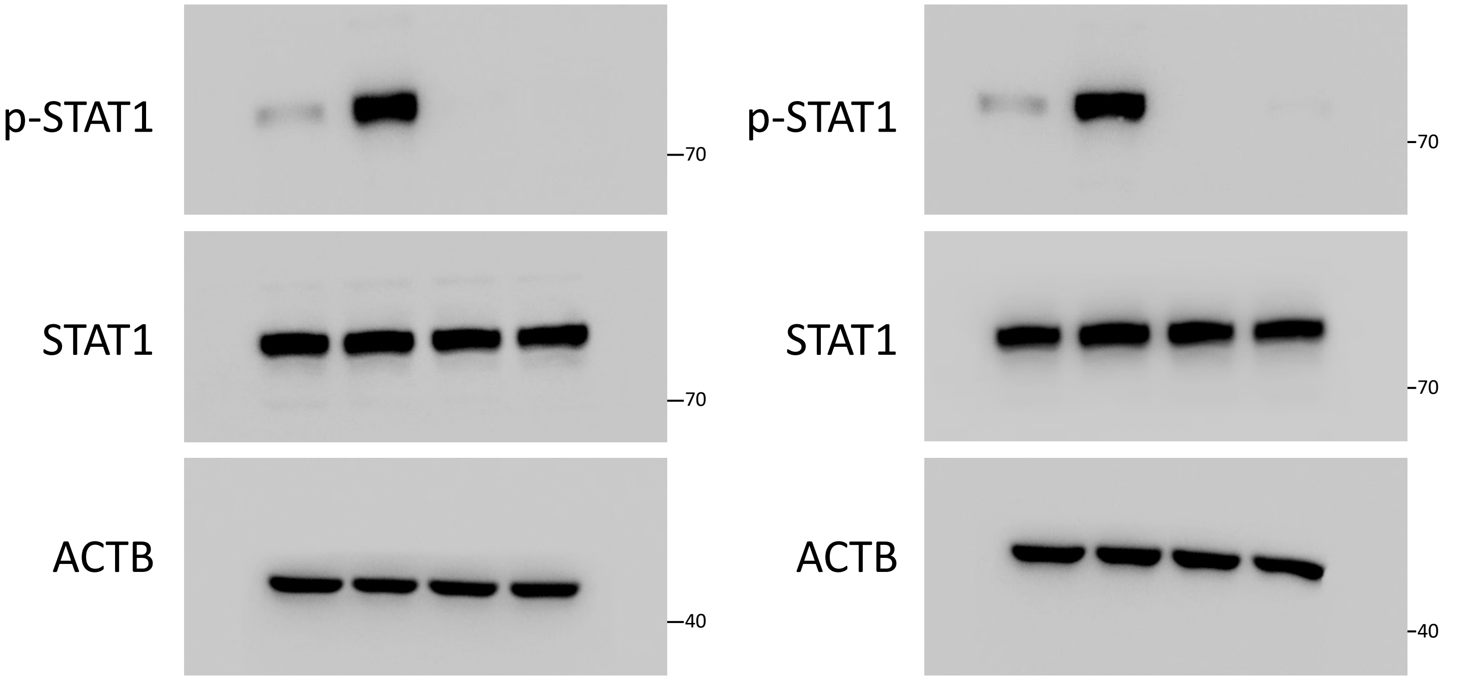

Supplement: Supplementary file 4 — Source data [file 41467_2023_38578_MOESM4_ESM.zip › Source data/Supplementary Figure 13/Supplementary Figure 13-uncropped gels.pdf]
