# Supplementary figures and images for: Transcriptional control of pancreatic cancer immunosuppression by metabolic enzyme CD73 in a tumor-autonomous and -autocrine manner
Source: Nat Commun. 2023 Jun 8;14:3364. doi: 10.1038/s41467-023-38578-3 (PMC10250326; doi:10.1038/s41467-023-38578-3)

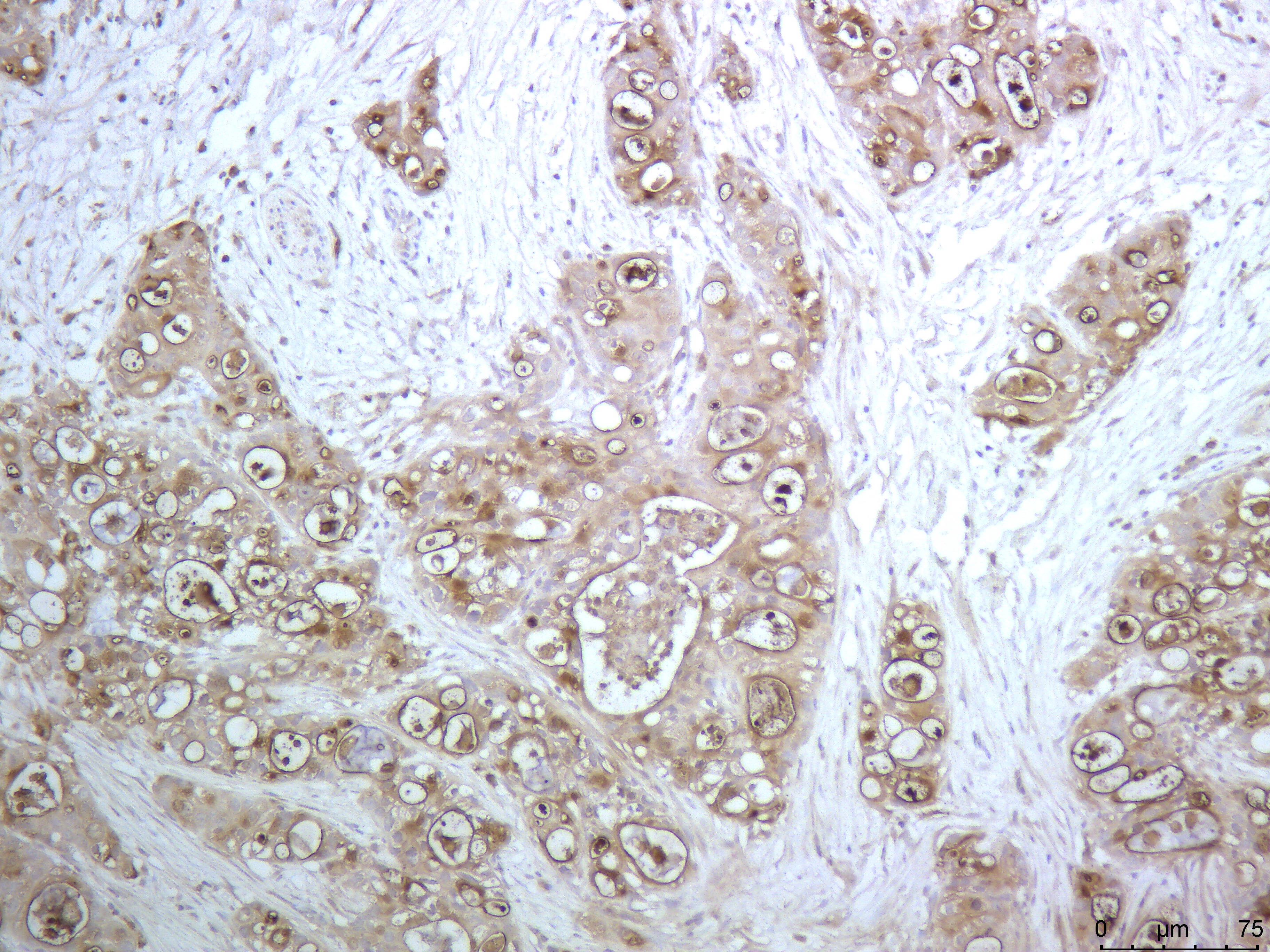

Supplement: Supplementary file 4 — Source data [file 41467_2023_38578_MOESM4_ESM.zip › Source data/Figure 2/Figure 2f/HUMAN CD73 HIGH CD73.tif]

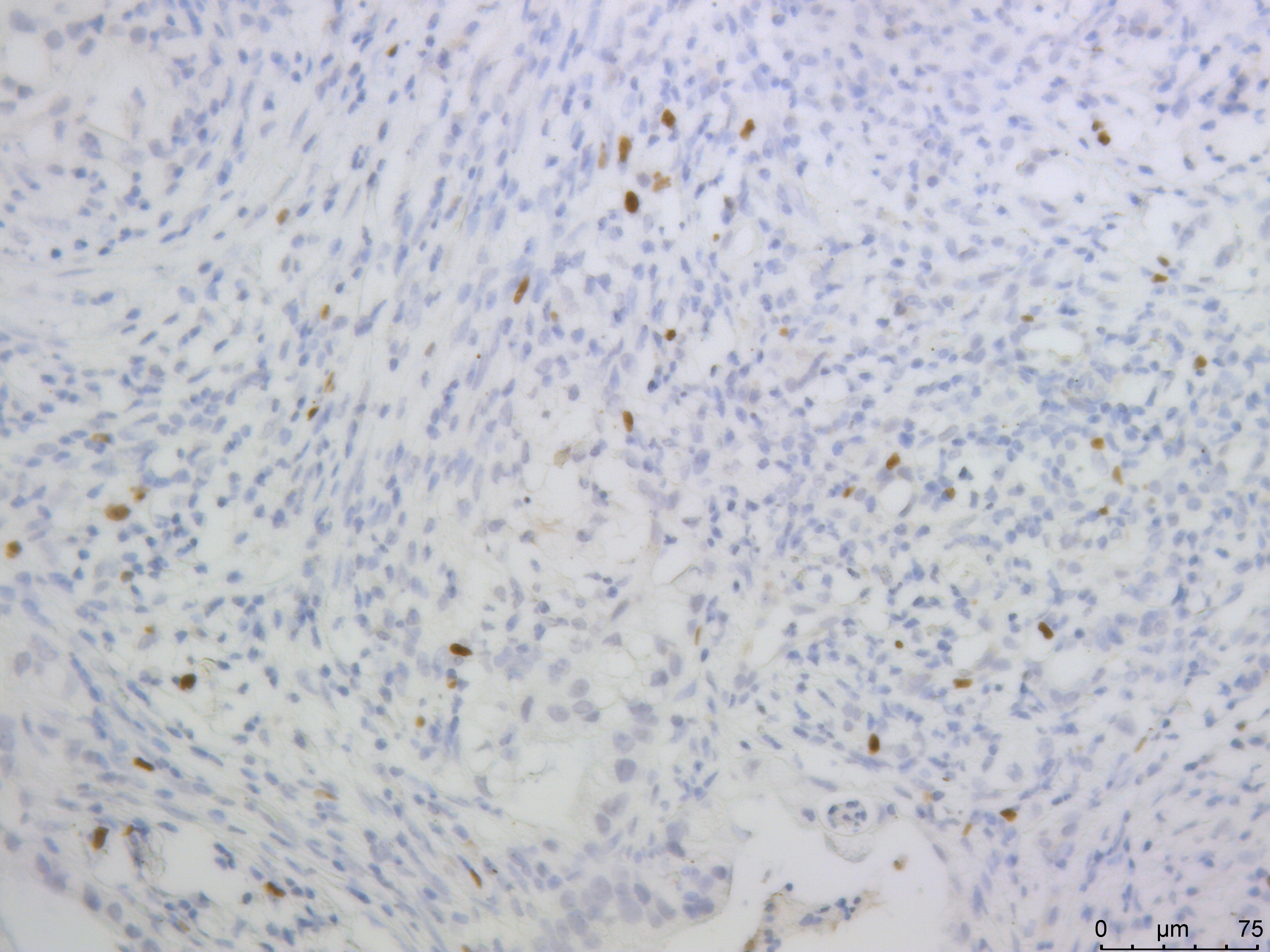

Supplement: Supplementary file 4 — Source data [file 41467_2023_38578_MOESM4_ESM.zip › Source data/Figure 2/Figure 2f/HUMAN CD73 HIGH FOXP3.tif]

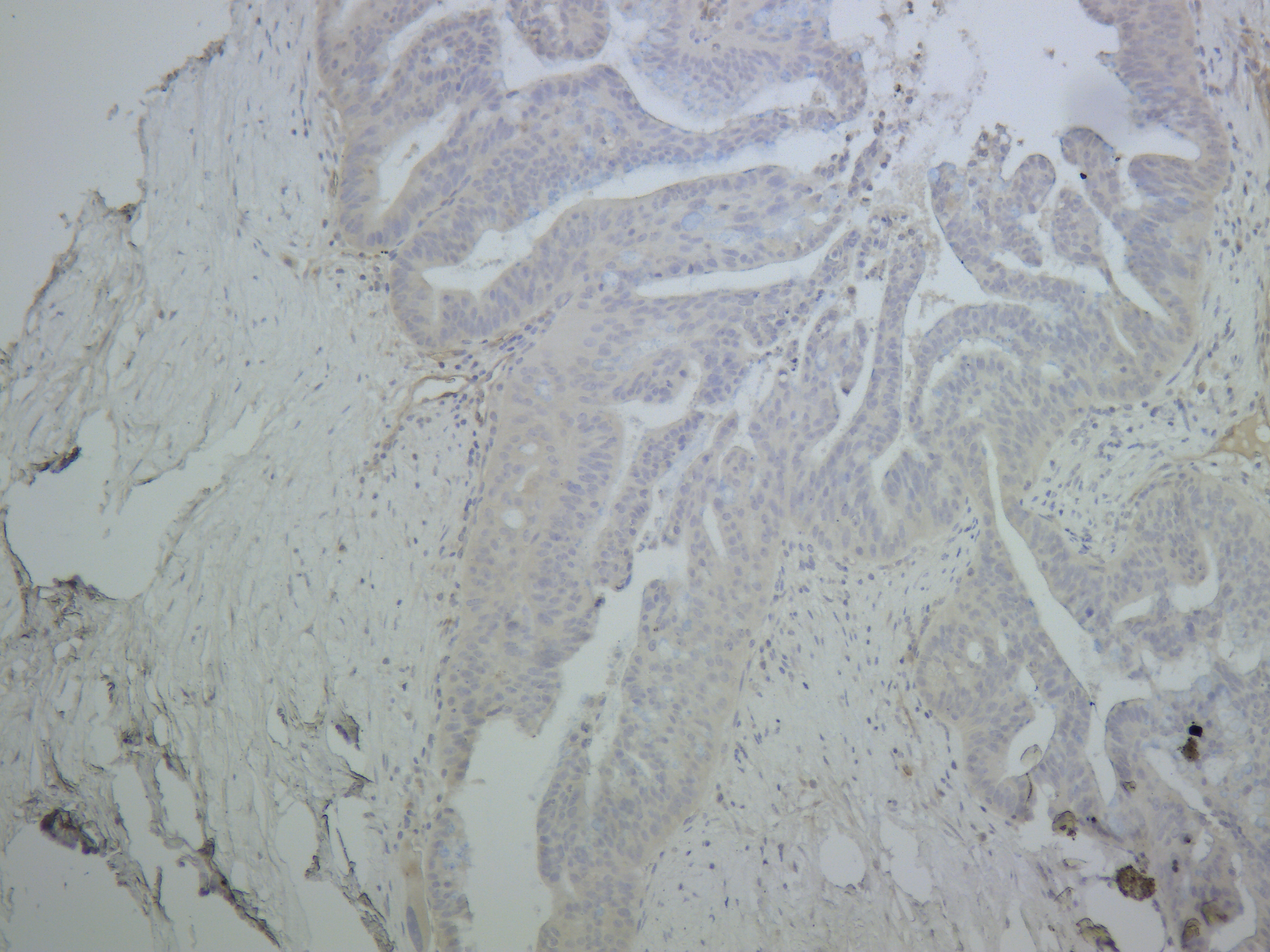

Supplement: Supplementary file 4 — Source data [file 41467_2023_38578_MOESM4_ESM.zip › Source data/Figure 2/Figure 2f/HUMAN CD73 LOW CD73.tif]

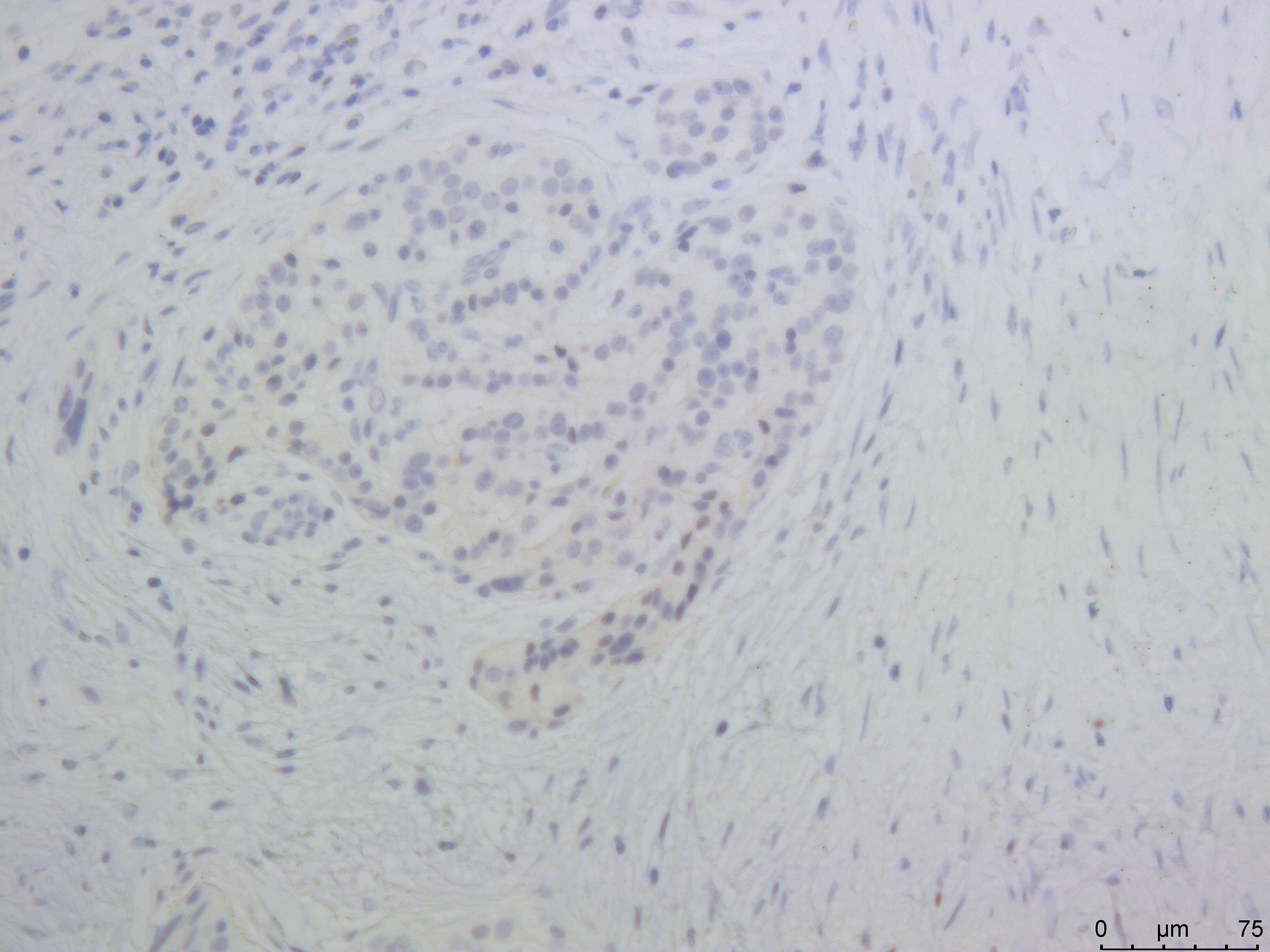

Supplement: Supplementary file 4 — Source data [file 41467_2023_38578_MOESM4_ESM.zip › Source data/Figure 2/Figure 2f/HUMAN CD73 LOW FOXP3.tif]

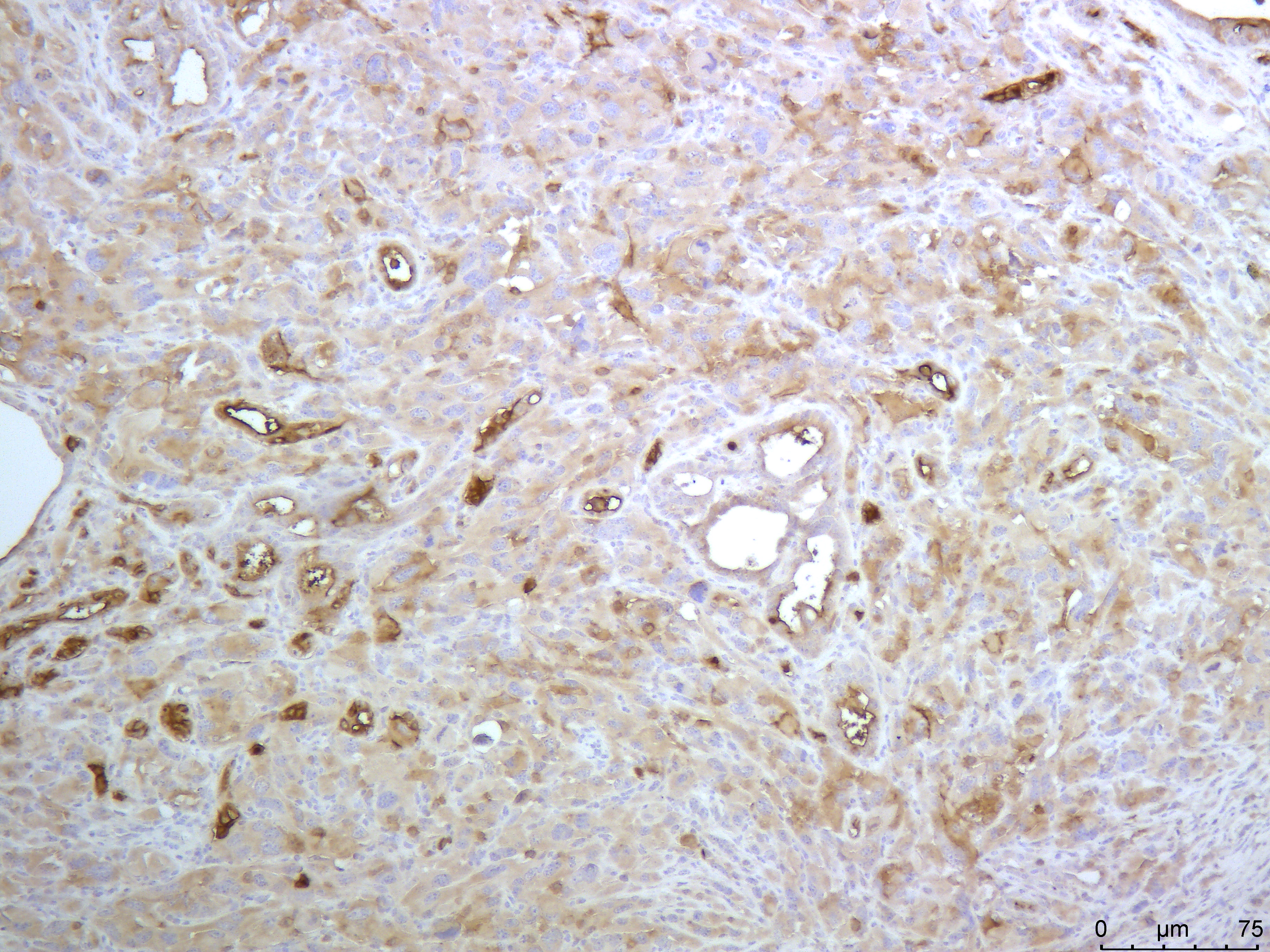

Supplement: Supplementary file 4 — Source data [file 41467_2023_38578_MOESM4_ESM.zip › Source data/Figure 2/Figure 2g/MOUSE CD73 HIGH CD73.tif]

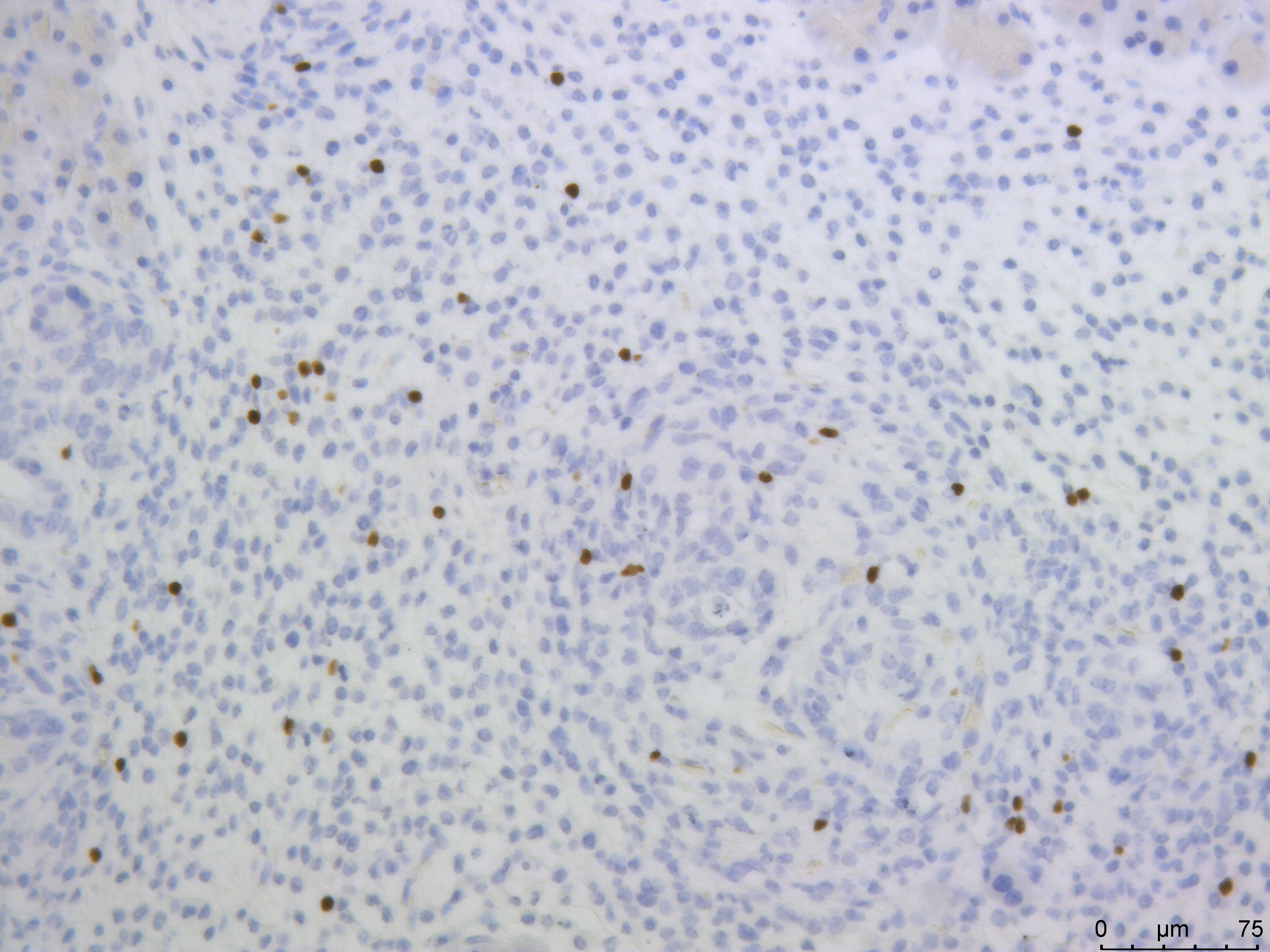

Supplement: Supplementary file 4 — Source data [file 41467_2023_38578_MOESM4_ESM.zip › Source data/Figure 2/Figure 2g/MOUSE CD73 HIGH FOXP3.tif]

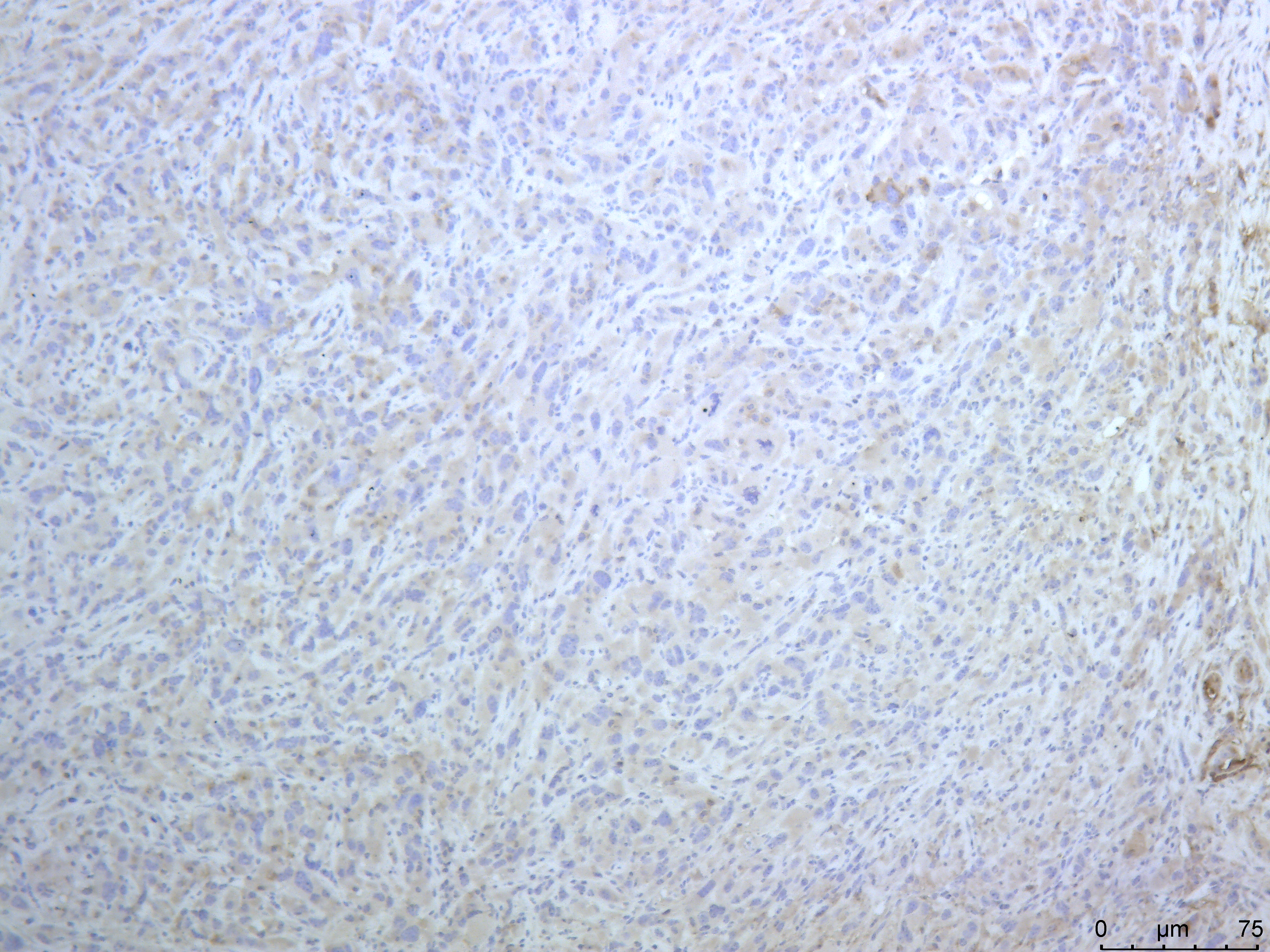

Supplement: Supplementary file 4 — Source data [file 41467_2023_38578_MOESM4_ESM.zip › Source data/Figure 2/Figure 2g/MOUSE CD73 LOW CD73.tif]

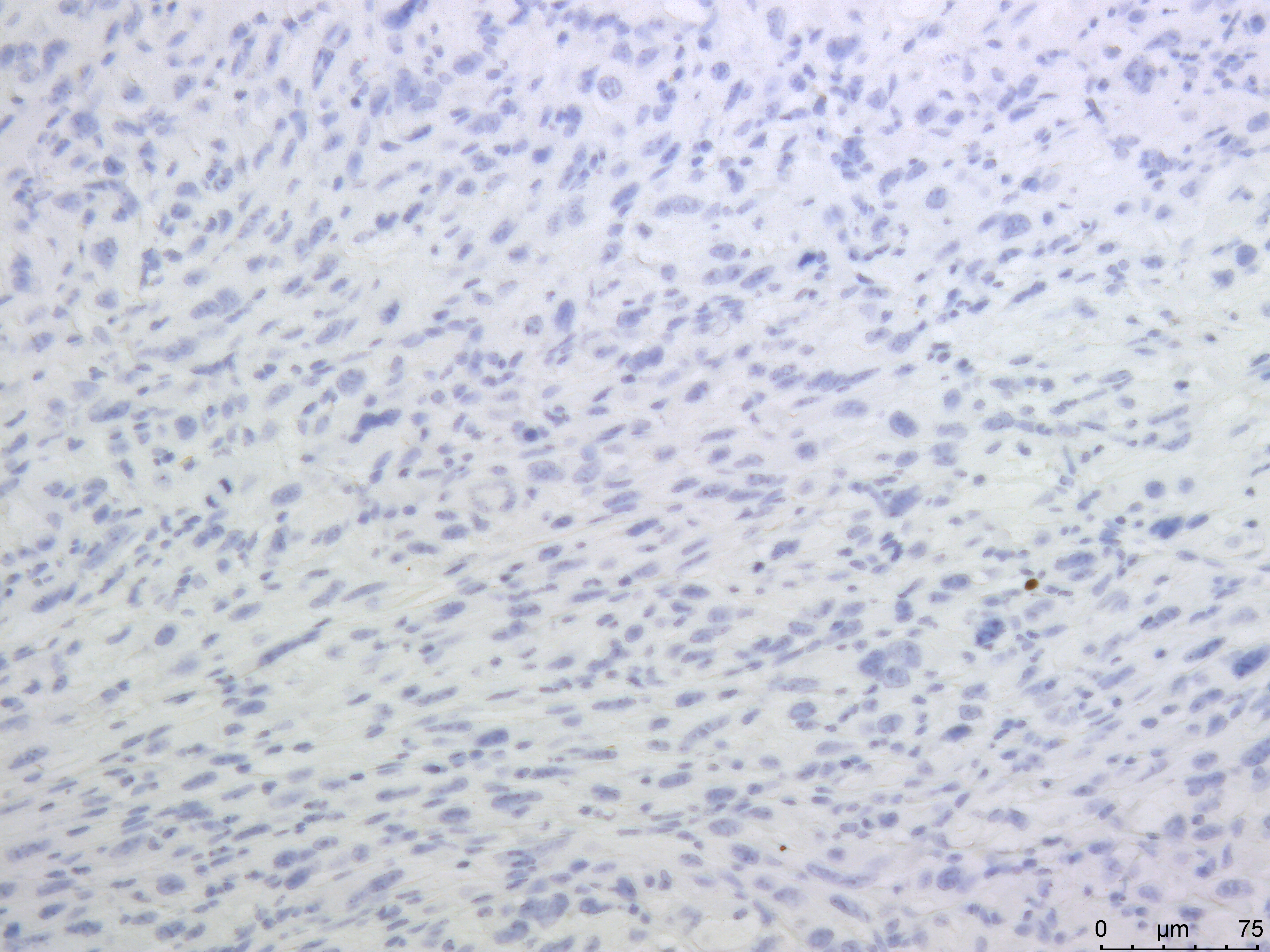

Supplement: Supplementary file 4 — Source data [file 41467_2023_38578_MOESM4_ESM.zip › Source data/Figure 2/Figure 2g/MOUSE CD73 LOW FOXP3.tif]

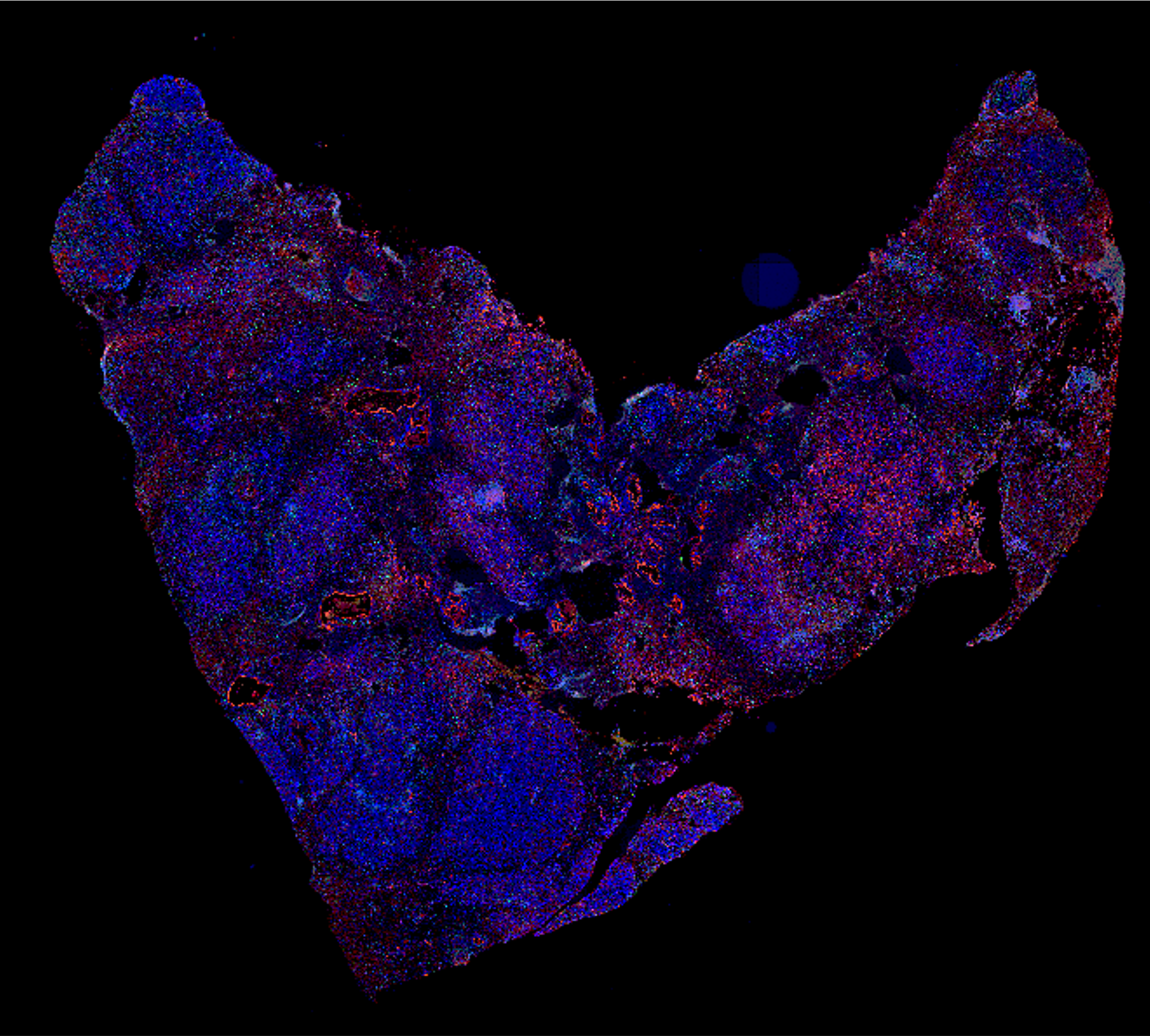

Supplement: Supplementary file 4 — Source data [file 41467_2023_38578_MOESM4_ESM.zip › Source data/Figure 2/Figure 2h/0.png]

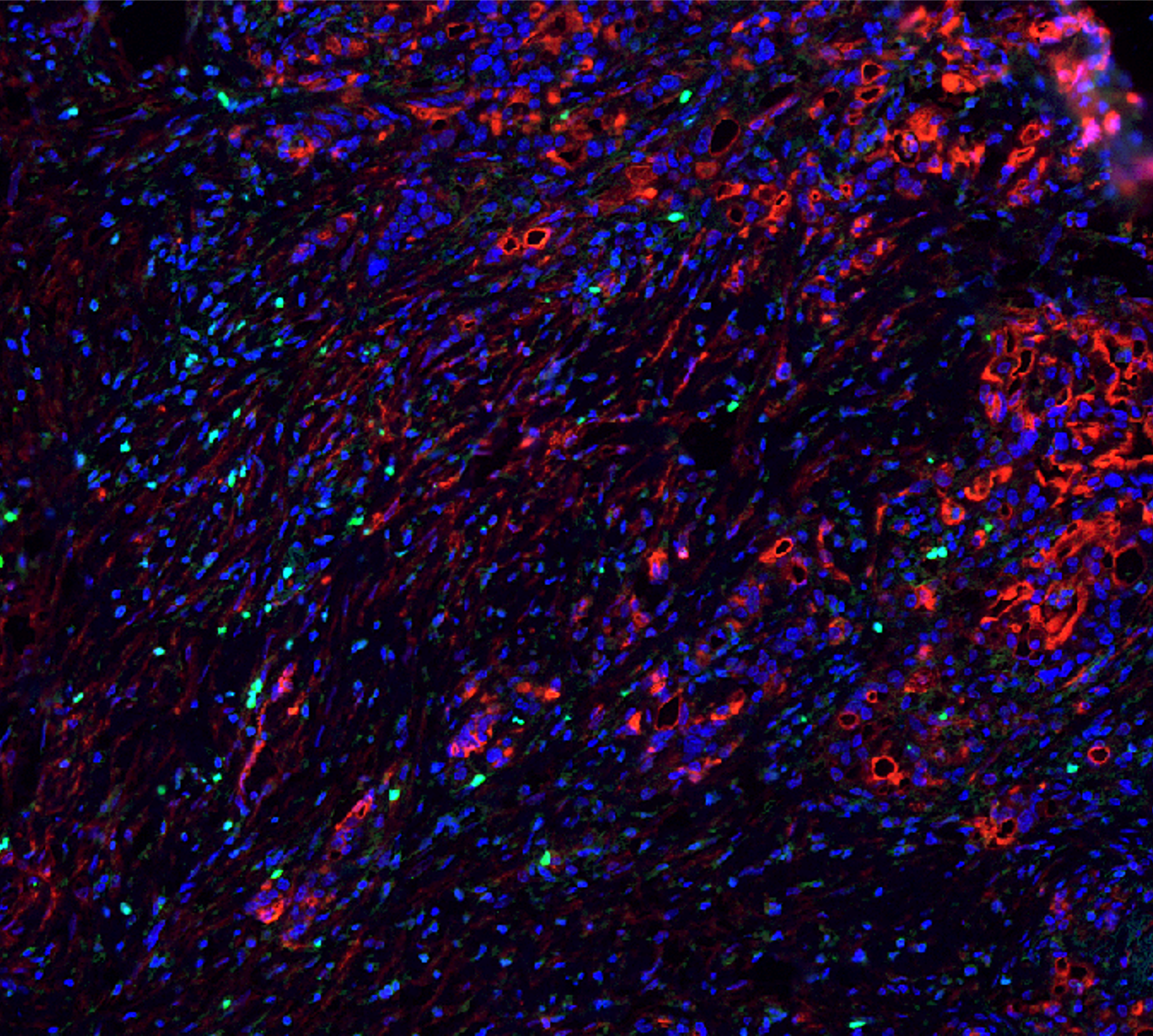

Supplement: Supplementary file 4 — Source data [file 41467_2023_38578_MOESM4_ESM.zip › Source data/Figure 2/Figure 2h/1.png]

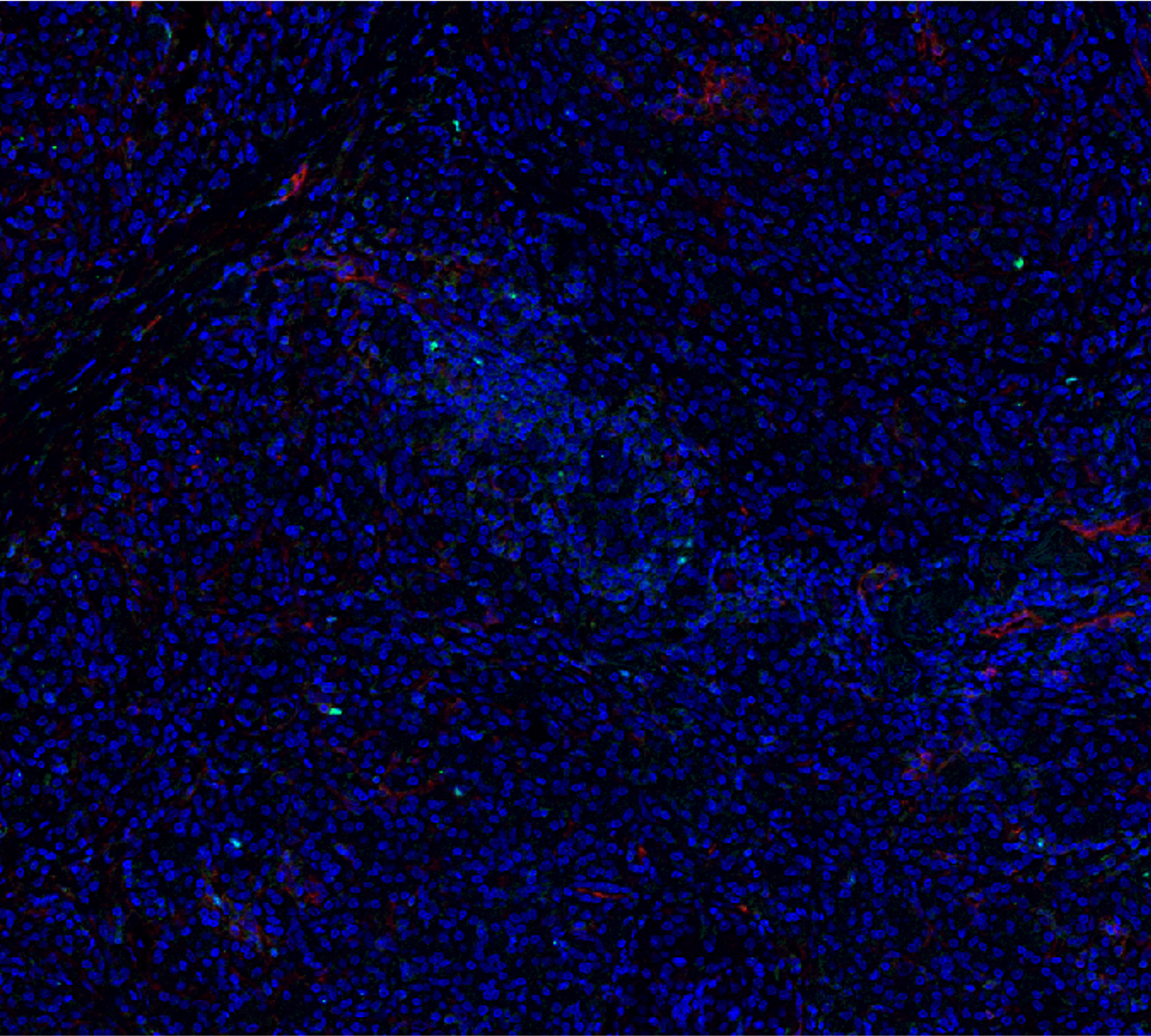

Supplement: Supplementary file 4 — Source data [file 41467_2023_38578_MOESM4_ESM.zip › Source data/Figure 2/Figure 2h/2.png]

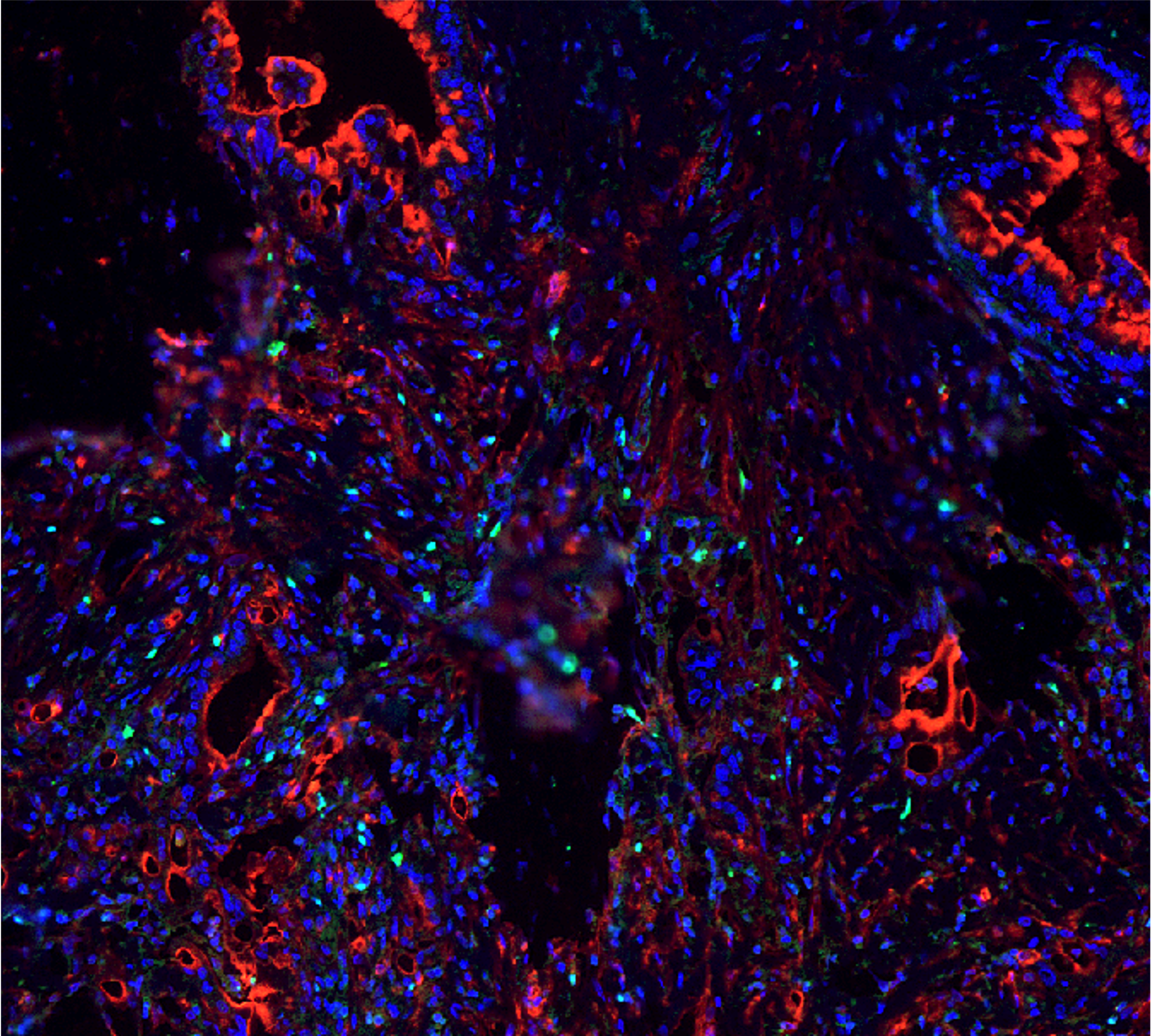

Supplement: Supplementary file 4 — Source data [file 41467_2023_38578_MOESM4_ESM.zip › Source data/Figure 2/Figure 2h/3.png]

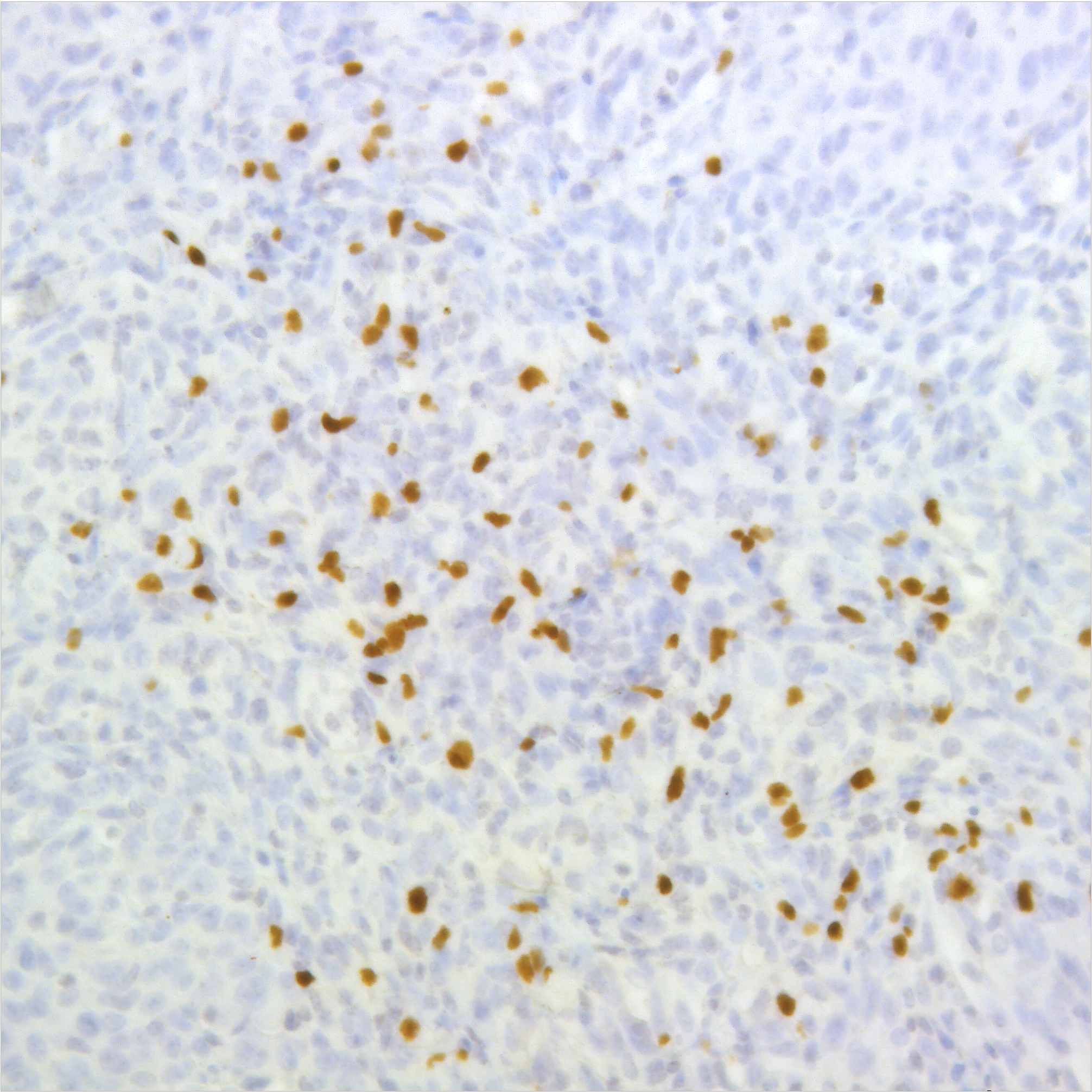

Supplement: Supplementary file 4 — Source data [file 41467_2023_38578_MOESM4_ESM.zip › Source data/Figure 3/Figure 3c/1.png]

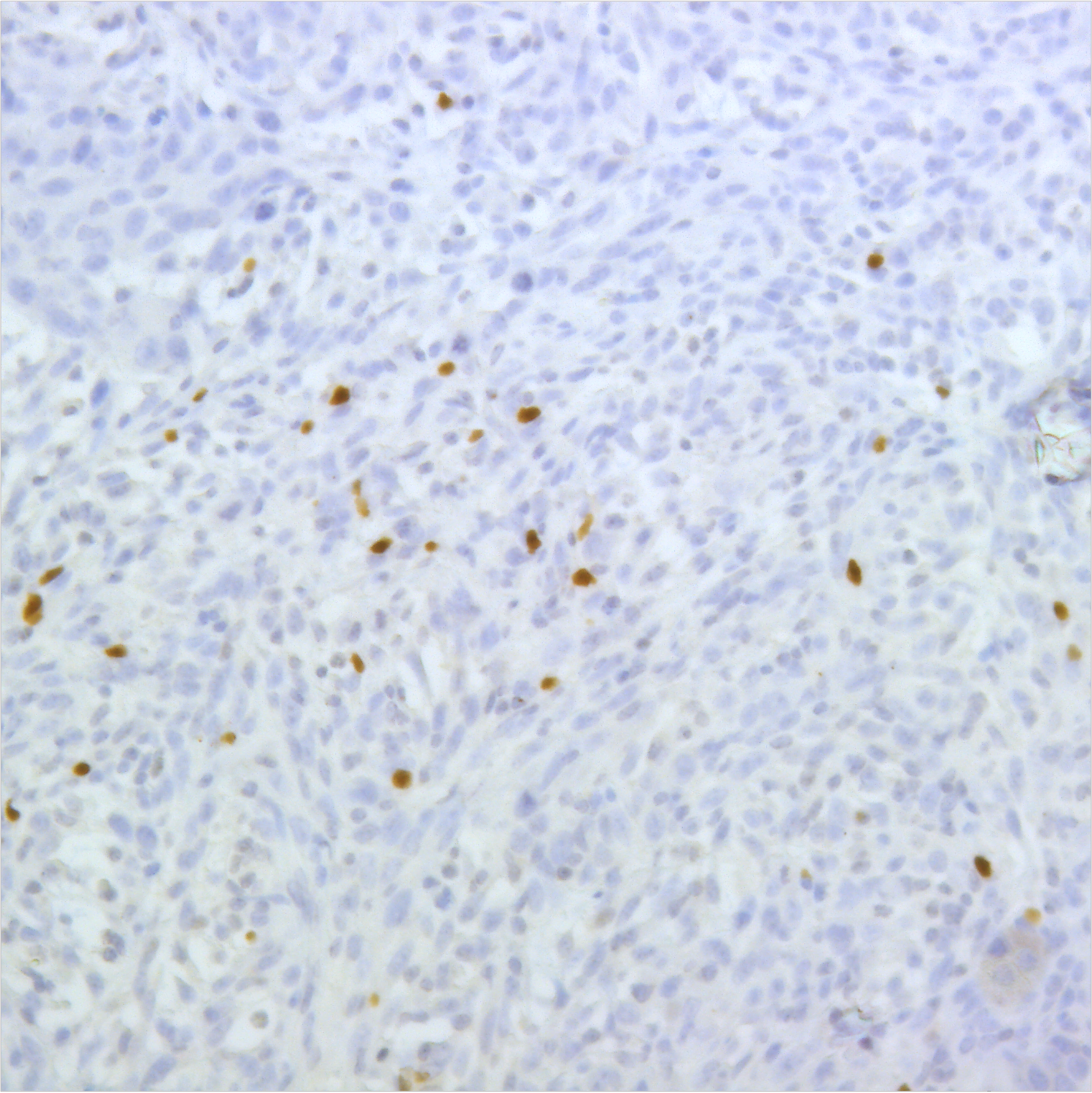

Supplement: Supplementary file 4 — Source data [file 41467_2023_38578_MOESM4_ESM.zip › Source data/Figure 3/Figure 3c/2.png]

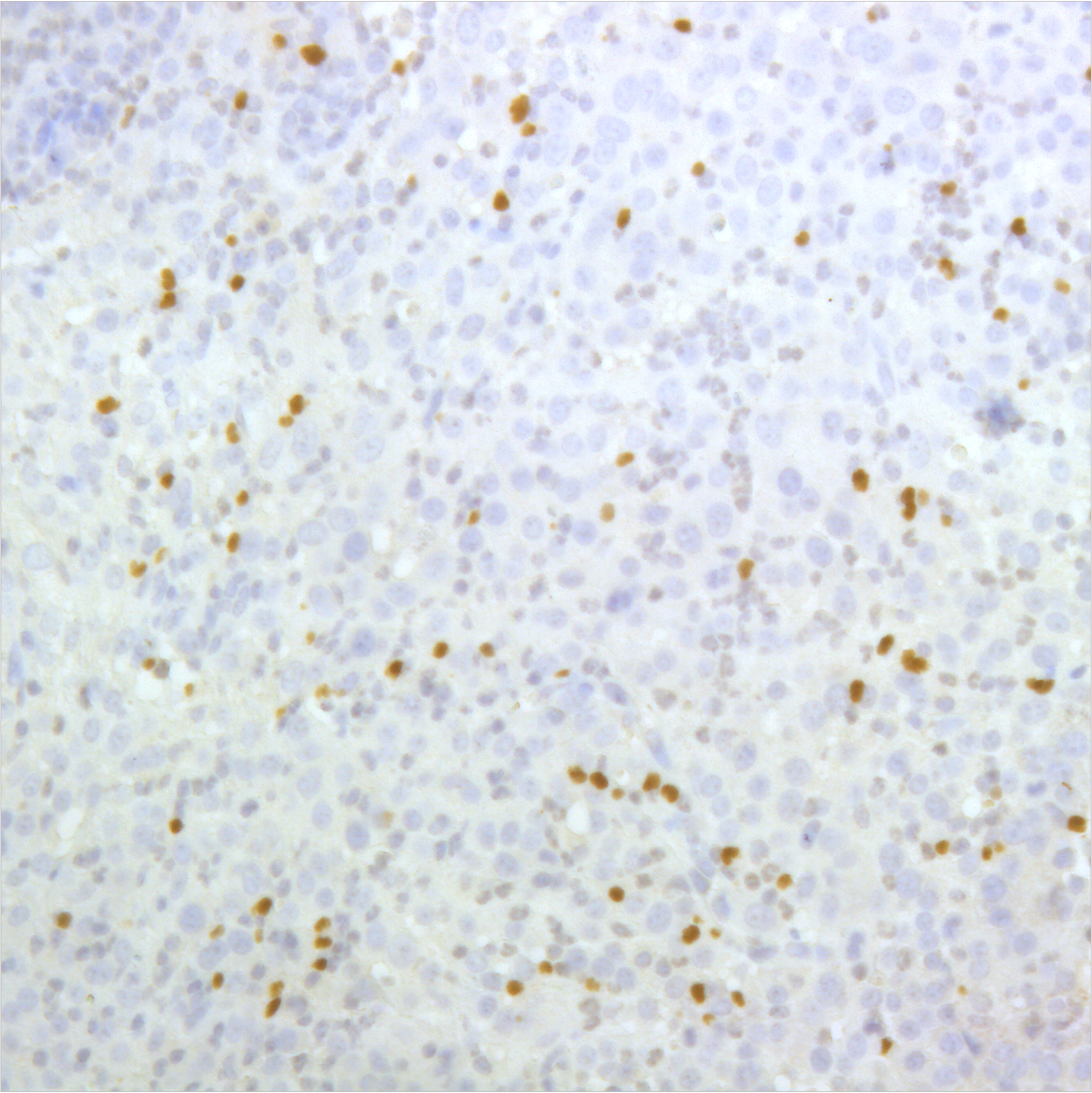

Supplement: Supplementary file 4 — Source data [file 41467_2023_38578_MOESM4_ESM.zip › Source data/Figure 3/Figure 3f/1.png]

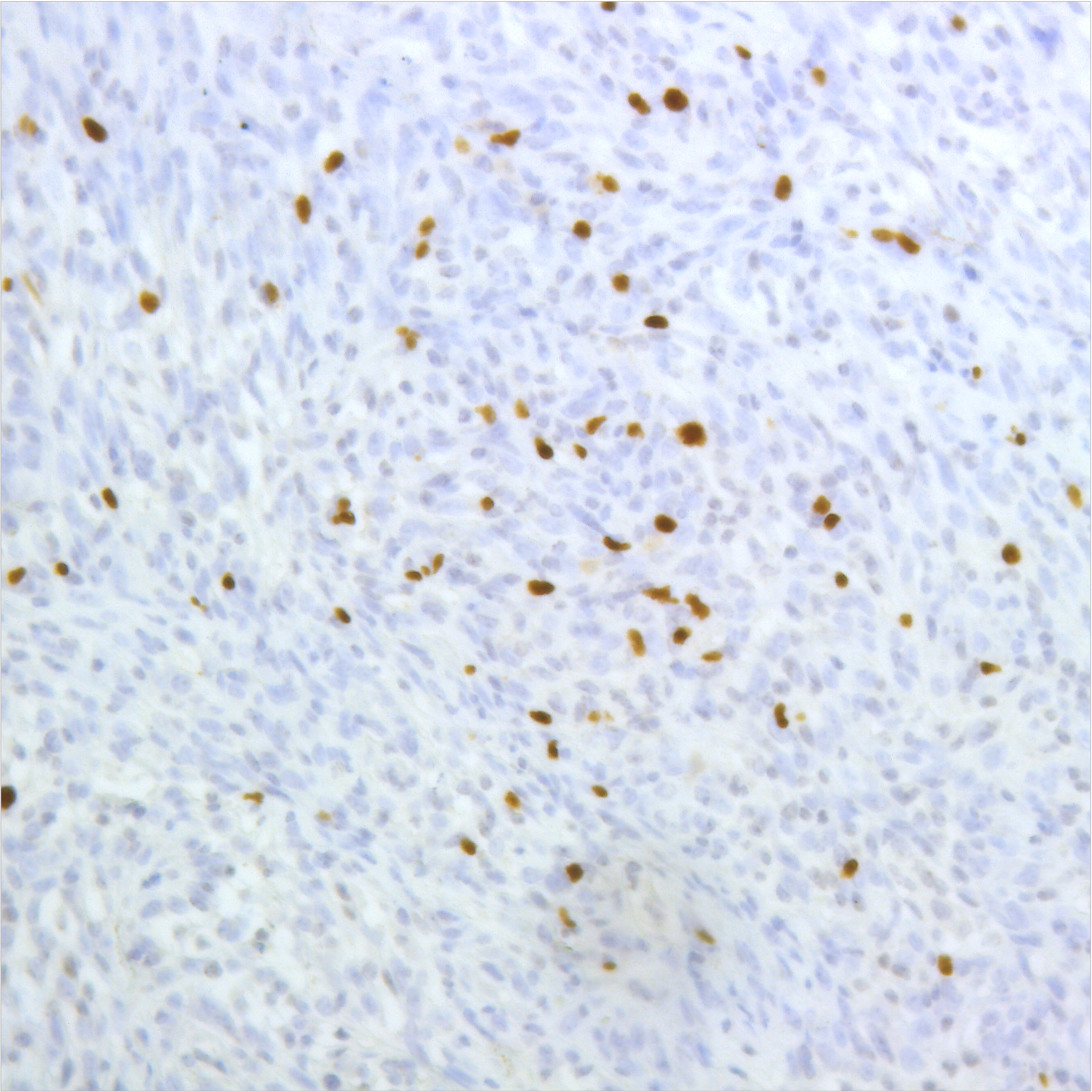

Supplement: Supplementary file 4 — Source data [file 41467_2023_38578_MOESM4_ESM.zip › Source data/Figure 3/Figure 3f/2.png]

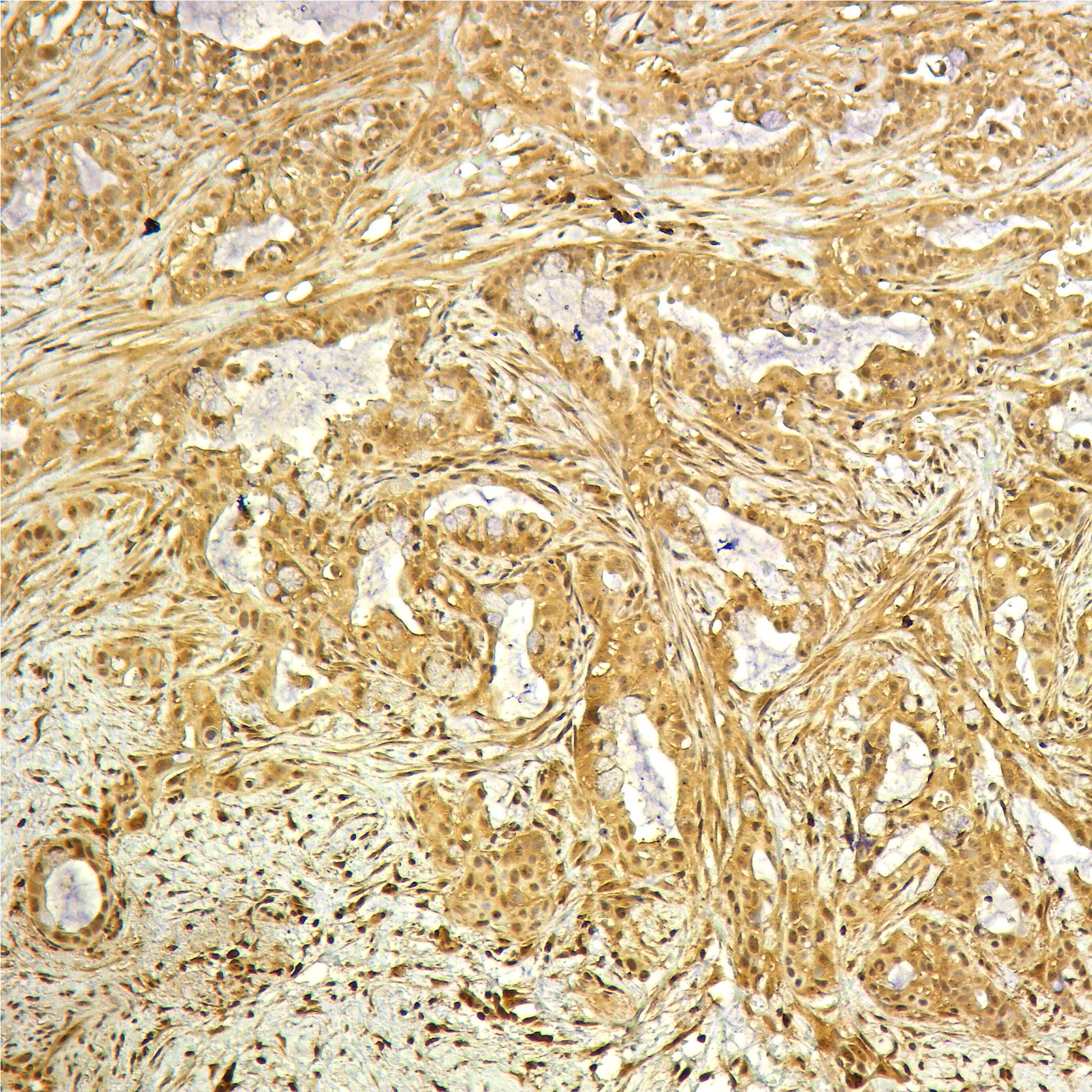

Supplement: Supplementary file 4 — Source data [file 41467_2023_38578_MOESM4_ESM.zip › Source data/Figure 4/Figure 4l/1.png]

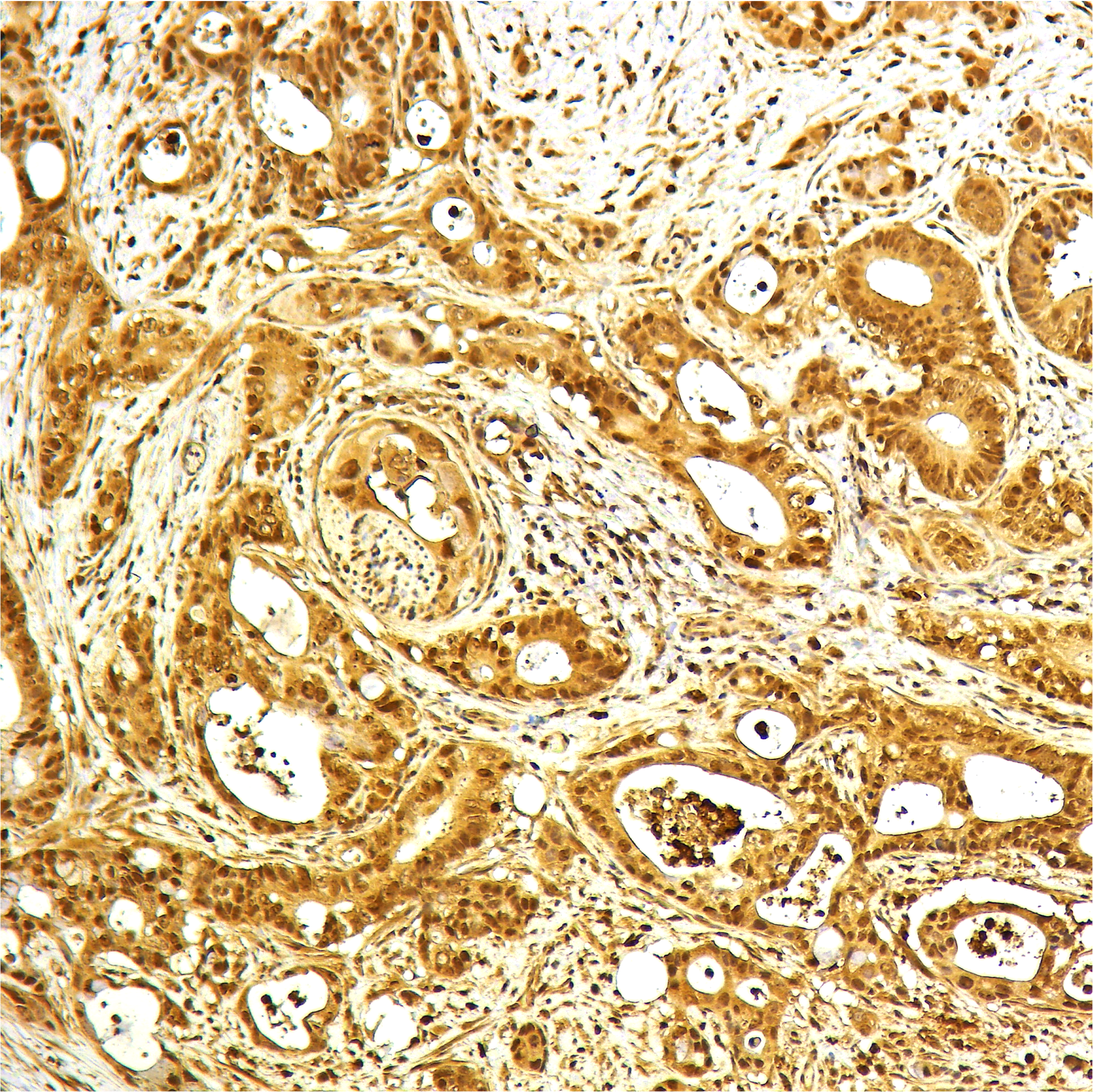

Supplement: Supplementary file 4 — Source data [file 41467_2023_38578_MOESM4_ESM.zip › Source data/Figure 4/Figure 4l/2.png]

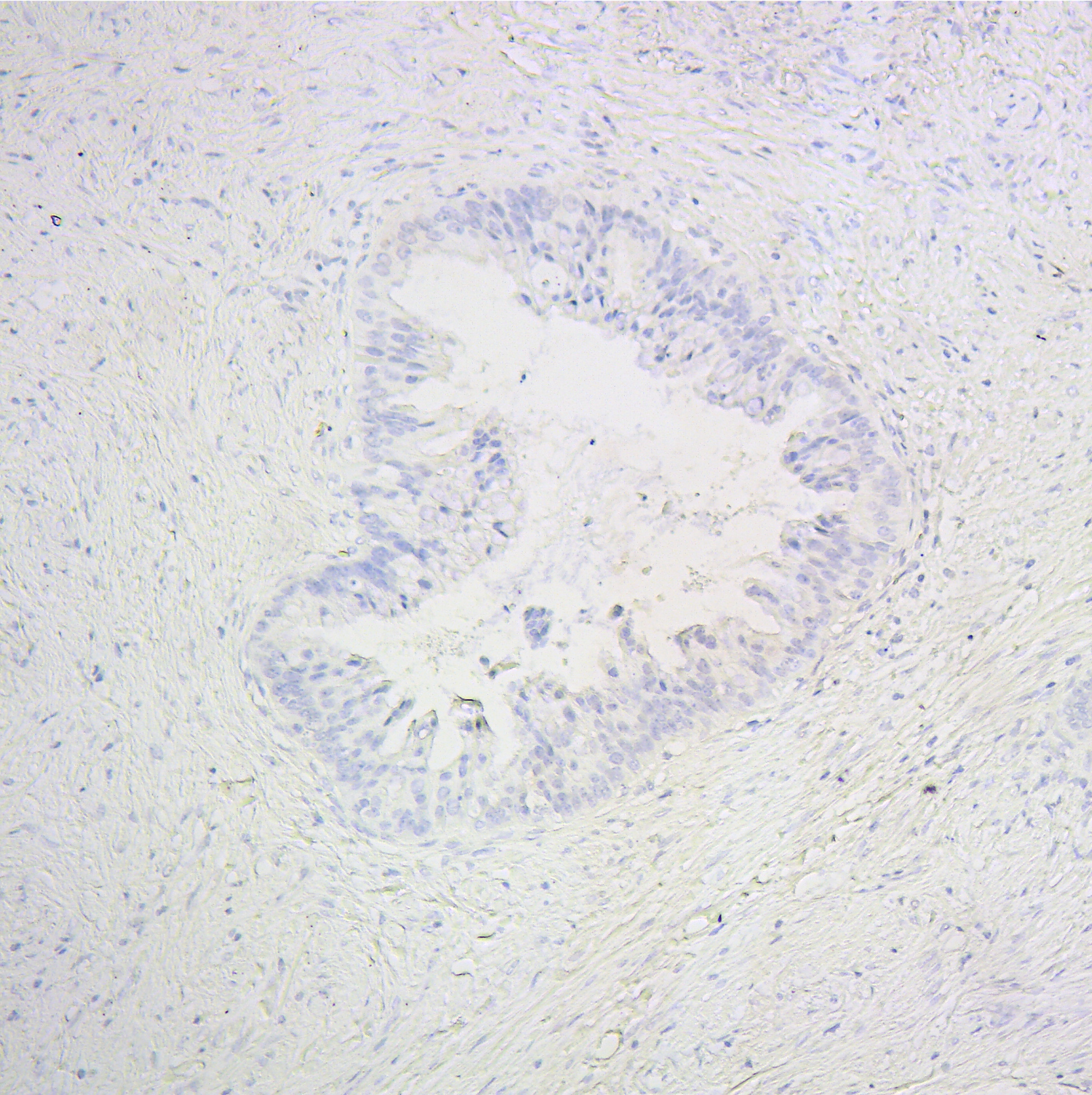

Supplement: Supplementary file 4 — Source data [file 41467_2023_38578_MOESM4_ESM.zip › Source data/Figure 4/Figure 4l/3.png]

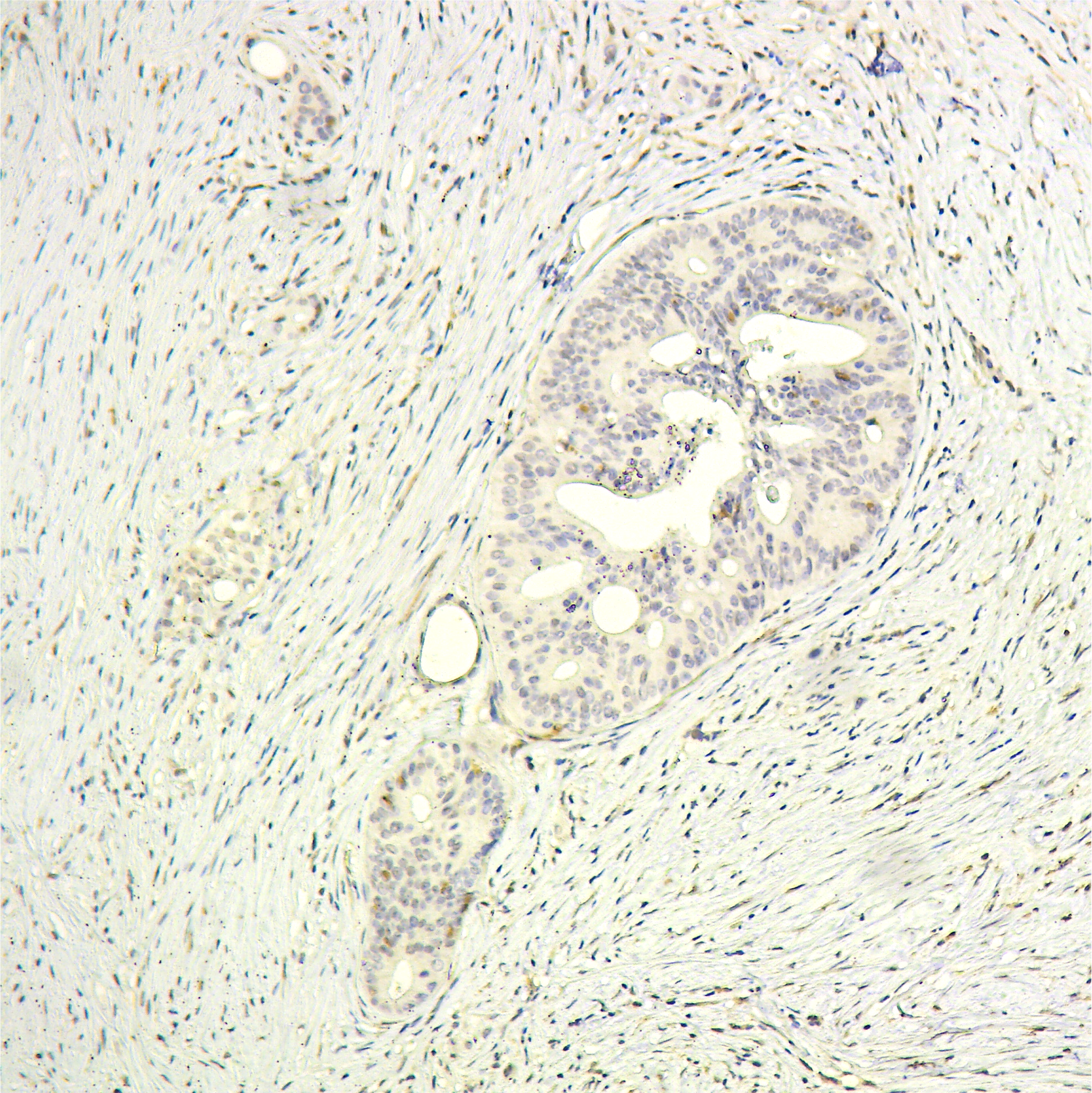

Supplement: Supplementary file 4 — Source data [file 41467_2023_38578_MOESM4_ESM.zip › Source data/Figure 4/Figure 4l/4.png]

Figure 5c

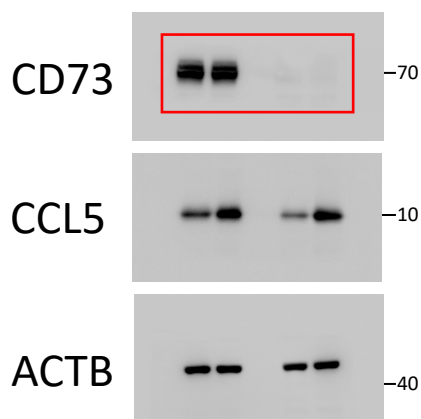

Figure 5d

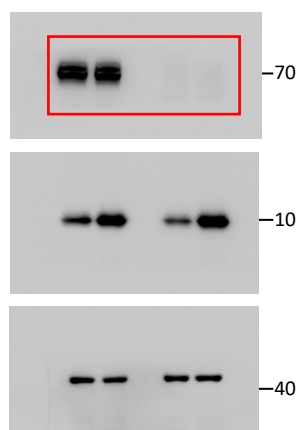

Figure 5e

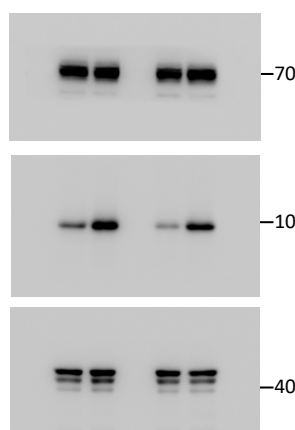

Figure 5f

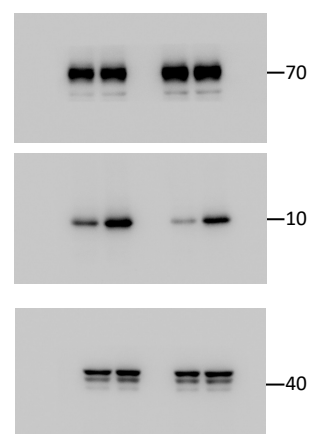

Figure 5g

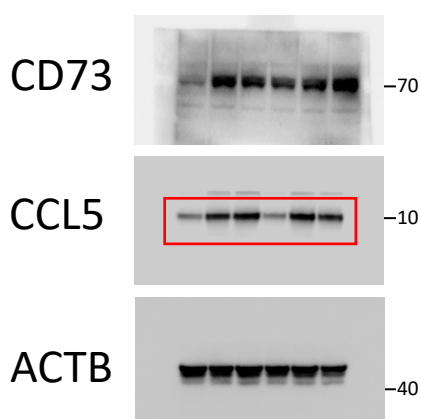

Figure 5h

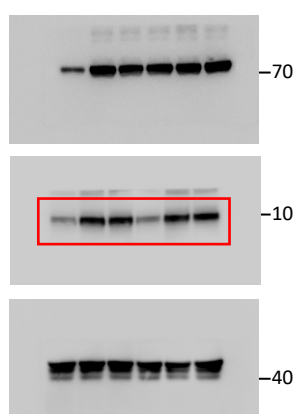

Figure 5i

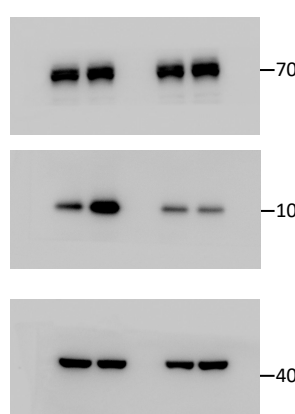

Figure 5k

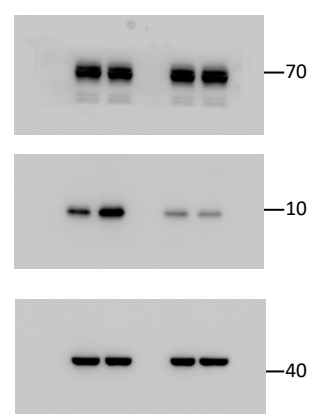

Supplement: Supplementary file 4 — Source data [file 41467_2023_38578_MOESM4_ESM.zip › Source data/Figure 5/Figure 5-uncropped gels.pdf]

Figure 6d

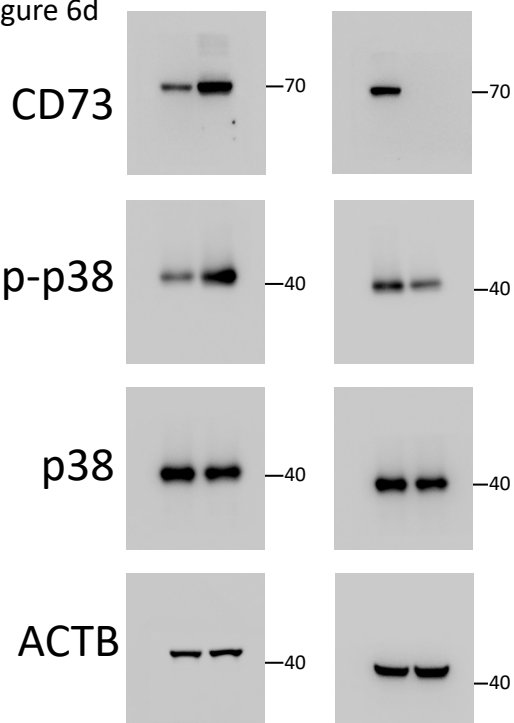

Figure 6e

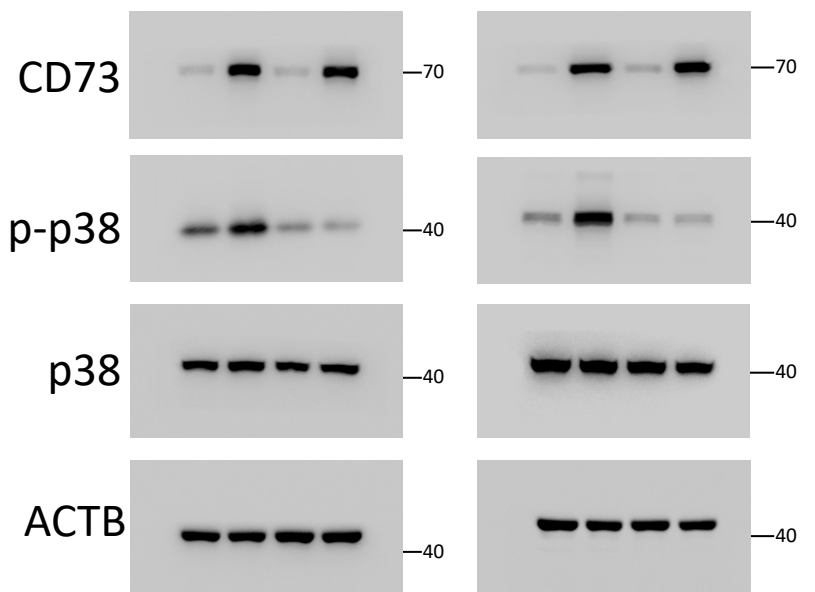

Figure 6f

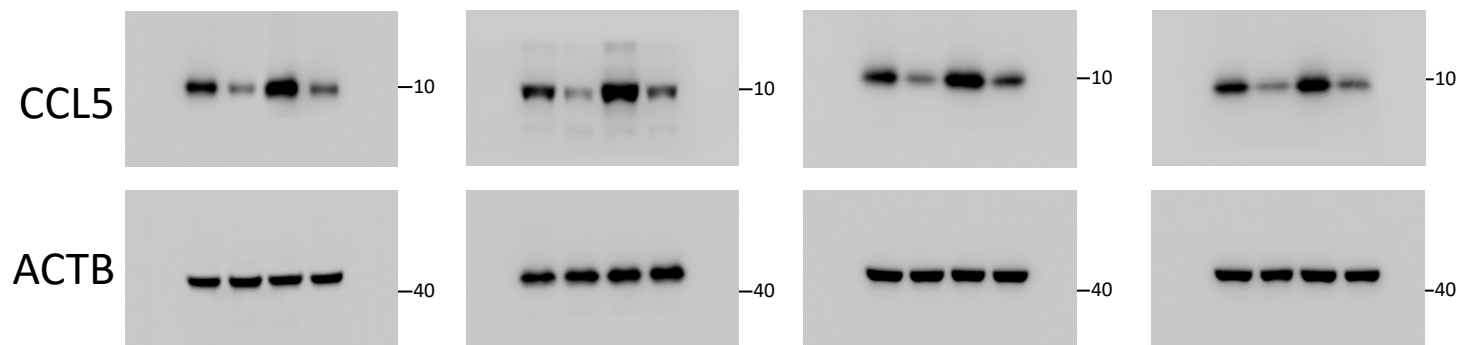

Figure 6h

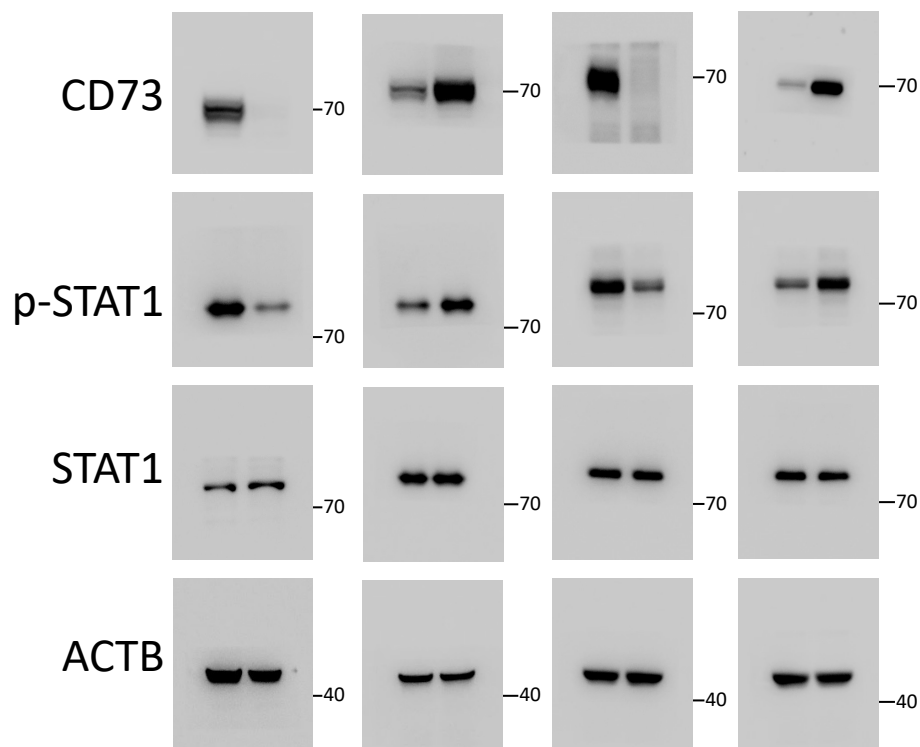

Figure 6i

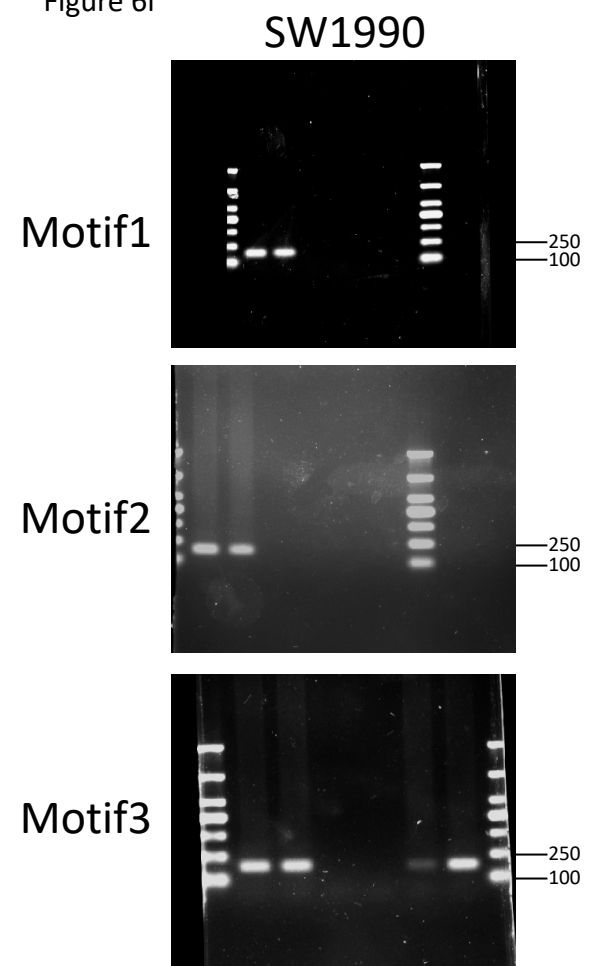

SW1990

Figure 6i

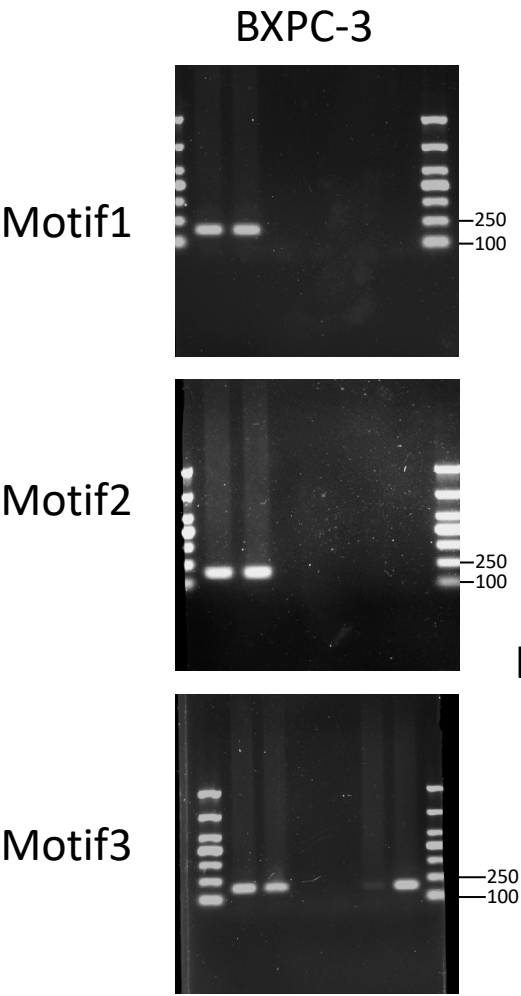

Figure 6j

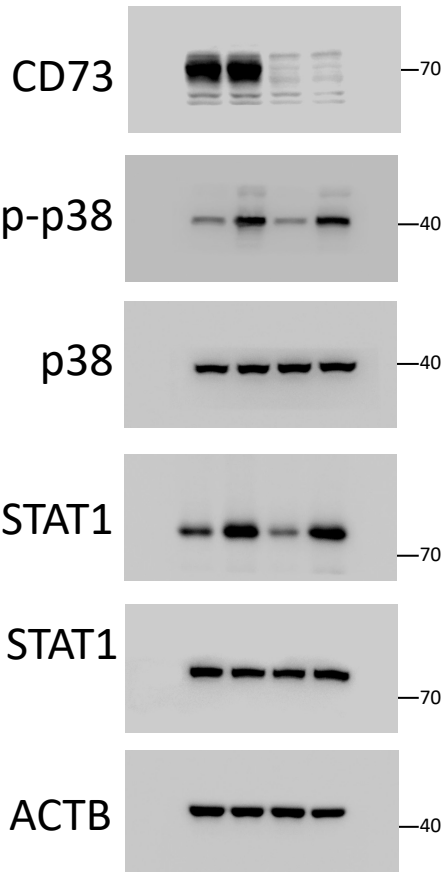

Figure 6k

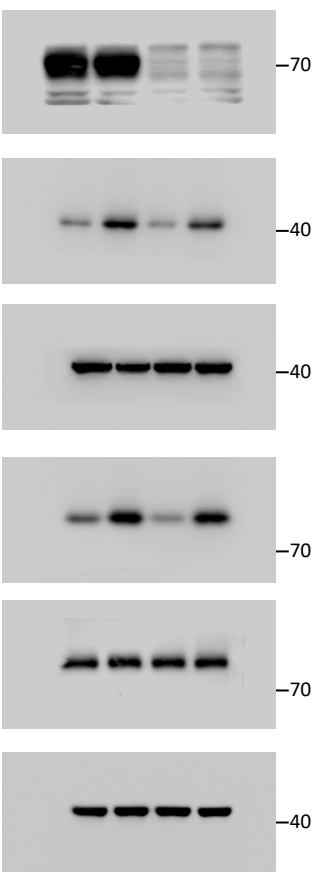

Figure 6i

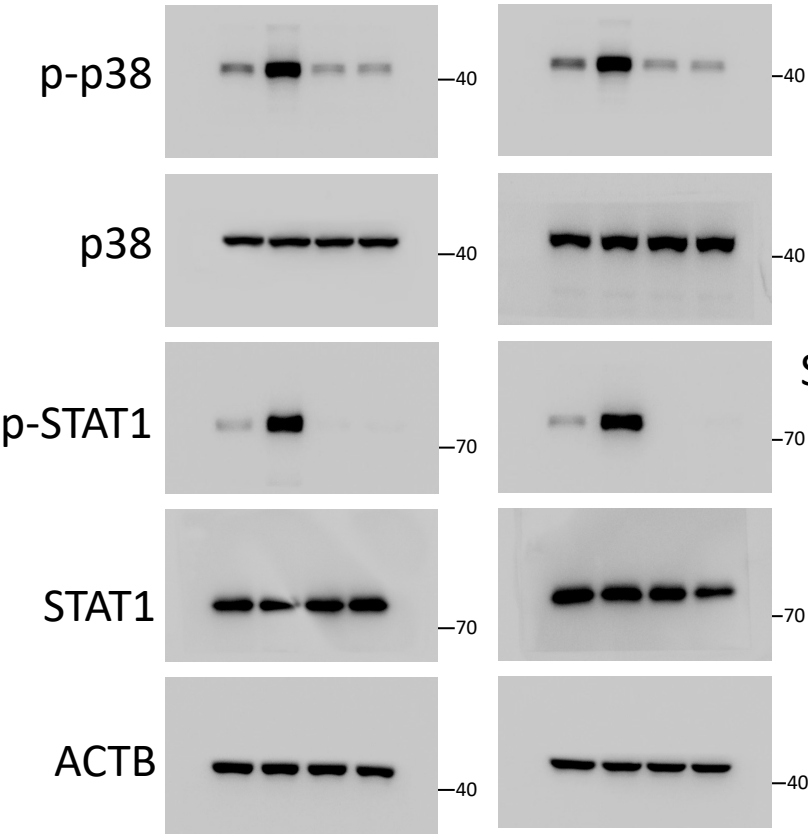

Figure 6m

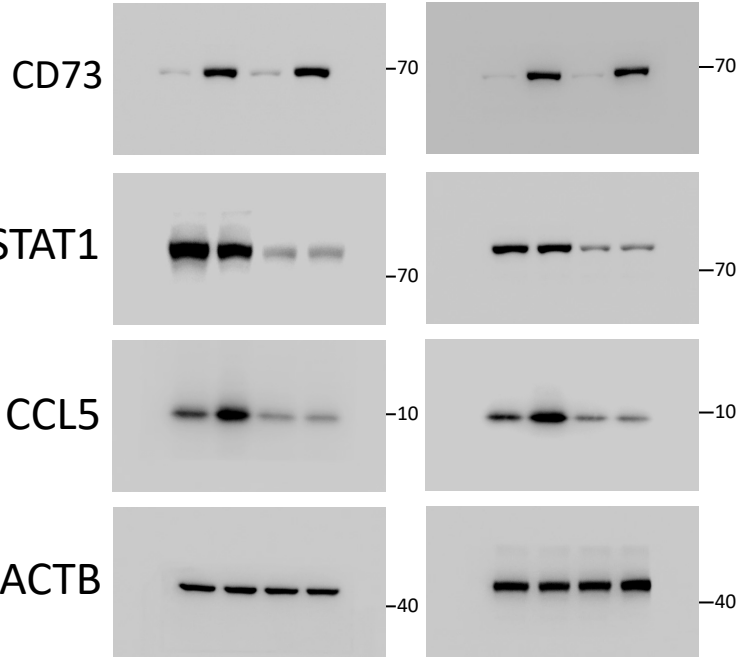

Supplement: Supplementary file 4 — Source data [file 41467_2023_38578_MOESM4_ESM.zip › Source data/Figure 6/Figure 6-uncropped gels.pdf]

Supplementary Figure 10a

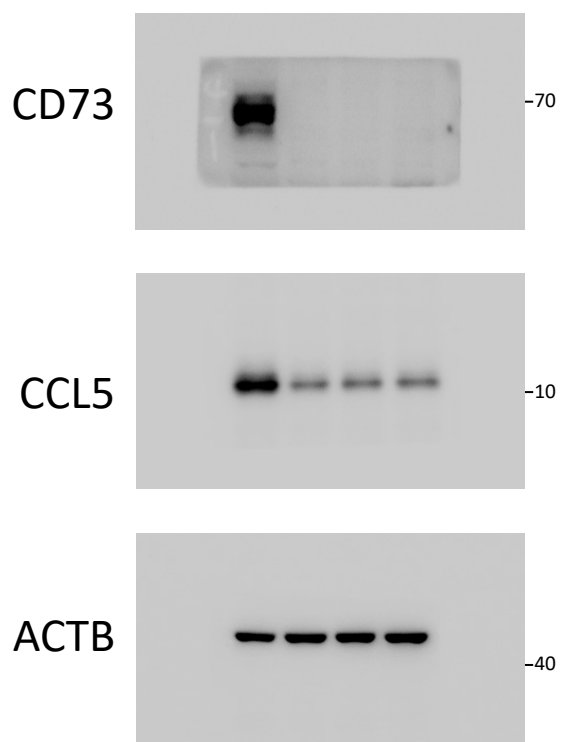

Supplementary Figure 10b

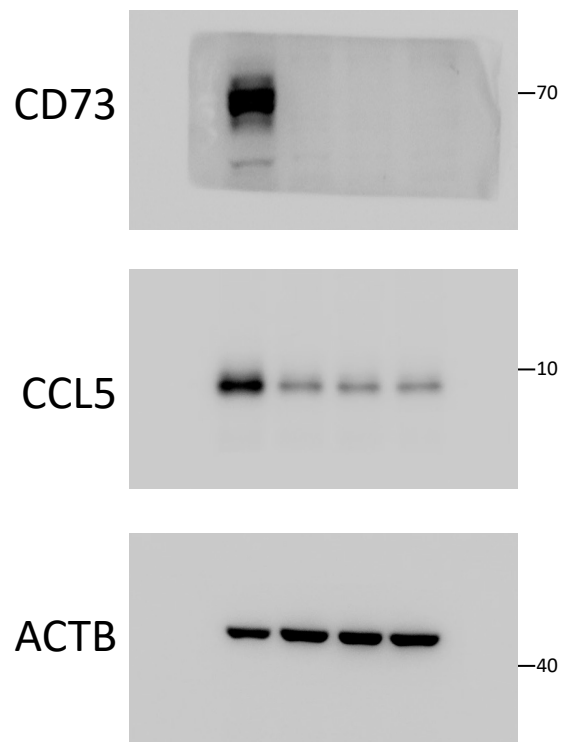

Supplement: Supplementary file 4 — Source data [file 41467_2023_38578_MOESM4_ESM.zip › Source data/Supplementary Figure 10/Supplementary Figure 10-uncropped gels.pdf]

Supplementary Figure 12c

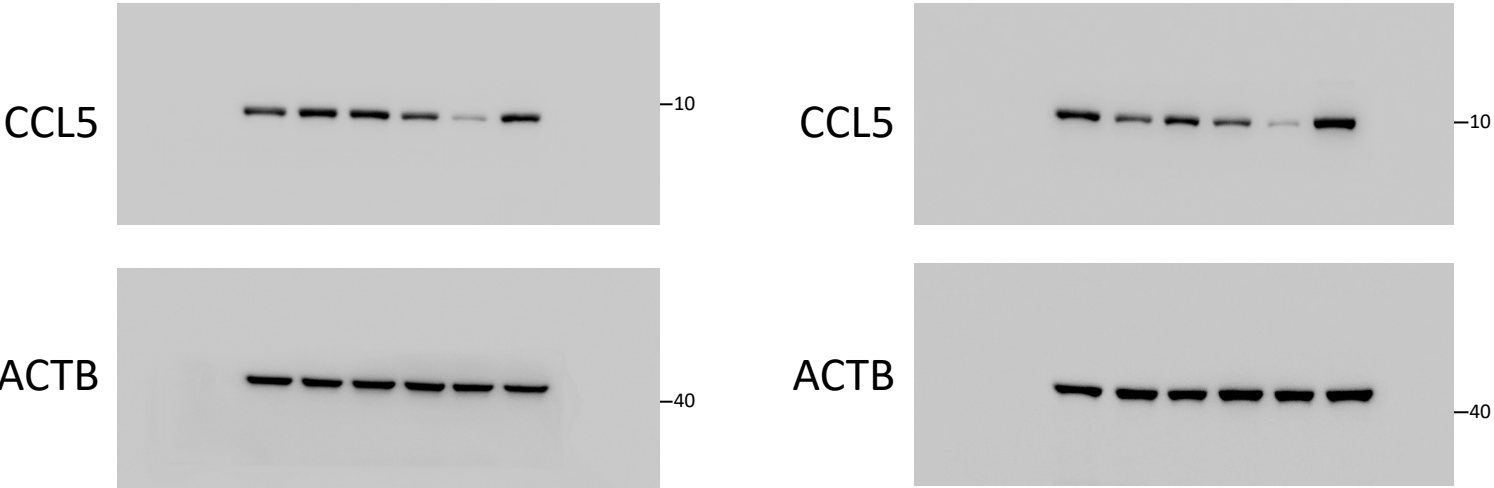

Supplementary Figure 12d

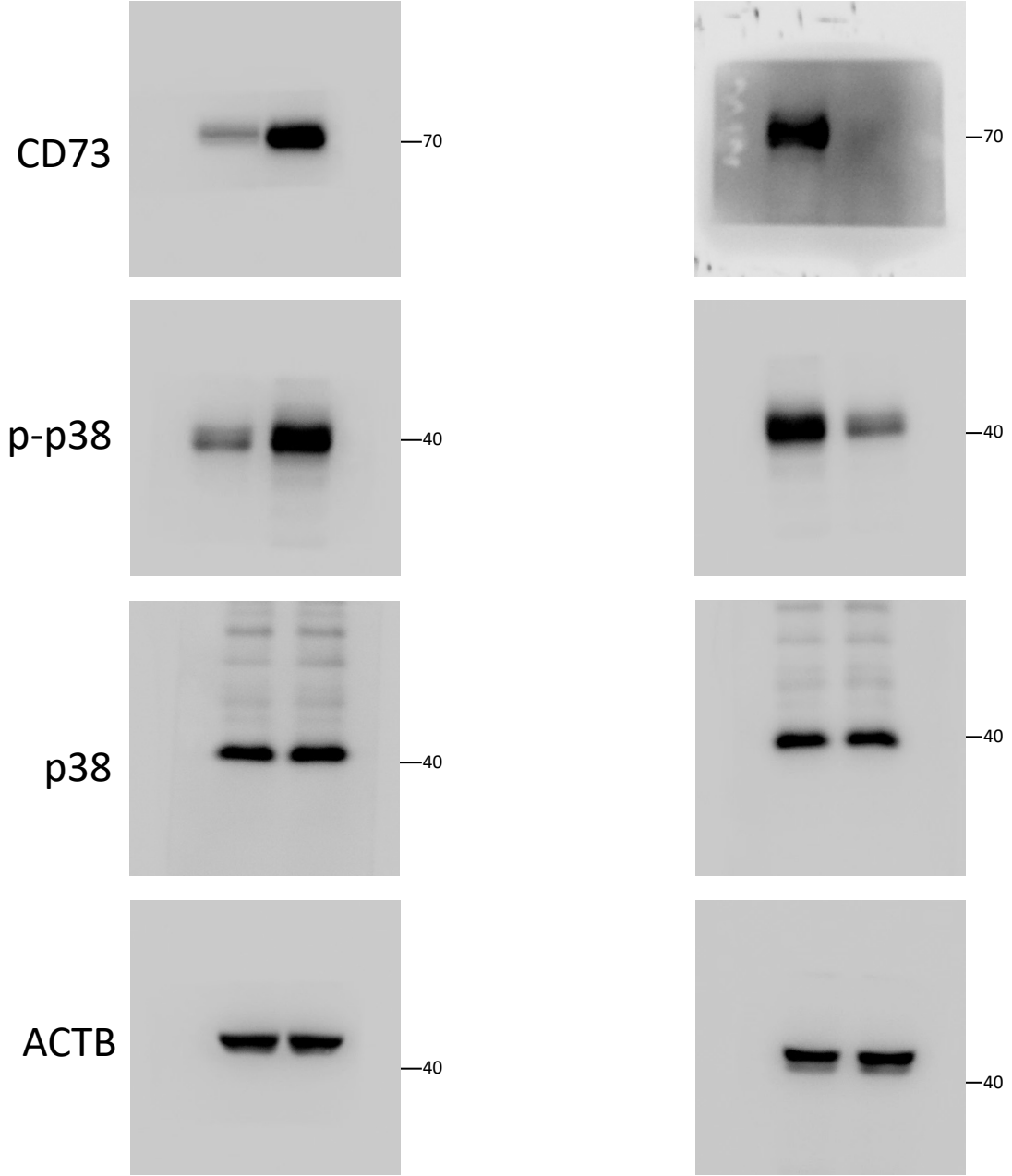

Supplement: Supplementary file 4 — Source data [file 41467_2023_38578_MOESM4_ESM.zip › Source data/Supplementary Figure 12/Supplementary Figure 12-uncropped gels.pdf]

Supplementary Figure 6a

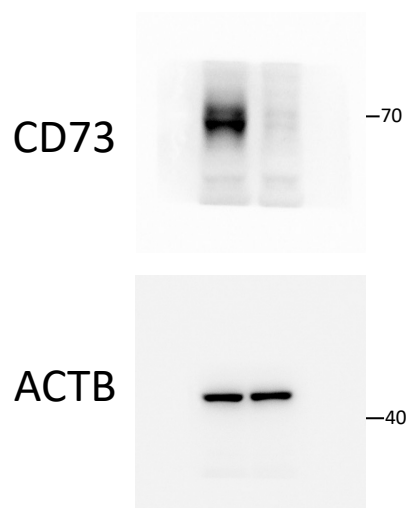

Supplementary Figure 6c

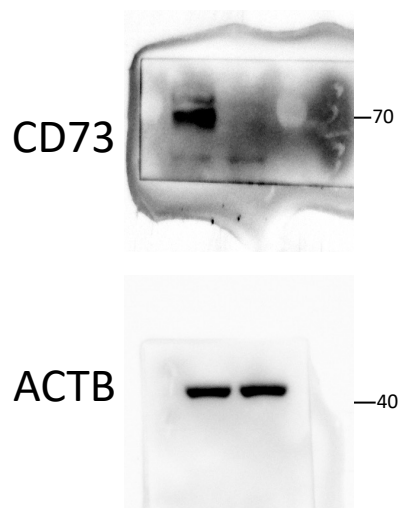

Supplement: Supplementary file 4 — Source data [file 41467_2023_38578_MOESM4_ESM.zip › Source data/Supplementary Figure 6/Supplementary Figure 6-uncropped gels.pdf]

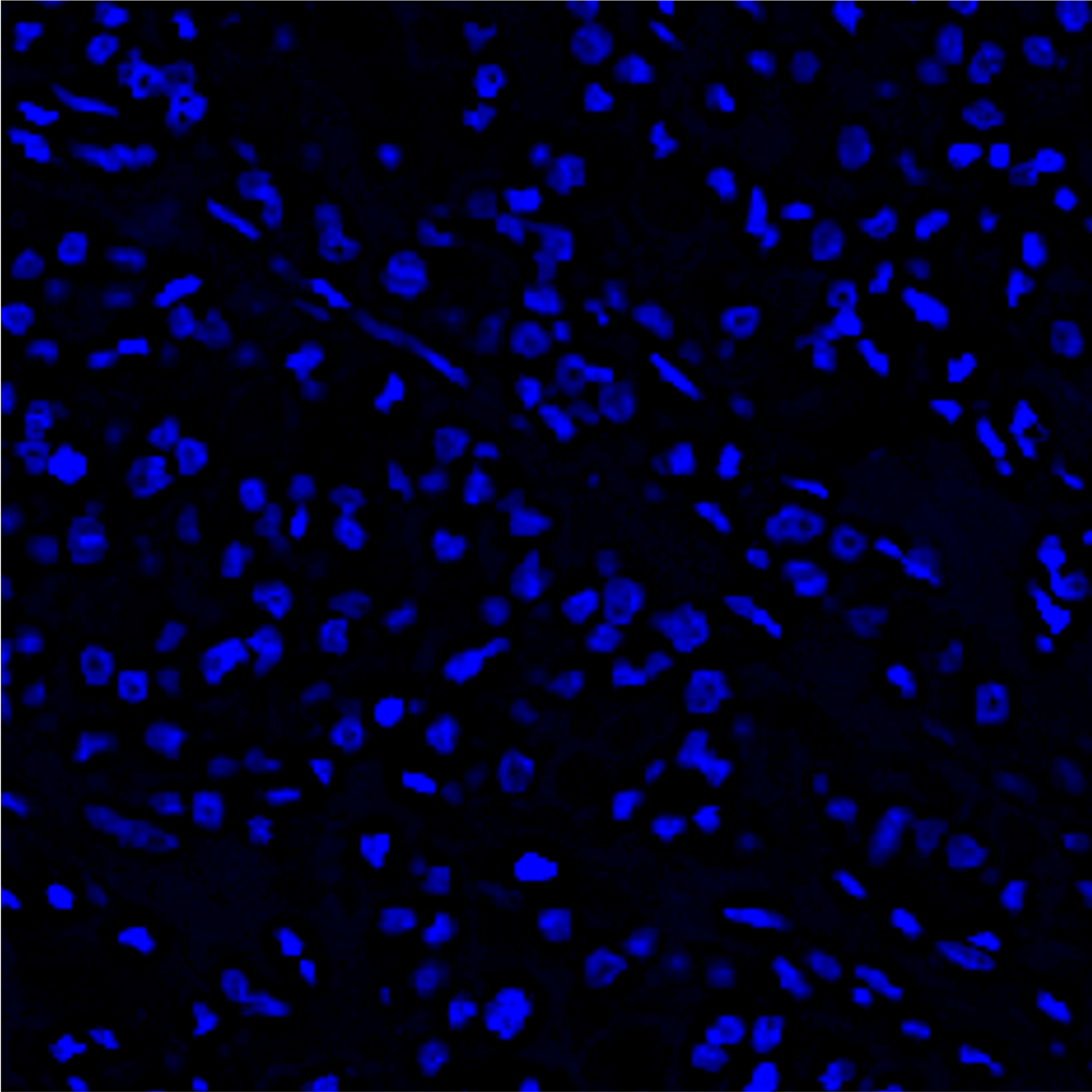

Supplement: Supplementary file 4 — Source data [file 41467_2023_38578_MOESM4_ESM.zip › Source data/Supplementary Figure 6/Supplementary Figure 6b/CD73 KO/1.png]

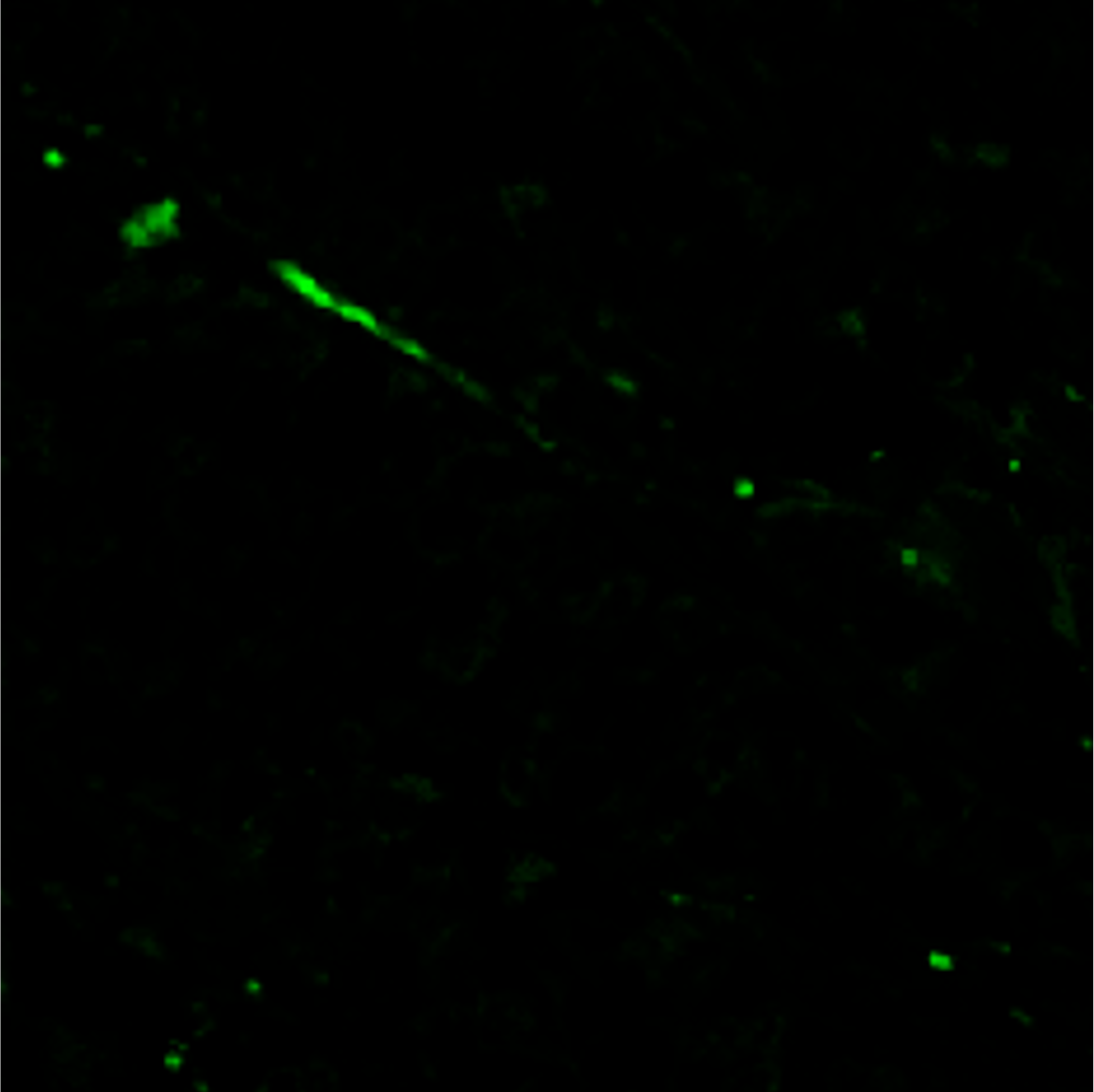

Supplement: Supplementary file 4 — Source data [file 41467_2023_38578_MOESM4_ESM.zip › Source data/Supplementary Figure 6/Supplementary Figure 6b/CD73 KO/2.png]

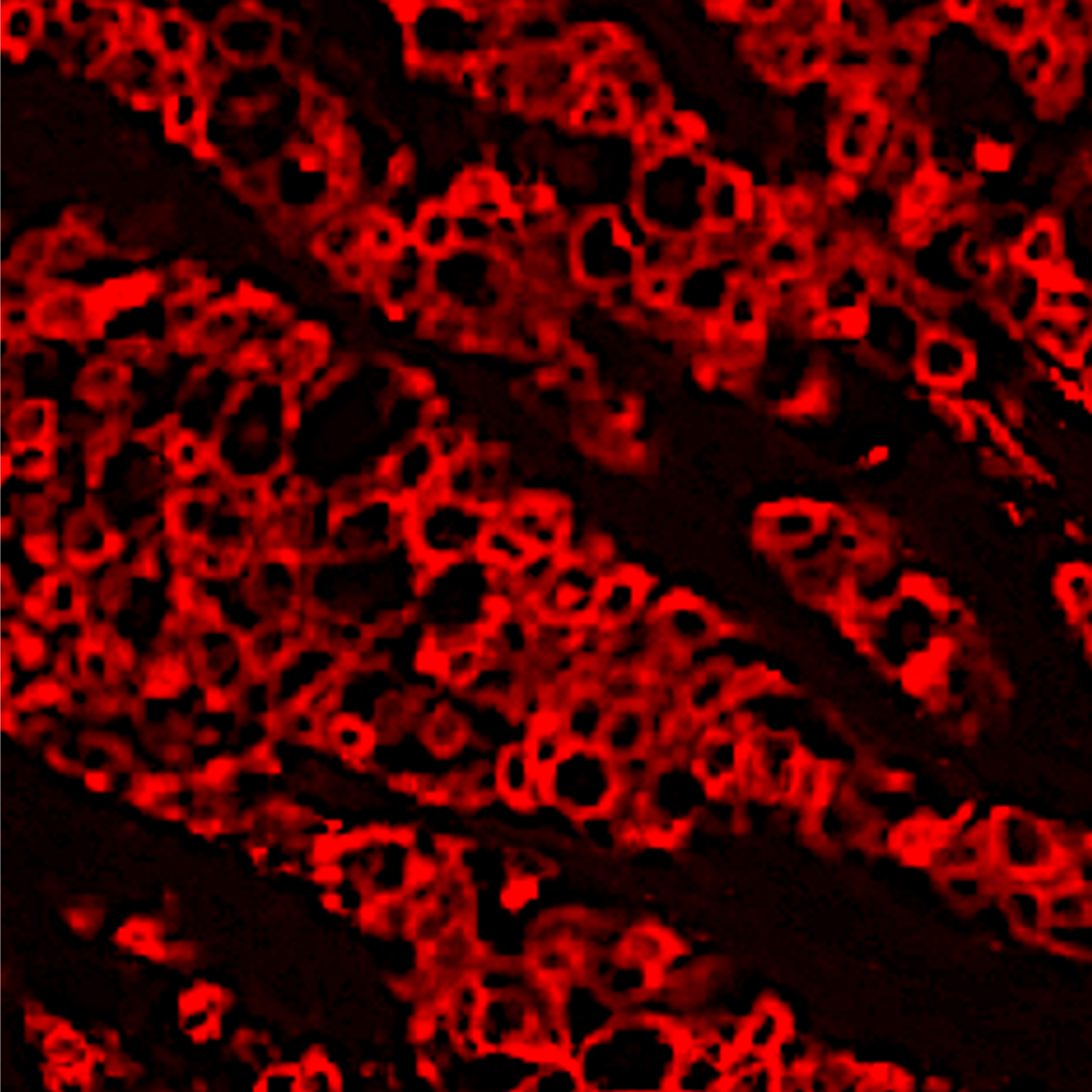

Supplement: Supplementary file 4 — Source data [file 41467_2023_38578_MOESM4_ESM.zip › Source data/Supplementary Figure 6/Supplementary Figure 6b/CD73 KO/3.png]

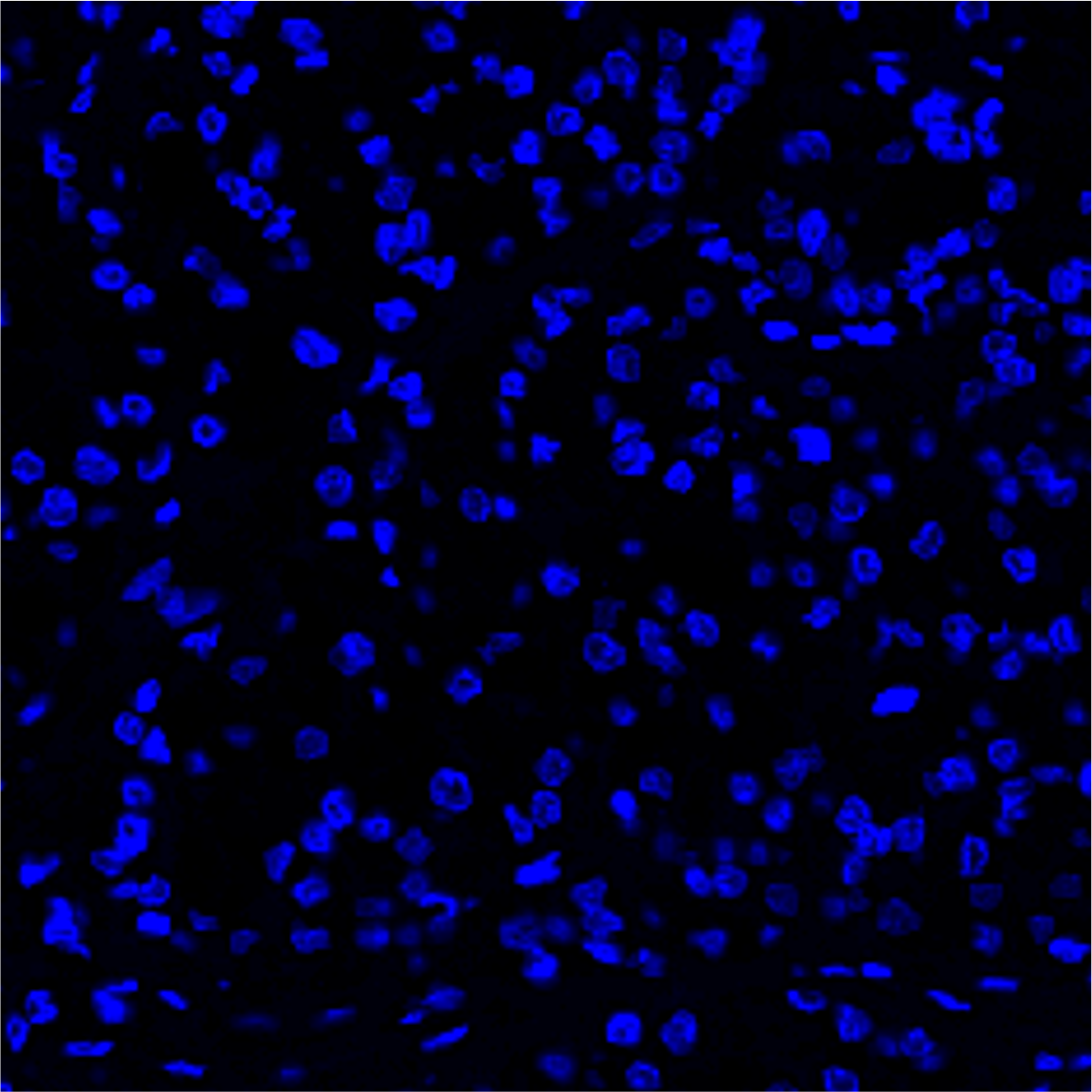

Supplement: Supplementary file 4 — Source data [file 41467_2023_38578_MOESM4_ESM.zip › Source data/Supplementary Figure 6/Supplementary Figure 6b/CD73 OE/1.png]

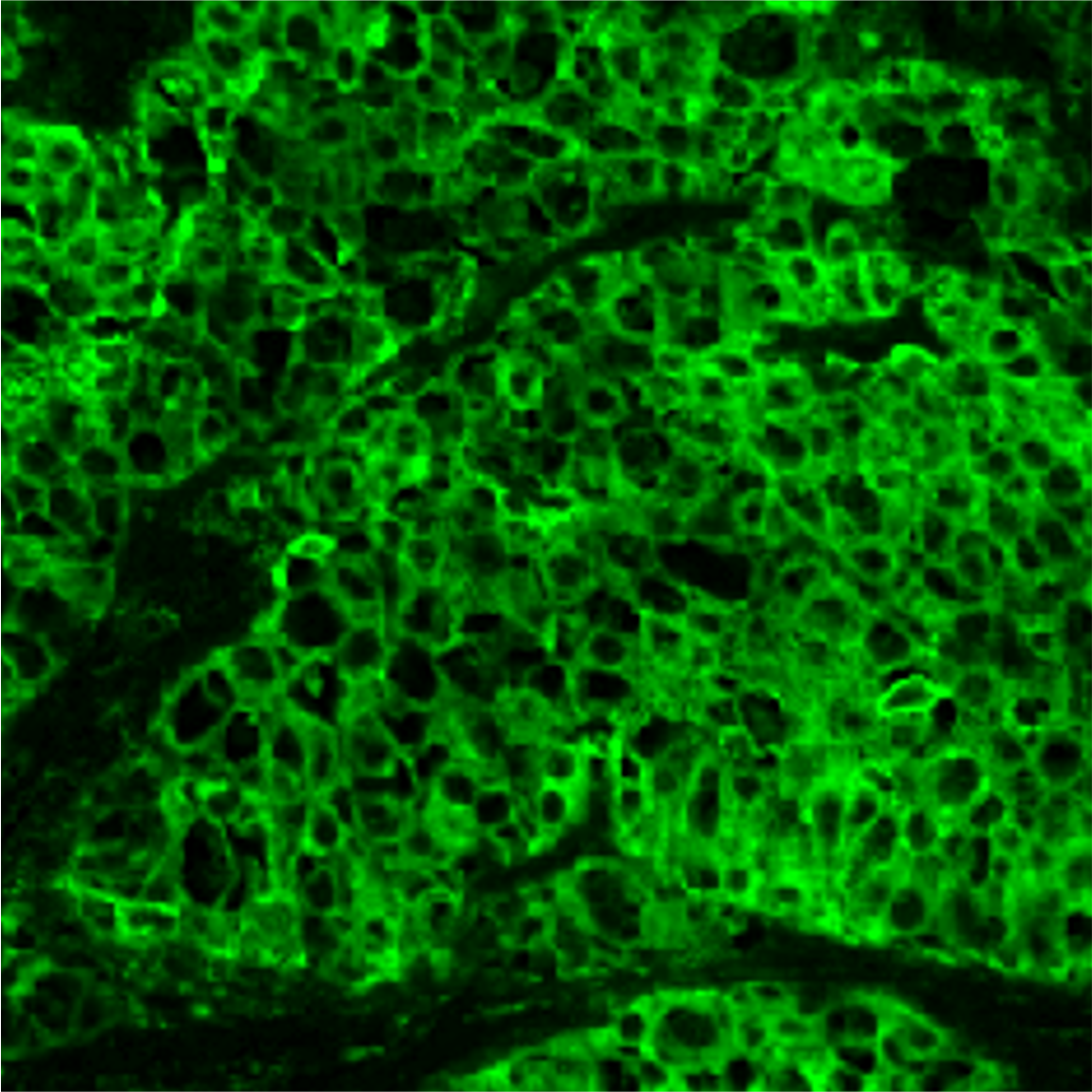

Supplement: Supplementary file 4 — Source data [file 41467_2023_38578_MOESM4_ESM.zip › Source data/Supplementary Figure 6/Supplementary Figure 6b/CD73 OE/2.png]

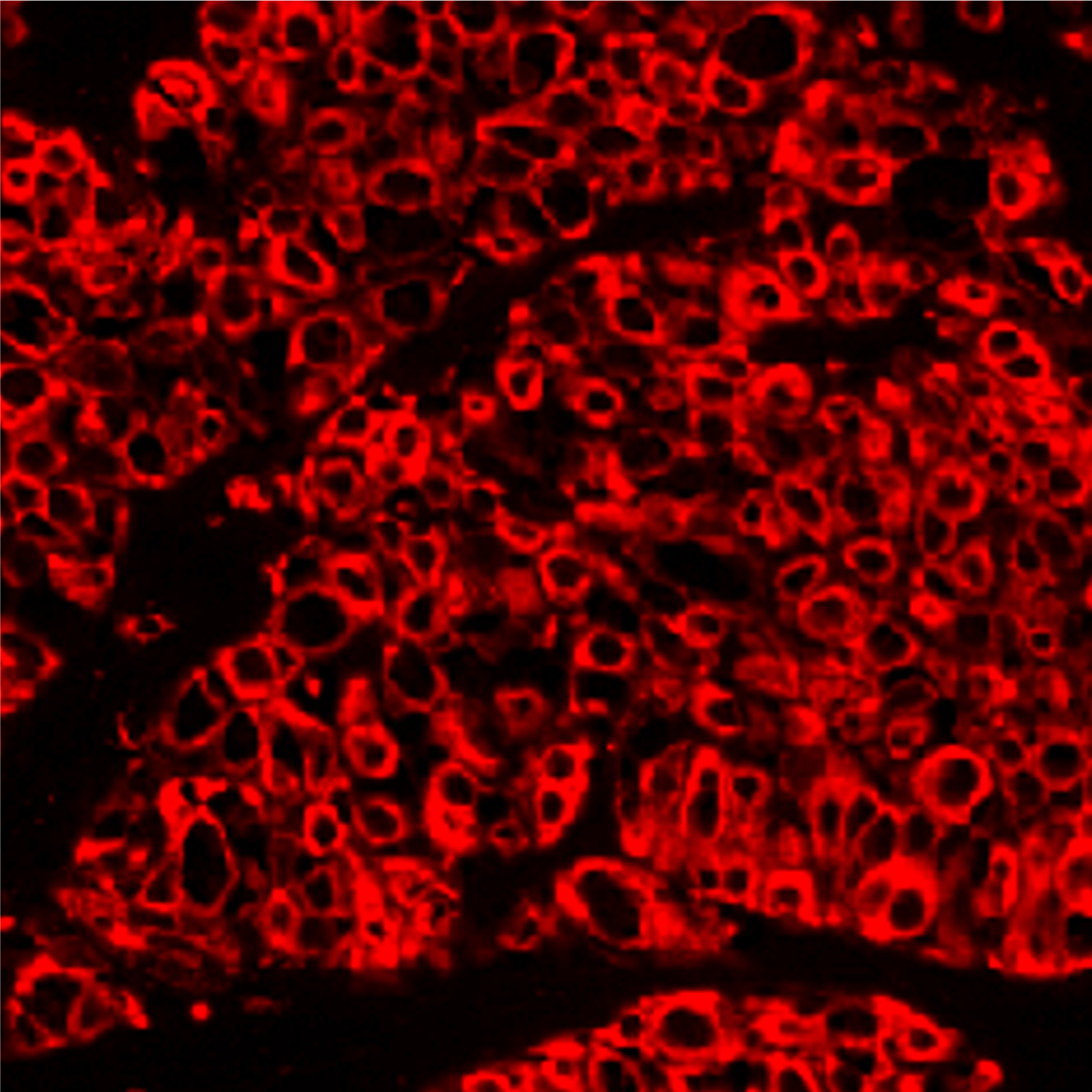

Supplement: Supplementary file 4 — Source data [file 41467_2023_38578_MOESM4_ESM.zip › Source data/Supplementary Figure 6/Supplementary Figure 6b/CD73 OE/3.png]

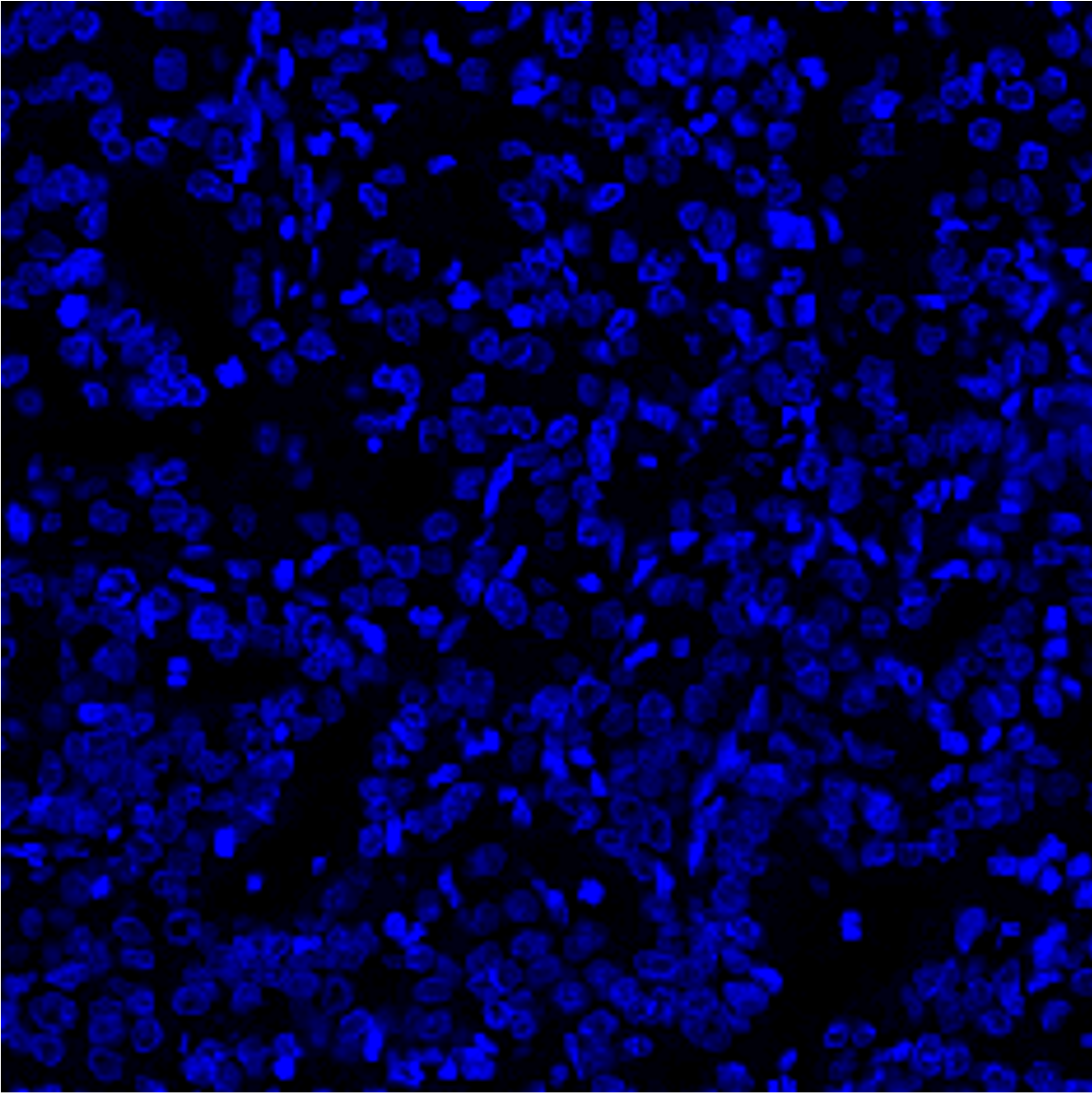

Supplement: Supplementary file 4 — Source data [file 41467_2023_38578_MOESM4_ESM.zip › Source data/Supplementary Figure 6/Supplementary Figure 6b/WT/1.png]

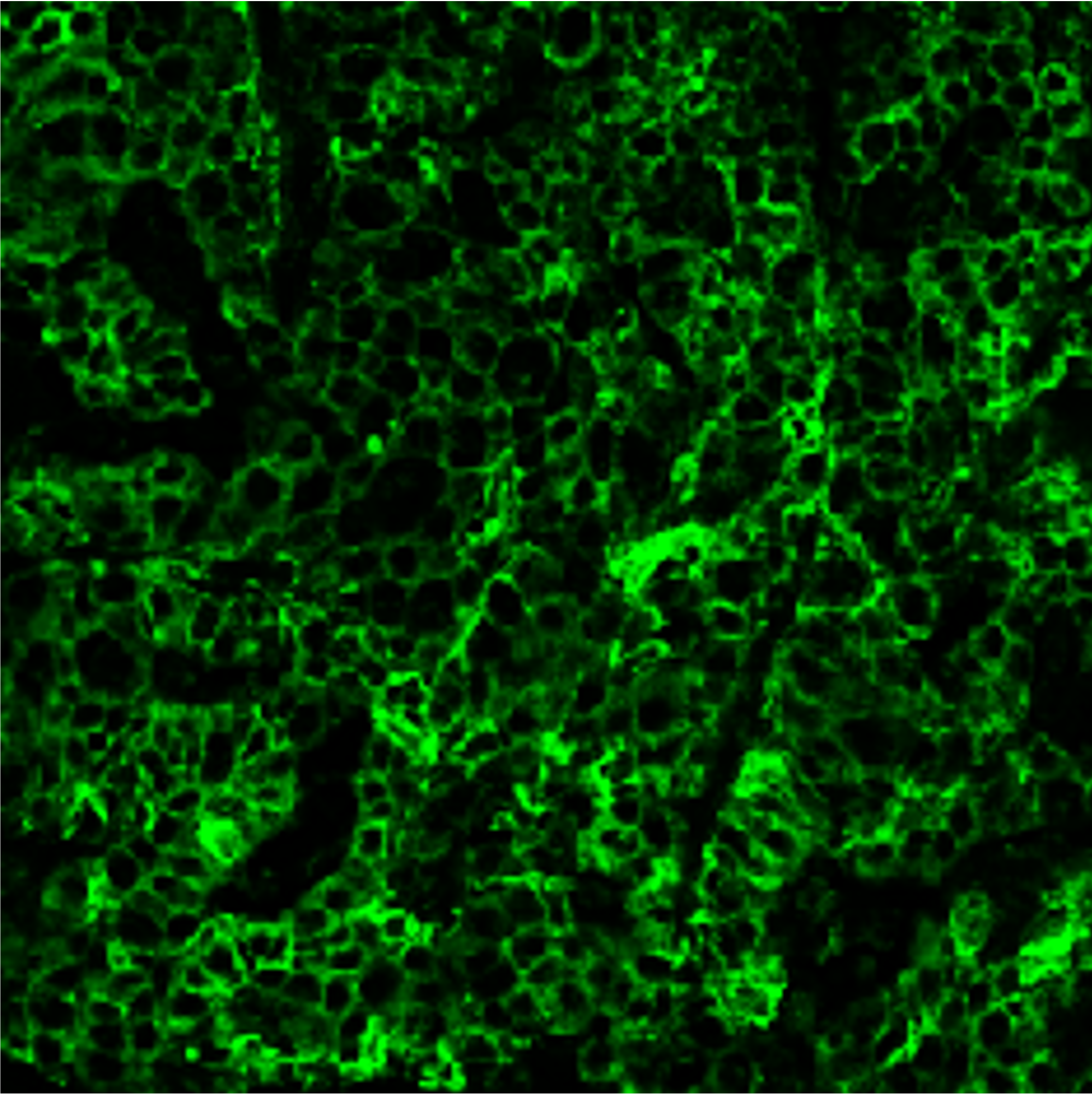

Supplement: Supplementary file 4 — Source data [file 41467_2023_38578_MOESM4_ESM.zip › Source data/Supplementary Figure 6/Supplementary Figure 6b/WT/2.png]

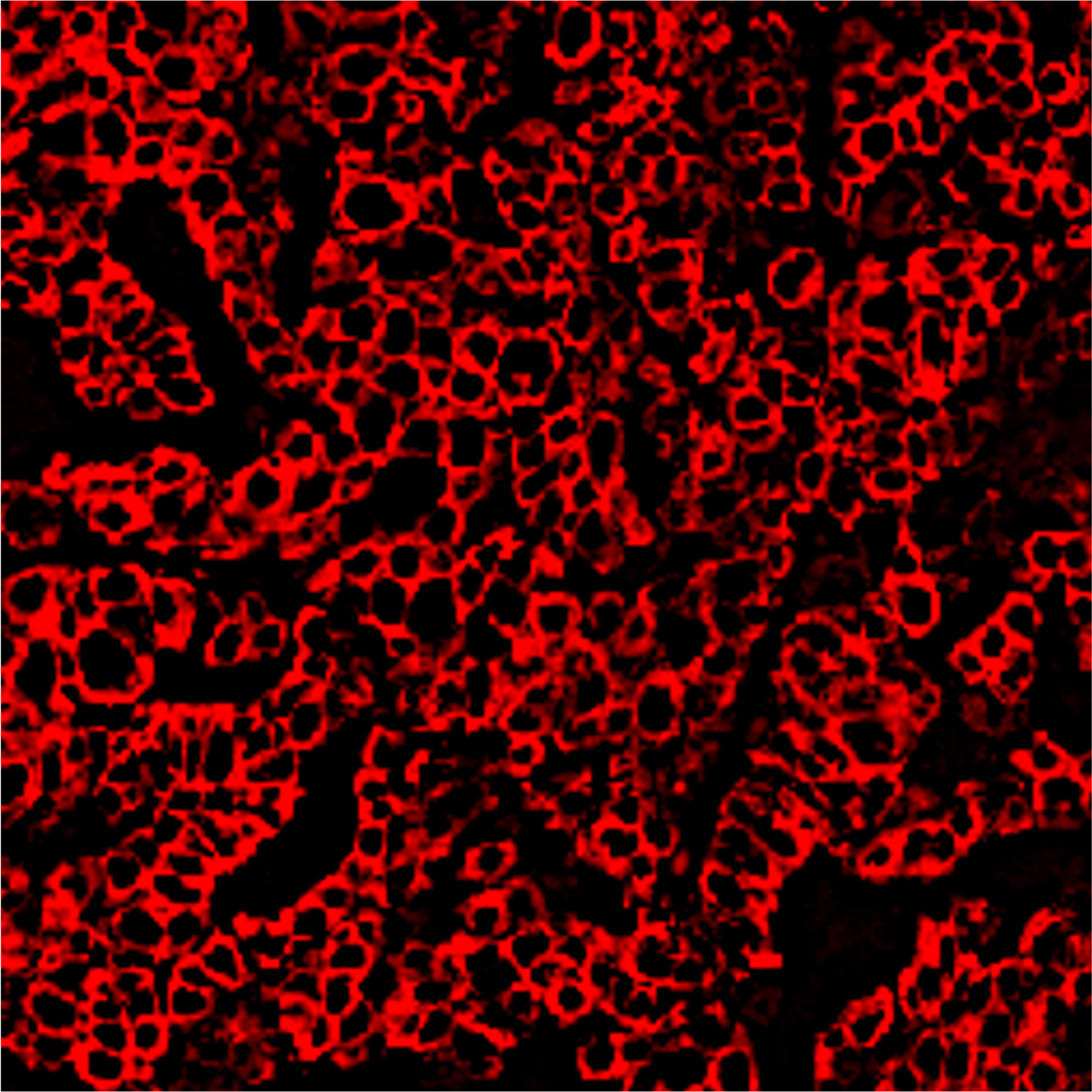

Supplement: Supplementary file 4 — Source data [file 41467_2023_38578_MOESM4_ESM.zip › Source data/Supplementary Figure 6/Supplementary Figure 6b/WT/3.png]

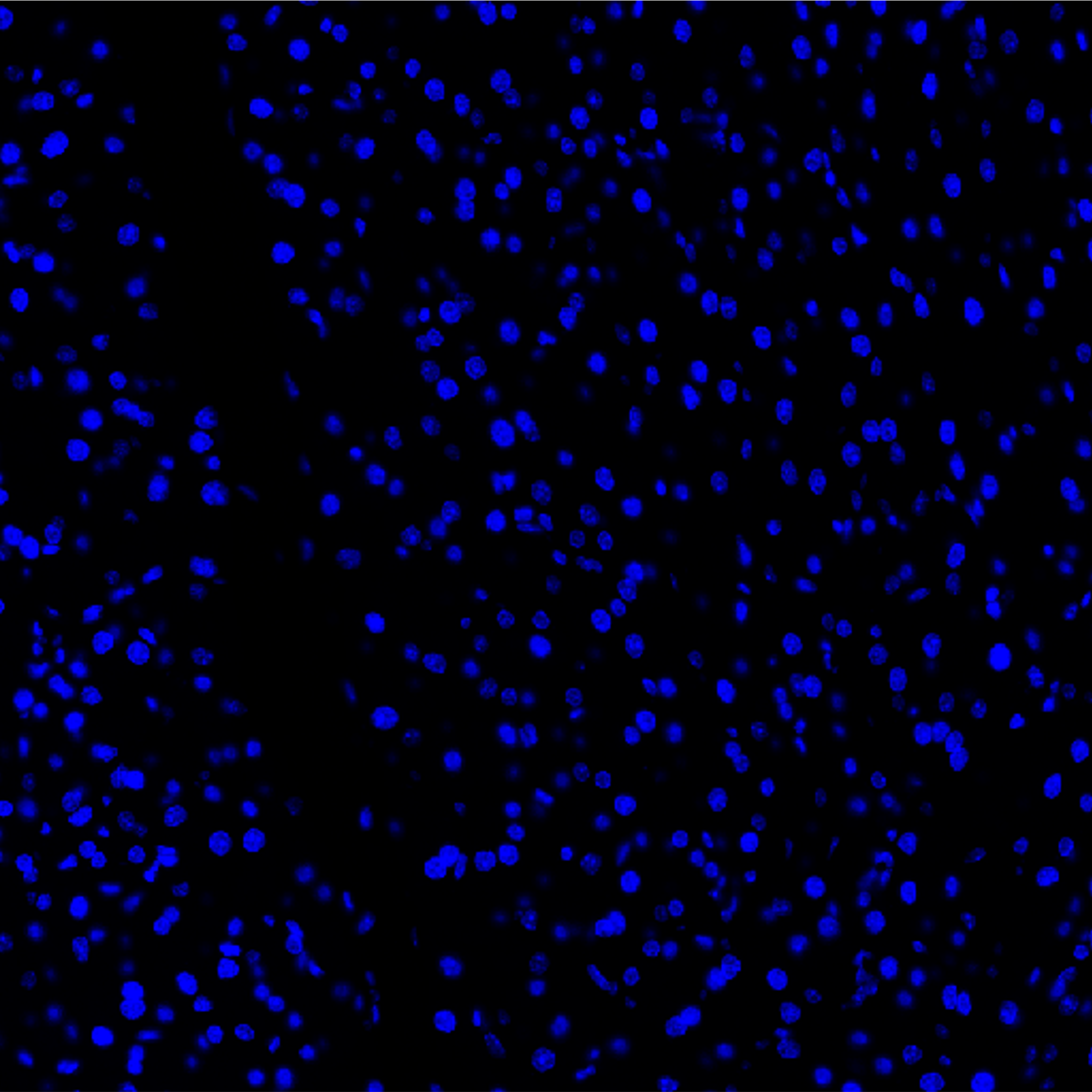

Supplement: Supplementary file 4 — Source data [file 41467_2023_38578_MOESM4_ESM.zip › Source data/Supplementary Figure 6/Supplementary Figure 6d/CD73null mice/3.png]

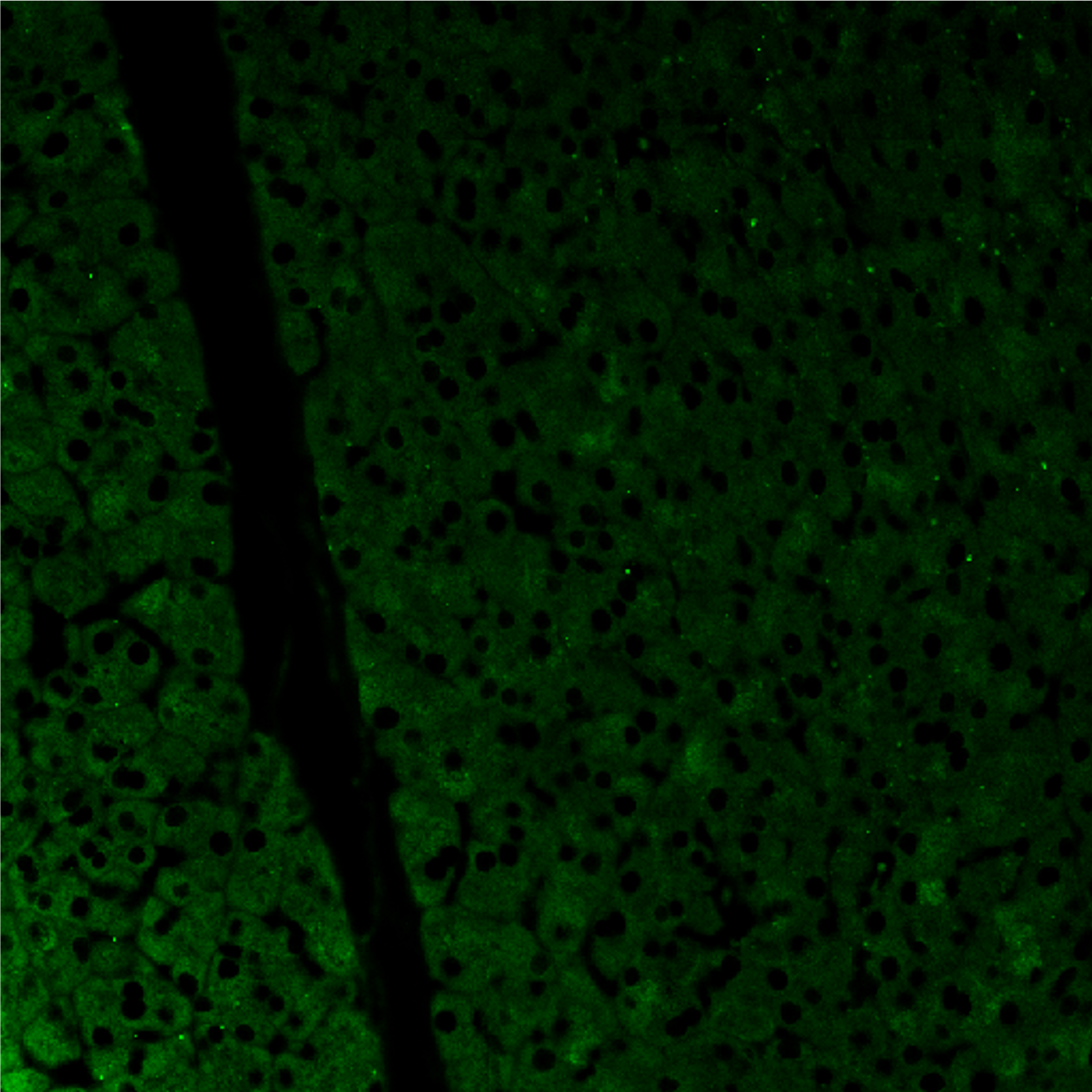

Supplement: Supplementary file 4 — Source data [file 41467_2023_38578_MOESM4_ESM.zip › Source data/Supplementary Figure 6/Supplementary Figure 6d/CD73null mice/4.png]

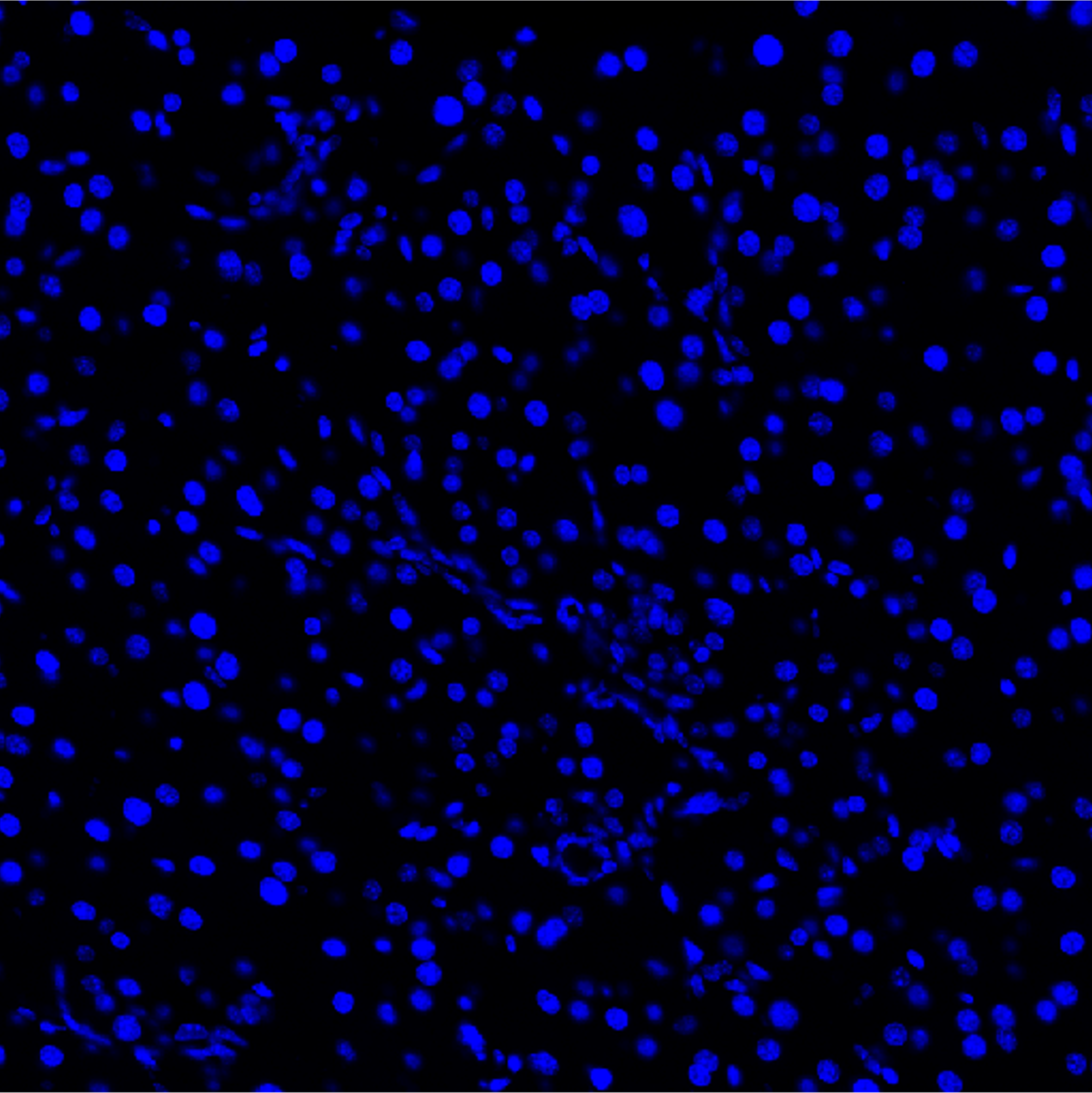

Supplement: Supplementary file 4 — Source data [file 41467_2023_38578_MOESM4_ESM.zip › Source data/Supplementary Figure 6/Supplementary Figure 6d/WT mice/1.png]

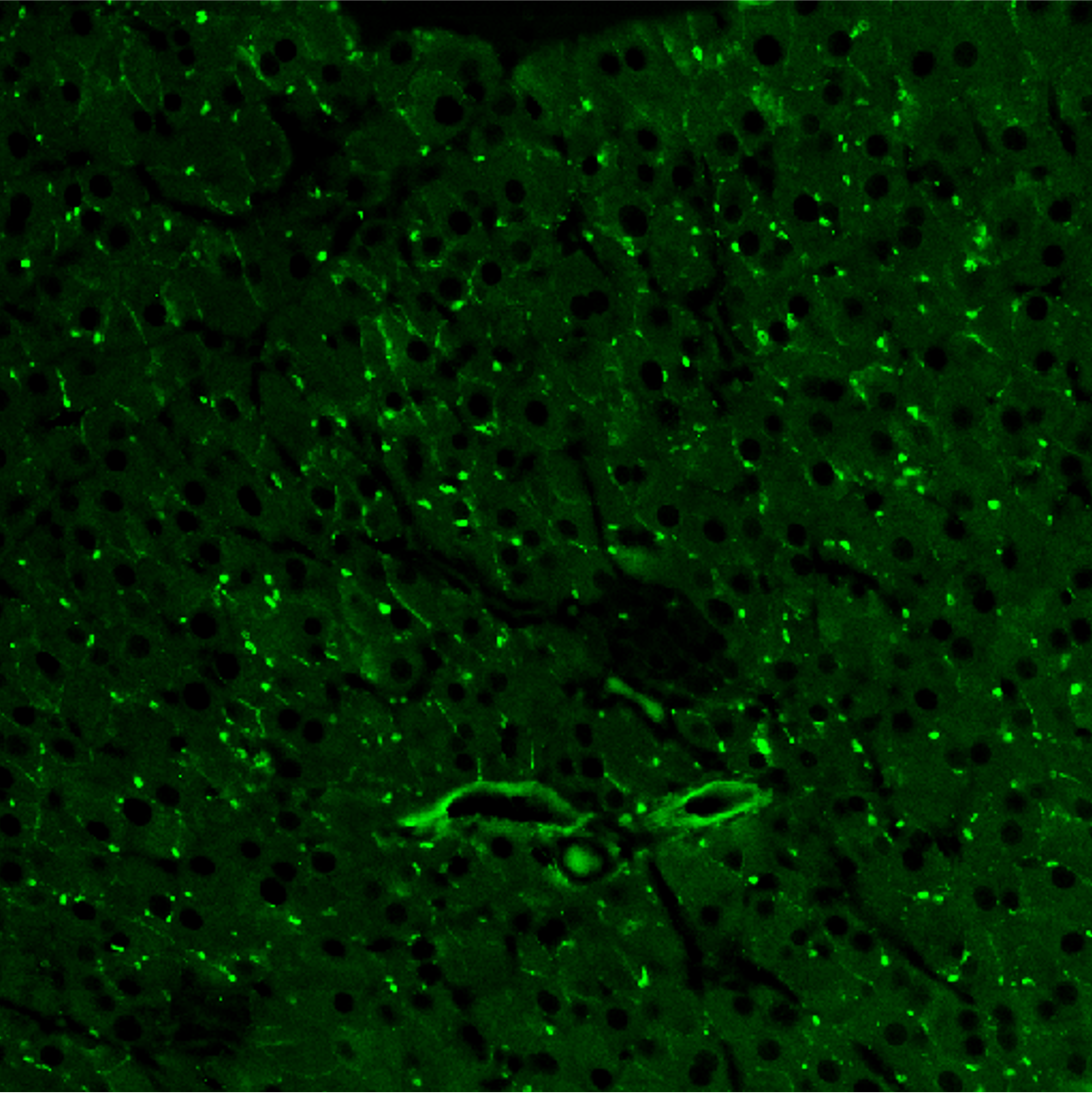

Supplement: Supplementary file 4 — Source data [file 41467_2023_38578_MOESM4_ESM.zip › Source data/Supplementary Figure 6/Supplementary Figure 6d/WT mice/2.png]

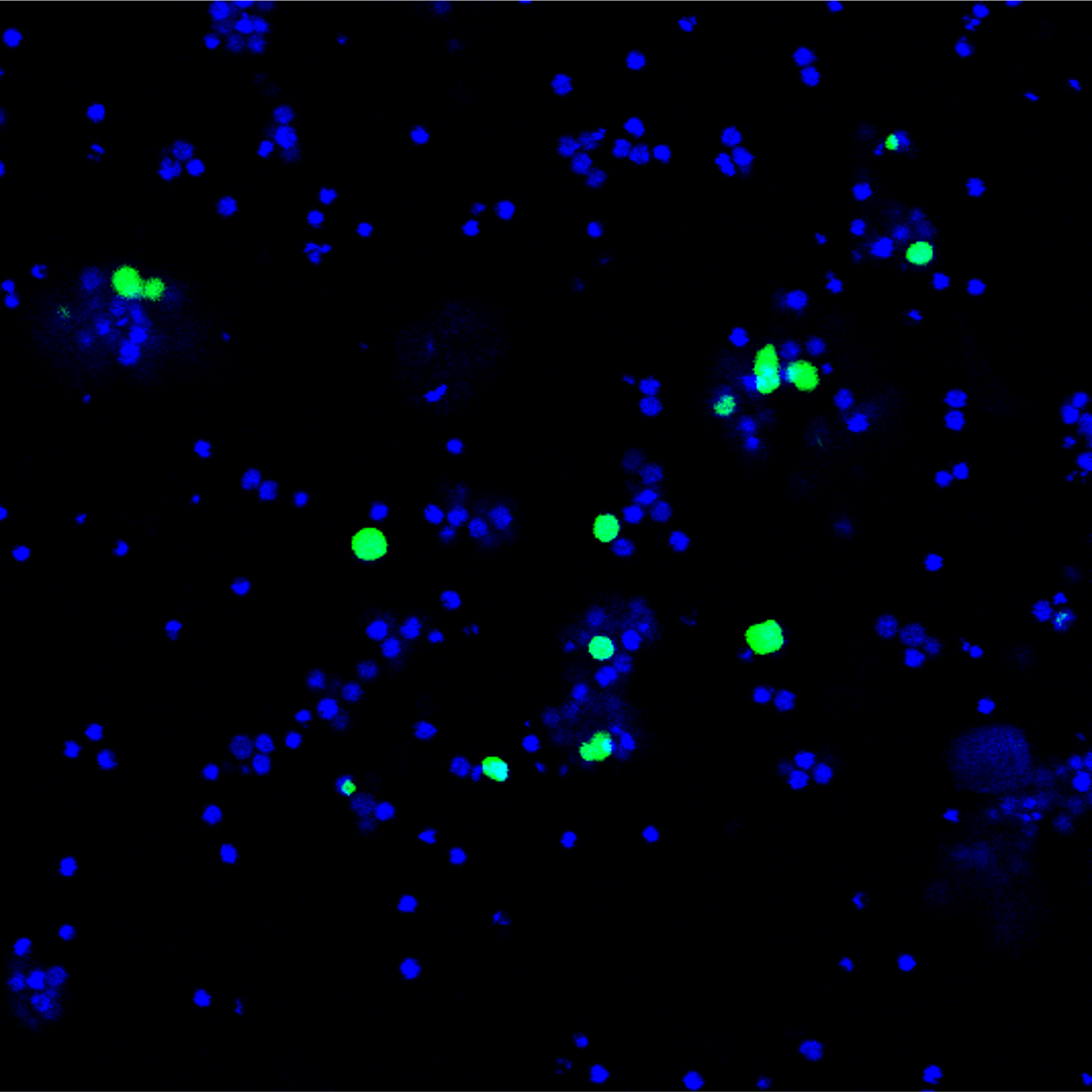

Supplement: Supplementary file 4 — Source data [file 41467_2023_38578_MOESM4_ESM.zip › Source data/Supplementary Figure 7/Supplementary Figure 7e-g/1.png]

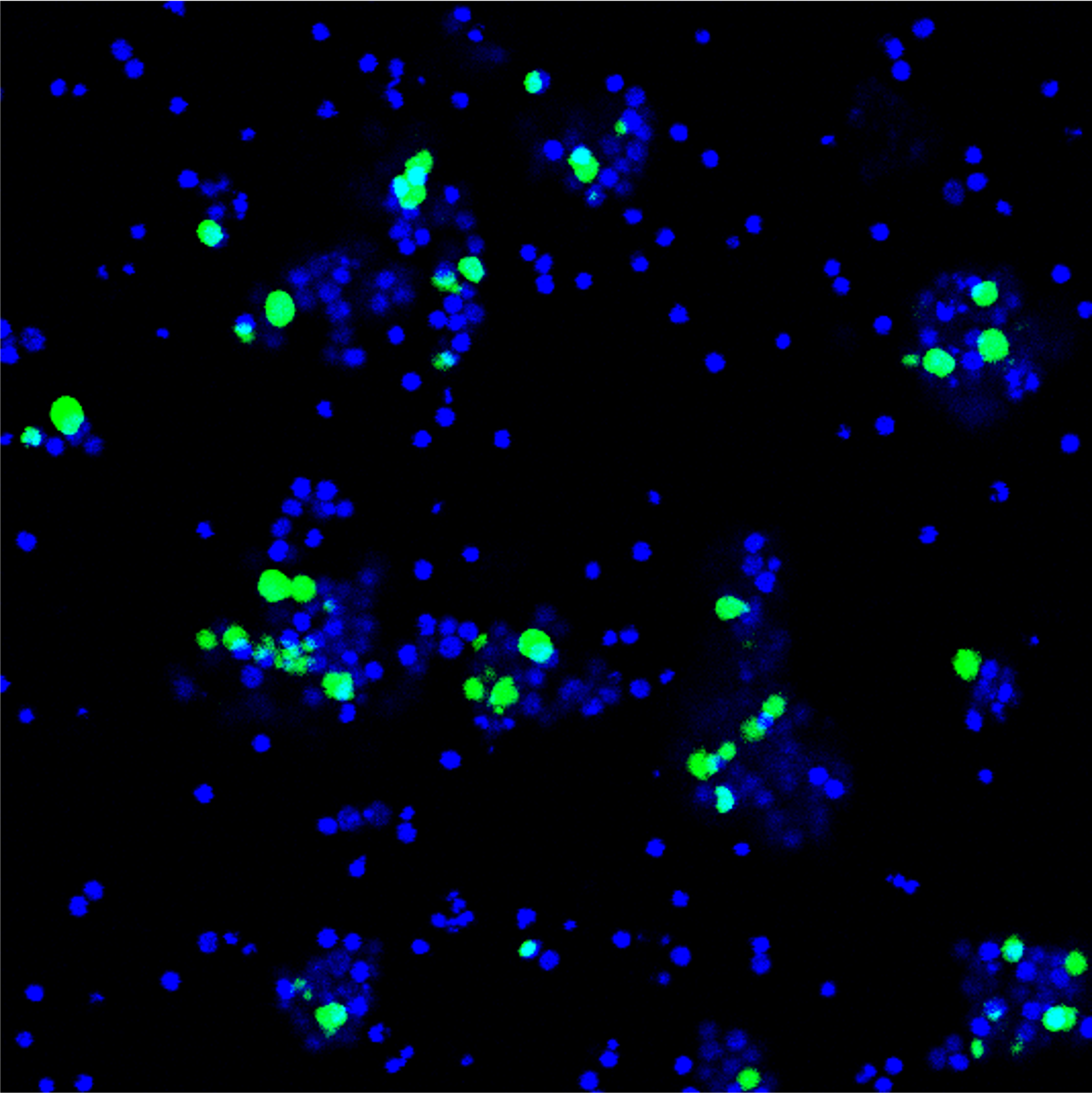

Supplement: Supplementary file 4 — Source data [file 41467_2023_38578_MOESM4_ESM.zip › Source data/Supplementary Figure 7/Supplementary Figure 7e-g/2.png]

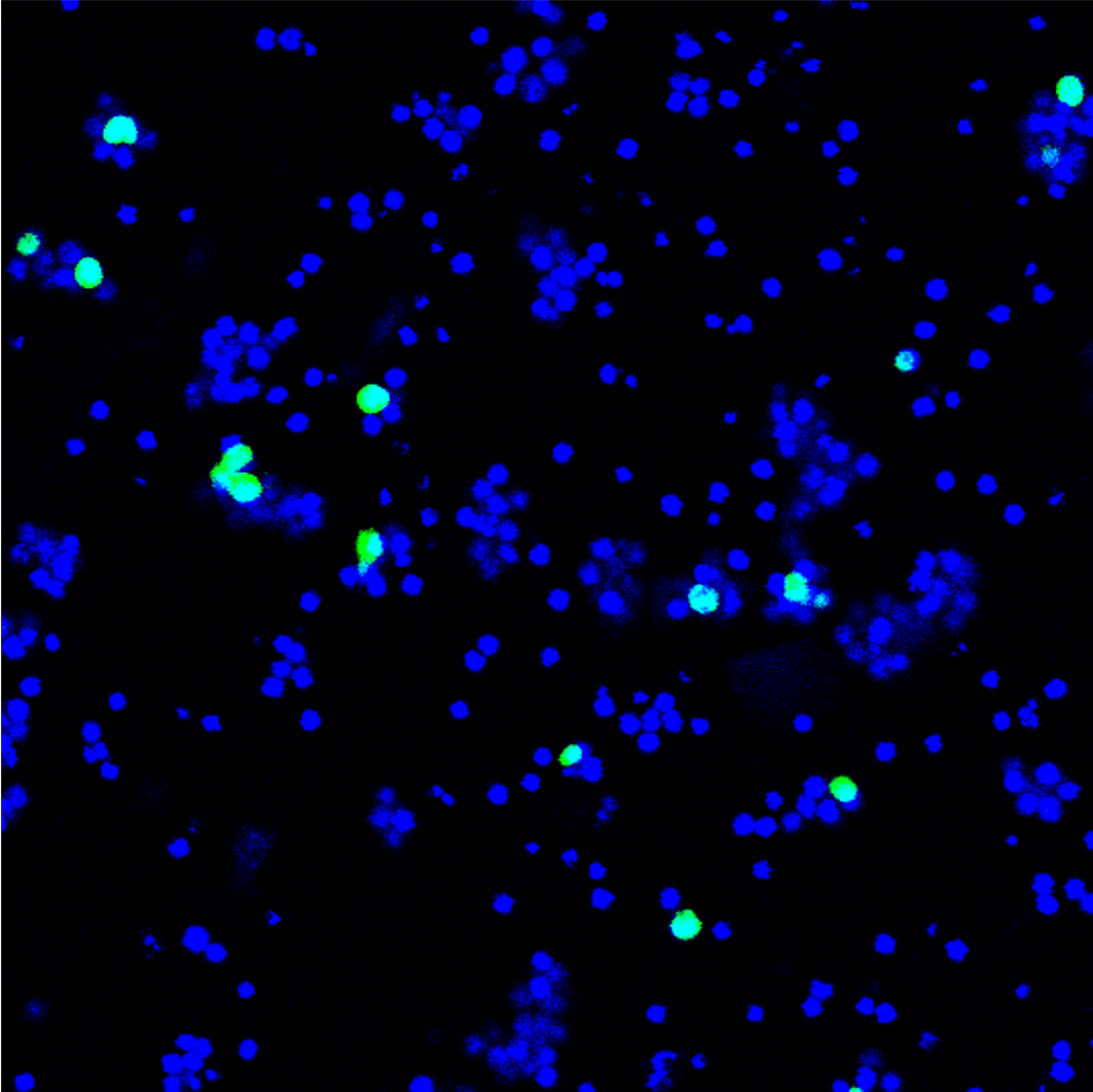

Supplement: Supplementary file 4 — Source data [file 41467_2023_38578_MOESM4_ESM.zip › Source data/Supplementary Figure 7/Supplementary Figure 7e-g/3.png]

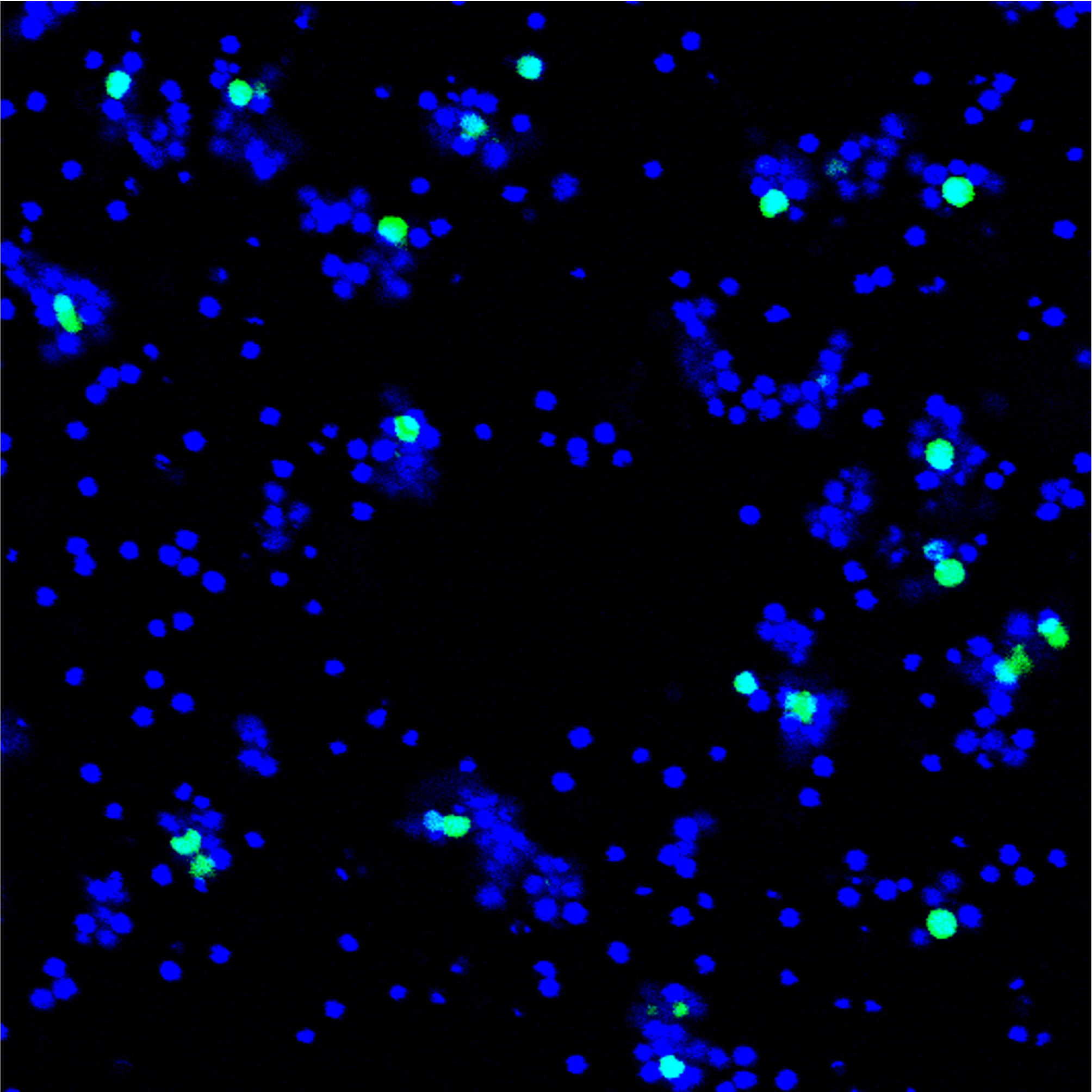

Supplement: Supplementary file 4 — Source data [file 41467_2023_38578_MOESM4_ESM.zip › Source data/Supplementary Figure 7/Supplementary Figure 7e-g/4.png]

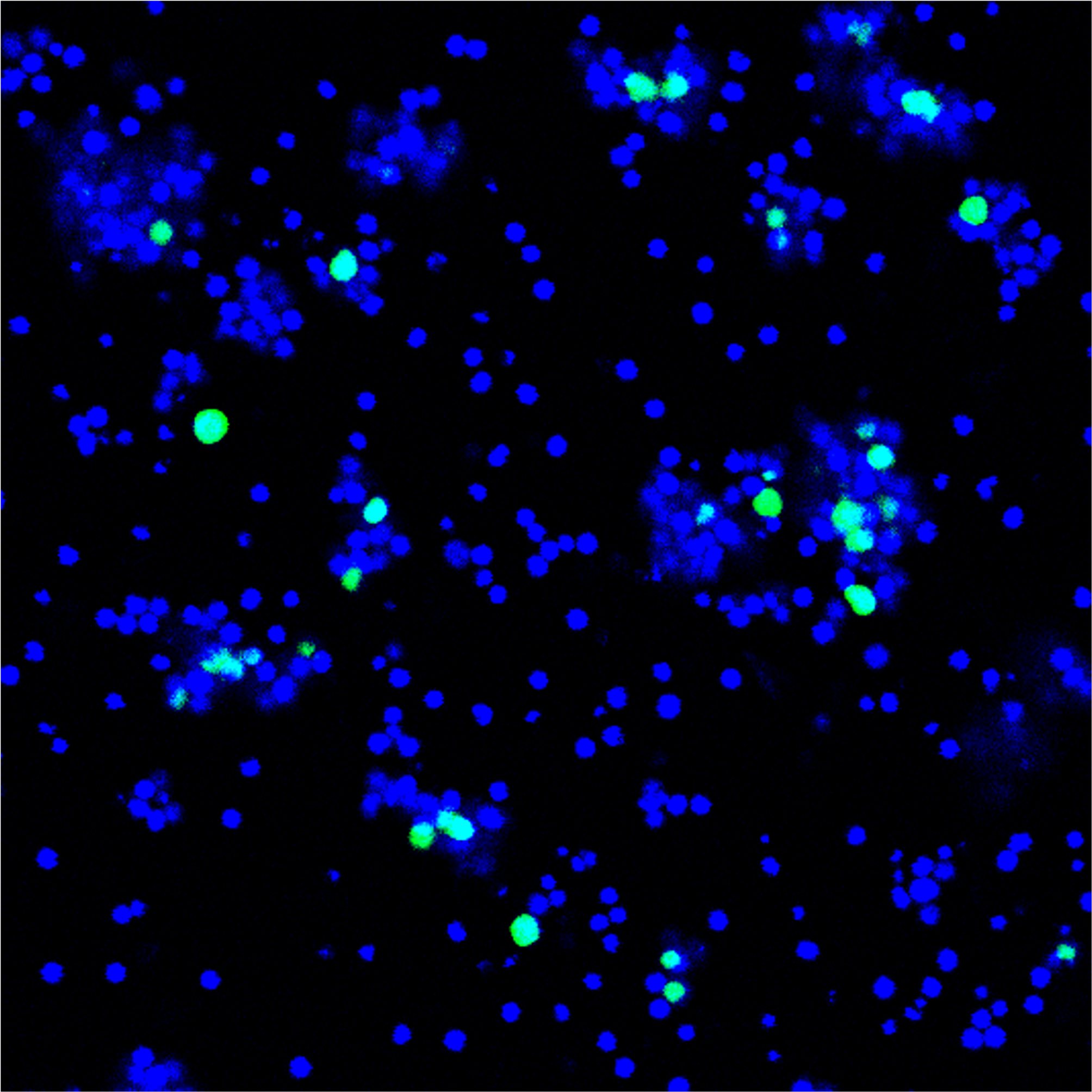

Supplement: Supplementary file 4 — Source data [file 41467_2023_38578_MOESM4_ESM.zip › Source data/Supplementary Figure 7/Supplementary Figure 7e-g/5.png]

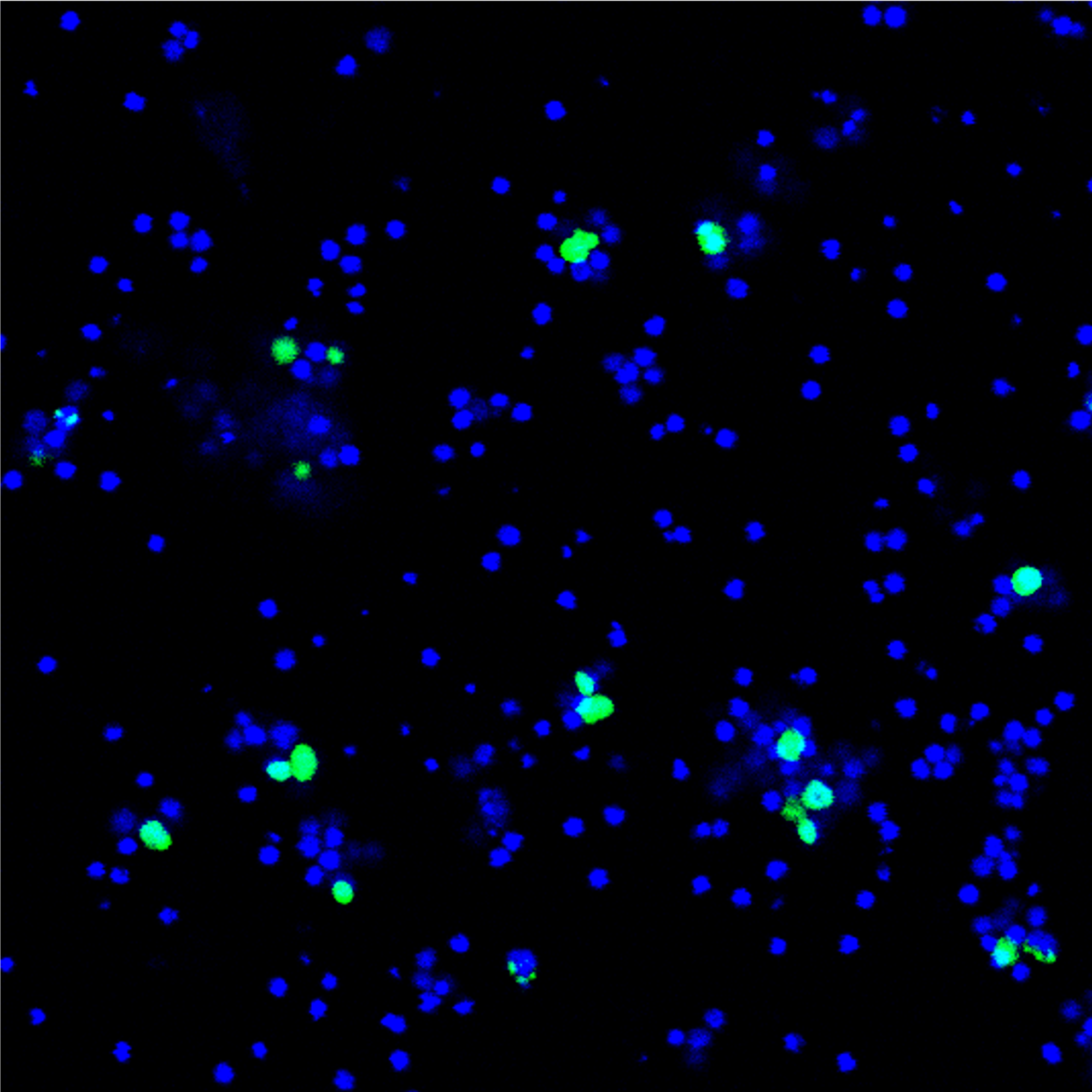

Supplement: Supplementary file 4 — Source data [file 41467_2023_38578_MOESM4_ESM.zip › Source data/Supplementary Figure 7/Supplementary Figure 7e-g/6.png]

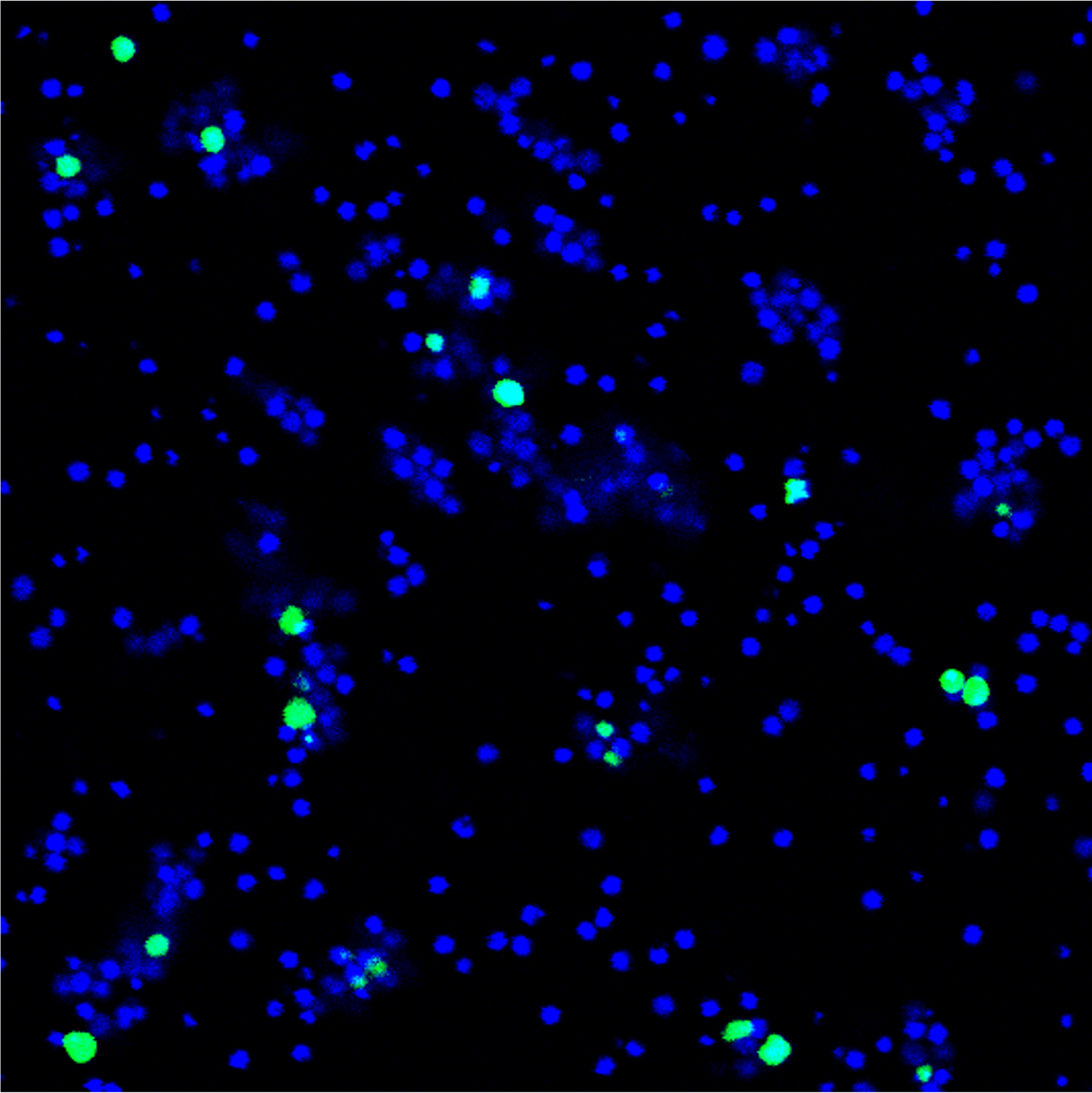

Supplement: Supplementary file 4 — Source data [file 41467_2023_38578_MOESM4_ESM.zip › Source data/Supplementary Figure 7/Supplementary Figure 7e-g/7.png]

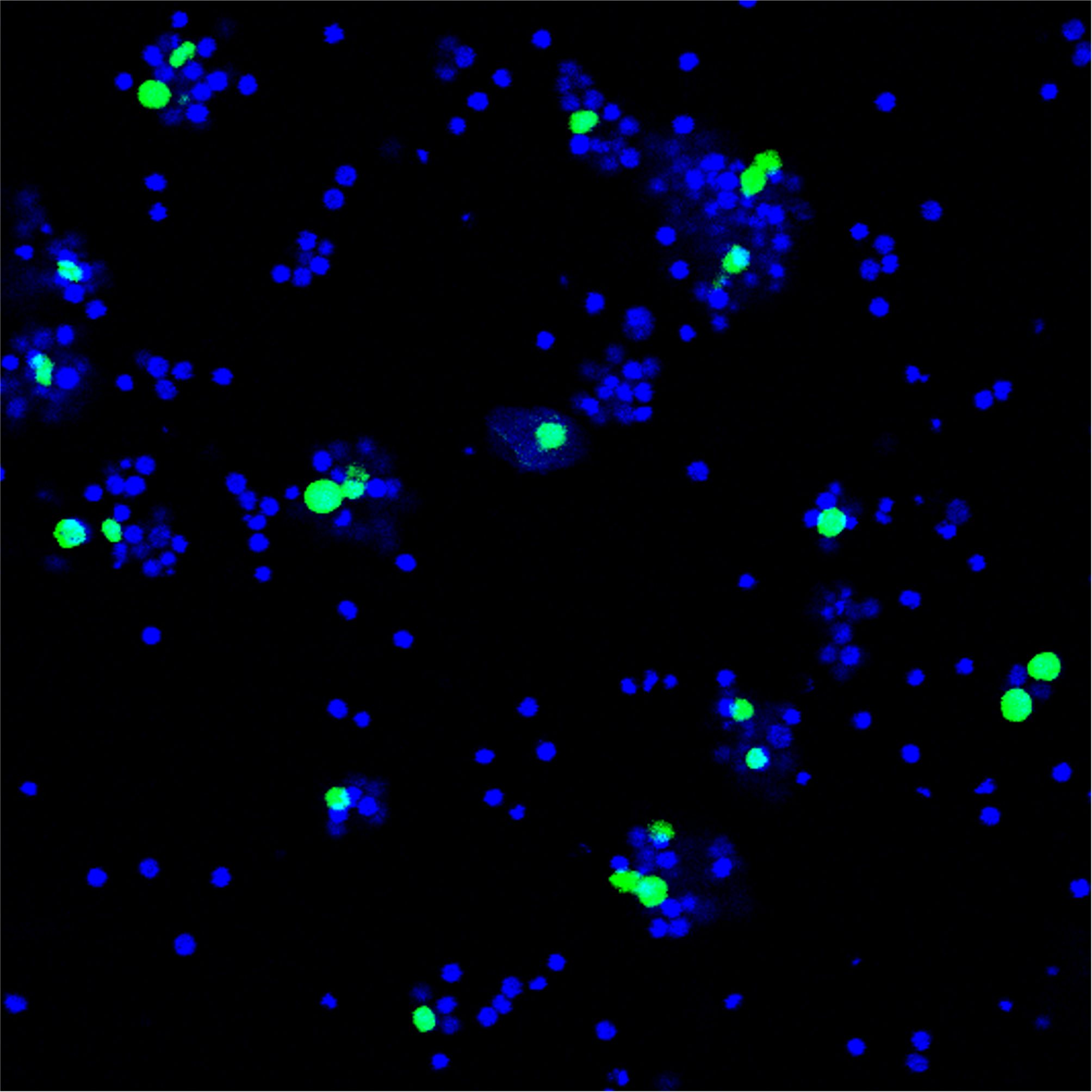

Supplement: Supplementary file 4 — Source data [file 41467_2023_38578_MOESM4_ESM.zip › Source data/Supplementary Figure 7/Supplementary Figure 7e-g/8.png]
