# Supplementary material for: Transcriptional control of pancreatic cancer immunosuppression by metabolic enzyme CD73 in a tumor-autonomous and -autocrine manner
Source: Nat Commun. 2023 Jun 8;14:3364. doi: 10.1038/s41467-023-38578-3 (PMC10250326; doi:10.1038/s41467-023-38578-3)
Supplement: Supplementary file 2 — Peer Review File [file 41467_2023_38578_MOESM2_ESM.pdf]

nature portfolio

Peer Review File

Transcriptional control of pancreatic cancer immunosuppression by metabolic enzyme CD73 in a tumor-autonomous and -autocrine manner

Open Access This file is licensed under a Creative Commons Attribution 4.0 International License, which permits use, sharing, adaptation, distribution and reproduction in any medium or format, as long as you give appropriate credit to the original author(s) and the source, provide a link to the Creative Commons license, and indicate if changes were made. In the cases where the authors are anonymous, such as is the case for the reports of anonymous peer reviewers, author attribution should be to 'Anonymous Referee' followed by a clear attribution to the source work. The images or other third party material in this file are included in the article's Creative Commons license, unless indicated otherwise in a credit line to the material. If material is not included in the article's Creative Commons license and your intended use is not permitted by statutory regulation or exceeds the permitted use, you will need to obtain permission directly from the copyright holder. To view a copy of this license, visit http://creativecommons.org/licenses/by/4.0/.

REVIEWER COMMENTS

Reviewer #1 (Remarks to the Author):

In the current manuscript, the authors investigate the role of membrane CD73, a critical enzyme in ATP metabolism, in pancreatic cancer. CD73 can be expressed on the cell surface, and this results in extracellular accumulation of adenosine which, in turn, inhibits the activity of infiltrating lymphocytes. In the current study, the authors explore a different expression of CD73, whereby membrane expression of this enzyme results in activation of the mapk38/Stat1 axis and, in turn, expression of CCL5, driving recruitment of regulatory T cells and suppression of CD4 and CD8+ T cell mediated anti-tumor immunity. The authors use genetic inactivation or overexpression of CD73 as well as pharmacological inhibition, and use different pancreatic cancer cell lines (KPC, PANC02, BXPC3) both in orthotopic and subcutaneous transplantation models. Overall, this article provides another mechanism of immunosuppression in pancreatic cancer, a disease characterized by lack of effective antitumor immunity and resistance to immunotherapy.

Main Comments:
The data supporting membrane expression of CD73 in human and mouse pancreatic cancer is limited to some immunohistochemistry and immunofluorescence staining; the data would be greatly strengthened by including co-IF with epithelial membrane markers (K19, pan-CK…) and by showing high magnification images. Further, antibody specificity should be validated, at least in mouse, by staining in parallel tumor derived from CD73 KO cells and CD73 OE cells. Strong data supporting membrane expression is fundamental to justify the rationale of this study.

While the notion that CD73 expression activated CCL5 expression is novel, the authors should determine whether there are changes on extracellular adenosine when CD73 is knocked out/overexpressed -and whether those changes cause the immune phenotype, in addition, or rather than CCL5 expression changes.

The authors should explain how the cell lines for this study were selected. Was only one KPC cell line used? KPC cells vary greatly in terms of eliciting an immune response, and it is important to validate results across lines. In addition, Panc02 cells (derived from chemical carcinogenesis) are highly immunogenic, do not harbor the hallmark mutations of pancreatic cancer in the KRAS gene, and are generally not considered a valid model for the human disease. BxPC3 cells also have wild type KRAS, representing less than 5% of human tumors; they are thus an unusual choice when a single human line is used.

Minor concerns
The flow data and CyTOF data do not match- the first shows an increase in CD8+ T cells while no change is observed in CyTOF. This inconsistency is not completely unusual but should be explained.

The Y axis of many panels in 2 are labeled as percentage but it is unclear what the total is: total cells? Immune cells? Total T cells?

Page 12, last paragraph – the rationale for using chloroquine and MG132, and the biological effect of those drugs, needs to be explained.

There is a concern as to many of the findings being based on overexpression models. CCL5 expression should be examined in CD73 KO tumors as well.

Reviewer #2 (Remarks to the Author):

In the present studies, the authors provide evidence for a functional role of CD73 in promoting immune supression during pancreatic cancer which promotes disease progression. Their findings suggest that targeting CD73 with CD73 inhibitors in protective during pancreatic cancer. The studies are well done and their data interpretation is supported by ample data. I have some suggestions to further improve a strong manuscript:

Major comments:

1.) Can the author provide some evidence that there targeting approach resulted in attenuated adenosine production? The authors may consider to use APCP as an alternative approach to blocking CD73. They may find APCP more effective in dampening CD73 function than the antibody treatment.

2.) The authors may consider expanding their introduction to explain in more detail the functional role of CD73 in extracellular adenosine production (e.g. PMID: 23338058, PMID: 16799190 and PMID: 23263788) and it's role in modulating regulatory T-cell responses, including its function in inflammation (e.g. PMID: 23413361 and PMID: 22389701) versus cancer (PMID: 35360246).

3.) Can the authors provide some genetic evidence for a functional role of Adora2a signaling in their responses? Also, have the authors considered a role of the Adora2b receptor? This should be at least discussed in more details.

Minor comment:

1.) Can the authors expand in their introduction on more broadly introducing adenosine receptor signaling functions, e.g. include the heart rate slowing effects of Adora1 (e.g. PMID: 19707555) or Adora2b in ischemia and reperfusion (PMID: 23540714), and Adora2a during inflammation (PMID: 36009485). It would be helpful for the broad readership of this journal to give a more general introduction.

2.) The authors may also discuss the functional roles of hypoxia in modulating CD73 and adenosine signaling (e.g. PMID: 28798196, PMID: 24391213), and also how this is important during pancreatic cancer.

3.) The authors may consider using the correct nomenclature for adenosine receptors - Adora1, Adora2a, Adora2b, Adora3.

Reviewer #3 (Remarks to the Author):

Tang et. al. show that CD73 on pancreatic tumor cells transcriptionally regulates CCL5 through STAT1 which results in the increased recruitment of immune-suppressive Treg cells to the tumor sites, thus facilitating tumor immune escape. The manuscript touches an important and interesting issue and proves the main claims in a well-designed way. CCL5 has been traditionally seen as a pro-tumor molecule, but the recent literatures have shown an anti-tumor molecule side of CCL5. This manuscript also illustrates the anti-tumor function of CCL5, thus facilitating more research on CCL5 field. The manuscript is even more interesting in that it provides a new mechanism of CCL5 regulation by a metabolic enzyme CD73, thus connecting a metabolic pathway with a chemokine expression. However, this reviewer feels uncomfortable to recommend the publication of this manuscript at Nat. Commnun. with the current status. As this reviewer explains below with 3 major points, overall, the way of data presentation and the quality check of the manuscript do not meet the criteria and standard of this journal (please see the Major points 1 and 2). Also, this reviewer asks little more experiments (if the authors do not possess such data already) to clearly illustrate the important point of the receptor side of the story (please see the Major point 3).

MAJOR POINTS

1. The Fig. 1 sets the scientific basis of this manuscript by testing the combinatorial immunotherapies with CD73 and PD-1 inhibition. The results are interesting, convincing and persuading, but this reviewer does not feel comfortable with inconsistent presentation of data throughout the manuscript. Some but not all examples are shown below.
a. For CD73 inhibition, an anti-CD73 antibody is used in panels A-E, meanwhile a CD73 inhibitor is used in panels F-K. While the panels A-K are done in ortholog implantation, additional data on non-ortholog implantation are presented in panels J-N. But in each case, the analysis methods are presented inconsistently without providing apparent reasons.
b. For example, with the anti-CD73 antibody inhibition experiment, Luciferase signals (B) and tumor weights (C), mice weight (D), T cell composition (E) and POS (F) are presented. But with the CD73 inhibitor experiment, Luciferase signals and POS are not shown, but instead tumor volume (H) are additionally presented which is not shown for the anti-CD73 antibody experiment.
c. As another example, in Fig.1 legend, panels A-E are indicated to use the orthotopic model. However, it is not clear what model the F panel is using from the figure legend. By reading the main text, this reviewer can guess that the panel F must be the orthotopic model. Therefore. the figure legend should say that the panels A-F not A-E are the orthotopic model. Then, the following panels G-K do not have any description of the models used. Again, by reading the main text, it appears that panels G-K must be the subcutaneous model.
d. Also, Fig. 2 CyTOF analysis uses the CD73 inhibitor with the orthotopic model. Since Fig. 1 used anti-CD73 antibody or inhibitor with the orthotopic or subcutaneous model, we need a specific reason for the choice of the CD73 inhibitor with the orthotopic model in Fig. 2.
e. Fig. 3J is in vivo experiment encompassing 3 weeks which makes possible that Treg could have proliferated more rather than just migrated more. Of course, this point has been addressed in the chemotaxis assay (Fig. 3G-I), however, these experiments are done with mouse Treg. Therefore, it is critical to perform the same chemotaxis assay with the human Treg cells to claim that CD73 on tumor cells affect the migration not the proliferation of human Treg cells. Please provide an explanation of different uses of Tregs (mouse or human origin) in panels Fig.3 G-J.
f. In the in vivo experiments, BXPC-3 tumor is used in Fig. 3J, but KPC tumor is used in Fig. 3K. Also, Fig. 3J uses the orthotopic model, but the subcutaneous model is used in Fig. 3K. not explained, and the tumor volume/weight is measure only with KPC but not with BXPC-3.

The authors use two inhibition methods, two mouse models, different tumor cell lines. Therefore, it is crucial to clearly indicate what is used in each panel throughout the manuscript (especially Fig. 1-3). More importantly, please provide the explanation or rationale about these inconsistent uses of methods and models and cells throughout the manuscript.

2. In Fig. 2C, the decreases of PD-1, TIM3, FoxP3 are observed and this becomes one of the main claims of this manuscript. However, the most significantly decreased cluster appears to be the C03 cluster which unfortunately has no assignment of a cell lineage. C03 does not appear to be lymphocytes since C03 has no CD3e, CD4 or CD8 expression in Fig S3A, while having expression of NK1.1 and FoxP3. Can the authors elaborate about the potential identity of this NK1.1 high and FoxP3 high C03 cluster? Why does C03 have the highest FoxP3 even though C03 has no CD4 expression? Due to this strange tSNE clustering, this reviewer cannot be confident that this unsupervised clustering has been properly performed.
This makes one of the major claims of the manuscript does not hold the stand. Therefore, this CyTOF results of Fig. 2 does not fully support the conclusion of Fig 1 (which stresses that the inhibitory effects of the combination therapy is completely T-cell dependent (Lines 194-197).

3. The main theme of the manuscript is the tumor-infiltration of Treg by the CCL5 production by tumor cells through CD73. Therefore, the migration/infiltration of Treg cells as well as other lineage cells needs to be examined more thoroughly.
a. The authors performed blocking CCL5 to inhibit Treg migration in vitro (Fig. 4O) and in vivo (Fig.

7). It should be considered that not all Treg cells express CCR5, one of the major cognate receptors of CCL5. Therefore, it is crucial to show the expression of CCR5 on Tregs and CD8 T cells. This reviewer strongly asks to examine the CCR5 expression levels on the Treg cells used in the chemotaxis assay (Fig. 4O) as well as both Treg and CD8 T cells of the in vivo assays (Fig. 7D).
b. The effect of tumor inhibition cannot be judged from a single-time point. Since Fig. 7 has an important position in the manuscript, the reviewer strongly asks to show the tumor volume graph with multiple time points for Fig. 7 experiments as in Fig. 1H.
c. CCL5 is expressed mainly by macrophages, NK cells and activated CD8 T cells, not only tumor cells. It will be necessary to stain CCL5 of TIL samples to show and compare CCL5 expression in a more systemic way in Fig. 7.

MINOR POINTS

1. Page 6 lines 122-124 in the Introduction tells the most important part of the Introduction section since the rationale of the research aim is presented. However, this description can be comprehended in a wrong way, so it could be rewritten to better convey the concise meaning of "immunosuppressive pathways inside tumor cells".

2. In Fig. S1C, most CD8+ cells are TNFa or IFNg single positive. From the experience of the reviewer with B16F10 and MC38 tumor-burden models, many CD8+ cells become double positive for TNFa and IFNg, especially after PD-1 treatment. Is this phenomenon common for KPC cells?

3. The figure legends needs more attention to clarify which mouse model (orthotopic or subcutaneous). For example, the model used by panel G of Fig. 1 is not described in the main text nor the figure legend. Also, the Figure 2 legend does not state the model used either.

4. Fig 1N highlights the superior tumor-suppressing activity of CD4 T cells which is a very interesting observation. Since the Fig S2 only show the percentages of CD8 T cells, inclusion of CD4 T cell percentages in Fig S2 should be included to properly access the outcome of Fig 1N. Also, FoxP3 staining in TILs might be necessary to support the claim of Fig 2.

5. Fig. 2A shows several populations, but the names are not consistent in the uses of plurals. For example, Macrophage and DC can be Macrophages and DCs to be consistent with the remaining.

6. Fig. 2B illustrates that there are no statistically significant changes in the cell population after CD73 inhibition. However, there are very strong presence of subtypes within Macrophages and Granulocytes (just below the names of the populations) which disappear after CD73 inhibition. Do the authors have any insight on these populations? This can be noted similarly to Fig. 2C.

7. The last graph of Fig. 2C says "TIGHT". Is this TIGIT? If so, please recheck spelling errors throughout the manuscript which is quite abundant. Also, If this is the exhaustion marker TIGIT, it should be mentioned in the main text or at least in the figure legend even if it is statistically not significant.

8. T cell compartments in Fig. S3B show a quite high expression of MHCII and Ly6c which are typically associated with B cells or myeloid lineages. Can the authors explain this peculiar expression?

9. I might have missed, but where the information about the CD73 KO mouse?

10. Fig. S4 legend put the description of G-J ("Blue dots: DAPI, Green dots: EdU") into C-F description area.

11. The colors of Fig.3 M-P does not match Fig. 3L, thus confusing.
12. Fig. S5C shows the activation status of CD8 T cells by IFNg. However, the small increase from

22% to 28% makes the conclusion from Fig. S5 less convincing. This reviewer asks to perform multiple repeats with the statistical analysis on all the data on Fig. S5.

13. Fig. 4 A-C examines chemokine expression from CD73 KO tumor cells and D-G from CD73 OE tumor cells according to the figure, but the main text says the opposite. Which one is right?

14. The second important message of this manuscript is the changes in CCL5 levels from tumor cells with CD73 expression. The authors examined 25 chemokines, but only several ones are shown in Fig. 4B. Since this is an important data, the review insists to show all 25 chemokines.

15. The line 293 states that “To access whether CD73 regulates the protein stability of CCL5”. It is not easy to understand how CD73 can affect the CCL5 stability. Please provide an example pathway to the text. Also, please provide a brief description of chloroquine and MG132 in the line 295.

16. The TCGA database analysis in Fig. S9A-H are interesting. Please provide information about which cancer type has been used in this analysis since this manuscript is about pancreatic cancer. This also apples to the Fig. S11.

17. Since the authors confirmed the STAT1 binding to the CCL5 locus by the manual ChIP not ChIP-seq, the selection criteria of the potential STAT1-binding region is critical. But the authors just state that “three predicted binding sites in the human CCL5 promoter region”. Please provide the rationale of the selection of these sites such as STAT1 binding motifs or high homology region.

18. Among the 4 cell lines, KPC, BXPC-3, Panc02, SW1990 which the authors used in Fig. 6, why BXPC-3 and SW1990 are chosen for the ChIP assays?

19. One of the complicating issues of research regarding chemokines such as CCL5 is the fact that the same chemokine can be used by both anti-inflammatory cells (Treg) or pro-inflammatory cells (CD8 T) or even from immune-regulatory cells such as dendritic cells. Therefore, the inhibition of a chemokine can have complicated results depending on the situations. Fig. 7 illustrates a very interesting point, but it should not be underestimated that the systemic inhibition of CCL5 can have multiple effects. This should be discussed in the Discussion section.

20. Fig. S10 requires statistical analysis with multiple repeats.

REVIEWER COMMENTS

Reviewer #1 (Remarks to the Author):

In the current manuscript, the authors investigate the role of membrane CD73, a critical enzyme in ATP metabolism, in pancreatic cancer. CD73 can be expressed on the cell surface, and this results in extracellular accumulation of adenosine which, in turn, inhibits the activity of infiltrating lymphocytes. In the current study, the authors explore a different expression of CD73, whereby membrane expression of this enzyme results in activation of the mapk38/Stat1 axis and, in turn, expression of CCL5, driving recruitment of regulatory T cells and suppression of CD4 and CD8+ T cell mediated anti-tumor immunity. The authors use genetic inactivation or overexpression of CD73 as well as pharmacological inhibition, and use different pancreatic cancer cell lines (KPC, PANC02, BXPC3) both in orthotopic and subcutaneous transplantation models. Overall, this article provides another mechanism of immunosuppression in pancreatic cancer, a disease characterized by lack of effective antitumor immunity and resistance to immunotherapy.

Response:

We deeply thank you for your recognition of our study. Indeed, this study may provide useful information for future clinical trials involving CD73 inhibition in wide range of patients. In addition, we would continue to focus on improving efficacy of immunotherapy in pancreatic cancer in future.

Main Comments:

The data supporting membrane expression of CD73 in human and mouse pancreatic cancer is limited to some immunohistochemistry and immunofluorescence staining; the data would be greatly strengthened by including co-IF with epithelial membrane

markers (K19, pan-CK…) and by showing high magnification images. Further, antibody specificity should be validated, at least in mouse, by staining in parallel tumor derived from CD73 KO cells and CD73 OE cells. Strong data supporting membrane expression is fundamental to justify the rationale of this study.

Response:

We deeply thank you for your critical and thoughtful comments. Actually, membrane expression of CD73 on tumor cells has been reported widely by previous studies (PMID: 28399672, PMID: 32514148). We have added results for CO-IF of pan-CK and CD73 in Figure S6B and S6D and in *Additional Figure 2A*. Antibodies used in this study were all commercial products from well-known companies, which has been validated by various studies. In this study, we validated the specificity of CD73 antibody using CD73 KO KPC cell line and tissue from CD73 depletion mice in Figure 4G and S6A-D.

While the notion that CD73 expression activated CCL5 expression is novel, the authors should determine whether there are changes on extracellular adenosine when CD73 is knocked out/overexpressed -and whether those changes cause the immune phenotype, in addition, or rather than CCL5 expression changes.

Response:

We deeply thank you for your critical and thoughtful comments. Actually, we have performed *in vivo* experiment that validated the immune phenotype is caused by CCL5 (Figure 7A-7R). Specifically, CCL5 blockade significantly inhibited CD73 overexpression-induced tumor progression with no influence on extracellular adenosine accumulation. In addition, injection of CCL5 largely abolished inhibitory effect of CD73 depletion on tumor growth without significant changes in extracellular adenosine concentration. These results determine that part of CD73 overexpression-induced

immune phenotype changes is extracellular adenosine independent but caused by the upregulation of CCL5.

The authors should explain how the cell lines for this study were selected. Was only one KPC cell line used? KPC cells vary greatly in terms of eliciting an immune response, and it is important to validate results across lines. In addition, Panc02 cells (derived from chemical carcinogenesis) are highly immunogenic, do not harbor the hallmark mutations of pancreatic cancer in the KRAS gene, and are generally not considered a valid model for the human disease. BxPC3 cells also have wild type KRAS, representing less than 5% of human tumors; they are thus an unusual choice when a single human line is used.

Response:

We deeply thank you for your critical and thoughtful comments. In this study, the KPC cell line obtained from the LSL-Kras G12D/+; LSL-Trp53 R172H/+; Pdx1-Cre mouse model was kindly provided by Prof. Raghu Kalluri (Department of Cancer Biology, Division of Basic Sciences, MD Anderson Cancer Center, Houston, TX, USA). This cell line has been widely used and validated in previous studies (e.g., PMID: 34315872, PMID: 35260434, PMID: 35288467). Although BxPC3 cells have wild type KRAS, we mainly focused on CD73/P38 MAPK/CCL5 axis in this study, in which KRAS was not directly involved. Moreover, BXPC-3 cells have also been widely used in previous studies focused on pancreatic cancer (e.g., PMID: 22490524, PMID: 12490654, PMID: 16361565, PMID: 12499282, PMID: 21350002). Although no direct evidence suggesting the influence of KRAS in previous studies, we have validated our findings using KRAS G12D mutated cancer cell SW1990 (Figure 3G, Figure 4G-H and *Additional Figure 1A-1G*) to exclude this possibility.

Minor concerns:

The flow data and CyTOF data do not match- the first shows an increase in CD8+ T cells while no change is observed in CyTOF. This inconsistency is not completely unusual but should be explained.

Response:

We deeply thank you for your critical and thoughtful comments. Indeed, inconsistency between flowcytometry and CyTOF is not completely unusual. However, there was no significant increase in CD8+ T cells in CD73 inhibition monotherapy group, comparing with control group (Figure 1D and 1I). Therefore, results from two experiments were consistent in this study (Figure 1D 1I and Figure 2B).

The Y axis of many panels in 2 are labeled as percentage but it is unclear what the total is: total cells? Immune cells? Total T cells?

Response:

We deeply thank you for your thoughtful comments and apologize for any insufficient description-caused misunderstanding. We have labeled the total cell as CD45+ immune cells (Figure 2B).

Page 12, last paragraph – the rationale for using chloroquine and MG132, and the biological effect of those drugs, needs to be explained.

Response:

We deeply thank you for your critical and thoughtful comments. We have added relevant description in the manuscript. CQ (Chloroquine) is a lysosome inhibitor that alters the acidic pH of lysosomes and inhibits the degradation of lysosomal protein.

MG132 is a potent proteasome inhibitor that suppresses the degradation of ubiquitin-conjugated proteins. The expression level of a protein can be regulated in multiple mechanisms ranging from transcription activation to post-translational degradation. In this study, we use CQ and MG132 to determine whether deregulation of CCL5 was caused by changes of CCL5 degradation.

There is a concern as to many of the findings being based on overexpression models. CCL5 expression should be examined in CD73 KO tumors as well.

Response:

We deeply thank you for your critical and thoughtful comments. We have performed additional experiments for CCL5 expression in CD73 KO *in vitro* models (Figure 4G) and *in vivo* models (Figure 4F).

Reviewer #2 (Remarks to the Author):

In the present studies, the authors provide evidence for a functional role of CD73 in promoting immune supression during pancreatic cancer which promotes disease progression. Their findings suggest that targeting CD73 with CD73 inhibitors in protective during pancreatic cancer. The studies are well done and their data interpretation is supported by ample data. I have some suggestions to further improve a strong manuscript:

Response:

We deeply thank you for your efforts, time and patience in evaluating this study and improving its quality. We have addressed all your concerns and revised the manuscript as you suggested.

Major comments:

1.) Can the author provide some evidence that there targeting approach resulted in attenuated adenosine production? The authors may consider to use APCP as an alternative approach to blocking CD73. They may find APCP more effective in dampening CD73 function than the antibody treatment.

Response:

We deeply thank you for your constructive suggestions. Indeed, AB680 is a highly potent, reversible and selective inhibitor of CD73, which has been validated in numerous studies (PMID: 32614585, PMID: 36409930, PMID: 34253638). In this study, AB680 were used for inhibition of CD73. We also agree with you that APCP is effective in dampening CD73 function. However, considering the consistent presentation of data, AB680 was mainly used for dampening CD73 function throughout the study.

2.) The authors may consider expanding their introduction to explain in more detail the functional role of CD73 in extracellular adenosine production (e.g. PMID: 23338058, PMID: 16799190 and PMID: 23263788) and it's role in modulating regulatory T-cell responses, including its function in inflammation (e.g. PMID: 23413361 and PMID: 22389701) versus cancer (PMID: 35360246).

Response:

We deeply thank you for your constructive suggestions. We have expanded our introduction in CD73 function in extracellular adenosine production and modulating immune responses.

3.) Can the authors provide some genetic evidence for a functional role of Adora2a signaling in their responses? Also, have the authors considered a role of the Adora2b receptor? This should be at least discussed in more details.

Response:

We deeply thank you for your constructive suggestions. We have performed additional experiments which demonstrated that Adora2a depletion completely abolished the ADO-induced upregulation of CCL5 at both the mRNA and protein levels (Figure S11E-F). We have also considered a role of the Adora2b receptor, and treated KPC and BXPC-3 cells overexpressing CD73 with inhibitor targeting Adora2b (CVT6883) (Figure 5H). We also agree with you that in some specific condition, adenosine may act on other receptors, including Adora1, Adora2b and Adora3, especially in hypoxia situation, which may possibly lead to the activation of distinct immunosuppressive pathways in different malignancies.

Minor comment:

1.) Can the authors expand in their introduction on more broadly introducing adenosine receptor signaling functions, e.g. include the heart rate slowing effects of Adora1 (e.g. PMID: 19707555) or Adora2b in ischemia and reperfusion (PMID: 23540714), and Adora2a during inflammation (PMID: 36009485). It would be helpful for the broad readership of this journal to give a more general introduction.

Response:

We deeply thank you for your constructive suggestions. We have expanded the introduction on more broadly introducing adenosine receptor signaling functions as you suggested.

2.) The authors may also discuss the functional roles of hypoxia in modulating CD73 and adenosine signaling (e.g. PMID: 28798196, PMID: 24391213), and also how this is important during pancreatic cancer.

Response:

We deeply thank you for your constructive suggestions. We have added discussion of functional roles of hypoxia in modulating CD73 and adenosine signaling as suggested.

3.) The authors may consider using the correct nomenclature for adenosine receptors - Adora1, Adora2a, Adora2b, Adora3.

Response:

We deeply thank you for your constructive suggestions. We have changed the nomenclature for adenosine receptors as you suggested.

Reviewer #3 (Remarks to the Author):

Tang et. al. show that CD73 on pancreatic tumor cells transcriptionally regulates CCL5 through STAT1 which results in the increased recruitment of immune-suppressive Treg cells to the tumor sites, thus facilitating tumor immune escape. The manuscript touches an important and interesting issue and proves the main claims in a well-designed way. CCL5 has been traditionally seen as a pro-tumor molecule, but the recent literatures have shown an anti-tumor molecule side of CCL5. This manuscript also illustrates the anti-tumor function of CCL5, thus facilitating more research on CCL5 field. The manuscript is even more interesting in that it provides a new mechanism of CCL5 regulation by a metabolic enzyme CD73, thus connecting a metabolic pathway with a chemokine expression.

Response:

We deeply thank you for your recognition of our study. Indeed, role of CCL5 plays in various malignancies is still complicated. CCL5 can used by various immune cells and inhibition of CCL5 can have different results depending on the situations. In our study, we found that overexpression of CD73 on pancreatic tumor cells induces transcription of CCL5 and CCR5 was preferentially expressed by infiltrated CD4+ Foxp3+ Tregs in pancreatic cancer, which may lead to the significant inhibition effect of CCL5 blockade in pancreatic cancer. This study may provide useful information for future clinical trials involving CCL5 inhibition in wide range of patients. In addition, we would continue to focus on improving efficacy of immunotherapy in pancreatic cancer in future.

However, this reviewer feels uncomfortable to recommend the publication of this manuscript at Nat. Commnun. with the current status. As this reviewer explains below with 3 major points, overall, the way of data presentation and the quality check of the manuscript do not meet the criteria and standard of this journal (please see the Major points 1 and 2). Also, this reviewer asks little more experiments (if the authors do not possess such data already) to clearly illustrate the important point of the receptor side of the story (please see the Major point 3).

Response:

We deeply thank you for your efforts, time and patience in evaluating this study and improving its quality. We have addressed all your concerns and revised the manuscript as you suggested.

MAJOR POINTS

1. The Fig. 1 sets the scientific basis of this manuscript by testing the combinatorial immunotherapies with CD73 and PD-1 inhibition. The results are interesting, convincing and persuading, but this reviewer does not feel comfortable with inconsistent presentation of data throughout the manuscript. Some but not all examples are shown below.

Response:

We deeply thank you for your critical comments and sincerely apologize for any inconsistent presentation of data throughout the manuscript. We have revised the manuscript and performed more experiments as suggested.

a. For CD73 inhibition, an anti-CD73 antibody is used in panels A-E, meanwhile a CD73 inhibitor is used in panels F-K. While the panels A-K are done in ortholog implantation, additional data on non-ortholog implantation are presented in panels J-N. But in each case, the analysis methods are presented inconsistently without providing apparent reasons.

Response:

We deeply thank you for critical comments and sincerely apologize for any inconsistent presentation of data. We have additionally performed *in vivo* experiments, in which mice with orthotopic tumors were treated with or without CD73 inhibitor and PD-1 blockade (Figure 1A-D) to address your concerns.

b. For example, with the anti-CD73 antibody inhibition experiment, Luciferase signals (B) and tumor weights (C), mice weight (D), T cell composition (E) and POS (F) are presented. But with the CD73 inhibitor experiment, Luciferase signals and POS are not

shown, but instead tumor volume (H) are additionally presented which is not shown for the anti-CD73 antibody experiment.

Response:

We deeply thank you for your critical and thoughtful comments. Indeed, in vivo imaging (measured by bioluminescence imaging system) and tumor volume (measured by caliper, tumor volume=length × width2 × 0.5) were both used for monitoring tumor growth. Caliper provides more direct and accurate tumor growth measurements as compared to bioluminescent imaging in subcutaneous model. In orthotopic tumor model, tumors were inaccessible to traditional caliper measurement. Therefore, those tumors were evaluated using bioluminescence imaging without direct measurement. In addition, orthotopic tumor models provide a clinically relevant, organ-specific tumor microenvironment. Therefore, we choose orthotopic tumor models for survival analysis of combination immunotherapy.

c. As another example, in Fig.1 legend, panels A-E are indicated to use the orthotopic model. However, it is not clear what model the F panel is using from the figure legend. By reading the main text, this reviewer can guess that the panel F must be the orthotopic model. Therefore. the figure legend should say that the panels A-F not A-E are the orthotopic model. Then, the following panels G-K do not have any description of the models used. Again, by reading the main text, it appears that panels G-K must be the subcutaneous model.

Response:

We deeply thank you for your critical comments and sincerely apologize for any insufficient description-caused misunderstanding. We have additionally highlighted the specific model used in figure legend as suggested.

d. Also, Fig. 2 CyTOF analysis uses the CD73 inhibitor with the orthotopic model. Since Fig. 1 used anti-CD73 antibody or inhibitor with the orthotopic or subcutaneous model, we need a specific reason for the choice of the CD73 inhibitor with the orthotopic model in Fig. 2.

Response:

We deeply thank you for your thoughtful comments and sincerely apologize for any inconsistent presentation of data. Indeed, both anti-CD73 antibody and inhibitor were highly effective options for treating mice with pancreatic cancer. We have additionally performed *in vivo* experiments in which mice with orthotopic tumors were treated with or without CD73 inhibitor and PD-1 blockade (Figure 1A-D) to address your concerns.

e. Fig. 3J is in vivo experiment encompassing 3 weeks which makes possible that Treg could have proliferated more rather than just migrated more. Of course, this point has been addressed in the chemotaxis assay (Fig. 3G-I), however, these experiments are done with mouse Treg. Therefore, it is critical to perform the same chemotaxis assay with the human Treg cells to claim that CD73 on tumor cells affect the migration not the proliferation of human Treg cells. Please provide an explanation of different uses of Tregs (mouse or human origin) in panels Fig.3 G-J.

Response:

We deeply thank you for your critical and thoughtful comments. We have performed the same chemotaxis assay with the human Treg cells as suggested (Figure 3G). In addition, we have re-performed the human Treg *in vivo* assay and harvested within one week after tail-vein injection of human Treg to address your concerns (Figure 3H).

f. In the in vivo experiments, BXPC-3 tumor is used in Fig. 3J, but KPC tumor is used in Fig. 3K. Also, Fig. 3J uses the orthotopic model, but the subcutaneous model is used in Fig. 3K. not explained, and the tumor volume/weight is measure only with KPC but not with BXPC-3.

Response:

We deeply thank you for your critical comments and sincerely apologize for any insufficient description-caused misunderstanding. For human Treg *in vivo* assays, experiments were performed on nude mice to avoid interference from mouse Tregs. Since nude mice lack normal immune system, this model only used to determine the difference in human Treg infiltration between WT and CD73 KO BXPC-3 tumors. Therefore, volume and weight of tumors were not measured. In addition, we have performed the *in vivo* mouse Tregs depletion assays in orthotopic KPC model to keep experiments consistent (Figure 3I-M).

The authors use two inhibition methods, two mouse models, different tumor cell lines. Therefore, it is crucial to clearly indicate what is used in each panel throughout the manuscript (especially Fig. 1-3). More importantly, please provide the explanation or rationale about these inconsistent uses of methods and models and cells throughout the manuscript.

Response:

We deeply thank you for thoughtful comments and sincerely apologize for any inconsistent presentation of data. We have added more detailed experiments and presented the data in a more consistent way.

2. In Fig. 2C, the decreases of PD-1, TIM3, FoxP3 are observed and this becomes one of the main claims of this manuscript. However, the most significantly decreased cluster appears to be the C03 cluster which unfortunately has no assignment of a cell lineage. C03 does not appear to be lymphocytes since C03 has no CD3e, CD4 or CD8 expression in Fig S3A, while having expression of NK1.1 and FoxP3. Can the authors elaborate about the potential identity of this NK1.1 high and FoxP3 high C03 cluster? Why does C03 have the highest FoxP3 even though C03 has no CD4 expression? Due to this strange tSNE clustering, this reviewer cannot be confident that this unsupervised clustering has been properly performed. This makes one of the major claims of the manuscript does not hold the stand. Therefore, this CyTOF results of Fig. 2 does not fully support the conclusion of Fig 1 (which stresses that the inhibitory effects of the combination therapy is completely T-cell dependent (Lines 194-197).

Response:

We deeply thank you for your critical and thoughtful comments. We agree with you that tSNE clustering should be improved. We have re-do the QC (quality control) procedure and re-analyzed the results (Figure 2A-C and Figure S5A) to address your concerns. The PD-1, TIM3, FoxP3 and TIGIT were significantly decreased in T cell clusters.

3. The main theme of the manuscript is the tumor-infiltration of Treg by the CCL5 production by tumor cells through CD73. Therefore, the migration/infiltration of Treg cells as well as other lineage cells needs to be examined more thoroughly.

a. The authors performed blocking CCL5 to inhibit Treg migration in vitro (Fig. 4O) and in vivo (Fig. 7). It should be considered that not all Treg cells express CCR5, one of the major cognate receptors of CCL5. Therefore, it is crucial to show the expression

of CCR5 on Tregs and CD8 T cells. This reviewer strongly asks to examine the CCR5 expression levels on the Treg cells used in the chemotaxis assay (Fig. 4O) as well as both Treg and CD8 T cells of the in vivo assays (Fig. 7D).

Response:

We deeply thank you for your thoughtful comments and constructive suggestions. We have examined the CCR5 expression levels on the Treg cells used in the chemotaxis assay (Figure S9E) as well as both Treg and CD8 T cells of the *in vivo* assays (Figure7H and Figure7Q) as suggested.

b. The effect of tumor inhibition cannot be judged from a single-time point. Since Fig. 7 has an important position in the manuscript, the reviewer strongly asks to show the tumor volume graph with multiple time points for Fig. 7 experiments as in Fig. 1H.

Response:

We deeply thank you for your thoughtful comments and constructive suggestions. However, in orthotopic tumor model, tumors were inaccessible to traditional caliper measurement. Therefore, those tumors were evaluated using bioluminescence imaging instead (Figure 7B and 7K). We have re-performed the experiments in Figure 7 and presented more detailed results.

c. CCL5 is expressed mainly by macrophages, NK cells and activated CD8 T cells, not only tumor cells. It will be necessary to stain CCL5 of TIL samples to show and compare CCL5 expression in a more systemic way in Fig. 7.

Response:

We deeply thank you for your thoughtful comments and constructive suggestions. We have stained CCL5 of various immune subsets and compare CCL5 expression in a more systemic way (Figure 4F and Figure S9D) as suggested.

MINOR POINTS

1. Page 6 lines 122-124 in the Introduction tells the most important part of the Introduction section since the rationale of the research aim is presented. However, this description can be comprehended in a wrong way, so it could be rewritten to better convey the concise meaning of “immunosuppressive pathways inside tumor cells”.

Response:

We deeply thank you for your thoughtful comments and constructive suggestions. We have rewritten the sentence as suggested.

2. In Fig. S1C, most CD8+ cells are TNFa or IFNg single positive. From the experience of the reviewer with B16F10 and MC38 tumor-burden models, many CD8+ cells become double positive for TNFa and IFNg, especially after PD-1 treatment. Is this phenomenon common for KPC cells?

Response:

We deeply thank you for your thoughtful comments. Indeed, this phenomenon common for KPC cells. In the revised manuscript, we have re-analyzed the infiltration and activation of TILs (Figure 1D and 1I and Figure S3).

3. The figure legends need more attention to clarify which mouse model (orthotopic or subcutaneous). For example, the model used by panel G of Fig. 1 is not described in

the main text nor the figure legend. Also, the Figure 2 legend does not state the model used either.

Response:

We deeply thank you for your thoughtful comments and constructive suggestions. We have clarified the mouse model in figure legends as suggested.

4. Fig 1N highlights the superior tumor-suppressing activity of CD4 T cells which is a very interesting observation. Since the Fig S2 only show the percentages of CD8 T cells, inclusion of CD4 T cell percentages in Fig S2 should be included to properly access the outcome of Fig 1N. Also, FoxP3 staining in TILs might be necessary to support the claim of Fig 2.

Response:

We deeply thank you for your thoughtful comments and constructive suggestions. We have provided more detailed results of infiltration and activation of CD4+ T cells as your suggested (Figure 1D and 1I, and Figure S3A and S3C). However, FoxP3 staining in TILs in Figure 1 might make CyTOF analysis (Figure 2A-D) meaningless, as one of the main claims of figure 2 is the decrease of Tregs. Therefore, we prefer to focus on Tregs in Figure 2.

5. Fig. 2A shows several populations, but the names are not consistent in the uses of plurals. For example, Macrophage and DC can be Macrophages and DCs to be consistent with the remaining.

Response:

We deeply thank you for your thoughtful comments and constructive suggestions. We have revised the Figure 2A as suggested.

6. Fig. 2B illustrates that there are no statistically significant changes in the cell population after CD73 inhibition. However, there are very strong presence of subtypes within Macrophages and Granulocytes (just below the names of the populations) which disappear after CD73 inhibition. Do the authors have any insight on these populations? This can be noted similarly to Fig. 2C.

Response:

We deeply thank you for your critical and thoughtful comments. Indeed, there are changes within other immune subtypes including Macrophages and Granulocytes, which may play critical roles in immune suppressive microenvironment in pancreatic cancer. Further studies are required to elucidate the underlying mechanism contributing to those changes.

7. The last graph of Fig. 2C says "TIGHT". Is this TIGIT? If so, please recheck spelling errors throughout the manuscript which is quite abundant. Also, If this is the exhaustion marker TIGIT, it should be mentioned in the main text or at least in the figure legend even if it is statistically not significant.

Response:

We deeply thank you for your critical comments and sincerely apologize for any spelling errors throughout the manuscript. We have rechecked and corrected the spelling errors throughout the manuscript, and added TIGIT in the manuscript.

8. T cell compartments in Fig. S3B show a quite high expression of MHCII and Ly6c which are typically associated with B cells or myeloid lineages. Can the authors explain this peculiar expression?

Response:

We deeply thank you for your critical and thoughtful comments. Indeed, previous studies demonstrated that MHCII distribution on B cells may be determined by ubiquitin chain length (PMID:22566640). In comparison to MHC II− B cells, MHC II+ B cells had a substantial advantage in proliferation and differentiation (PMID: 29715484). In addition, previous study demonstrated Ly6c distribution on B cells, which was associated with treatment effect in varying diseases (PMID: 11754008, PMID: 35222390).

9. I might have missed, but where the information about the CD73 KO mouse?

Response:

We deeply thank you for your thoughtful comments. Indeed, we have provided information about the CD73 KO mouse in methods. The CD73 KO mouse was also labeled for gene name as NT5E KO mouse.

10. Fig. S4 legend put the description of G-J ("Blue dots: DAPI, Green dots: EdU") into C-F description area.

Response:

We deeply thank you for your thoughtful comments. We have corrected the legends of figures as you suggested (Figure S7A-S7H).

11. The colors of Fig.3 M-P does not match Fig. 3L, thus confusing.

Response:

We deeply thank you for your critical and thoughtful comments. We have changed the colors of figures and presented the data more consistent as your suggested (Figure 3I-3M).

12. Fig. S5C shows the activation status of CD8 T cells by IFNg. However, the small increase from 22% to 28% makes the conclusion from Fig. S5 less convincing. This reviewer asks to perform multiple repeats with the statistical analysis on all the data on Fig. S5.

Response:

We deeply thank you for your critical and thoughtful comments. We have performed multiple repeats with the statistical analysis as suggested (Figure S8B- S8C).

13. Fig. 4 A-C examines chemokine expression from CD73 KO tumor cells and D-G from CD73 OE tumor cells according to the figure, but the main text says the opposite. Which one is right?

Response:

We deeply thank you for your thoughtful comments, and sincerely apologize for the incorrect description. We have corrected relevant statements in the main text.

14. The second important message of this manuscript is the changes in CCL5 levels from tumor cells with CD73 expression. The authors examined 25 chemokines, but only several ones are shown in Fig. 4B. Since this is an important data, the review insists to show all 25 chemokines.

Response:

We deeply thank you for your thoughtful comments. Indeed, the chemokine arrays we used incorporated 25 chemokines. However, few chemokines in supernatant from pancreatic cell lines failed to be detectable due to the extremely low concentrations. Eventually, 22 chemokines were detected in supernatant from KPC cell lines and 21 chemokines were detected in supernatant from PANC02 cell lines. We have showed all of chemokines detected in revised figures (Figure 4B and Figure S9B).

15. The line 293 states that “To access whether CD73 regulates the protein stability of CCL5”. It is not easy to understand how CD73 can affect the CCL5 stability. Please provide an example pathway to the text. Also, please provide a brief description of chloroquine and MG132 in the line 295.

Response:

We deeply thank you for your thoughtful comments. CQ (Chloroquine) is a lysosome inhibitor that alters the acidic pH of lysosomes and inhibits lysosomal protein degradation. MG132 is a potent proteasome inhibitor that reduces the degradation of ubiquitin-conjugated proteins. The expression level of certain protein can be regulated in multiple mechanisms ranging from transcription activation to post-translational degradation. In this study, we use CQ and MG132 to determine whether deregulation of CCL5 was caused by changes of CCL5 degradation.

16. The TCGA database analysis in Fig. S9A-H are interesting. Please provide information about which cancer type has been used in this analysis since this manuscript is about pancreatic cancer. This also apples to the Fig. S11.

Response:

We deeply thank you for your thoughtful comments and constructive suggestions. These TCGA database analysis were all conducted using pancreatic cancer data (Figure S13A-H and Figure S15A-C).

17. Since the authors confirmed the STAT1 binding to the CCL5 locus by the manual ChIP not ChIP-seq, the selection criteria of the potential STAT1-binding region is critical. But the authors just state that "three predicted binding sites in the human CCL5 promoter region". Please provide the rationale of the selection of these sites such as STAT1 binding motifs or high homology region.

Response:

We deeply thank you for your thoughtful comments and constructive suggestions. We have provided the rationale of the selection of these sites in main text and provided STAT1 binding motifs as suggested (Figure S13I).

18. Among the 4 cell lines, KPC, BXPC-3, Panc02, SW1990 which the authors used in Fig. 6, why BXPC-3 and SW1990 are chosen for the ChIP assays?

Response:

We deeply thank you for your critical and thoughtful comments. Indeed, STAT1 binding sites prediction of CCL5 promoter was conducted based on NCBI and JASPAR online database, which was mainly focused on human TFs. In fact, both TF motifs and promoter sequences vary greatly between distinct species, which limits the value for ChIP assays based on mouse cell lines. Therefore, among the 4 cell lines, two human pancreatic cancer cell lines, BXPC-3 and SW1990, were chosen for further CHIP assays, which may provide valuable information for further clinical study.

19. One of the complicating issues of research regarding chemokines such as CCL5 is the fact that the same chemokine can be used by both anti-inflammatory cells (Treg) or pro-inflammatory cells (CD8 T) or even from immune-regulatory cells such as dendritic cells. Therefore, the inhibition of a chemokine can have complicated results depending on the situations. Fig. 7 illustrates a very interesting point, but it should not be underestimated that the systemic inhibition of CCL5 can have multiple effects. This should be discussed in the Discussion section.

Response:

We deeply thank you for your thoughtful comments and constructive suggestions. Indeed, role of CCL5 plays in various malignancies is still complicated. CCL5 can used by various immune cells and inhibition of CCL5 can have different results depending on the situations. In our study, we found that overexpression of CD73 on pancreatic tumor cells induces transcription of CCL5 and CCR5 was preferentially expressed by infiltrated CD4+ Foxp3+ Tregs in pancreatic cancer, which may lead to the significant inhibition effect of CCL5 blockade in pancreatic cancer. We have additionally discussed the multiple effects of inhibition of CCL5 in the Discussion section as suggested.

20. Fig. S10 requires statistical analysis with multiple repeats.

Response:

We deeply thank you for your thoughtful comments and constructive suggestions. We have added statistical analysis with multiple repeats as suggested.

REVIEWERS' COMMENTS

Reviewer #1 (Remarks to the Author):

The authors have addressed my previous concerns.

Reviewer #2 (Remarks to the Author):

Thank you for considering my suggestions.

Reviewer #3 (Remarks to the Author):

The way of data presentation in Figure 1 and 2 has been greatly improved for better presentation, and the Figures 1 and 2 are now in an excellent shape. The inclusion of human Treg in Figure 3 also has nicely clarified my concern about the inconsistent presentation. Therefore, I have no more issues with my major point 1.

The reanalysis of CyTof in Figure 2 now nicely supports the main claim of the manuscript. Therefore, I have no more issues with my major point 2.

The more detailed analysis on CCR5 and CCL5 in Figure 7 now nicely address this reviewer's concerns. The CCR5 and CCL5 expression supports the author's claim very well (as a minor note, CCR5 expression was shown in Figure 7I and 7O, not 7H and 7Q). Therefore, I have no more issues with my major point 3.

All of my minor points are well addressed from the revised manuscript except minor point 17. If Figure S13I indicates the STAT1 binding motifs from the transcriptional start site (TSS), Motif 1 and 3 might be enhancers not promoters. If so, the authors might state "the promoter and enhancer of CCL5" not just "the promoter of CCL5" throughout the manuscript. This is just a suggestion not request.

Overall, the manuscript is in a much refined and improved shape by this revision. The manuscript will greatly contribute to this field. I appreciate the efforts put into this revision by the authors.

REVIEWERS' COMMENTS

Reviewer #1 (Remarks to the Author):

The authors have addressed my previous concerns.

Response:

We deeply thank you for your efforts, time and patience in evaluating this study and improving its quality. Your suggestions and feedback were instrumental in improving our manuscript.

Reviewer #2 (Remarks to the Author):

Thank you for considering my suggestions.

Response:

We deeply thank you for your efforts, time and patience in evaluating this study and improving its quality. We are grateful for your expertise and attention to our research.

Reviewer #3 (Remarks to the Author):

The way of data presentation in Figure 1 and 2 has been greatly improved for better presentation, and the Figures 1 and 2 are now in an excellent shape. The inclusion of human Treg in Figure 3 also has nicely clarified my concern about the inconsistent presentation. Therefore, I have no more issues with my major point 1.

Response:

We deeply thank you for your efforts, time and patience in evaluating this study and improving its quality. Your suggestions helped us to gain a deeper understanding of our findings and will make our research more credible and persuasive.

The reanalysis of CyTof in Figure 2 now nicely supports the main claim of the manuscript. Therefore, I have no more issues with my major point 2.

Response:

We deeply thank you for your efforts, time and patience in evaluating this study and improving its quality. Your suggestions helped us to gain a deeper understanding of our

findings and will make our research more credible and persuasive.

The more detailed analysis on CCR5 and CCL5 in Figure 7 now nicely address this reviewer’s concerns. The CCR5 and CCL5 expression supports the author’s claim very well (as a minor note, CCR5 expression was shown in Figure 7I and 7O, not 7H and 7Q). Therefore, I have no more issues with my major point 3.

Response:

We deeply thank you for your efforts, time and patience in evaluating this study and improving its quality. Your suggestions helped us to gain a deeper understanding of our findings and will make our research more credible and persuasive.

All of my minor points are well addressed from the revised manuscript except minor point 17. If Figure S13I indicates the STAT1 binding motifs from the transcriptional start site (TSS), Motif 1 and 3 might be enhancers not promoters. If so, the authors might state “the promoter and enhancer of CCL5” not just “the promoter of CCL5” throughout the manuscript. This is just a suggestion not request.

Response:

We deeply thank you for your constructive suggestions. We have changed the “the promoter of CCL5” to “the promoter and enhancer of CCL5” as you suggested.

Overall, the manuscript is in a much refined and improved shape by this revision. The manuscript will greatly contribute to this field. I appreciate the efforts put into this revision by the authors.

Response:

We deeply thank you for your efforts, time and patience in evaluating this study and improving its quality. Your review and suggestions have had a profound impact on our research and will help us to better serve this field in future studies.
